# Adjuvant nivolumab in resected stage IIB/C melanoma: primary results from the randomized, phase 3 CheckMate 76K trial

**DOI:** 10.1038/s41591-023-02583-2

**Published:** 2023-10-16

**Authors:** John M. Kirkwood, Michele Del Vecchio, Jeffrey Weber, Christoph Hoeller, Jean-Jacques Grob, Peter Mohr, Carmen Loquai, Caroline Dutriaux, Vanna Chiarion-Sileni, Jacek Mackiewicz, Piotr Rutkowski, Petr Arenberger, Gaelle Quereux, Tarek M. Meniawy, Paolo A. Ascierto, Alexander M. Menzies, Piyush Durani, Maurice Lobo, Federico Campigotto, Brian Gastman, Georgina V. Long

**Affiliations:** 1 UPMC Hillman Cancer Center, Pittsburgh, PA USA; 2 Fondazione IRCCS Istituto Nazionale dei Tumori, Milan, Italy; 3 NYU Langone Medical Center, New York, NY USA; 4 Medizinische Universität Wien, Vienna, Austria; 5 Hôpital de la Timone, Marseille, France; 6 Elbe Klinikum Buxtehude, Buxtehude, Germany; 7 University Medical Center Mainz, Mainz, Germany; 8 Hôpital Saint André, Bordeaux, France; 9 Istituto Oncologico Veneto, IOV-IRCCS, Padova, Italy; 10 Institute of Oncology, Poznan University of Medical Sciences, Poznan, Poland; 11 Maria Skłodowska-Curie National Research Institute of Oncology, Warsaw, Poland; 12 Charles University Third Faculty of Medicine and University Hospital Královské Vinohrady, Prague, Czech Republic; 13 Nantes University Hospital, Nantes, France; 14 University of Western Australia and Sir Charles Gairdner Hospital, Perth, WA Australia; 15 Istituto Nazionale Tumori IRCCS ‘Fondazione G. Pascale’, Naples, Italy; 16 Melanoma Institute Australia, University of Sydney, and Royal North Shore and Mater Hospitals, Sydney, NSW Australia; 17 Bristol Myers Squibb, Princeton, NJ USA; 18 Cleveland Clinic, Cleveland, OH USA

**Keywords:** Melanoma, Cancer immunotherapy

## Abstract

Patients with resected stage IIB/C melanoma have high recurrence risk, similar to those with resected stage IIIA/B disease. The phase 3, double-blind CheckMate 76K trial assessed 790 patients with resected stage IIB/C melanoma randomized 2:1 (stratified by tumor category) to nivolumab 480 mg or placebo every 4 weeks for 12 months. The primary endpoint was investigator-assessed recurrence-free survival (RFS). Secondary endpoints included distant metastasis-free survival (DMFS) and safety. At 7.8 months of minimum follow-up, nivolumab significantly improved RFS versus placebo (hazard ratio (HR) = 0.42; 95% confidence interval (CI): 0.30–0.59; *P* < 0.0001), with 12-month RFS of 89.0% versus 79.4% and benefit observed across subgroups; DMFS was also improved (HR = 0.47; 95% CI: 0.30–0.72). Treatment-related grade 3/4 adverse events occurred in 10.3% (nivolumab) and 2.3% (placebo) of patients. One treatment-related death (0.2%) occurred with nivolumab. Nivolumab is an effective and generally well-tolerated adjuvant treatment in patients with resected stage IIB/C melanoma. ClinicalTrials.gov identifier: NCT04099251.

## Abstract (web summary)

In this pre-specified interim analysis, patients with resected stage IIB/C melanoma who received adjuvant nivolumab had significantly prolonged recurrence-free survival compared to placebo-treated patients, providing another treatment option for this population.

## Main

The incidence of melanoma is rising worldwide, and patients with node-negative stage IIB/C disease comprise a large population at significant risk of recurrence^1–4^. Per the American Joint Committee on Cancer (AJCC) Cancer Staging Manual, 8th edition, patients with stage IIB disease have primary tumors that are >2 mm and ≤4 mm thick with ulceration (T3b) or >4 mm thick without ulceration (T4a), whereas patients with stage IIC disease have primary tumors >4 mm thick with ulceration (T4b) (ref. ^4^). Although stage II melanoma is less advanced than stage III or stage IV disease, stage II disease is associated with a greater absolute number of eventual deaths due to its far greater incidence^3,5^. Real-world studies evaluating patients before the approval of adjuvant checkpoint inhibitors suggest that the 5-year risk of recurrence in patients with stage IIB or stage IIC disease is approximately 35% and 50%^3^, respectively, with 5-year melanoma-specific survival (MSS) rates of 83–87% for IIB and 70–82% for IIC. MSS rates in patients with stage IIB/IIC disease are similar to those for stage IIIA (93%), stage IIIB (83%) and stage IIIC (69%) disease^4,6^.

Checkpoint inhibitors have transformed the adjuvant treatment landscape of resectable stage III and stage IV melanoma^7–11^, prompting investigation in earlier stages of disease^2^. Data from the phase 3 KEYNOTE-716 trial led to US Food and Drug Administration and European Medicines Agency approval of adjuvant pembrolizumab in patients with resected stage IIB/C melanoma, where treatment with pembrolizumab improved the recurrence-free survival (RFS) compared with placebo (hazard ratio (HR) = 0.65; 95% confidence interval (CI): 0.46–0.92) (ref. ^12^).

Here we present a pre-specified interim analysis of the ongoing, randomized, double-blind, phase 3 CheckMate 76K trial evaluating nivolumab versus placebo as adjuvant treatment for patients with resected stage IIB/C melanoma, including the primary analysis of RFS.

## Results

### Patients and exposure

**Fig. 1:**
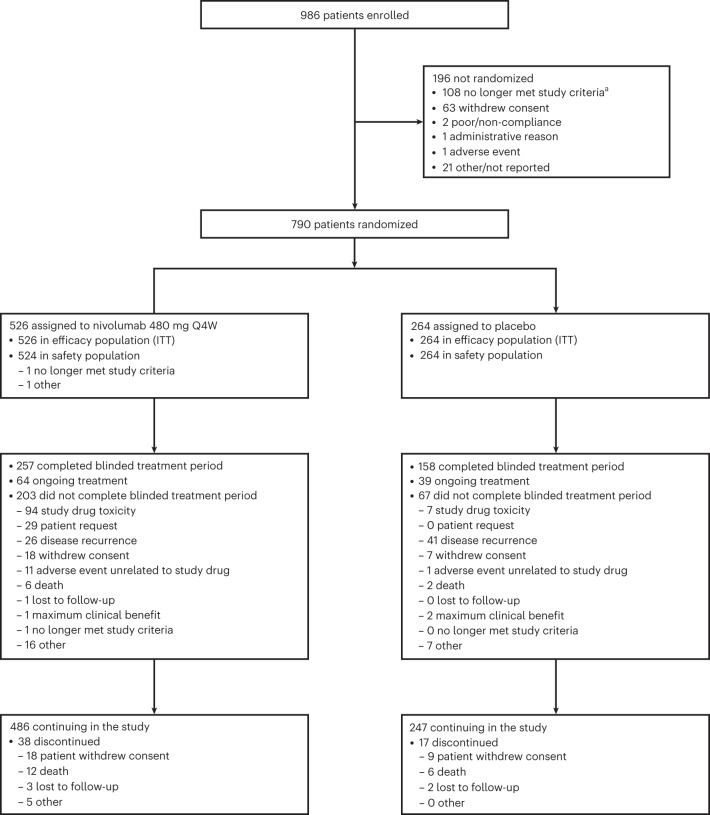
CONSORT flow diagram. ^a^The most common reasons for no longer meeting study criteria included change in staging or residual disease on pathology (*n* = 24); recurrence, disease progression or metastasis before randomization (*n* = 16); lack of or missing baseline imaging before randomization (*n* = 14); and diagnosis of a second tumor type within 3 years before enrollment (*n* = 10). The remaining 44 patients no longer met study criteria for other varied reasons, including non-compliance with study procedures, disqualifying comorbidities and laboratory abnormalities. ITT, intention-to-treat.

From 28 October 2019 through 3 November 2021, 986 patients in 20 countries worldwide were screened at 129 sites, and 790 were randomized 2:1 to receive nivolumab (526 patients) or placebo (264 patients) at 119 sites (Fig. 1); two patients in the nivolumab group did not receive study treatment because they no longer met study criteria (0.2%, 1/526) or because of other reasons (0.2%, 1/526). Patient characteristics at baseline were well balanced between treatment groups. Most patients (58.6%, 463/790) were from Western Europe; 50.5% (399/790) had nodular melanoma; and 39.4% (311/790) had stage IIC disease (Table 1). At the data cutoff date of 28 June 2022, there was an overall minimum follow-up (defined as, time between last patient randomized and data cutoff) of 7.8 months for all patients and a median follow-up (defined as, median time between randomization date and death or last known alive date) of 15.8 months (interquartile range (IQR): 11.9–20.3) and 15.9 months (IQR: 12.0–20.4) for the nivolumab group and the placebo group, respectively. Of the patients treated with nivolumab, 49.0% (257/524) completed blinded phase treatment and 12.2% (64/524) were still on treatment; for placebo, this was 59.8% (158/264) and 14.8% (39/264), respectively (Fig. 1). The most common reasons for discontinuation were study drug toxicity in 17.9% (94/524) of patients treated with nivolumab and 2.7% (7/264) of patients treated with placebo or disease recurrence in 5.0% (26/524) and 15.5% (41/264) of patients, respectively (Fig. 1). Although discontinuations due to patient request (5.5% (29/524) of patients treated with nivolumab versus 0% of patients treated with placebo) were mainly related to logistical issues with the trial for patients or patient decisions, the contribution of potential low-grade adverse events on some of these discontinuations cannot be ruled out. Patients received a median of 12 doses (range, 1–14) of nivolumab for a median duration of 11.0 months (range, 0–12.1) and a median of 13 doses (range, 1–14) of placebo for a median duration of 11.1 months (range, 0–12.7) (Supplementary Table 1). Subsequent therapy was received by 9.5% (50/526) of patients in the nivolumab group and 23.5% (62/264) of patients in the placebo group, including surgery in 6.8% and 14.8% of patients and systemic therapy in 5.7% and 18.6% of patients, respectively (Supplementary Table 2).

**Table 1:** Baseline patient demographics and disease characteristics

Characteristic^a^	Nivolumab, 480 mg Q4W (*n* = 526)	Placebo, Q4W (*n* = 264)	Total (*n* = 790)
**Median age (range)**, **year**	62 (21–87)	61 (19–92)	62 (19–92)
**Sex**, ***n*** **(%)**			
Male	322 (61.2)	161 (61.0)	483 (61.1)
Female	204 (38.8)	103 (39.0)	307 (38.9)
**ECOG PS**, ***n*** **(%)**			
0	495 (94.1)	245 (92.8)	740 (93.7)
1	31 (5.9)	19 (7.2)	50 (6.3)
**Disease stage**, ***n*** **(%)**			
IIB	316 (60.1)	163 (61.7)^b^	479 (60.6)
IIC	210 (39.9)	101 (38.3)	311 (39.4)
**T category**, ***n*** **(%)**			
T3b	204 (38.8)	104 (39.4)	308 (39.0)
T4a	112 (21.3)	58 (22.0)	170 (21.5)
T4b	210 (39.9)	102 (38.6)	312 (39.5)
**Melanoma subtype**, ***n*** **(%)**			
Superficial spreading	151 (28.7)	82 (31.1)	233 (29.5)
Nodular	266 (50.6)	133 (50.4)	399 (50.5)
Lentigo	13 (2.5)	3 (1.1)	16 (2.0)
Acral lentiginous	28 (5.3)	15 (5.7)	43 (5.4)
Desmoplastic	21 (4.0)	8 (3.0)	29 (3.7)
Other	44 (8.4)	22 (8.3)	66 (8.4)
Not reported	3 (0.6)	1 (0.4)	4 (0.5)
**Location of primary tumor**, ***n*** **(%)**			
Trunk	193 (36.7)	89 (33.7)	282 (35.7)
Leg	116 (22.1)	59 (22.3)	175 (22.2)
Arm	109 (20.7)	58 (22.0)	167 (21.1)
Head and neck	108 (20.5)	58 (22.0)	166 (21.0)
**Lactate dehydrogenase**, ***n*** **(%)**			
≤ULN	470 (89.4)	232 (87.9)	702 (88.9)
>ULN	50 (9.5)	25 (9.5)	75 (9.5)
Not reported	6 (1.1)	7 (2.7)	13 (1.6)
***BRAF***^**v600**^ **mutation status**, ***n*** **(%)**			
Mutant	148 (28.1)	81 (30.7)	229 (29.0)
Wild-type	293 (55.7)	136 (51.5)	429 (54.3)
Not evaluable/not reported	85 (16.2)	47 (17.8)	132 (16.7)
**PD-L1 expression**, ***n*** **(%)**			
≥1%	109 (20.7)	58 (22.0)	167 (21.1)
<1%	80 (15.2)	53 (20.1)	133 (16.8)
Not evaluable/not reported	337 (64.1)^c^	153 (58.0)	490 (62.0)
**Geographic region**, ***n*** **(%)**			
US and Canada	97 (18.4)	46 (17.4)	143 (18.1)
Western Europe	303 (57.6)	160 (60.6)	463 (58.6)
Eastern Europe	58 (11.0)	28 (10.6)	86 (10.9)
Australia	68 (12.9)	30 (11.4)	98 (12.4)

^a^Percentages may not total 100 because of rounding.

^b^One of these patients was recategorized as having stage IIC disease after data cutoff.

^c^Two of these patients were recategorized as having <1% PD-L1 expression after data cutoff.

ECOG PS, Eastern Cooperative Oncology Group performance status; Q4W, every 4 weeks; ULN, upper limit of normal.

### Efficacy

**Fig. 2:**
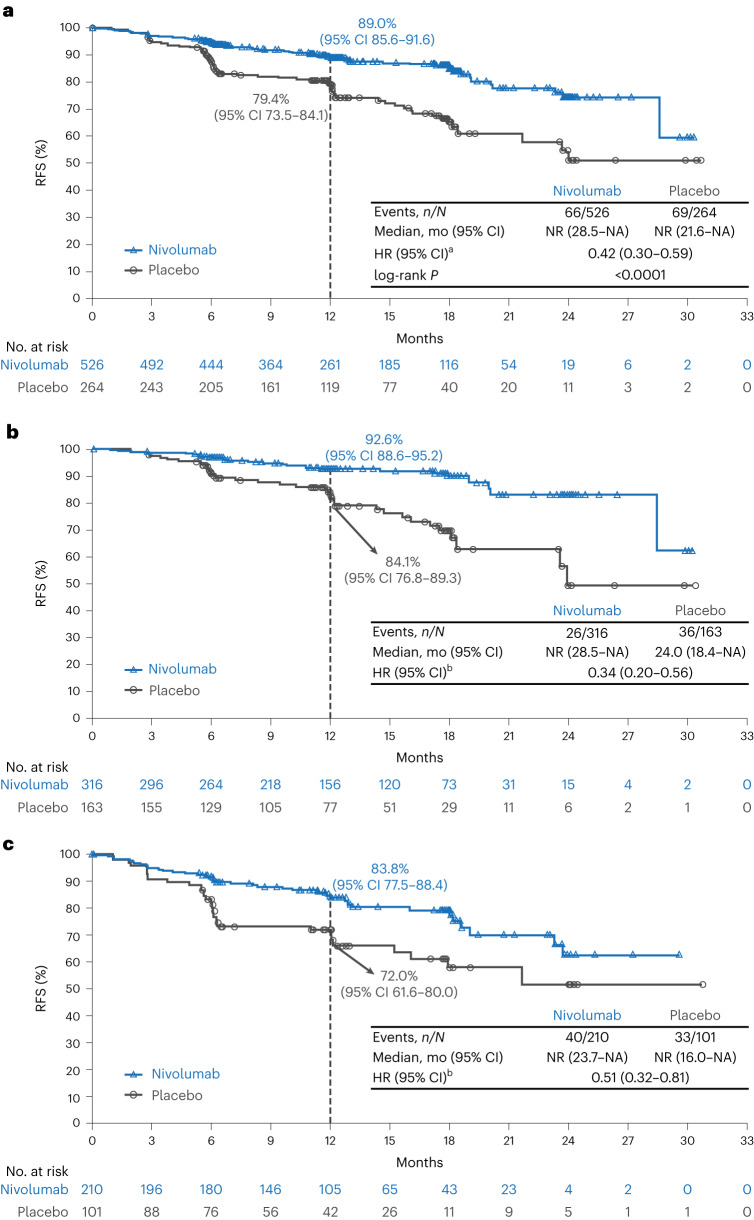
Kaplan–Meier estimates of RFS in the intention-to-treat population (a) and in patients with stage IIB disease (b) and stage IIC disease (c). ^a^Based on Cox proportional hazards model stratified by AJCC 8th edition T category (T3b versus T4a versus T4b) with treatment group as a covariate. The two-sided log-rank *P* value was <0.0001. ^b^Based on unstratified Cox proportional hazards model. mo, months; NA, not available; NR, not reached.

At the time of data cutoff, 12.5% (66/526) of patients in the nivolumab group and 26.1% (69/264) of patients in the placebo group had recurrence or death, with an HR for RFS of 0.42 (95% CI: 0.30–0.59; *P* < 0.0001) and 12-month RFS rates of 89.0% (95% CI: 85.6–91.6) versus 79.4% (95% CI: 73.5–84.1) (Fig. 2a). The improvement in RFS with nivolumab versus placebo was presumably driven by numerically fewer distant recurrences (4.9% (26/526) versus 11.7% (31/264)) and regional recurrences (2.1% (11/526) versus 7.6% (20/264), respectively) (Extended Data Table 1). Occurrence of new primary invasive melanomas at first recurrence was low at 0.8% (4/526) in the nivolumab group and 1.1% (3/264) in the placebo group (for melanoma in situ, it was 1.3% (7/526) and 1.9% (5/264), respectively), and a sensitivity analysis excluding new primary melanoma (invasive and in situ) RFS events was consistent with the primary analysis (HR = 0.39; 95% CI: 0.27–0.57; Supplementary Fig. 1). Sites of lesions/metastases at first recurrence for nivolumab versus placebo were most commonly found in skin (3.6% (19/526) versus 6.1% (16/264)), lungs (3.2% (17/526) versus 9.1% (24/264)) and lymph nodes (3.2% (17/526) versus 12.5% (33/264), respectively) (Extended Data Table 2); six patients in each group had an initial local or regional recurrence or new primary melanoma and went on to have a distant recurrence (Supplementary Fig. 2).

**Fig. 3:**
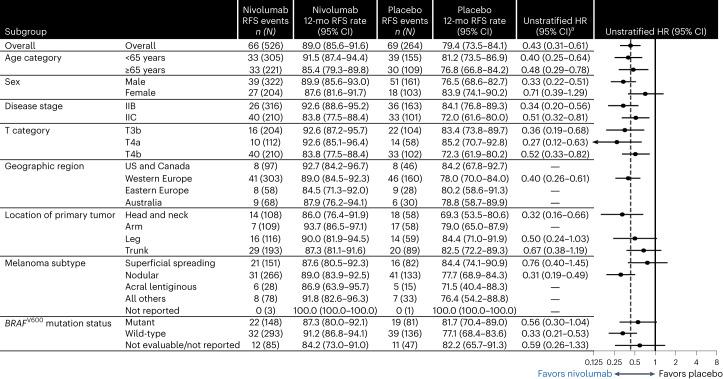
RFS according to subgroup. Shown is a forest plot of unstratified HRs (estimated using an unstratified Cox proportional hazards model) for disease recurrence or death among subgroups of patients. Circles indicate HRs among subgroups of patients, horizontal lines indicate corresponding 95% CIs and the vertical dotted line indicates the HR for the overall population. ^a^Per the statistical analysis plan, HR was not computed for subset categories with fewer than 10 events per treatment group.

**Extended Data Fig. 1:**
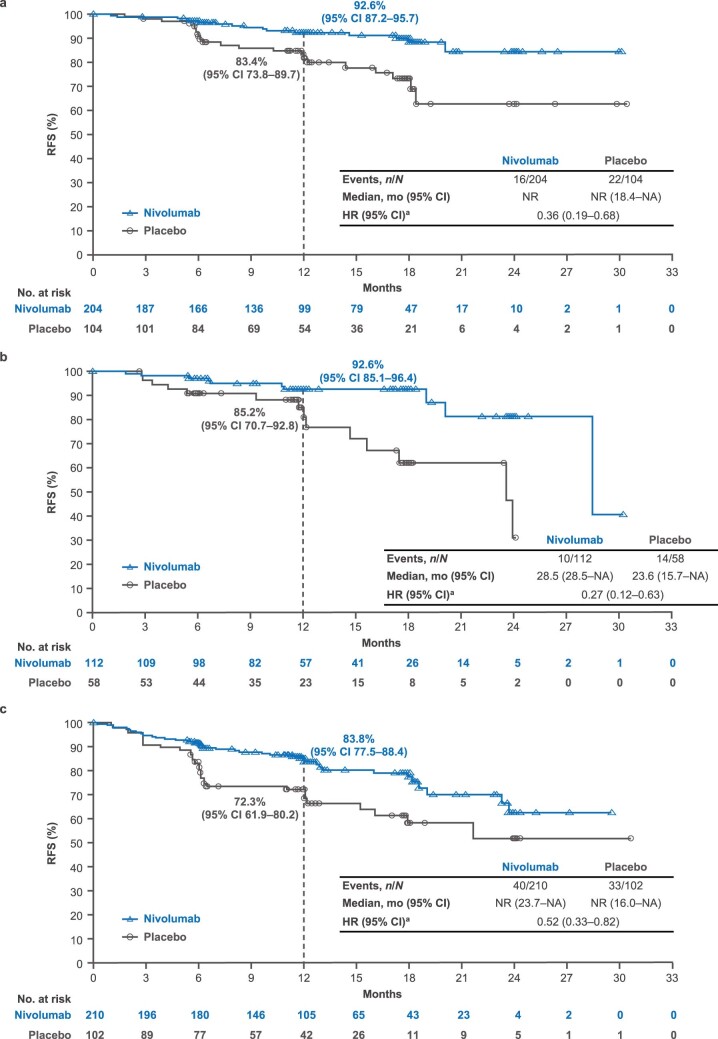
RFS by T category. Shown are Kaplan–Meier estimates of RFS in patients with T3b category disease (**a**), T4a category disease (**b**) and T4b category disease (**c**). ^a^Based on unstratified Cox proportional hazards model. NA, not available; NR, not reached; RFS, recurrence-free survival; T, tumor.

**Extended Data Fig. 2:**
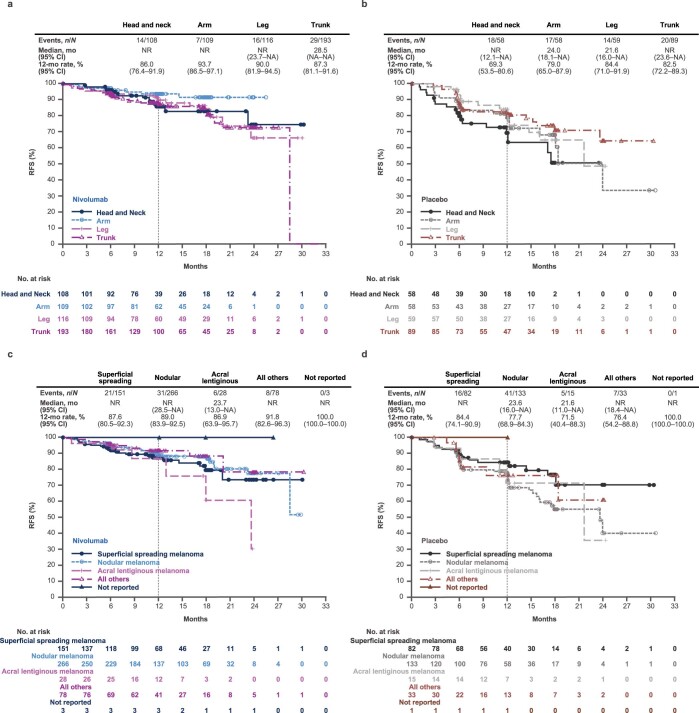
RFS by histology and location of primary tumor. Kaplan–Meier estimates of RFS according to location of primary tumor in patients treated with nivolumab (**a**) and placebo (**b**), as well as according to melanoma subtype in patients treated with nivolumab (**c**) or placebo (**d**). NA, not available; NR, not reached; RFS, recurrence-free survival.

The RFS benefit of nivolumab versus placebo was consistent across stages, with an HR of 0.34 (95% CI: 0.20–0.56) for patients with stage IIB disease and 0.51 (95% CI: 0.32–0.81) for patients with stage IIC disease (Fig. 2b,c). In general, benefit was observed across all pre-specified patient subgroups, with HRs similar to the intention-to-treat HR of 0.43 (95% CI: 0.31–0.61) (Fig. 3). In tumor (T)-category subgroups, 12-month RFS rates (95% CI) for patients in the nivolumab group were 92.6% in T3b (87.2–95.7) and T4a (85.1–96.4) disease and 83.8% (77.5–88.4) in T4b disease; for patients in the placebo group, 12-month RFS rates were 83.4% (73.8–89.7), 85.2% (70.7–92.8) and 72.3% (61.9–80.2), respectively (Fig. 3 and Extended Data Fig. 1). In patients with *BRAF*^V600^ mutations, RFS HR for nivolumab versus placebo was 0.56 (95% CI: 0.30–1.04), with 12-month RFS rates (95% CI) of 87.3% (80.0–92.1) and 81.7% (70.4–89.0), respectively (Fig. 3). In patients with *BRAF*^V600^–wild-type disease, RFS HR for nivolumab versus placebo was 0.33 (95% CI: 0.21–0.53), with 12-month RFS rates (95% CI) of 91.2% (86.8–94.1) and 77.1% (68.4–83.6), respectively. In patients with *BRAF*^V600^ mutation status not evaluable/not reported, RFS HR for nivolumab versus placebo was 0.59 (95% CI: 0.26–1.33), with 12-month RFS rates (95% CI) of 84.2% (73.0–91.0) and 82.2% (65.7–91.3), respectively. Post hoc analyses in patients grouped by primary melanoma site and by primary melanoma histology also showed benefit across these subgroups (Extended Data Fig. 2) and benefit of nivolumab versus placebo (Fig. 3).

**Fig. 4:**
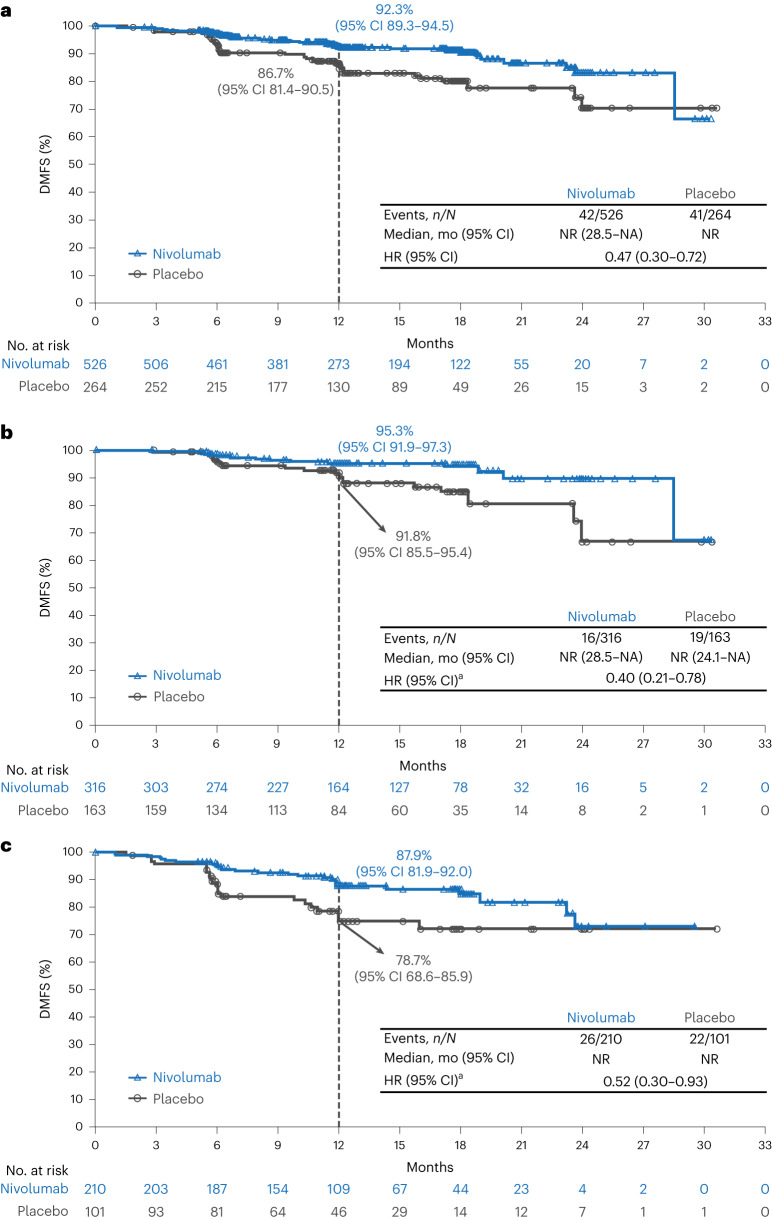
Kaplan–Meier estimates of DMFS in the intention-to-treat population (a) and in patients with stage IIB disease (b) and stage IIC disease (c). ^a^Based on Cox proportional hazards model stratified by AJCC 8th edition T category (T3b versus T4a versus T4b) with treatment group as a covariate. ^b^Based on unstratified Cox proportional hazards model. mo, months; NA, not available; NR, not reached.

Improvement was also observed with adjuvant nivolumab for the key secondary endpoint of distant metastasis-free survival (DMFS), with 8.0% (42/526) of patients in the nivolumab group and 15.5% (41/264) of patients in the placebo group experiencing distant recurrence or death, with an HR of 0.47 (95% CI: 0.30–0.72) for the intention-to-treat population (Fig. 4a). HRs of 0.40 (95% CI: 0.21–0.78) and 0.52 (95% CI: 0.30–0.93) were observed among patients with stage IIB and stage IIC disease, respectively (Fig. 4b,c). Additional planned secondary endpoints not reported in this manuscript were overall survival (OS) and progression-free survival through next-line therapy (known as PFS2).

### Safety

Any-grade treatment-related adverse events ≤30 d after the last dose of study therapy occurred in 82.6% (433/524) of patients with nivolumab and 53.8% (142/264) of patients with placebo, with grade 3 or 4 events in 10.3% (54/524) and 2.3% (6/264) of patients, respectively. Treatment-related adverse events led to treatment discontinuation in 14.7% (77/524) of patients in the nivolumab group and 2.7% (7/264) of patients in the placebo group (Table 2). Overall, there was one treatment-related death in the nivolumab treatment group, due to acute kidney injury and heart failure (not related to myocarditis).

**Table 2:** Summary of adverse events

Adverse event^a^	Nivolumab 480 mg Q4W (*n* = 524)		Placebo Q4W (*n* = 264)	
	Any grade	Grade 3 or 4	Any grade	Grade 3 or 4
**Any adverse event**, ***n*** **(%)**	502 (95.8)^b^	115 (21.9)	229 (86.7)^c^	32 (12.1)
Led to discontinuation of treatment	91 (17.4)	37 (7.1)	9 (3.4)	2 (0.8)
**Treatment-related adverse event**, ***n*** **(%)**	433 (82.6)	54 (10.3)	142 (53.8)	6 (2.3)
Led to discontinuation of treatment	77 (14.7)	29 (5.5)	7 (2.7)	2 (0.8)
**Treatment-related adverse event in ≥5% of patients**^**d**^, ***n*** **(%)**				
Fatigue	106 (20.2)	0	53 (20.1)	1 (0.4)
Pruritus	97 (18.5)	1 (0.2)	25 (9.5)	0
Diarrhea	80 (15.3)	4 (0.8)	25 (9.5)	0
Rash	57 (10.9)	4 (0.8)	18 (6.8)	0
Hypothyroidism	54 (10.3)	0	0	0
Arthralgia	54 (10.3)	1 (0.2)	15 (5.7)	0
Nausea	39 (7.4)	0	7 (2.7)	0
Asthenia	38 (7.3)	0	18 (6.8)	0
Dry mouth	36 (6.9)	0	7 (2.7)	0
Hyperthyroidism	36 (6.9)	1 (0.2)	3 (1.1)	0
Increased alanine aminotransferase	33 (6.3)	4 (0.8)	13 (4.9)	0
Increased aspartate aminotransferase	30 (5.7)	6 (1.1)	6 (2.3)	1 (0.4)
Increased blood creatine phosphokinase	30 (5.7)	6 (1.1)	13 (4.9)	0
Myalgia	28 (5.3)	0	14 (5.3)	0
Infusion-related reaction	27 (5.2)	0	2 (0.8)	0
**Treatment-related death**^**e**^, ***n*** **(%)**	1 (0.2)		0	

^a^The safety population included all patients who received at least one dose of trial drug. The investigators determined whether adverse events were related to a trial drug. The events listed here occurred between the first dose and either 30 d after the last dose (adverse events and treatment-related adverse events) or 100 d after the last dose (treatment-related deaths). The severity of adverse events was graded according to the National Cancer Institute Common Terminology Criteria for Adverse Events, version 5.0.

^b^Including one patient with a grade 5 adverse event of myocardial ischemia.

^c^Including one patient with a grade 5 adverse event of ‘sudden death’.

^d^Occurring in ≥5% of the patients in either treatment group.

^e^There was one treatment-related death due to acute kidney injury and heart failure (not related to myocarditis).

Q4W, every 4 weeks.

The most common any-grade non-endocrine immune-mediated adverse events by category for the nivolumab-treated patients were rash (8.6%, 45/524), diarrhea/colitis (4.6%, 24/524) and hepatitis (4.2%, 22/524); for any-grade endocrine immune-mediated adverse events, it was hypothyroidism/thyroiditis (12.2%, 64/524), hyperthyroidism (7.6%, 40/524) and adrenal insufficiency (2.3%, 12/524) (Supplementary Table 3). The most common non-endocrine immune-mediated adverse events (rash and diarrhea/colitis) had resolved by the time of data cutoff in 60.0% (27/45) and 73.9% (17/23) of patients at a median of 30.6 months and 7.9 months, respectively (Supplementary Table 4). In total, 14.9% (78/524) of patients treated with nivolumab and 1.5% (4/264) of patients treated with placebo received hormonal therapy for an endocrine immune-mediated adverse event (Supplementary Table 5). Grade 3 or 4 adverse event categories for other treatment-related adverse events of special interest in which there were ≥2 patients treated with nivolumab were myocarditis (0.4%, 2/524) and myositis/rhabdomyolysis (1.0%, 5/524) (Supplementary Table 6).

## Discussion

CheckMate 76K met its primary endpoint of RFS. Nivolumab demonstrated a statistically significant and clinically meaningful 58% reduction in the risk of recurrence or death versus placebo in patients with resected stage IIB/C melanoma (HR = 0.42; 95% CI: 0.30–0.59; *P* < 0.0001), with 12-month RFS rates of 89.0% for nivolumab and 79.4% for placebo. The benefit of nivolumab over placebo was observed across all pre-specified subgroups, including all disease stage and T-category subgroups. In addition, a clinically meaningful improvement in DMFS was observed with nivolumab versus placebo (HR = 0.47; 95% CI: 0.30–0.72). The safety profile of nivolumab was consistent with previous reports of adjuvant nivolumab in melanoma, and adverse events were manageable using well-established treatment algorithms.

In this trial, nivolumab reduced the risk of disease recurrence or death by 58% and reduced the risk of distant metastasis by 53% in the intention-to-treat population. The improved outcomes with adjuvant nivolumab in this population follow those reported in KEYNOTE-716, where pembrolizumab reduced the risk of recurrence by 35% (HR = 0.65; 95%: CI: 0.46–0.92) and the risk of distant metastasis by 36% (HR = 0.64; 95% CI: 0.47–0.88)^12,13^. Absolute numbers cannot be compared across trials because of differences in trial and patient characteristics and definitions of study endpoints (in KEYNOTE-716, deaths were not included as events in the DMFS analysis). However, these trials together provide compelling evidence that adjuvant anti-PD-1 immunotherapy can have a significant impact, improving outcomes for patients with resected stage IIB/C melanoma whose risk of recurrence is high. In addition, the fact that the placebo arms for these trials show similar results to those reported for the German Central Malignant Melanoma Registry study allows the interpretation of these results in relation to current real-world outcomes^3^.

Although there is debate about whether new primary melanomas, particularly melanoma in situ, should be considered as recurrence events, the sensitivity analysis excluding these events demonstrated a nearly identical HR (0.39 (95% CI: 0.27–0.57)), indicating that the inclusion of new primary melanomas in the definition of RFS did not impact the magnitude of observed benefit with adjuvant nivolumab. The rate of new primary melanomas was low among patients with a recurrence. However, regular skin surveillance is still needed given the high risk of subsequent primary melanomas in this patient population^14,15^.

RFS benefit from nivolumab was observed across subgroups, including disease stage and T category. Although the initial RFS results from KEYNOTE-716 showed an HR for pembrolizumab versus placebo in patients with T4b melanoma of 0.94 (95% CI: 0.56–1.59)^12^, the HR has improved over time to 0.65 (95% CI: 0.45–0.94), with a DMFS HR of 0.57 (95% CI: 0.36–0.88)^16^. The outcomes of patients with T4b (that is, stage IIC) melanoma in CheckMate 76K (RFS HR = 0.52 (95% CI: 0.33–0.82); DMFS HR = 0.52 (95% CI: 0.30–0.93)) confirm that adjuvant anti-PD-1 immunotherapy shows clinical benefit in patients with resected stage IIB/C disease regardless of T category. A high proportion of patients with nodular melanoma in CheckMate 76K is also notable, where higher recurrence risk is expected. The HR for nivolumab versus placebo among patients with nodular melanoma was 0.31 (95% CI: 0.19–0.49), suggesting a marked benefit with adjuvant nivolumab here. Nivolumab also appeared to be effective regardless of primary tumor location. The placebo group had a lower 12-month RFS rate in patients with head and neck primaries (69.3%) compared with patients with trunk, arm or leg primaries (range, 79.0–84.4%), and the HR for nivolumab versus placebo in this subgroup was 0.32. The subgroup data presented here highlight patients with higher absolute recurrence risk (those with stage IIC disease, head and neck primaries or nodular disease) who would especially benefit from adjuvant treatment. Further analyses combining clinical and translational factors could help to refine benefit–risk discussions and identify which patients with stage IIB/C disease might benefit more or less from adjuvant treatment. Additional analyses evaluating the potential impact of early treatment discontinuation on clinical outcomes may also be warranted, although longer follow-up and a greater number of observed recurrence events would be necessary to draw meaningful conclusions.

Fewer distant as well as local and regional recurrences were observed with nivolumab compared with placebo. Location of first recurrence (locoregional and distant combined) in both groups was predominately skin, lung and lymph node, as expected for melanoma. Fewer patients treated with adjuvant nivolumab experienced metastases at each of these sites versus those treated with placebo. Too few recurrences have occurred thus far at other distant sites to assess trends in the impact of adjuvant nivolumab on reducing metastases to other specific sites. In addition, a lower proportion of patients treated with nivolumab had multiple lesions detected at first recurrence versus those treated with placebo (3.4% versus 9.1%).

The nivolumab safety profile observed here for patients with resected stage IIB/C disease was similar to that observed in patients with resected stage III or stage IV disease, including time to onset and time to resolution of immune-mediated adverse events, and was similar to that in pembrolizumab-treated patients with resected stage IIB/C disease^8,12^. Most adverse events were reversible, excluding endocrine-related adverse events other than hyperthyroidism. Overall, 14.9% of patients in the nivolumab arm required treatment with hormone replacement therapy, primarily driven by thyroid abnormalities, which are fairly common with immunotherapies and are manageable.

The melanoma field has changed considerably from the time when adjuvant therapy was first considered for patients with resected stage IIB/C disease, in the period from 1995 to 2017, when the only available therapy was interferon alpha (IFNα), which was substantially less effective and more toxic than anti-PD-1 immunotherapy^17–19^. With the widespread availability of more effective salvage regimens for regional or distant recurrences, the use of IFNα diminished. The benefit of adjuvant nivolumab treatment for 1 year in CheckMate 76K reinforces the KEYNOTE-716 data and, along with the substantially lower acute toxicity and generally manageable safety profile of anti-PD-1 immunotherapy, supports the view that the large population of node-negative patients with deeper primaries (stage IIB/C) stands to benefit from adjuvant anti-PD-1 immunotherapy. Continued assessment of recurrence patterns, as well as biomarker data, will provide additional insight into the patients who would most benefit from adjuvant treatment; additional biomarker analyses in CheckMate 76K are ongoing. These trials can also inform disease-specific monitoring strategies, as there is currently no consensus on the optimal frequency of imaging assessments in this population, with recommendations from international guidelines varying from every 3 months to every 12 months. Collectively, these data will add insight to outstanding questions regarding adjuvant treatment and surveillance for patients with stage IIB/C disease and can help individual patient benefit–risk discussions. Moreover, one could look further into neoadjuvant treatment as well. Recent findings from the phase 2 SWOG S1801 trial indicated that event-free survival is prolonged in patients with resectable stage IIIB–D with clinically detectable (macroscopic) disease or stage IV melanoma who received neoadjuvant and adjuvant pembrolizumab compared with patients who received only adjuvant pembrolizumab^20^. These results led to the recommendation of perioperative pembrolizumab use in patients with resectable stage IIIB–D disease by the Australian Pharmaceutical Benefits Advisory Committee. However, neoadjuvant/perioperative therapy has primarily been explored in patients with clinically detectable lymph node involvement, and the results cannot be immediately extrapolated to neoadjuvant/perioperative treatment for resectable stage IIB/C melanoma, although this is an area of active exploration.

Although OS benefit with anti-PD-1 immunotherapy has been established in patients with unresectable or metastatic melanoma^21,22^, OS data in the adjuvant setting are limited. At 4.9 years of median follow-up, OS data from the phase 3 EORTC 1325/KEYNOTE-054 trial evaluating adjuvant pembrolizumab versus placebo in patients with resected stage III melanoma were still immature^23^. The phase 3 CheckMate 238 trial did not find a statistically significant difference in OS between adjuvant nivolumab and ipilimumab in patients with resected stage III or stage IV melanoma at a follow-up of 5 years^9^, although an indirect comparison using data from CheckMate 238 and the phase 3 EORTC 18071 trial, adjusted for increases in post-recurrence survival over time with the approval of more effective therapies in the metastatic setting, has suggested that adjuvant nivolumab may prolong OS versus placebo^24^. Although OS data from CheckMate 76K, KEYNOTE-716 and other studies are eagerly awaited, the reduction in the risk of recurrence observed with adjuvant anti-PD-1 immunotherapy, driven primarily by fewer regional and distant recurrences, is compelling. Disease recurrence, particularly regional and distant recurrence, is a clinically important medical event for patients that can lead to physical and psychological morbidity^25^, and, thus, reducing the risk of this event is an important treatment goal. In addition, disease recurrence often portends poor prognosis and an increased risk of mortality, suggesting that it may be useful as a surrogate marker for OS with adjuvant immunotherapy, as already shown in clinical trials evaluating adjuvant IFNα and ipilimumab in patients with resected melanoma^26,27^.

Findings from CheckMate 76K were robust, although potential limitations should be acknowledged. Due to the pre-specified nature of this interim analysis, long-term outcomes (such as OS, as previously discussed) could not be evaluated. In addition, the lack of a blinded independent central review could be viewed as a limitation. However, although efficacy outcomes were assessed by investigators, the inclusion of only patients with completely resected disease, along with the requirement of objective confirmation of recurrence (preferably by biopsy), minimized potential variability in determining whether a recurrence event had occurred. Another trial specification that could be viewed as a limitation is the 2:1 patient randomization because unequal patient allocation may raise concerns of potentially reduced statistical power^28^. However, the 2:1 randomization ratio used in CheckMate 76K was accounted for when performing sample size calculations and determining stopping boundaries for this interim analysis. Lastly, although eligible for enrollment, no patients aged 12–18 years were enrolled, and there was limited racial and ethnic diversity in the enrolled patient population. However, the results of the study are fully generalizable to the population of patients who are most at risk of developing melanoma.

In conclusion, adjuvant nivolumab significantly improved RFS in patients with resected stage IIB/C melanoma by 58% compared with placebo, with clinical benefit observed across disease subgroups, including all T categories. Adjuvant nivolumab also demonstrated substantial improvements in DMFS compared with placebo in this patient population. The observed adverse event profile of nivolumab was similar to the established anti-PD-1 monotherapy profile, which should be taken into account when making treatment decisions with patients. Based on these findings, the European Medicines Agency has approved the use of nivolumab as an adjuvant treatment option for patients ≥12 years of age with completely resected stage IIB/C melanoma. Nivolumab is an effective adjuvant treatment in resected stage IIB/C melanoma.

## Methods

### Patients

Patients ≥12 years of age with an Eastern Cooperative Oncology Group performance status (ECOG PS) of 0 or 1 were eligible if they had histologically confirmed stage IIB or stage IIC cutaneous melanoma (per AJCC Cancer Staging Manual, 8th edition) that was completely resected, with wide excision margins and negative sentinel node biopsy (per local standard), within 12 weeks of randomization. Patients were excluded from the trial if they had any prior treatment for melanoma other than surgery, a history of ocular or mucosal melanoma, other prior malignancy within 3 years of randomization or autoimmune disease or any other condition requiring systemic immunosuppressive medications within 14 d of randomization. Full eligibility criteria are available in the protocol provided in the Supplementary Information.

### Trial design and treatment

**Extended Data Fig. 3:**
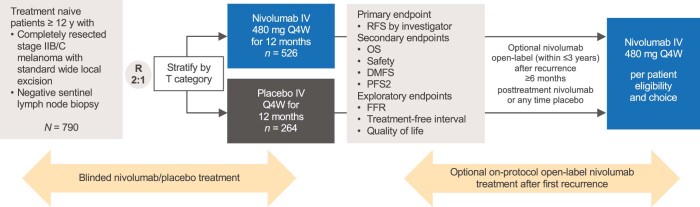
Trial design. RFS was defined as the time between randomization and first recurrence (recurrence events included local, regional, or distant recurrence, new primary melanomas [including in situ], and death [due to any cause]). Imaging assessments occurred every 26 weeks during years 1 through 3 and every 52 weeks in years 4 and 5. DMFS, distant metastasis-free survival (the time between randomization and first distant recurrence or death); FFR, freedom from relapse (with censoring patients who died from causes other than disease); IV, intravenous; OS, overall survival; PFS2, progression-free survival through next-line therapy; Q4W, every 4 weeks; RFS, recurrence-free survival; T, tumor.

In this phase 3, double-blind trial (ClinicalTrials.gov identifier: NCT04099251), eligible patients were randomized 2:1 to receive nivolumab 480 mg or placebo every 4 weeks via intravenous infusion for 12 months or until disease recurrence, unacceptable toxicity or withdrawal of consent (Extended Data Fig. 3). Permuted block randomization, stratified according to AJCC 8th edition T category (T3b versus T4a versus T4b), was performed via interactive web response technology using a randomization schedule. At randomization, patients were assigned to the next available treatment arm in the schedule. Patients, investigators and site staff were blinded to treatment arm assignments during the study, with patients being assigned participant identification numbers to ensure that outcomes could be tracked while concealing treatment allocation. Dose modifications were not permitted, but a dose could be delayed (≤8 weeks in most cases) until the relevant adverse event returned to baseline or decreased in severity to grade ≤1. CheckMate 76K design included optional on-protocol open-label nivolumab treatment after recurrence on either nivolumab (if ≥6 months from last treatment) or placebo (at any time after recurrence) (Extended Data Fig. 3).

### Endpoints and assessments

The primary endpoint for the trial was investigator-assessed RFS, defined as the time between randomization and the first recurrence event. Events included local, regional or distant recurrence; new primary melanomas (including in situ); and death due to any cause. Patients could continue on blinded adjuvant treatment with a diagnosis of melanoma in situ but not with an invasive new primary melanoma. Imaging was required every 26 weeks for the first 3 years and annually in years 4 and 5. Tumor assessments were performed using contrast-enhanced computed tomography of the chest, abdomen, pelvis and all other relevant sites based on known or suspected disease sites (slice thickness ≤5 mm, with no intervening gaps). Cytological and/or histological evidence of recurrence was required in all cases unless a biopsy was deemed to be clinically unsafe or not feasible by the investigator.

Secondary efficacy endpoints included DMFS (presented here), defined as the time between randomization and first distant recurrence or death due to any cause, as well as OS and progression-free survival through next-line therapy (follow-up is ongoing, and those results are not presented here). The secondary endpoint of safety for the blinded phase of the trial is presented here for treatment-related adverse events ≤30 d after the last dose of study therapy as well as for immune-mediated adverse events (non-endocrine events requiring immunomodulators and endocrine events, regardless of immune-modulating treatment) ≤100 d after the last dose of study therapy. Time to onset and resolution data are also presented for immune-mediated adverse events. Occurrences and severity of adverse events were defined by the National Cancer Institute Common Terminology Criteria for Adverse Events, version 5.0 Exploratory health-related quality of life and tissue-based biomarker analyses, including those by PD-L1 expression, are ongoing.

### Trial oversight

The protocol and amendments for this trial were reviewed by the institutional review board or ethics committee for each trial site (Supplementary Table 7). The trial was conducted in accordance with the Declaration of Helsinki and with Good Clinical Practice as defined by the International Conference on Harmonisation. All patients provided written informed consent before enrollment. No patients received monetary compensation. The trial was designed by the lead author and the sponsor, Bristol Myers Squibb. An independent data monitoring committee was established to provide oversight of protocol safety and efficacy. The data were collected by the sponsor and analyzed in collaboration with the authors.

### Statistical analyses

An estimated sample size of 780 patients (520 patients in the nivolumab group and 260 patients in the placebo group) was planned to achieve the required 154 RFS events to detect a statistically significant difference between the treatment arms at final analysis with ≥90% statistical power if the average HR of nivolumab versus placebo was 0.573, with a two-sided alpha of 0.05 by a stratified log-rank test. Per protocol, the current pre-specified interim analysis was scheduled to be conducted when approximately 123 RFS events (80% information fraction) had occurred among all randomized patients; a critical HR ≤0.65 would indicate significant improvement with nivolumab versus placebo with ≥62.8% statistical power. The stopping boundaries for both interim and final analyses were derived using the Lan–DeMets alpha spending function with O’Brien–Fleming boundaries. A total of 790 patients were randomized, and, at the data cutoff date of 28 June 2022, there were 135 reported RFS events (88% information fraction), resulting in a critical HR of 0.678 and 76.8% statistical power to detect a difference of RFS between the arms. DMFS was a descriptive secondary endpoint, and the trial was not powered to compare this endpoint between the two treatment groups.

A two-sided log-rank test stratified by AJCC 8th edition T category was used to compare the treatments for the intention-to-treat populations for both RFS and DMFS. HRs and CIs were estimated using a stratified Cox proportional hazards model with treatment group as a covariate. Comparison for subgroups was estimated using an unstratified Cox proportional hazards model. Survival was estimated using the Kaplan–Meier product-limit method. Medians and rates were estimated, and corresponding 95% CIs were computed based on a log–log-transformed CI for the survivor function.

The efficacy analysis endpoints included all randomized patients (defined as, the intention-to-treat population). Safety was assessed in patients who received at least one dose of study drug.

### Reporting summary

Further information on research design is available in the Nature Portfolio Reporting Summary linked to this article.

## Online content

Any methods, additional references, Nature Portfolio reporting summaries, source data, extended data, supplementary information, acknowledgements, peer review information; details of author contributions and competing interests; and statements of data and code availability are available at 10.1038/s41591-023-02583-2.

### Supplementary information

Supplementary Information Supplementary Tables 1–7, Supplementary Figs. 1 and 2 and Clinical Trial Protocol.

Reporting Summary

## Figures and Tables

**Extended Data Table 1:**
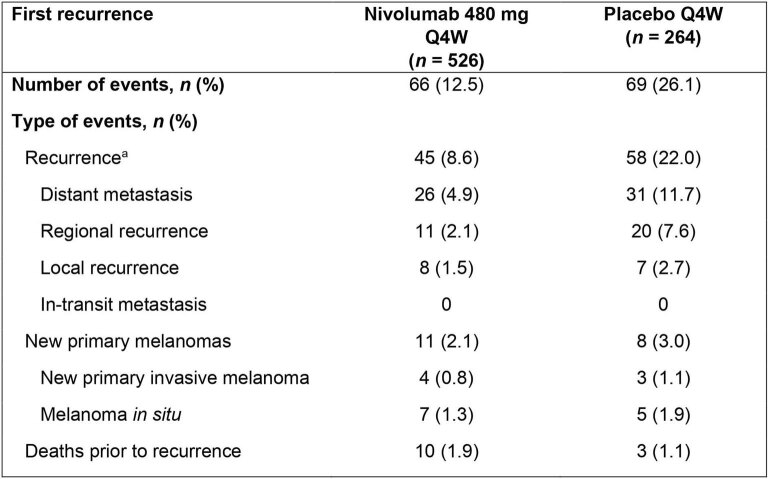
Patterns of first recurrence

^a^For patients who had multiple recurrences that were identified on the same day, the most serious type is tabulated according to the pre-specified hierarchy as listed here.

Q4W, every 4 weeks.

**Extended Data Table 2:**
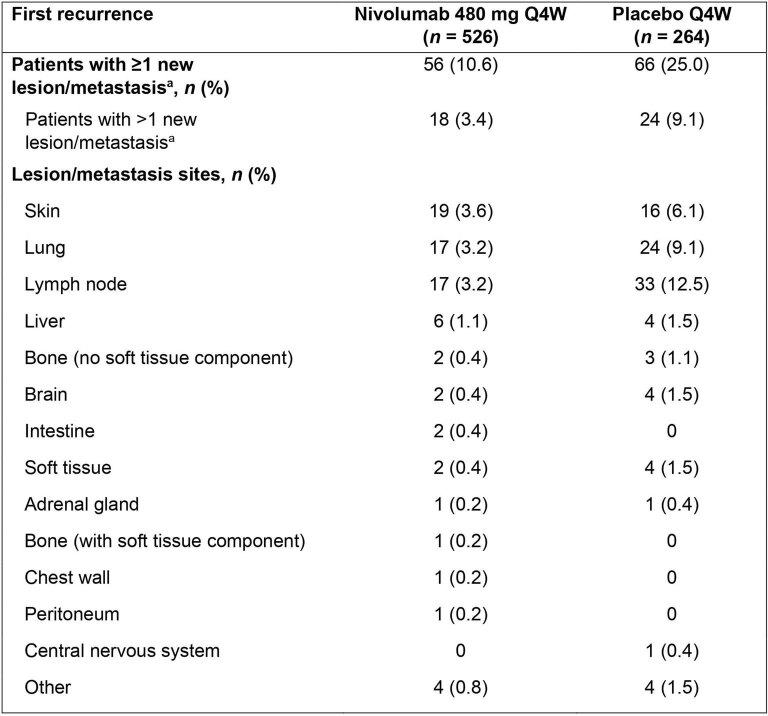
Location of first recurrence

^a^Includes all recurrences: local, regional, distant and new primary (including in situ).

Q4W, every 4 weeks.

## Data Availability

Qualified researchers may submit a proposal to access de-identified and anonymized datasets for this study to Bristol Myers Squibb. Data will be made available to researchers whose proposals are approved by the independent review committee (Duke University), with available information dependent upon the individual request. The option to submit data requests as well as review criteria for data requests are available at https://vivli.org/ourmember/bristol-myers-squibb/. Additional information on Bristol Myers Squibb’s policy on data sharing may be found at https://www.bms.com/researchers-and-partners/clinical-trials-and-research/disclosure-commitment.html. The study protocol of CheckMate 76K is provided in the Supplementary Information.

## Code availability

No custom code was used for statistical analyses in CheckMate 76K. Medidata Classic Rave (version 2022.3.2) was used for data collection throughout this study. All analyses were performed using SAS software (version 9.4).
