# Publisher Correction: Adjuvant nivolumab in resected stage IIB/C melanoma: primary results from the randomized, phase 3 CheckMate 76K trial

**DOI:** 10.1038/s41591-023-02661-5

**Published:** 2023-11-03

**Authors:** John M. Kirkwood, Michele Del Vecchio, Jeffrey Weber, Christoph Hoeller, Jean-Jacques Grob, Peter Mohr, Carmen Loquai, Caroline Dutriaux, Vanna Chiarion-Sileni, Jacek Mackiewicz, Piotr Rutkowski, Petr Arenberger, Gaelle Quereux, Tarek M. Meniawy, Paolo A. Ascierto, Alexander M. Menzies, Piyush Durani, Maurice Lobo, Federico Campigotto, Brian Gastman, Georgina V. Long

**Affiliations:** 1 UPMC Hillman Cancer Center, Pittsburgh, PA USA; 2 Fondazione IRCCS Istituto Nazionale dei Tumori, Milan, Italy; 3 NYU Langone Medical Center, New York, NY USA; 4 Medizinische Universität Wien, Vienna, Austria; 5 Hôpital de la Timone, Marseille, France; 6 Elbe Klinikum Buxtehude, Buxtehude, Germany; 7 University Medical Center Mainz, Mainz, Germany; 8 Hôpital Saint André, Bordeaux, France; 9 Istituto Oncologico Veneto, IOV-IRCCS, Padova, Italy; 10 Institute of Oncology, Poznan University of Medical Sciences, Poznan, Poland; 11 Maria Skłodowska-Curie National Research Institute of Oncology, Warsaw, Poland; 12 Charles University Third Faculty of Medicine and University Hospital Královské Vinohrady, Prague, Czech Republic; 13 Nantes University Hospital, Nantes, France; 14 University of Western Australia and Sir Charles Gairdner Hospital, Perth, WA Australia; 15 Istituto Nazionale Tumori IRCCS ‘Fondazione G. Pascale’, Naples, Italy; 16 Melanoma Institute Australia, University of Sydney, and Royal North Shore and Mater Hospitals, Sydney, NSW Australia; 17 Bristol Myers Squibb, Princeton, NJ USA; 18 Cleveland Clinic, Cleveland, OH USA

**Keywords:** Melanoma, Cancer immunotherapy

Correction to: *Nature Medicine* 10.1038/s41591-023-02583-2, published online 16 October 2023.

In the version of the article initially published, John M. Kirkwood was presented in the author list without a middle initial. The error has been corrected in the HTML and PDF versions of the article.

