# Author Correction: Adjuvant nivolumab in resected stage IIB/C melanoma: primary results from the randomized, phase 3 CheckMate 76K trial

**DOI:** 10.1038/s41591-023-02775-w

**Published:** 2024-01-04

**Authors:** John M. Kirkwood, Michele Del Vecchio, Jeffrey Weber, Christoph Hoeller, Jean-Jacques Grob, Peter Mohr, Carmen Loquai, Caroline Dutriaux, Vanna Chiarion-Sileni, Jacek Mackiewicz, Piotr Rutkowski, Petr Arenberger, Gaelle Quereux, Tarek M. Meniawy, Paolo A. Ascierto, Alexander M. Menzies, Piyush Durani, Maurice Lobo, Federico Campigotto, Brian Gastman, Georgina V. Long

**Affiliations:** 1 UPMC Hillman Cancer Center, Pittsburgh, PA USA; 2 Fondazione IRCCS Istituto Nazionale dei Tumori, Milan, Italy; 3 NYU Langone Medical Center, New York, NY USA; 4 Medizinische Universität Wien, Vienna, Austria; 5 Hôpital de la Timone, Marseille, France; 6 Elbe Klinikum Buxtehude, Buxtehude, Germany; 7 University Medical Center Mainz, Mainz, Germany; 8 Hôpital Saint André, Bordeaux, France; 9 Istituto Oncologico Veneto, IOV-IRCCS, Padova, Italy; 10 Institute of Oncology, Poznan University of Medical Sciences, Poznan, Poland; 11 Maria Skłodowska-Curie National Research Institute of Oncology, Warsaw, Poland; 12 Charles University Third Faculty of Medicine and University Hospital Královské Vinohrady, Prague, Czech Republic; 13 Nantes University Hospital, Nantes, France; 14 University of Western Australia and Sir Charles Gairdner Hospital, Perth, WA Australia; 15 Istituto Nazionale Tumori IRCCS ‘Fondazione G. Pascale’, Naples, Italy; 16 Melanoma Institute Australia, University of Sydney, and Royal North Shore and Mater Hospitals, Sydney, NSW Australia; 17 Bristol Myers Squibb, Princeton, NJ USA; 18 Cleveland Clinic, Cleveland, OH USA

**Keywords:** Melanoma, Cancer immunotherapy

Correction to: *Nature Medicine* 10.1038/s41591-023-02583-2, published online 16 October 2023.

In the version of the article originally published, there was an error in the inset table in Extended Data Fig. 1a, where the text now reading “HR (95% CI)” previously read “Log-rank *P*”. This has now been corrected in the HTML and PDF versions of the article.

