# Supplementary material for: Adjuvant nivolumab in resected stage IIB/C melanoma: primary results from the randomized, phase 3 CheckMate 76K trial
Source: Nat Med. 2023 Oct 16;29(11):2835–43. doi: 10.1038/s41591-023-02583-2 (PMC10667090; doi:10.1038/s41591-023-02583-2)
Supplement: Supplementary file 1 — Supplementary Tables 1–7, Supplementary Figs. 1 and 2 and Clinical Trial Protocol. [file 41591_2023_2583_MOESM1_ESM.pdf]

nature medicine

Article https://doi.org/10.1038/s41591-023-02583-2

# Adjuvant nivolumab in resected stage IIB/C melanoma: primary results from the randomized, phase 3 CheckMate 76K trial

In the format provided by the authors and unedited

**Supplementary appendix**

Adjuvant nivolumab versus placebo in resected stage IIB/C melanoma: results from the randomized, double-blind, phase 3 CheckMate 76K trial

J. M. Kirkwood, M. Del Vecchio, J. Weber, C. Hoeller, J.-J. Grob, P. Mohr, C. Loquai, C. Dutriaux, V. Chiarion-Sileni, J. Mackiewicz, P. Rutkowski, P. Arenberger, G. Quereux, T. Meniawy, P. A. Ascierto, A. Menzies, P. Durani, M. Lobo, F. Campigotto, B. Gastman, G. V. Long


| | |
|---|---|
| Supplementary Table 1 \| Treatment exposure and duration of therapy | Page 2 |
| Supplementary Table 2 \| Subsequent cancer therapy | Page 3 |
| Supplementary Table 3 \| Immune-mediated adverse events | Page 4 |
| Supplementary Table 4 \| Time to onset and resolution of immune-mediated adverse events | Page 6 |
| Supplementary Table 5 \| Endocrine immune-mediated adverse events treated with hormonal therapy | Page 8 |
| Supplementary Table 6 \| Treatment-related adverse events of special interest | Page 9 |
| Supplementary Table 7 \| Independent ethics committees and institutional review boards | Page 10 |
| Supplementary Fig. 1 \| Recurrence-free survival sensitivity analysis excluding patients with new primaries | Page 31 |
| Supplementary Fig. 2 \| Course of disease and therapy of patients with a recurrence event. | Page 32 |
| Clinical Trial Protocol | Page 33 |

**Supplementary Table 1 | Treatment exposure and duration of therapy**

| **Treatment** | **Nivolumab 480 mg Q4W (*n* = 524)** | **Placebo Q4W (*n* = 264)** |
|---|---|---|
| **Median number of doses received (range)** | 12.0 (1–14) | 13.0 (1–14) |
| **Duration of therapy** | | |
| Median (range), months | 11.0 (0.0–12.1) | 11.1 (0.0–12.7) |
| Distribution, *n* (%) | | |
| >3 months | 454 (86.6) | 249 (94.3) |
| >6 months | 406 (77.5) | 233 (88.3) |
| >9 months | 352 (67.2) | 206 (78.0) |
| >12 months | 17 (3.2) | 10 (3.8) |

Q4W, every 4 weeks.

**Supplementary Table 2 | Subsequent cancer therapy[a]**

| Subsequent therapy | Nivolumab 480 mg Q4W (*n* = 526) | Placebo Q4W (*n* = 264) |
|---|---|---|
| **Any therapy, *n* (%)** | 50 (9.5) | 62 (23.5) |
| Surgery[b] | 36 (6.8) | 39 (14.8) |
| Radiotherapy | 10 (1.9) | 3 (1.1) |
| Systemic therapy | 30 (5.7) | 49 (18.6) |
| Anti–PD-(L)1 agent | 15 (2.9) | 44 (16.7) |
| Nivolumab | 13 (2.5) | 43 (16.3) |
| Pembrolizumab | 3 (0.6) | 1 (0.4) |
| Retifanlimab | 1 (0.2) | 0 |
| Ipilimumab | 9 (1.7) | 13 (4.9) |
| Ipilimumab + nivolumab | 5 (1.0) | 3 (1.1) |
| Nivolumab + relatlimab | 1 (0.2) | 2 (0.8) |
| Relatlimab | 0 | 1 (0.4) |
| BRAF inhibitor | 5 (1.0) | 2 (0.8) |
| MEK inhibitor | 6 (1.1) | 3 (1.1) |
| BRAF + MEK inhibitors | 2 (0.4) | 1 (0.4) |
| Other and chemotherapy | 3 (0.6) | 2 (0.8) |
| Investigational | 3 (0.6) | 2 (0.8) |

[a]Patients may have received more than one type of post-protocol therapy and more than one agent within each type. All percentages are based on total number of patients in each group.

[b]Including tumor resection for diagnostic purposes and biopsies. For nivolumab, *n* = 32 (6.1%) were recorded as curative and *n* = 4 (0.8%) were recorded as “other”; for placebo, *n* = 33 (12.5%) were recorded as “curative,” *n* = 3 (1.1%) as “palliative,” and *n* = 3 (1.1%) as “other.”

PD-(L)1, programmed death (ligand) 1; Q4W, every 4 weeks.

**Supplementary Table 3 | Immune-mediated adverse events**

| Adverse event[a] | Nivolumab 480 mg Q4W (*n* = 524) | | Placebo Q4W (*n* = 264) | |
|---|---|---|---|---|
| | **Any grade** | **Grade 3 or 4** | **Any grade** | **Grade 3 or 4** |
| **Non-endocrine immune-mediated adverse event where immune modulating mediation was initiated, *n* (%)** | | | | |
| **Pneumonitis** | 4 (0.8) | 1 (0.2) | 2 (0.8) | 0 |
| Immune-mediated pneumonitis | 4 (0.8) | 1 (0.2) | 2 (0.8) | 0 |
| **Diarrhea/colitis** | 24 (4.6) | 6 (1.1) | 2 (0.8) | 1 (0.4) |
| Diarrhea | 19 (3.6) | 3 (0.6) | 0 | 0 |
| Colitis | 6 (1.1) | 2 (0.4) | 1 (0.4) | 0 |
| Autoimmune colitis | 2 (0.4) | 0 | 1 (0.4) | 1 (0.4) |
| Autoimmune enteropathy | 1 (0.2) | 0 | 0 | 0 |
| Enterocolitis | 1 (0.2) | 1 (0.2) | 0 | 0 |
| **Hepatitis** | 22 (4.2) | 14 (2.7) | 1 (0.4) | 0 |
| Increased ALT | 13 (2.5) | 6 (1.1) | 1 (0.4) | 0 |
| Increased AST | 12 (2.3) | 8 (1.5) | 0 | 0 |
| Hepatitis | 5 (1.0) | 3 (0.6) | 0 | 0 |
| Autoimmune hepatitis | 2 (0.4) | 2 (0.4) | 0 | 0 |
| Hepatotoxicity | 1 (0.2) | 1 (0.2) | 0 | 0 |
| **Nephritis and renal dysfunction** | 3 (0.6) | 2 (0.4) | 1 (0.4) | 0 |
| Acute kidney injury | 2 (0.4) | 2 (0.4) | 0 | 0 |
| Blood creatinine increased | 1 (0.2) | 0 | 0 | 0 |
| Nephritis | 0 | 0 | 1 (0.4) | 0 |
| **Rash** | 45 (8.6) | 4 (0.8) | 4 (1.5) | 0 |
| Rash | 23 (4.4) | 3 (0.6) | 1 (0.4) | 0 |
| Maculopapular rash | 14 (2.7) | 1 (0.2) | 1 (0.4) | 0 |
| Dermatitis | 2 (0.4) | 0 | 1 (0.4) | 0 |
| Macular rash | 2 (0.4) | 0 | 1 (0.4) | 0 |
| Pruritic rash | 2 (0.4) | 0 | 0 | 0 |
| Dermatitis acneiform | 1 (0.2) | 0 | 0 | 0 |

| Adverse event[a] | Nivolumab 480 mg Q4W (*n* = 524) | | Placebo Q4W (*n* = 264) | |
|---|---|---|---|---|
| | Any grade | Grade 3 or 4 | Any grade | Grade 3 or 4 |
| Papular rash | 1 (0.2) | 0 | 0 | 0 |
| Pustular rash | 1 (0.2) | 0 | 0 | 0 |
| **Hypersensitivity** | 7 (1.3) | 0 | 0 | 0 |
| Infusion-related reaction | 7 (1.3) | 0 | 0 | 0 |
| **Endocrine immune-mediated adverse event regardless of immune-modulating medication initiation, *n* (%)** | | | | |
| **Adrenal insufficiency** | 12 (2.3) | 3 (0.6) | 3 (1.1) | 0 |
| Adrenal insufficiency | 12 (2.3) | 3 (0.6) | 3 (1.1) | 0 |
| **Hypothyroidism/thyroiditis[b]** | 64 (12.2) | 0 | 0 | 0 |
| Hypothyroidism | 59 (11.3) | 0 | 0 | 0 |
| Thyroiditis | 4 (0.8) | 0 | 0 | 0 |
| Autoimmune thyroiditis | 2 (0.4) | 0 | 0 | 0 |
| Autoimmune hypothyroidism | 1 (0.2) | 0 | 0 | 0 |
| **Diabetes mellitus** | 3 (0.6) | 3 (0.6) | 0 | 0 |
| Diabetes mellitus | 3 (0.6) | 3 (0.6) | 0 | 0 |
| **Hyperthyroidism** | 40 (7.6) | 1 (0.2) | 4 (1.5) | 0 |
| **Hypophysitis** | 9 (1.7) | 5 (1.0) | 2 (0.8) | 0 |
| Hypophysitis | 5 (1.0) | 3 (0.6) | 2 (0.8) | 0 |
| Hypopituitarism | 2 (0.4) | 1 (0.2) | 0 | 0 |
| Lymphocytic hypophysitis | 2 (0.4) | 1 (0.2) | 0 | 0 |

[a]All immune-mediated adverse events are shown. Listed are events that were reported between the first dose and 100 days after the last dose (both non-endocrine events requiring immune-modulating medication and endocrine events regardless of treatment and not requiring specific laboratory criteria). The severity of adverse events was graded according to the National Cancer Institute Common Terminology Criteria for Adverse Events, version 5.0. [b]Some events may overlap in patients with hyperthyroidism. ALT, alanine aminotransferase; AST, aspartate aminotransferase; Q4W, every 4 weeks.

## Supplementary Table 4 | Time to onset and resolution of immune-mediated adverse events[a]

| AE | Nivolumab 480 mg Q4W (*n* = 524) | | Placebo Q4W (*n* = 264) | |
|---|---|---|---|---|
| | Any grade | Grade 3–5 | Any grade | Grade 3–5 |
| **Non-endocrine immune-mediated AEs** | | | | |
| **Pneumonitis** | | | | |
| Patients with AE, *n*/*N* (%) | 4/524 (0.8) | 1/524 (0.2) | 2/264 (0.8) | 0 |
| Median time to onset, weeks (range) | 26.6 (15.1–31.0) | 31.0 (31.0–31.0) | 54.0 (52.3–55.7) | NA |
| Patients with resolution, *n*/*N* (%) | 3/4 (75.0) | 1/1 (100) | 2/2 (100) | NA |
| Median time to resolution, weeks (range) | 11.2 (7.6–15.1+) | 0.7 (0.7–0.7) | 15.1 (12.6–17.7) | NA |
| **Diarrhea/colitis** | | | | |
| Patients with AE, *n*/*N* (%) | 24/524 (4.6) | 6/524 (1.1) | 2/264 (0.8) | 1/264 (0.4) |
| Median time to onset, weeks (range) | 22.9 (0.3–54.4) | 20.8 (13.1–46.4) | 36.9 (17.0–56.9) | 17.0 (17.0–17.0) |
| Patients with resolution, *n*/*N* (%) | 17/23 (73.9) | 5/7 (71.4) | 2/2 (100.0) | 1/1 (100.0) |
| Median time to resolution, weeks (range) | 7.9 (1.1–40.0+) | 11.0 (1.3–39.3+) | 1.9 (1.7–2.1) | 1.7 (1.7–1.7) |
| **Hepatitis** | | | | |
| Patients with AE, *n*/*N* (%) | 22/524 (4.2) | 14/524 (2.7) | 1/264 (0.4) | 0 |
| Median time to onset, weeks (range) | 13.4 (2.1–49.1) | 6.6 (3.3–49.1) | 15.7 (15.7–15.7) | NA |
| Patients with resolution, *n*/*N* (%) | 19/22 (86.4) | 12/14 (85.7) | 0 | NA |
| Median time to resolution, weeks (range) | 6.5 (0.7–46.4+) | 6.8 (0.7–26.0+) | NA | NA |
| **Nephritis and renal dysfunction** | | | | |
| Patients with AE, *n*/*N* (%) | 3/524 (0.6) | 2/524 (0.4) | 1/264 (0.4) | 0 |
| Median time to onset, weeks (range) | 15.7 (9.9–20.4) | 12.8 (9.9–15.7) | 14.1 (14.1–14.1) | NA |
| Patients with resolution, *n*/*N* (%) | 2/3 (66.7) | 1/2 (50.0) | 1/1 (100.0) | NA |
| Median time to resolution, weeks (range) | 7.7 (0.7–7.9+) | NA (0.7–7.9+) | 36.4 (36.4–36.4) | NA |
| **Rash** | | | | |
| Patients with AE, *n*/*N* (%) | 45/524 (8.6) | 4/524 (0.8) | 4/264 (1.5) | 0 |
| Median time to onset, weeks (range) | 9.0 (0.1–48.1) | 14.7 (1.1–48.1) | 25.1 (14.1–36.3) | NA |
| Patients with resolution, *n*/*N* (%) | 27/45 (60.0) | 2/5 (40.0) | 2/4 (50.0) | NA |
| Median time to resolution, weeks (range) | 30.6 (0.4–86.6+) | 34.1 (7.7+–38.1+) | NA (4.0–57.4+) | NA |
| **Hypersensitivity** | | | | |
| Patients with AE, *n*/*N* (%) | 7/524 (1.3) | 0 | 0 | 0 |
| Median time to onset, weeks (range) | 7.9 (4.0–42.1) | NA | NA | NA |
| Patients with resolution, *n*/*N* (%) | 7/7 (100.0) | NA | NA | NA |
| Median time to resolution, weeks (range) | 0.1 (0.1–0.1) | NA | NA | NA |
| **Endocrine-related AEs** | | | | |
| **Adrenal insufficiency** | | | | |
| Patients with AE, *n*/*N* (%) | 12/524 (2.3) | 3/524 (0.6) | 3/264 (1.1) | 0 |
| Median time to onset, weeks (range) | 32.1 (7.6–66.1) | 28.0 (7.6–66.1) | 32.1 (23.6–44.9) | NA |
| Patients with resolution, *n*/*N* (%) | 3/12 (25.0) | 2/3 (66.7) | 0/3 | NA |
| Median time to resolution, weeks (range) | NA (0.4–61.7+) | 0.9 (0.4–35.9+) | NA | NA |
| **Hypothyroidism/thyroiditis** | | | | |
| Patients with AE, *n*/*N* (%) | 64/524 (12.2) | 0 | 0 | 0 |
| Median time to onset, weeks (range) | 12.4 (2.3–63.7) | NA | NA | NA |
| Patients with resolution, *n*/*N* (%) | 15/64 (23.4) | NA | NA | NA |
| Median time to resolution, weeks (range) | NA (1.4–126.9+) | NA | NA | NA |
| **Hypothyroidism** | | | | |
| Patients with AE, *n*/*N* (%) | 60/524 (11.5) | 0 | 0 | 0 |
| Median time to onset, weeks (range) | 12.2 (2.3–63.7) | NA | NA | NA |
| Patients with resolution, *n*/*N* (%) | 15/60 (25.0) | NA | NA | NA |
| Median time to resolution, weeks (range) | NA (1.4–122.9+) | NA | NA | NA |
| **Thyroiditis** | | | | |
| Patients with AE, *n*/*N* (%) | 6/524 (1.1) | 0 | 0 | 0 |
| Median time to onset, weeks (range) | 14.6 (3.7–20.3) | NA | NA | NA |
| Patients with resolution, *n*/*N* (%) | 2/6 (33.3) | NA | NA | NA |
| Median time to resolution, weeks (range) | NA (1.4–113.6+) | NA | NA | NA |
| **Diabetes mellitus** | | | | |
| Patients with AE, *n*/*N* (%) | 3/524 (0.6) | 3/524 (0.6) | 0 | 0 |

| AE | Nivolumab 480 mg Q4W (*n* = 524) | | Placebo Q4W (*n* = 264) | |
|---|---|---|---|---|
| | **Any grade** | **Grade 3–5** | **Any grade** | **Grade 3–5** |
| Median time to onset, weeks (range) | 28.1 (3.9–38.1) | 28.1 (3.9–38.1) | NA | NA |
| Patients with resolution, *n*/*N* (%) | 0/3 | 0/3 | NA | NA |
| Median time to resolution, weeks (range) | NA | NA | NA | NA |
| **Hyperthyroidism** | | | | |
| Patients with AE, *n*/*N* (%) | 40/524 (7.6) | 1/524 (0.2) | 4/264 (1.5) | 0 |
| Median time to onset, weeks (range) | 7.9 (3.6–40.1) | 20.9 (20.9–20.9) | 40.2 (17.1–55.1) | NA |
| Patients with resolution, *n*/*N* (%) | 37/40 (92.5) | 1/1 (100.0) | 2/4 (50.0) | NA |
| Median time to resolution, weeks (range) | 4.4 (1.6+–95.1+) | 4.3 (4.3–4.3) | 19.4 (4.1–19.4) | NA |
| **Hypophysitis** | | | | |
| Patients with AE, *n*/*N* (%) | 9/524 (1.7) | 5/524 (1.0) | 2/264 (0.8) | 0 |
| Median time to onset, weeks (range) | 31.0 (7.9–42.1) | 28.6 (7.9–40.1) | 29.9 (12.0–47.9) | NA |
| Patients with resolution, *n*/*N* (%) | 3/9 (33.3) | 3/5 (60.0) | 2/2 (100.0) | NA |
| Median time to resolution, weeks (range) | NA (1.0–76.1+) | 22.3 (1.0–58.0+) | 5.1 (2.3–7.9) | NA |

[a]Included are events that were reported between the first dose and 100 days after the last dose. Time-to resolution of AE (any grade) for a specific category is defined as the longest time from onset to complete resolution or improvement to the grade at baseline among all clustered AEs experienced by the subject in this category per adverse event criteria category. Events which worsened into grade 5 events (death) or have a resolution date equal to the date of death are considered unresolved. If a clustered AE is considered as unresolved, the resolution date will be censored to the last known alive date. Improvement to the grade at baseline implies that all different events in the clustered adverse event should at least have improved to the corresponding (i.e., with same preferred term) baseline grade. Patients who experienced an event without worsening from baseline grade were excluded from the time to resolution analysis. Therefore, in some cases, the number of patients with onset may not be the same as the denominator for the percent of patients with resolution. AE, adverse event; NA not available; Q4W, every 4 weeks.

**Supplementary Table 5 | Endocrine immune-mediated adverse events treated with hormonal therapy[a]**

| Adverse event | Nivolumab 480 mg Q4W (*n* = 524) | Placebo Q4W (*n* = 264) |
|---|---|---|
| **Total, *n* (%)** | 78 (14.9) | 4 (1.5) |
| Hypothyroidism | 52 (9.9) | 0 |
| Adrenal insufficiency | 12 (2.3) | 3 (1.1) |
| Hypophysitis | 5 (1.0) | 1 (0.4) |
| Thyroiditis | 2 (0.4) | 0 |
| Diabetes mellitus | 3 (0.6) | 0 |
| Autoimmune thyroiditis | 1 (0.2) | 0 |
| Hypopituitarism | 2 (0.4) | 0 |
| Lymphocytic hypophysitis | 2 (0.4) | 0 |
| Autoimmune hypothyroidism | 1 (0.2) | 0 |

[a]Steroid, thyroxine, or insulin. Endocrine immune-mediated adverse events for blinded phase within a 100-day safety window are displayed. Q4W, every 4 weeks.

**Supplementary Table 6 | Treatment-related adverse events of special interest**

| Adverse event[a] | Nivolumab 480 mg Q4W (*n* = 524) | | Placebo Q4W (*n* = 264) | |
|---|---|---|---|---|
| | **Any grade** | **Grade 3 or 4** | **Any grade** | **Grade 3 or 4** |
| **Pancreatitis, *n* (%)** | 7 (1.3) | 1 (0.2) | 0 | 0 |
| Pancreatitis | 6 (1.1) | 1 (0.2) | 0 | 0 |
| Autoimmune pancreatitis | 1 (0.2) | 0 | 0 | 0 |
| **Uveitis, *n* (%)** | 1 (0.2) | 0 | 0 | 0 |
| **Myocarditis, *n* (%)** | 3 (0.6) | 2 (0.4) | 0 | 0 |
| **Myositis/rhabdomyolysis, *n* (%)** | 8 (1.5) | 5 (1.0) | 2 (0.8) | 1 (0.4) |
| Myositis | 5 (1.0) | 3 (0.6) | 0 | 0 |
| Immune-mediated myositis | 2 (0.4) | 1 (0.2) | 0 | 0 |
| Rhabdomyolysis | 2 (0.4) | 2 (0.4) | 2 (0.8) | 1 (0.4) |
| Autoimmune myositis | 1 (0.2) | 0 | 0 | 0 |

[a]Listed are events that were reported between the first dose and 100 days after the last dose. The severity of adverse events was graded according to the National Cancer Institute Common Terminology Criteria for Adverse Events, version 5.0. Events also investigated with none reported were: myasthenic syndrome, demyelination event, Guillain-Barré Syndrome, encephalitis, graft versus host disease, autoimmune cytopenia, autoimmune eye disorder, and immune-mediated arthritis. Q4W, every 4 weeks.

**Supplementary Table 7 | Independent ethics committees and institutional review boards**

| Site no. | Principal investigator name | Name and address of IRB/IEC and IRB/IEC chairperson or FWA number |
|---|---|---|
| CA209-76K-0001[a] | Jalving, Mathilde, MD, PhD | Universitair Medisch Centrum Groningen<br>Medisch Ethische Toetsinscommissie<br>T.a.v. Mr. J.w.p. De Vroedt Mha Ambtelijk Secretaris<br>Hanzeplein 1<br>Groningen, 9713 GZ<br>Netherlands<br><br>Chairperson: Kamps, WA, MD, PhD |
| CA209-76K-0002[a] | Suijkerbuijk, Karijn P, MD, PhD | Universitair Medisch Centrum Groningen<br>Medisch Ethische Toetsinscommissie<br>T.a.v. Mr. J.w.p. De Vroedt Mha Ambtelijk Secretaris<br>Hanzeplein 1<br>Groningen, 9713 GZ<br>Netherlands<br><br>Chairperson: Kamps, WA, MD, PhD |
| CA209-76K-0003[a] | Elander, Nils Oskar, MD | Etikprovningsmyndigheten<br>Drottninggatan 4, Box 2110<br>Uppsala, 750 02<br>Sweden<br><br>Chairperson: Julius, Hakan, JD |
| CA209-76K-0005[a] | Winge-Main, Anna K, MD | Regional Committee for Ethics of Medical Research<br>Postboks 1130<br>Blindern<br>Oslo, 0318<br>Norway<br><br>Chairperson: Nesheim, Britt-Ingjerd, MD, PhD |
| CA209-76K-0007[a] | Schmidt, Henrik, MD | De Videnskabsetiske Komiteer for Region Midtjylland<br>Skottenborg 26<br>Viborg, 8800<br>Denmark<br><br>Chairperson: Norrelund, Helene |
| CA209-76K-0008[a] | Buyse, Veronique, MD | Cliniques Universitaires Saint-Luc<br>Commission D'ethiquebiomedicale<br>Hospitalo- Facultaire<br>Promenade de l'Alma 51<br>bte B1.43.03<br>Bruxelles, 1200<br>Belgium |

| Site no. | Principal investigator name | Name and address of IRB/IEC and IRB/IEC chairperson or FWA number |
|---|---|---|
| | | Chairperson: Maloteaux, Jean-Marie, MD |
| CA209-76K-0010[a] | Rorive, Andree, MD | Cliniques Universitaires Saint-Luc<br>Commission D'ethiquebiomedicale<br>Hospitalo- Facultaire<br>Promenade de l'Alma 51<br>bte B1.43.03<br>Bruxelles, 1200<br>Belgium<br><br>Chairperson: Maloteaux, Jean-Marie, MD |
| CA209-76K-0011[a] | Vulsteke, Christof A, MD, PhD | Cliniques Universitaires Saint-Luc Commission D'ethiquebiomedicale Hospitalo-Facultaire Promenade de l'Alma 51 bte B1.43.03<br>Bruxelles, 1200<br>Belgium<br><br>Chairperson: Maloteaux, Jean-Marie, MD |
| CA209-76K-0012[a] | Svane, Inge Marie, MD, PhD | De Videnskabsetiske Komiteer for Region Midtjylland<br>Skottenborg 26<br>Viborg, 8800<br>Denmark<br><br>Chairperson: Norrelund, Helene |
| CA209-76K-0013[a] | Hoejberg, Lise, MD, PhD | De Videnskabsetiske Komiteer for Region Midtjylland<br>Skottenborg 26<br>Viborg, 8800<br>Denmark<br><br>Chairperson: Norrelund, Helene |
| CA209-76K-0014[a] | Hernberg, Micaela, MD | Joint Authority Administration<br>Biomedicum Helsinki 2C<br>PL 705<br>Helsinki, 00029<br>Finland<br><br>Chairperson: Marjamaa, Johan |
| CA209-76K-0015[a] | Skytta, Tanja, MD | Joint Authority Administration<br>Biomedicum Helsinki 2C<br>PL 705<br>Helsinki, 00029<br>Finland<br><br>Chairperson: Marjamaa, Johan |
| CA209-76K-0016[a] | Long, Georgina V, MBBS | Melanoma Institute of Australia Governance<br>The Poche Centre |

| Site no. | Principal investigator name | Name and address of IRB/IEC and IRB/IEC chairperson or FWA number |
|---|---|---|
| | | 40 Rocklands Road<br>North Sydney, New South Wales 2060<br>Australia<br><br>St Vincent's Hospital (Sydney) HREC<br>Translational Research Centre<br>97-105 Boundary Street<br>Darlinghurst, New South Wales 2010<br>Australia<br><br>Chairperson: Brien, Jo-Anne, MBBS |
| CA209-76K-0017[a] | Atkinson, Victoria G, MBBS, FRACP | Ramsay Health Care Queensland HREC<br>Greenslopes Private Hospital<br>Newdegate Street<br>Greenslopes, Queensland 4120<br>Australia<br><br>Chairpersons: Lewandowski, Richard, MBBS; Crawford, Darrell, MD (Previous) |
| CA209-76K-0018[a] | Van der Westhuizen, Andre, MBChB, MMed, FRACP | Calvary Mater Newcastle Research Governance Office Cnr Edith & Platt Street<br>Waratah, New South Wales 2298<br>Australia<br><br>St Vincent's Hospital (Sydney) HREC<br>Translational Research Centre<br>97-105 Boundary Street<br>Darlinghurst, New South Wales 2010<br>Australia<br><br>Chairperson: Brien, Jo-Anne, MBBS |
| CA209-76K-0019[a] | Parente, Phillip, MD<br>Mant, Andrew M, MD (Previous) | St Vincent's Hospital (Sydney) HREC<br>Translational Research Centre<br>97-105 Boundary Street<br>Darlinghurst, New South Wales 2010<br>Australia<br><br>Box Hill Hospital<br>16 Arnold Street<br>Level 2<br>Box Hill, Victoria 3128<br>Australia<br><br>Chairperson: Brien, Jo-Anne, MBBS |
| CA209-76K-0020[a] | Schenker, Michael, MD | Comisia Nationala de Bioetica a Medicamentului si a Dispozitivelor Medicale<br>Stefan Cel Mare Sos 19-21<br>Bucuresti, 020125 |

| Site no. | Principal investigator name | Name and address of IRB/IEC and IRB/IEC chairperson or FWA number |
|---|---|---|
| | | Romania<br><br>Chairperson: Antonescu, Dinu, MD |
| CA209-76K-0021[a] | Alexandru, Aurelia, MD | Comisia Nationala de Bioetica a Medicamentului si a Dispozitivelor Medicale<br>Stefan Cel Mare Sos 19-21<br>Bucuresti, 020125<br>Romania<br><br>Chairperson: Antonescu, Dinu, MD |
| CA209-76K-0022[a] | Rutkowski, Piotr, MD, PhD | Komisja Bioetyczna Przy Narodowym Instytucie Onkologii im. M. Sklodowskiej-Curie Panstwowy Instytut Badawczy w Warszawie<br>Ul. Roentgena 5<br>Warsaw, 02-781<br>Poland<br><br>Chairperson: Meder, Janusz, MD, PhD |
| CA209-76K-0023[a] | Jassem, Jacek, MD | Komisja Bioetyczna przy Narodowym Instytucie Onkologii im. M. Sklodowskiej- Curie Panstwowy Instytut Badawczy w Warszawie<br>Ul. Roentgena 5<br>Warszawa, 02-781<br>Poland<br><br>Chairperson: Meder, Janusz, MD, PhD |
| CA209-76K-0024[a] | Eastgate, Melissa, MBBS, FRACP | St Vincent's Hospital (Sydney) HREC<br>Translational Research Centre<br>97-105 Boundary Street<br>Darlinghurst, New South Wales 2010<br>Australia<br><br>Royal Brisbane and Women's Hospital HREC Office<br>Butterfield Street<br>Herston, Queensland 4031<br>Australia<br><br>St Vincent’s Sydney HREC Research Office<br>Level 6 Delacy Building<br>Darlinghurst, New South Wales 2010 Australia<br><br>Chairperson: Brien, Jo-Anne, MBBS |
| CA209-76K-0025[a] | Carlino, Matteo, MBBS, FRACP | St Vincent's Hospital (Sydney) HREC<br>Translational Research Centre<br>97-105 Boundary Street<br>Darlinghurst, New South Wales 2010 |

| Site no. | Principal investigator name | Name and address of IRB/IEC and IRB/IEC chairperson or FWA number |
|---|---|---|
| | | Australia<br><br>Western Sydney Local Health District HREC<br>Research Office<br>Rm 1072, Lvl 1, Education Blk Westmead<br>Hospital Hawkesbury and Darcy Roads<br>Westmead, New South Wales 2145<br>Australia<br><br>Chairpersons: Carland, Jane, MD<br>Brien, Jo-Anne, MBBS (Previous) |
| CA209-76K-0026[a] | Valachis, Antonios, MD | Etikprovningsmyndigheten<br>Drottninggatan 4, Box 2110<br>Uppsala, 750 02<br>Sweden<br><br>Chairperson: Julius, Hakan, JD |
| CA209-76K-0027[a] | Garabet, Louay, MD | Regional Committee for Ethics of Medical Research<br>Postboks 1130<br>Blindern<br>Oslo, 0318<br>Norway<br><br>Chairperson: Nesheim, Britt-Ingjerd, MD, PhD |
| CA209-76K-0028[a] | Lonchay, Christophe, MD | Cliniques Universitaires Saint-Luc<br>Commission D'ethiquebiomedicale<br>Hospitalo-Facultaire<br>Promenade de l’Alma 51<br>bte B1.43.03<br>Bruxelles, 1200<br>Belgium<br><br>Chairperson: Maloteaux, Jean-Marie, MD |
| CA209-76K-0030[a] | Veldt, Astrid Van der, MD, PhD | Universitair Medisch Centrum Groningen<br>Medisch Ethische Toetsinscommissie<br>T.a.v. Mr. J.w.p. De Vroedt Mha Ambtelijk Secretaris<br>Hanzeplein 1<br>Groningen, 9713 GZ<br>Netherlands<br><br>Chairperson: Kamps, WA, MD, PhD |
| CA209-76K-0031 | Kirkwood, John Munn, MD | Western IRB<br>1019 39th Avenue SE<br>Suite 120<br>Puyallup, WA 98374<br>United States |

| Site no. | Principal investigator name | Name and address of IRB/IEC and IRB/IEC chairperson or FWA number |
| --- | --- | --- |
| | | FWA Number: FWA00000533 |
| CA209-76K-0032[a] | Dalle, Stephane, MD | C.P.P. Sud-Est Iv<br>Centre Leon Berard<br>28 rue Laennec<br>Lyon Cedex 08, 69373<br>France<br><br>Chairperson: Bertrand Reynaud, Amandine, MD |
| CA209-76K-0033[a] | Gaudy, Caroline, MD<br>Grob, Jean-Jacques, MD (Previous) | C.P.P. Sud-Est Iv<br>Centre Leon Berard<br>28 rue Laennec<br>Lyon Cedex 08, 69373<br>France<br><br>Chairperson: Bertrand Reynaud, Amandine, MD |
| CA209-76K-0034[a] | Robert, Caroline, MD | C.P.P. Sud-Est Iv<br>Centre Leon Berard<br>28 rue Laennec<br>Lyon Cedex 08, 69373<br>France<br><br>Chairperson: Bertrand Reynaud, Amandine, MD |
| CA209-76K-0035[a] | Quereux, Gaelle, MD<br>Dreno, Brigitte, MD (Previous) | C.P.P. Sud-Est Iv<br>Centre Leon Berard<br>28 rue Laennec<br>Lyon Cedex 08, 69373<br>France<br><br>Chairperson: Bertrand Reynaud, Amandine, MD |
| CA209-76K-0036[a] | Lebbe, Celeste, MD | C.P.P. Sud-Est Iv<br>Centre Leon Berard<br>28 rue Laennec<br>Lyon Cedex 08, 69373<br>France<br><br>Chairperson: Bertrand Reynaud, Amandine, MD |
| CA209-76K-0037[a] | Del Vecchio, Michele, MD | Comitato Etico INT Milano Istituto Nazionale Dei Tumori Via Venezian, 1<br>Milano, 20133<br>Italy<br><br>Chairperson: Labianca, Roberto, MD |
| CA209-76K-0038[a] | Maio, Michele, MD | Comitato Etico Toscana Area Vasta Sud Est<br>Viale Bracci 16<br>Siena, 53100<br>Italy |

| Site no. | Principal investigator name | Name and address of IRB/IEC and IRB/IEC chairperson or FWA number |
|---|---|---|
| | | Chairperson: Gonnelli, Stefano, MD |
| CA209-76K-0039[a] | Chiarion-Sileni, Vanna, MD | Comitato Etico IOV<br>Irccs Istituto Oncologico Veneto Via Gattamelata, 64<br>Padova, 35128<br>Italy<br><br>Chairperson: Pegoraro, Renzo, MD |
| CA209-76K-0040[a] | Merelli, Barbara, MD<br>Mandala, Mario, MD (Previous) | Comitato Etico Provincia di Bergamo ASST Papa Giovanni XXIII<br>Piazza OMS 1<br>Bergamo, 24127<br>Italy<br><br>Chairperson: Spagnolo, Antonio, MD |
| CA209-76K-0044[a] | Kumar, Satish, MD | South West-Central Bristol Research Ethics Committee<br>Bristol HRA Centre<br>Whitefriars<br>Level 3, Block B<br>Lewins Mead<br>Bristol, BS1 2NT<br>United Kingdom<br><br>Chairperson: Woodley, Julie |
| CA209-76K-0046[a] | Rinaldi, Gaetana, MD | Comitato Etico Palermo 1<br>Via Del Vespro 127<br>Palermo, 90127<br>Italy<br><br>Chairperson: Leone, Salvino |
| CA209-76K-0047[a] | Ciuleanu, Tudor Eliade, MD | Comisia Nationala de Bioetica a Medicamentului si a Dispozitivelor Medicale<br>Stefan Cel Mare Sos 19-21<br>Bucuresti, 020125<br>Romania<br><br>Chairperson: Antonescu, Dinu, MD |
| CA209-76K-0048[a] | Hoeller, Christoph, MD | Ethikkommission Der Med. Fakultaet Wien<br>Borschekegasse 8b/6<br>Wien, 1090<br>Austria<br><br>Chairperson: Zezula, Juergen, MD |
| CA209-76K-0049[a] | Wolf, Ingrid, MD<br>Richtig, Erika, MD (Previous) | Ethikkommission Der Med. Fakultaet Wien<br>Borschekegasse 8b/6<br>Wien, 1090 |

| Site no. | Principal investigator name | Name and address of IRB/IEC and IRB/IEC chairperson or FWA number |
|---|---|---|
| | | Austria<br><br>Chairperson: Zezula, Juergen, MD |
| CA209-76K-0050[a] | Koelblinger, Peter, MD | Ethikkommission Der Med. Fakultaet Wien<br>Borschekegasse 8b/6<br>Wien, 1090<br>Austria<br><br>Chairperson: Zezula, Juergen, MD |
| CA209-76K-0051[a] | Nguyen, Van Anh, MD | Ethikkommission Der Med. Fakultaet Wien<br>Borschekegasse 8b/6<br>Wien, 1090<br>Austria<br><br>Chairperson: Zezula, Juergen, MD |
| CA209-76K-0052[a] | Dummer, Reinhard, MD | Commission cantonal d´ethique<br>Avenue de Chailly 23<br>Lausanne, 1012<br>Switzerland<br><br>Chairperson: Sprumont, Dominique |
| CA209-76K-0053[a] | Michielin, Olivier, MD | Commission cantonal d´ethique<br>Avenue de Chailly 23<br>Lausanne, 1012<br>Switzerland<br><br>Chairperson: Sprumont, Dominique |
| CA209-76K-0054[a] | Schadendorf, Dirk, MD[b] | Ethikkommission Der Med. Fakultaet Alte<br>Glockengiesserei 11/1<br>Heidelberg, 69115<br>Germany<br><br>Chairperson: Strowitzki, Thomas, MD, PhD |
| CA209-76K-0055[a] | Hassel, Jessica Cecile, MD | Ethikkommission Der Med. Fakultaet Alte<br>Glockengiesserei 11/1<br>Heidelberg, 69115<br>Germany<br><br>Chairperson: Strowitzki, Thomas, MD, PhD |
| CA209-76K-0056[a] | Heinzerling, Lucie M, MPH<br>Schlaak, Max, MD (Previous) | Ethikkommission Der Med. Fakultaet Alte<br>Glockengiesserei 11/1<br>Heidelberg, 69115<br>Germany<br><br>Chairperson: Strowitzki, Thomas, MD, PhD |
| CA209-76K-0057[a] | Terheyden, Patrick, MD | Ethikkommission Der Med. Fakultaet<br>Alte Glockengiesserei 11/1<br>Heidelberg, 69115 |

| Site no. | Principal investigator name | Name and address of IRB/IEC and IRB/IEC chairperson or FWA number |
|---|---|---|
| | | Germany<br><br>Chairperson: Strowitzki, Thomas, MD, PhD |
| CA209-76K-0058[a] | Thomas, Ioannis, MD<br>Eigentler, Thomas Kurt, MD (Previous) | Ethikkommission Der Med. Fakultaet Alte Glockengiesserei 11/1<br>Heidelberg, 69115<br>Germany<br><br>Chairperson: Strowitzki, Thomas, MD, PhD |
| CA209-76K-0060[a] | Grimmelmann, Imke, MD<br>Gutzmer, Ralf, MD (Previous) | Ethikkommission Der Med. Fakultaet Alte Glockengiesserei 11/1<br>Heidelberg, 69115<br>Germany<br><br>Chairperson: Strowitzki, Thomas, MD, PhD |
| CA209-76K-0061[a] | Mohr, Peter, MD | Ethikkommission Der Med. Fakultaet Alte Glockengiesserei 11/1<br>Heidelberg, 69115<br>Germany<br><br>Chairperson: Strowitzki, Thomas, MD, PhD |
| CA209-76K-0062[a] | Kaatz, Martin, MD | Ethikkommission Der Med. Fakultaet Alte Glockengiesserei 11/1<br>Heidelberg, 69115<br>Germany<br><br>Chairperson: Strowitzki, Thomas, MD, PhD |
| CA209-76K-0063[a] | Straume, Oddbjorn, MD | Regional Committee for Ethics of Medical Research<br>Postboks 1130<br>Blindern<br>Oslo, 0318<br>Norway<br><br>Chairperson: Nesheim, Britt-Ingjerd, MD, PhD |
| CA209-76K-0064[a] | Garcia, Almudena, MD[b] | CEIm Hospital Univ. La Paz<br>Paseo De La Castellana 261<br>Madrid, 28046<br>Spain<br><br>Chairperson: Castro, Almudena, MD |
| CA209-76K-0065[a] | Manzano Mozo, Jose Luis, MD | CEIm Hospital Univ. La Paz<br>Paseo De La Castellana 261<br>Madrid, 28046<br>Spain<br><br>Chairperson: Castro, Almudena, MD |

| Site no. | Principal investigator name | Name and address of IRB/IEC and IRB/IEC chairperson or FWA number |
|---|---|---|
| CA209-76K-0066[a] | Soria Rivas, Ainara, MD | CEIm Hospital Univ. La Paz<br>Paseo De La Castellana 261<br>Madrid, 28046<br>Spain<br><br>Chairperson: Castro, Almudena, MD |
| CA209-76K-0067[a] | Quindos Varela, Maria, MD | CEIm Hospital Univ. La Paz<br>Paseo De La Castellana 261<br>Madrid, 28046<br>Spain<br><br>Chairperson: Castro, Almudena, MD |
| CA209-76K-0068[a] | Berciano Guerrero, Miguel Angel, MD | CEIm Hospital Univ. La Paz<br>Paseo De La Castellana 261<br>Madrid, 28046<br>Spain<br><br>Chairperson: Castro, Almudena, MD |
| CA209-76K-0069[a] | Juan Fita, Maria Jose, MD<br>Soriano Teruel, Virtudes, MD (Previous) | CEIm Hospital Univ. La Paz<br>Paseo De La Castellana 261<br>Madrid, 28046<br>Spain<br><br>Chairperson: Castro, Almudena, MD |
| CA209-76K-0070[a] | Espinosa Arranz, Enrique, MD | CEIm Hospital Univ. La Paz<br>Paseo De La Castellana 261<br>Madrid, 28046<br>Spain<br><br>Chairperson: Castro, Almudena, MD |
| CA209-76K-0071[a] | Gonzalez-Cao, Maria, MD | CEIm Hospital Univ. La Paz<br>Paseo De La Castellana 261<br>Madrid, 28046<br>Spain<br><br>Chairperson: Castro, Almudena, MD |
| CA209-76K-0072[a] | Landsberg, Jennifer, MD<br>Sirokay, Judith, MD (Previous) | Ethikkommission Der Med. Fakultaet Alte Glockengiesserei 11/1 Heidelberg, 69115<br>Germany<br><br>Chairperson: Strowitzki, Thomas, MD, PhD |
| CA209-76K-0073[a] | Arenberger, Petr, MD | Eticka Komise Fakultni Nemocnice<br>Kralovske Vinohrady<br>Srobarova 1150/50<br>Praha 10, 100 34<br>Czech Republic<br><br>Chairperson: Pachl, Jan, MD |

| Site no. | Principal investigator name | Name and address of IRB/IEC and IRB/IEC chairperson or FWA number |
|---|---|---|
| CA209-76K-0074[a] | Krajsova, Ivana, MD, MBA | Eticka Komise Fakultni Nemocnice Kralovske Vinohrady<br>Srobarova 1150/50<br>Praha 10, 100 34<br>Czech Republic<br><br>Eticka komise Vseobecne fakultni nemocnice v Praze<br>Na Bojisti 1<br>Praha 2, 128 08<br>Czech Republic<br><br>Chairpersons: Pachl, Jan, MD<br>Sklenar, Zbynek, MD<br>Sedivy, Josef, MD (Previous) |
| CA209-76K-0075[a] | Vantuchova, Yvetta, MD | Eticka Komise Fakultni Nemocnice Kralovske Vinohrady<br>Srobarova 1150/50<br>Praha 10, 100 34<br>Czech Republic<br><br>Eticka komise Fakultni nemocnice Ostrava<br>17. listopadu 1790<br>Ostrava – Poruba, 708 52<br>Czech Republic<br><br>Chairpersons: Pachl, Jan, MD<br>Zelenik, Karol, MD, PhD |
| CA209-76K-0076 | Curti, Brendan D, MD | Providence St. Joseph Health IRB<br>1801 Lind Avenue SW<br>Renton, WA 98057<br>United States<br><br>Providence Health & Services<br>5251 NE Glisan Street Building A – 3rd Floor<br>Portland, OR 97213<br>United States (Previous)<br><br>FWA Number: FWA00001033 |
| CA209-76K-0077 | Kim, Kevin B, MD | Western IRB<br>1019 39th Avenue SE<br>Suite 120<br>Puyallup, WA 98374<br>United States<br><br>FWA Number: FWA00000533 |
| CA209-76K-0078 | Ott, Patrick, MD, PhD | Dana Farber Cancer Institute IRB<br>450 Brookline Avenue, OS229 |

| Site no. | Principal investigator name | Name and address of IRB/IEC and IRB/IEC chairperson or FWA number |
|---|---|---|
| | | Boston, MA 02215<br>United States<br><br>FWA Number: FWA00001121 |
| CA209-76K-0079 | Hauke, Ralph J, MD[b] | Western IRB<br>1019 39th Avenue SE<br>Suite 120<br>Puyallup, WA 98374<br>United States<br><br>FWA Number: FWA00000533 |
| CA209-76K-0080 | Faries, Mark B, MD | Western IRB<br>1019 39th Avenue SE<br>Suite 120<br>Puyallup, WA 98374<br>United States<br><br>FWA Number: FWA00000533 |
| CA209-76K-0081 | Amatruda, Thomas T, MD | Western IRB<br>1019 39th Avenue SE<br>Suite 120<br>Puyallup, WA 98374<br>United States<br><br>FWA Number: FWA00000533 |
| CA209-76K-0082[a] | Gogas, Helen, MD | National Ethics Committee<br>284 Mesogeion Avenue<br>Cholargos, 15562<br>Greece<br><br>Chairperson: Kolettis, Theofilos, MD, PhD |
| CA209-76K-0083[a] | Baka, Sofia, MD | National Ethics Committee<br>284 Mesogeion Avenue<br>Cholargos, 15562<br>Greece<br><br>Chairperson: Kolettis, Theofilos, MD, PhD |
| CA209-76K-0084[a] | Bafaloukos, Dimitrios, MD | National Ethics Committee<br>284 Mesogeion Avenue<br>Cholargos, 15562<br>Greece<br><br>Chairperson: Kolettis, Theofilos, MD, PhD |
| CA209-76K-0085 | Cowey, Charles Lance, MD | Copernicus Group IRB<br>5000 Centregreen Way<br>Suite 200<br>Cary, NC 27513<br>United States |

| Site no. | Principal investigator name | Name and address of IRB/IEC and IRB/IEC chairperson or FWA number |
|---|---|---|
| | | FWA Number: FWA00000533 |
| CA209-76K-0086 | Atlas, Jennifer Lynn, MD | Advarra IRB<br>6940 Columbia Gateway Drive<br>Suite 110<br>Columbia, MD 21046<br>United States<br><br>FWA Number: FWA00000971 |
| CA209-76K-0087 | Beck, J Thaddeus, MD | Western IRB<br>1019 39th Avenue SE<br>Suite 120<br>Puyallup, WA 98374<br>United States<br><br>FWA Number: FWA00000533 |
| CA209-76K-0088 | Dubay, John William, MD<br>Conry, Robert, MD (Previous)<br>Holder, Ashley Margaret, MD (Previous) | Western IRB<br>1019 39th Avenue SE<br>Suite 120<br>Puyallup, WA 98374<br>United States<br><br>FWA Number: FWA00000533 |
| CA209-76K-0089 | Gibney, Geoffrey Thomas, MD | MedStar Health Research Institute<br>Georgetown University Oncology IRB<br>SW 104 Medical Dental Building<br>3900 Reservoir Road, NW<br>Washington, DC 20057<br>United States<br><br>FWA Number: FWA00000504 |
| CA209-76K-0090 | Jang, Sekwon P, MD | Western IRB<br>1019 39th Avenue SE<br>Suite 120<br>Puyallup, WA 98374<br>United States<br><br>FWA Number: FWA00000533 |
| CA209-76K-0091 | Medina, Theresa Michelle, MD | Western IRB<br>1019 39th Avenue SE<br>Suite 120<br>Pullyap, WA 98374<br>United States<br><br>FWA Number: FWA00000533 |
| CA209-76K-0092 | Nair, Suresh G, MD | Lehigh Valley Health Network IRB<br>Research Participant Protection Office<br>1255 S Cedar Crest Boulevard, Ste 3200 |

| Site no. | Principal investigator name | Name and address of IRB/IEC and IRB/IEC chairperson or FWA number |
|---|---|---|
| | | Allentown, PA 18103<br>United States<br><br>FWA Number: FWA00000624 |
| CA209-76K-0093 | Pecora, Andrew Louis, MD | Western IRB<br>1019 39th Avenue SE<br>Suite 120<br>Pullyap, WA 98374<br>United States<br><br>FWA Number: FWA00000533 |
| CA209-76K-0094 | Weber, Jeffrey S, MD | NYU School of Medicine IRB<br>1 Park Avenue, 6th Floor<br>New York, NY 10016<br>United States<br><br>FWA Number: FWA00004952 |
| CA209-76K-0095[a] | Wheater, Matthew J, MD | South West-Central Bristol Research Ethics Committee<br>Bristol HRA Centre<br>Whitefriars<br>Level 3, Block B Lewins Mead<br>Bristol, BS1 2NT<br>United Kingdom<br><br>Chairperson: Woodley, Julie, MD |
| CA209-76K-0098[a] | Meier, Friedegund, MD | Ethikkommission Der Med. Fakultaet Alte Glockengiesserei 11/1<br>Heidelberg, 69115<br>Germany<br><br>Chairperson: Strowitzki, Thomas, MD, PhD |
| CA209-76K-0099 | Gastman, Brian R, MD | Cleveland Clinic IRB<br>9500 Euclid Avenue<br>Cleveland, OH 44195<br>United States<br><br>FWA Number: FWA00000536 |
| CA209-76K-0100[a] | Grabbe, Stephan, MD<br>Loquai, Carmen, MD (Previous) | Ethikkommission Der Med. Fakultaet Alte Glockengiesserei 11/1<br>Heidelberg, 69115<br>Germany<br><br>Chairperson: Strowitzki, Thomas, MD, PhD |
| CA209-76K-0101[a] | Ascierto, Paolo A, MD | Comitato Etico Istituto Nazionale Tumori Fondazione Pascale<br>Comitato Etico Indipendente Via M. M Semmola<br>Napoli, 80131 |

| Site no. | Principal investigator name | Name and address of IRB/IEC and IRB/IEC chairperson or FWA number |
|---|---|---|
| | | Italy<br><br>Chairperson: Casavola, Francesco Paolo, MD |
| CA209-76K-0102[a] | Haferkamp, Sebastian, MD | Ethikkommission Der Med. Fakultaet Alte Glockengiesserei 11/1<br>Heidelberg, 69115<br>Germany<br><br>Chairperson: Strowitzki, Thomas, MD, PhD |
| CA209-76K-0103[a] | Mackiewicz, Jacek, MD, PhD | Komisja Bioetyczna Przy Narodowym Instytucie Onkologii im. M. Sklodowskiej-Curie Panstwowy Instytut Badawczy w<br>Warszawie<br>Ul. Roentgena 5<br>Warsaw, 02-781<br>Poland<br><br>Chairperson: Meder, Janusz, MD, PhD |
| CA209-76K-0104[a] | Meniawy, Tarek M, MBBS | Sir Charles Gairdner Hospital Herc Corp. Medical Services<br>Sir Charles Gairdner Hospital<br>Nedlands, Western Australia 6009<br>Australia<br><br>Sir Charles Gairdner Hospital Research Governance<br>2nd Floor, A Block<br>Sir Charles Gairdner Hospital<br>Hospital Avenue<br>Nedlands, Western Australia 6009<br>Australia<br><br>Chairperson: Flack, Felicity, MD |
| CA209-76K-0105[a] | Lyle, Megan K, MBBS, FRACP | Townsville Hospital & Health Service Governance<br>100 Angus Smith Drive<br>Douglas, Queensland 4814<br>Australia<br><br>Cairns and Hinterland Hospital & Health Service Governance Professional House<br>88 Abbott Street |

| Site no. | Principal investigator name | Name and address of IRB/IEC and IRB/IEC chairperson or FWA number |
|---|---|---|
| | | PO Box 902<br>Cairns, Queensland 4870<br>Australia<br><br>Townsville Health Service District Ethics Committee<br>Executive Office, The Townsville Hospital<br>PO Box 670<br>Townsville, Queensland 4810<br>Australia<br><br>Chairperson: Smyth, Wendy, MD |
| CA209-76K-0106[a] | Haydon, Andrew M, MBBS | St Vincent's Hospital (Sydney) HREC<br>Translational Research Centre<br>97-105 Boundary Street<br>Darlinghurst, New South Wales 2010<br>Australia<br><br>Chairperson: Brien, Jo-Anne, MBBS |
| CA209-76K-0107[a] | Westgeest, Hans, MD, PhD<br>Ten Tije, A.J. J, MD (Previous) | Universitair Medisch Centrum Groningen<br>Medisch Ethische Toetsinscommissie<br>T.a.v. Mr. J.w.p. De Vroedt Mha Ambtelijk Secretaris<br>Hanzeplein 1<br>Groningen, 9713 GZ<br>Netherlands<br><br>Chairperson:<br>Kamps, WA, MD, PhD |
| CA209-76K-0109 | Suga, Jennifer Marie, MD | Kaiser Foundation Research Institute, Kaiser Permanente Northern California Institutional Rev<br>1800 Harrison Street, 10th Floor<br>Oakland, CA 94612<br>United States<br><br>FWA Number FWA0000241 |
| CA209-76K-0110[a] | Mattila, Kalle Ensio, MD | Joint Authority Administration Biomedicum Helsinki 2C<br>PL 705<br>Helsinki, 00029<br>Finland<br><br>Chairperson: Marjamaa, Johan, MD |
| CA209-76K-0111[a] | Mortier, Laurent Pierre, MD | C.P.P. Sud-Est Iv Centre Leon Berard<br>28 rue Laennec<br>Lyon Cedex 08, 69373<br>France |

| Site no. | Principal investigator name | Name and address of IRB/IEC and IRB/IEC chairperson or FWA number |
|---|---|---|
| | | Chairperson: Bertrand Reynaud, Amandine, MD |
| CA209-76K-0112[a] | Dutriaux, Caroline, MD | C.P.P. Sud-Est Iv Centre Leon Berard<br>28 rue Laennec<br>Lyon Cedex 08, 69373<br>France<br><br>Chairperson: Bertrand Reynaud, Amandine, MD |
| CA209-76K-0113[a] | Legoupil, Delphine, MD | C.P.P. Sud-Est Iv Centre Leon Berard<br>28 rue Laennec<br>Lyon Cedex 08, 69373<br>France<br><br>Chairperson: Bertrand Reynaud, Amandine, MD |
| CA209-76K-0114[a] | Thoms, Kai-Martin, MD | Ethikkommission Der Med. Fakultaet Alte Glockengiesserei 11/1 Heidelberg, 69115<br>Germany<br><br>Chairperson: Strowitzki, Thomas, MD, PhD |
| CA209-76K-0116[a] | Jamal, Rahima, MD, FRCP | Le Comite d'ethique de la recherche du CIUSSS De L'Estrie-CHUS<br>3001 12e Avenue Nord<br>Sherbrooke, QC, J1H 5N4<br>Canada<br><br>Chairpersons: Cumyn, Annabelle, MD<br>Bernardi, Marie-Josee, MD, PhD (Previous) |
| CA209-76K-0119 | Suga, Jennifer Marie, MD[b]<br>Truong, Thach-giao, MD (Previous) | Kaiser Foundation Research Institute, Kaiser Permanente Northern California Institutional Rev<br>1800 Harrison Street, 10th Floor<br>Oakland, CA 94612<br>United States<br><br>FWA Number: FWA0000241 |
| CA209-76K-0120 | Suga, Jennifer Marie, MD[b]<br>Truong, Thach-giao, MD (Previous) | Kaiser Foundation Research Institute, Kaiser Permanente Northern California Institutional Rev<br>1800 Harrison Street, 10th Floor<br>Oakland, CA 94612<br>United States<br><br>FWA Number: FWA0000241 |
| CA209-76K-0121 | Suga, Jennifer Marie, MD[b] | Kaiser Foundation Research Institute, Kaiser Permanente Northern California (KPNC) Institutional Rev<br>1800 Harrison Street, 10th Floor<br>Oakland, CA 94612-3433 United States<br><br>FWA Number: FWA0000241 |

| Site no. | Principal investigator name | Name and address of IRB/IEC and IRB/IEC chairperson or FWA number |
|---|---|---|
| CA209-76K-0122 | Suga, Jennifer Marie, MD | Kaiser Foundation Research Institute, Kaiser Permanente Northern California Institutional Rev<br>1800 Harrison Street, 10th Floor<br>Oakland, CA 94612<br>United States<br><br>FWA Number: FWA0000241 |
| CA209-76K-0123[a] | Hanel, Robert, MD | Le Comite d'Ethique de la recherche du CIUSSS de l'Estrie-CHUS<br>3001 12e Avenue Nord<br>Sherbrooke, QC, J1H 5N4<br>Canada<br><br>Chairpersons: Cumyn, Annabelle, MD<br>Bernardi, Marie-Josee, MD (Previous) |
| CA209-76K-0124[a] | Baetz, Tara, MD | Queen'S University Hlth Sc. & Aff.Teaching Hosp<br>78 Fifth Field Company Lane<br>Kingston, ON K7L 3N6<br>Canada<br><br>Chairperson: Clark, Albert, MD |
| CA209-76K-0125[a] | Lomax, Anna, MBBS | Barwon Health Research Ethics Governance & Integrity Unit<br>The Geelong Hospital<br>PO Box 281<br>Geelong, Victoria 3220<br>Australia<br><br>St Vincent's Hospital (Sydney) HREC<br>Translational Research Centre<br>97-105 Boundary Street<br>Darlinghurst, New South Wales 2010<br>Australia<br><br>Chairperson: Brien, Jo-Anne, MBBS |
| CA209-76K-0126 | Shaheen, Montaser, MD | Western IRB<br>1019 39th Avenue SE<br>Suite 120<br>Puyallup, WA 98374<br>United States<br><br>FWA Number: FWA00000533 |
| CA209-76K-0127 | Ott, Patrick, MD, PhD | Dana Farber Cancer Institute IRB<br>450 Brookline Avenue, OS229<br>Boston, MA 02215<br>United States<br><br>FWA Number: FWA00000533 |

| Site no. | Principal investigator name | Name and address of IRB/IEC and IRB/IEC chairperson or FWA number |
|---|---|---|
| CA209-76K-0128[a] | Brady, Benjamin, MBBS, FRACP[b] | Cabrini Governance Office<br>154 Wattletree Road<br>Malvern, Victoria 3144<br>Australia<br><br>Monash Health HREC<br>Monash Medical Centre<br>246 Clayton Road<br>Clayton, Victoria 3168<br>Australia<br><br>Chairperson: Bower, Simon, MBBS |
| CA209-76K-0129[a] | Nardin, Charlee, MD | C.P.P. Sud-Est Iv Centre Leon Berard<br>28 rue Laennec<br>Lyon Cedex 08, 69373<br>France<br><br>Chairperson: Bertrand Reynaud, Amandine, MD |
| CA209-76K-0130[a] | Montaudie, Henri, MD | C.P.P. Sud-Est Iv Centre Leon Berard<br>28 rue Laennec<br>Lyon Cedex 08, 69373<br>France<br><br>Chairperson: Bertrand Reynaud, Amandine, MD |
| CA209-76K-0131[a] | Macfarlane, Robyn J, MD | Nova Scotia Health Authority Research Ethics Board<br>5790 University Avenue<br>Halifax, NS B3H 1V7<br>Canada<br><br>Chairperson: Macknight, Christopher, MD |
| CA209-76K-0132 | Chandra, Sunandana, MD<br>Sosman, Jeffrey A, MD (Previous) | Western IRB<br>1019 39th Avenue SE<br>Suite 120<br>Puyallup, WA 98374<br>United States<br><br>FWA Number: FWA00000533 |
| CA209-76K-0133[a] | Metcalf, Corey, MD[b] | UBC BC Cancer Research Ethics Board<br>Fairmont Medical Building<br>750 West Broadway<br>Suite 1315<br>Vancouver, BC V5Z 1J3<br>Canada<br><br>Chairperson: Gelmon, Karen, MD |
| CA209-76K-0134[a] | Monzon, Jose E, MD | Health Research Ethics Board of Alberta<br>1500 10104 –103 Avenue NW |

| Site no. | Principal investigator name | Name and address of IRB/IEC and IRB/IEC chairperson or FWA number |
|---|---|---|
| | | Edmonton, AB T5J 4A7<br>Canada<br><br>Chairperson: Dewhurst, Dale, MD |
| CA209-76K-0135 | Gibney, Geoffrey Thomas, MD[b] | MedStar Health Research Institute<br>Georgetown University Oncology IRB<br>3900 Reservoir Road NW<br>SW104 Med-Dent Building<br>Washington, DC 20057<br>United States<br><br>Chairperson: Kelly Fitzpatrick MD |
| CA209-76K-0138[a] | Hill, Andrew Graham, MBBS | Bellberry HREC<br>129 Glen Osmond Road<br>Eastwood, South Australia 5063<br>Australia<br><br>Chairpersons: Brian, Stofell, MD<br>Slee, Mark, MD (Previous) |
| CA209-76K-0140[a] | Saibil, Sam, MD | University Health Network Research Ethics Board<br>700 University Avenue<br>Toronto, ON, M5G 1Z5<br>Canada<br><br>Chairperson:<br>Hogg, David Hartwick, MD |
| CA209-76K-0141 | Kounalakis, Nicole, MD | Western IRB<br>1019 39th Avenue SE<br>Suite 120<br>Pullyap, WA 98374<br>United States<br><br>FWA Number: FWA00000533 |
| CA209-76K-0142[a] | McWhirter, Elaine, MD<br>Healy, Brian, MD (Previous) | Hamilton Integrated Research Ethics Board<br>293 Wellington Street North<br>Hamilton, ON L8L 8E7<br>Canada<br><br>Chairperson: Inman, Mark, MD |
| CA209-76K-0143 | Ott, Patrick, MD, PhD[b] | Dana Farber Cancer Institute IRB<br>450 Brookline Avenue, OS229<br>Boston, MA 02215<br>United States<br><br>FWA Number: FWA00001121 |
| CA209-76K-0144 | Yorio, Jeffrey T, MD | Copernicus Group IRB 5000<br>Centregreen Way |

| Site no. | Principal investigator name | Name and address of IRB/IEC and IRB/IEC chairperson or FWA number |
|---|---|---|
| | | Suite 200<br>Cary, NC 27513<br>United States<br><br>FWA Number: FWA00000533 |
| CA209-76K-0145[a] | Mandala, Mario, MD | Comitato Etico Aziende Sanitarie Umbria<br>Regione Umbria<br>Palazzo Broletto, 3° piano Via Angeloni, 61<br>Perugia, 06124<br>Italy<br><br>Chairperson: Agnelli, Giancarlo, MD |
| CA209-76K-0146[a] | Queirolo, Paola, MD | Comitato Etico IRCCS IEO e Centro Cardiologico Monzino<br>Via Ripamonti 435<br>Milano, 20141<br>Italy<br><br>Chairperson: Cerutti, Sergio |
| CA209-76K-0148 | Portnoy, David C, MD | Western IRB<br>1019 39th Avenue SE<br>Suite 120<br>Puyallup, WA 98374<br>United States<br><br>FWA Number: FWA00000533 |

[a]Non-IND sites. [b]Indicates sites with drug shipped but zero enrollment. FWA, federal-wide assurance; HREC, human research ethics committee; IEC, independent ethics committee; IND, investigational new drug; IRB, institutional review board.

**Supplementary Fig. 1 | Recurrence-free survival analysis excluding patients with new primaries.**

Shown is Kaplan–Meier estimates of RFS in the intention-to-treat population excluding new primary melanomas and melanoma *in situ* as events. NA, not available; NR, not reached; RFS, recurrence-free survival.

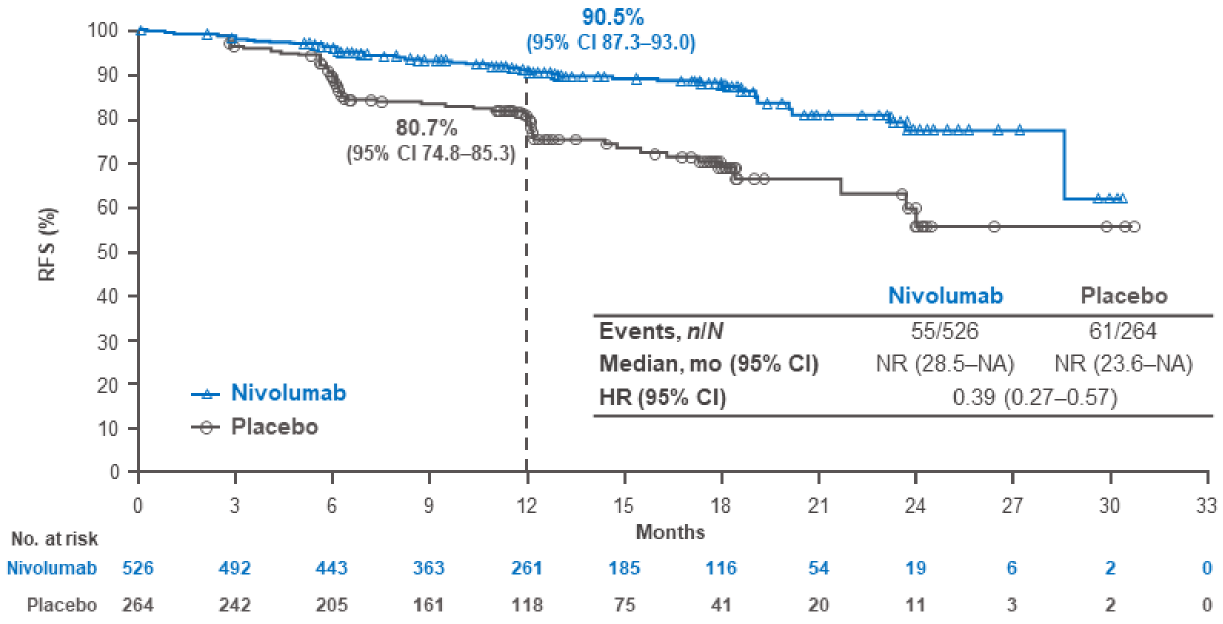

**Supplementary Fig. 2 | Course of disease and therapy in patients with a recurrence event.**

Figure shows course of disease and therapy for patients who had a recurrence or death after the randomization date and before subsequent anticancer therapy or second nonmelanoma primary cancer. LR/NPM/MIS, local recurrence, new primary invasive melanoma, or melanoma *in situ*.

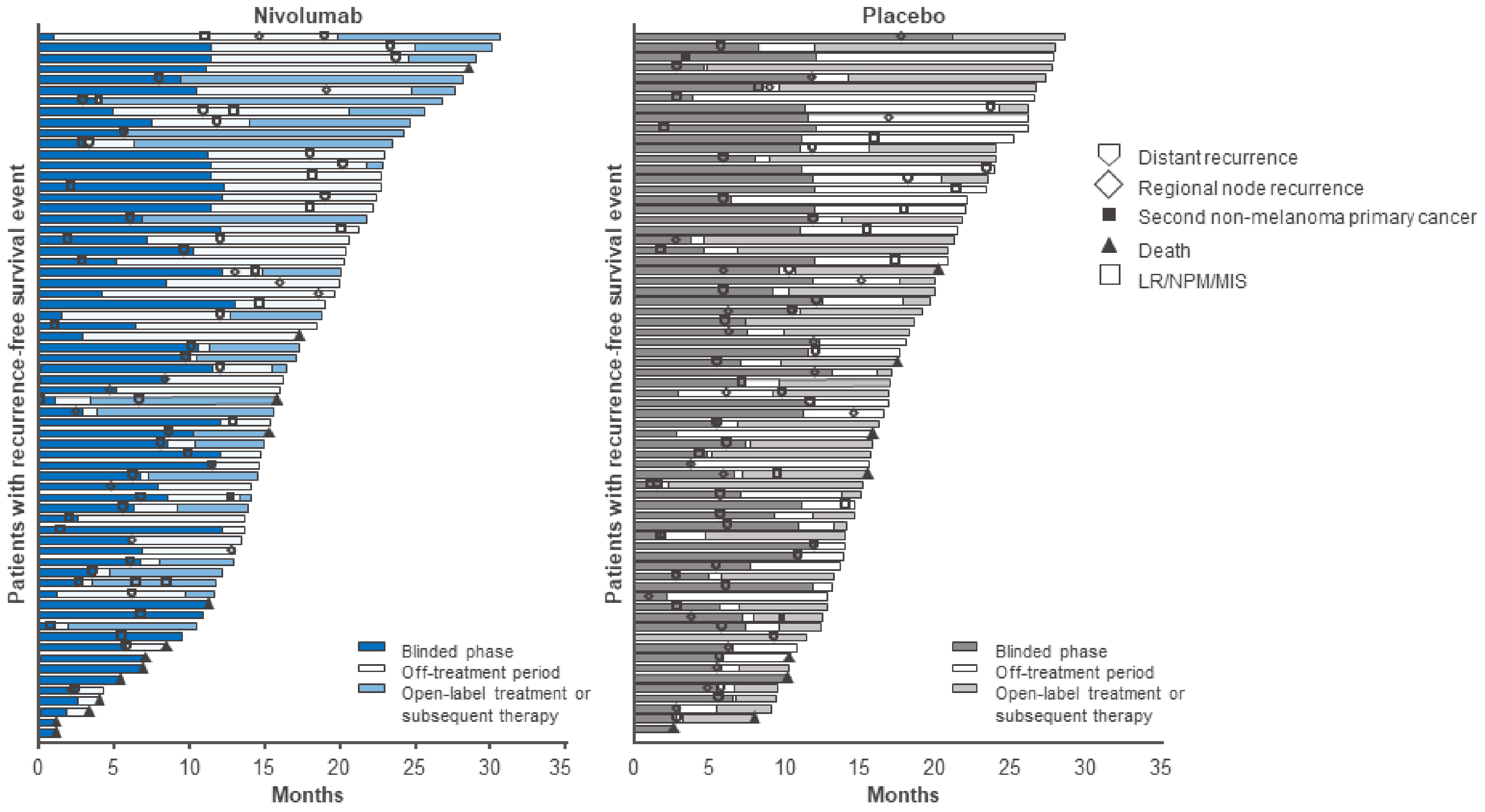

Protocol Number: CA20976K
IND Number: 115,195
EUDRACT Number: 2019-001230-34
Date: 13-May-2019
Revised Date: 28-Apr-2022

# CLINICAL PROTOCOL CA20976K

A Phase 3, Randomized, Double-Blind Study of Adjuvant Immunotherapy with Nivolumab versus Placebo after Complete Resection of Stage IIB/C Melanoma

(CheckMate 76K: CHECKpoint pathway and nivoluMAb clinical Trial Evaluation 76K)

**Short Title:**

Efficacy Study of Nivolumab Compared to Placebo in Prevention of Recurrence of Melanoma After Complete Resection of Stage IIB/C Melanoma (CheckMate 76K)

**Protocol Amendment Number: 03**

**Clinical Trial Physician - Medical Monitor**
[redacted]
Uxbridge Business Park, Sanderson Road
Uxbridge, UB8 1DH, UK
Telephone (office): [redacted]

**Clinical Scientist**
[redacted]
86 Morris Ave
Summit-East, NJ 07901
Telephone (mobile): [redacted]

**24-hr Emergency Telephone Number**
USA: [redacted]
International: [redacted]

**Bristol-Myers Squibb Research and Development**
3401 Princeton Pike
Lawrenceville, NJ 08648
Avenue de Finlande 4
B-1420 Braine-l'Alleud, Belgium

**This document is the confidential and proprietary information of Bristol-Myers Squibb Company and its global affiliates (BMS). By reviewing this document, you agree to keep it confidential and to use and disclose it solely for the purpose of assessing whether your organization will participate in and/or the performance of the proposed BMS sponsored study. Any permitted disclosures will be made only on a confidential "need to know" basis within your organization or to your independent ethics committee(s). Any other use, copying, disclosure or dissemination of this information is strictly prohibited unless expressly authorized in writing by BMS. Any supplemental information (eg, amendments) that may**

**be added to this document is also confidential and proprietary to BMS and must be kept in confidence in the same manner as the contents of this document. Any person who receives this document without due authorization from BMS is requested to return it to BMS or promptly destroy it. All other rights reserved. References to BMS in this protocol may apply to partners to which BMS has transferred obligations, eg, a Contract Research Organization (CRO).**



Approved v1.0 930137673 4.0

# DOCUMENT HISTORY

| Document | Date of Issue | Summary of Change |
|---|---|---|
| Protocol Amendment 03 | 28-Apr-2022 | • Study personnel updated.<br>• Definition of progression-free survival through next-line therapy (PFS2) was rephrased.<br>• Revised overall survival (OS) estimates and added formal OS interim analysis.<br>• Edited text throughout to refer to the number of Recurrence-free Survival and OS events to be observed as “approximately” instead of “at least.”<br>• Other clarifications and administrative/editorial updates made throughout the protocol. |
| Protocol Amendment 02 | 15-Oct-2021 | • Protocol updated to align the management of adverse events (AEs) in trial participants, as well as the reporting of such AEs, per the Common Terminology Criteria for Adverse Events (CTCAE) version 5.0.<br>• Language was inserted to provide descriptive OS data at the time of positive read out of the primary endpoint (recurrence-free survival [RFS]), as well as the projected number of deaths at the time of interim and final RFS analysis.<br>• Clarifications to eligibility criteria regarding: (a) handling of indeterminate nodules found during screening; (b) deletion of requirement for male contraception, and appropriate adjustments to text under the Age and Reproductive Status criteria; (c) extension of pregnancy testing window up to 72 hours prior to treatment start in situations where results cannot be obtained within 24 hours; and (d) flexibility in allowing enrollment of participants who may not have completed the full dosing schedule of coronavirus disease 2019 (COVID-19) vaccines.<br>• Appendices 1, 2, 3, 4, and 5 were updated.<br>• Other clarifications and administrative/editorial updates made throughout the protocol. |
| Administrative Letter 02 | 12-Jul-2021 | • Study personnel updated. |
| Revised Protocol 01 | 16-Oct-2020 | • Revised accrual rate assumptions. Updated the Interim Analysis for Recurrence-Free Survival (RFS) analysis to be conducted at increased number of events (80% Information Fraction). Inserted condition for final RFS analysis to be conducted at 90% of planned events in case event rate is slower than anticipated.<br>• Adjusted the sample size from [redacted] to [redacted] patients to align with revised statistical parameters.<br>[redacted] |



Approved v4.0 930137673 4.0

| Document | Date of Issue | Summary of Change |
|---|---|---|
| | | • Clarified body imaging language regarding imaging of extremities.<br>[redacted]<br>• Inserted rationale for continued adjuvant study therapy for participants diagnosed with melanoma in situ.<br>[redacted]<br>• Inserted text for criteria to resume study treatment in participants with confirmed or suspected SARS-CoV-2 infection.<br>• Inserted text requiring cytological/histological confirmation of melanoma recurrence when clinically feasible and safe.<br>• Inserted text regarding determination of date of recurrence of melanoma regardless of method used to determine recurrence.<br>• Pharmacokinetics: clarified timing of sample draw for End of Infusion timepoint.<br>[redacted]<br>• Updated the management algorithms and added myocarditis algorithm to Appendix 5. Clarified that reporting of AEs is per CTCAE v5, however, management of AEs is per CTCAE v4.<br>• Clarified the reporting of AEs/SAEs in the blinded and open-label portions of the trial. Introduced text on the collection of AEs/SAEs related to SARS-CoV-2 infection. |



Approved v1.0 930137673 4.0

| Document | Date of Issue | Summary of Change |
|---|---|---|
| | | • Changes as per Amendment 03 (for sites in Norway) were added as part of this revision.<br>• Other typographical/editorial changes throughout. |
| Administrative Letter 01 | 04-Oct-2019 | **Page 159 – Appendix 9 – Country-Specific Appendix**<br>*Previously written:*<br>Criteria for exclusion of HIV-positive subjects in Argentina, Czech Republic, France, Germany, Italy, Spain, and any other countries where exclusion of HIV-positive participants is locally mandated.<br>*Changed to:*<br>Criteria for exclusion of HIV-positive subjects in Argentina, Czech Republic, Germany, and any other countries where exclusion of HIV-positive participants is locally mandated. |
| Original Protocol | 13-May-2019 | Not applicable |



Approved v1.0 930137673 4.0

## OVERALL RATIONALE FOR PROTOCOL AMENDMENT 03:

[redacted]

| SUMMARY OF KEY CHANGES FOR PROTOCOL AMENDMENT 03 | | |
|---|---|---|
| **Section Number and Title** | **Description of Change** | **Brief Rationale** |
| Title Page | Changed the name and contact information of the Clinical Scientist. | Updated Clinical Scientist per study personnel change. |



Approved v1.0 930137673 4.0

| SUMMARY OF KEY CHANGES FOR PROTOCOL AMENDMENT 03 | | |
|---|---|---|
| **Section Number and Title** | **Description of Change** | **Brief Rationale** |
| Section 2: Schedule of Activities<br>Table 2-2: On-Treatment Assessments CA20976K (Applies to All Participants: Blinded or Open-label Treatment)<br>Biopsy of affected organs<br><br>Dispense Study Treatment | Included a statement regarding the optional procedure to biopsy affected organs "Upon occurrence of ≥ Grade 3 drug-related AE and/or lab abnormalities regarded as a drug-related SAE."<br><br>Included statement to use Interactive Response Technology (IRT) to dispense study treatment. | Text added in the "On-Treatment" table (Table2-2), since a ≥ Grade 3 drug-related adverse event (AE) and/or lab abnormality regarded as a drug-related serious adverse event (SAE) could happen during treatment as well as post-treatment discontinuation (optional biopsy text reflecting the post-treatment discontinuation period is already present in the long-term follow-up table [Table2-3]).<br><br>Text was inserted to serve as a reminder for the site to use the IRT to dispense study treatment. |
| Section 2: Schedule of Activities<br>Table 2-3: Long-Term Follow-Up CA20976K (Applies to All Participants: Blinded or Open-label Treatment)<br>Survival Status | Modified the length of follow-up from "5 years" to "until the OS final analysis." | Modification of follow-up visits was completed for consistency with other sections. |
| [redacted] | [redacted] | |



Approved v1.0 930137673 4.0

## SUMMARY OF KEY CHANGES FOR PROTOCOL AMENDMENT 03

| Section Number and Title | Description of Change | Brief Rationale |
|---|---|---|
| Section 4: Objectives and Endpoints<br>Table 4-1: Objectives and Endpoints | Definition of progression-free survival through next-line therapy (PFS2) was rephrased. | Definition was editorially rephrased for better clarity. |
| Section 5.1.1: Nivolumab or Placebo Blinded Treatment<br>Survival Status Follow-Up (in-person or by telephone)<br>Section 8.1.1: Post Study Treatment Study Follow-up | Changed survival status follow-up from "up to 5 years" to "until the OS final analysis." | Update was made to align with the anticipated timing of the final OS analysis [redacted] |
| Section 10.1.1: RFS | Edited text throughout to refer to the number of RFS events to be observed as "approximately" instead of "at least." | Changes were made to consistently refer to the number of RFS events to be observed as the "approximate: number of events. Previously, text was inconsistently listed in some places referring to the number of events to be observed as "at least" and in some other places as "approximately." |
| Section 10.1.2: Overall Survival<br>[redacted] | Edited text throughout to refer to the number of OS events to be observed as "approximately" instead of "at least." | Changes were made to consistently refer to the number of OS events to be observed as the "approximate: number of events. Previously, text was inconsistently listed in some places referring to the number of events to be observed as "at least" and in some other places as "approximately." |
| | [redacted] | |



Approved v1.0 930137673 4.0

| SUMMARY OF KEY CHANGES FOR PROTOCOL AMENDMENT 03 | | |
|---|---|---|
| **Section Number and Title** | **Description of Change** | **Brief Rationale** |
| [redacted] | | |
| Section 10.3.1.2 Secondary Endpoint Analyses<br>Investigator-Assessed Outcomes on Next-Line Therapies | Definition of PFS2 was rephrased. | Definition was editorially rephrased for better clarity. |
| Section 10.3.4: Interim Analysis | [redacted]<br>Updated statistical parameters (number of events, power, cHR, and projected number of deaths at the time of the OS interim analyses). | [redacted]<br>Updates were made based on real-word survival data in the Stage IIB/C study population, and accounting additionally for the impact of post-recurrence anti-cancer therapy. |



Approved v1.0 930137673 4.0

| SUMMARY OF KEY CHANGES FOR PROTOCOL AMENDMENT 03 | | |
|---|---|---|
| **Section Number and Title** | **Description of Change** | **Brief Rationale** |
| All | Edited minor formatting and typographical corrections throughout protocol. | Minor, therefore, have not been summarized. |



Approved v4.0 930137673 4.0

# TABLE OF CONTENTS

TITLE PAGE ..... 1
DOCUMENT HISTORY ..... 3
OVERALL RATIONALE FOR PROTOCOL AMENDMENT 03: ..... 6
SUMMARY OF KEY CHANGES FOR PROTOCOL AMENDMENT 03 ..... 6
TABLE OF CONTENTS ..... 11
1 SYNOPSIS ..... 16
2 SCHEDULE OF ACTIVITIES ..... 25
3 INTRODUCTION ..... 39
3.1 Study Rationale ..... 39
*3.1.1 Overview* ..... 39
*3.1.2 Research Hypothesis* ..... 39
3.2 Background ..... 39
*3.2.1 Pediatric Melanoma* ..... 40
*3.2.2 The Adjuvant Treatment of Patients with Stage IIB/C Melanoma with Nivolumab is a Promising Strategy* ..... 42
*3.2.2.1 Harnessing the Success of Immunotherapy in Patients with Late Stage Melanoma to Patients with Stage IIB/C Melanoma* ..... 43
*3.2.3 Nivolumab Mechanism of Action* ..... 49
3.3 Benefit/Risk Assessment ..... 50
4 OBJECTIVES AND ENDPOINTS ..... 51
5 STUDY DESIGN ..... 53
5.1 Overall Design ..... 53
*5.1.1 Nivolumab or Placebo Blinded Treatment* ..... 53
*5.1.2 Optional On-Protocol Open-Label Nivolumab Treatment After First Recurrence* ..... 56
*5.1.3 Data Monitoring Committee and Other External Committees* ..... 57
5.2 Number of Participants ..... 58
5.3 End of Study Definition ..... 58
5.4 Scientific Rationale for Study Design ..... 58
*5.4.1 Rationale for Treatment with Blinded Nivolumab* ..... 59
[redacted]
*5.4.5 Rationale for Blinding (Nivolumab Versus Placebo)* ..... 63
[redacted]



Approved v4.0 930137673 4.0

5.5 Justification for Dose ........ 69
5.5.1 Rationale for 480 mg Flat Dose Every 4 Weeks ........ 69
[redacted]
5.5.3 Rationale for Nivolumab 30-Minute Infusion Duration ........ 71
6 STUDY POPULATION ........ 71
6.1 Inclusion Criteria ........ 71
6.1.1 Inclusion Criteria - Blinded Nivolumab or Placebo Treatment ........ 71
6.1.2 Inclusion Criteria for Participants Receiving Treatment after Recurrence ........ 74
6.2 Exclusion Criteria ........ 75
6.2.1 Exclusion Criteria - Blinded Nivolumab or Placebo Treatment ........ 75
6.2.2 Exclusion Criteria for Participants Receiving Treatment After Recurrence ........ 77
6.3 Lifestyle Restrictions ........ 77
6.4 Screen Failures ........ 77
6.4.1 Retesting During Screening or Lead-In Period ........ 77
7 TREATMENT ........ 78
7.1 Treatments Administered ........ 81
7.1.1 Treatment with Nivolumab or Placebo ........ 81
7.1.2 General Treatment Administration Considerations ........ 81
7.2 Method of Treatment Assignment ........ 82
7.2.1 Blinded Nivolumab or Placebo Treatment ........ 82
7.2.2 Optional Open-Label Nivolumab Treatment After First Recurrence ........ 83
7.3 Blinding ........ 83
7.3.1 Unblinding: Emergency, Accidental, and in the Event of Disease Recurrence ........ 84
7.3.2 Unblinding in the Event of Disease Recurrence ........ 84
7.4 Dosage Modification ........ 85
7.4.1 Criteria to Delay, Resume, or Discontinue Nivolumab or Nivolumab-Placebo ........ 85
7.4.1.1 Additional Dose Delay Criteria/Details ........ 90
7.4.1.2 Additional Dose Resumption Criteria/Details ........ 91
7.4.1.3 Additional Dose Discontinuation Criteria/Details ........ 91
7.4.2 Treatment of Nivolumab-Related Infusion Reactions ........ 92
7.5 Preparation/Handling/Storage/Accountability ........ 93
7.5.1 Retained Samples for Bioavailability / Bioequivalence / Biocomparability ........ 94
7.6 Treatment Compliance ........ 94
7.7 Concomitant Therapy ........ 94
7.7.1 Prohibited and/or Restricted Treatments ........ 94
7.7.2 Other Restrictions and Precautions ........ 95
7.7.3 Imaging Restriction and Precautions ........ 95
7.7.4 Permitted Therapy ........ 95
7.8 Treatment After the End of the Study ........ 96
7.8.1 Treatment Beyond Disease Progression ........ 96



Approved v1.0 930137673 4.0

7.8.1.1 Participants Receiving Optional Open-Label Nivolumab with Unresectable/Metastatic Disease (Arm 2) ........ 96
8 DISCONTINUATION CRITERIA ........ 97
8.1 Discontinuation from Study Treatment ........ 97
8.1.1 Post Study Treatment Study Follow-up........ 98
8.2 Discontinuation from the Study ........ 98
8.3 Lost to Follow-Up........ 98
9 STUDY ASSESSMENTS AND PROCEDURES ........ 99
9.1 Efficacy Assessments........ 100
9.1.1 Imaging Assessment for the Study........ 100
9.1.1.1 Methods of Measurement........ 100
9.1.2 Investigator Assessment of Baseline Disease Status........ 101
9.1.2.1 General Considerations for Determining Recurrence........ 101
9.1.2.2 Investigator Assessment of Recurrence (Nivolumab/Placebo-Blinded Treatment and Open-Label Nivolumab - Arm 1) ........ 102
9.1.2.3 Definitions........ 103
9.1.2.4 Date of Recurrence ........ 104
9.1.2.5 Investigator Assessment of Progression for Participants Receiving Open-Label Nivolumab (Arm 2: Unresectable/Metastatic Disease) .... 104
[redacted]
9.2 Adverse Events ........
9.2.1 Time Period and Frequency for Collecting AE and SAE Information .... 106
9.2.2 Method of Detecting AEs and SAEs........ 108
9.2.3 Follow-up of AEs and SAEs........ 108
9.2.4 Regulatory Reporting Requirements for SAEs........ 108
9.2.5 Pregnancy ........ 109
9.2.6 Laboratory Test Result Abnormalities ........ 110
9.2.7 Potential Drug Induced Liver Injury (DILI) ........ 110
9.2.8 Other Safety Considerations ........ 110
9.3 Overdose ........ 110
9.4 Safety ........ 110
9.4.1 Physical Examinations........ 111
9.4.2 Vital signs........ 111
9.4.3 Electrocardiograms ........ 111
9.4.4 Clinical Safety Laboratory Assessments........ 111
9.4.5 Imaging Safety Assessment ........ 112
9.5 Pharmacokinetic and Immunogenicity Assessments ........ 112
9.6 Pharmacodynamics ........ 115
9.7 Pharmacogenomics ........ 115
[redacted]
9.8.1 Additional Research Collection ........ 118
9.8.2 Tissue Specimens ........ 119
[redacted]



Approved v1.0 930137673 4.0

[redacted]

9.10 Other Assessments ........ 123
10 STATISTICAL CONSIDERATIONS ........ 123
10.1 Sample Size Determination ........ 123
*10.1.1 RFS* ........ 123
*10.1.2 Overall Survival* ........ 124
10.2 Populations for Analyses ........ 126
10.3 Statistical Analyses ........ 126
*10.3.1 Efficacy Analyses* ........ 126
*10.3.1.1 Primary Endpoint Analysis* ........ 126
*10.3.1.2 Secondary Endpoint Analyses* ........ 127
*10.3.2 Safety Analyses* ........ 128
*10.3.3 Other Analyses* ........ 129
[redacted]
*10.3.3.3 Pharmacokinetic Analyses* ........ 129
[redacted]
*10.3.3.5 Immunogenicity Analyses* ........ 130
*10.3.3.6 Analyses of Follow-Up Data from Participants Receiving Optional Open-Label Nivolumab Treatment* ........ 130
*10.3.4 Interim Analyses* ........ 130
11 REFERENCES ........ 131
12 APPENDICES ........ 139
APPENDIX 1 ABBREVIATIONS AND TRADEMARKS ........ 140
APPENDIX 2 STUDY GOVERNANCE CONSIDERATIONS ........ 146
APPENDIX 3 ADVERSE EVENTS AND SERIOUS ADVERSE EVENTS: DEFINITIONS AND PROCEDURES FOR RECORDING, EVALUATING, FOLLOW-UP, AND REPORTING ........ 156
APPENDIX 4 WOMEN OF CHILDBEARING POTENTIAL DEFINITIONS AND METHODS OF CONTRACEPTION ........ 161
APPENDIX 5 MANAGEMENT ALGORITHMS FOR STUDIES UNDER CTCAE VERSION 5.0 ........ 165
APPENDIX 6 ECOG PERFORMANCE STATUS ........ 174
APPENDIX 7 RESPONSE EVALUATION CRITERIA IN SOLID TUMORS GUIDELINES (VERSION 1.1) WITH BMS MODIFICATIONS ........ 175
APPENDIX 8 AJCC MELANOMA STAGING ........ 183
APPENDIX 9 COUNTRY SPECIFIC REQUIREMENTS ........ 187



Approved v4.0 930137673 4.0

APPENDIX 10 PROTOCOL AMENDMENT SUMMARY OF CHANGE HISTORY .......... 188

# 1 SYNOPSIS

**Protocol Title: A Phase 3, Randomized, Double-Blind Study of Adjuvant Immunotherapy with Nivolumab versus Placebo after Complete Resection of Stage IIB/C Melanoma**

**Short Title:**

Efficacy Study of Nivolumab Compared to Placebo in Prevention of Recurrence of Melanoma After Complete Resection of Stage IIB/C Melanoma (CheckMate 76K)

**Study Phase:** 3

**Study Overview:**

CA20976K is a Phase 3, randomized, double-blind study designed to evaluate the use of adjuvant immunotherapy with nivolumab versus placebo after complete resection of Stage IIB/C melanoma in adults and pediatric participants ≥ 12 years old.

In this study, participants with resected Stage IIB/C melanoma and no evidence of disease will be randomized to treatment with either nivolumab or placebo for a duration of 12 months. Results from this study will help to define the role of nivolumab as an adjuvant immunotherapy in patients with resected Stage IIB and IIC cutaneous melanoma. In the event of disease recurrence, participants will have the option to receive on-study open-label nivolumab treatment. Placebo-treated participants who experience disease recurrence within 3 years after the last dose of placebo, and nivolumab-treated participants who experience recurrence greater than 6 months and within 3 years after completing treatment, will be eligible to participate in on study unblinded /open-label nivolumab treatment. Participants with resectable / recurrent disease will be offered nivolumab for a maximum duration of 12 months, whereas participants with unresectable or metastatic disease will be offered nivolumab monotherapy for a maximum of 24 months. Participants with disease recurrence who do not participate in the optional open-label nivolumab treatment portion of the study will be treated per standard of care as recommended by their treating physician.

**Overall Study Rationale:**

There is currently an unmet medical need for effective adjuvant therapies for patients diagnosed with Stage IIB and IIC cutaneous melanoma. Results from this study will help to define the role of nivolumab as an adjuvant immunotherapy in patients with Stage IIB and IIC cutaneous melanoma.

Cutaneous malignant melanomas are staged according to the American Joint Committee on Cancer (AJCC) tumor/node/metastasis (TNM) classification and staging system. The AJCC 8th edition tumor staging for patients presenting with Stage II cutaneous malignant melanomas is shown below.[1]



Approved v1.0 930137673 4.0

| When T is… | And N is… | And M is…. | The pathological stage is…. |
|---|---|---|---|
| T2b/T3a | N0 | M0 | IIA |
| T3b/T4a | N0 | M0 | IIB |
| T4b | N0 | M0 | IIC |
| T2: >1.0–2.0 mm; T3: >2.0–4.0 mm; T4: >4.0 mm; a: without ulceration; b: with ulceration; N0: No regional metastases detected; M0: No evidence of distant metastasis | | | |

Stage IIB (T3b/T4a, N0, M0) and IIC (T4b, N0, M0) melanomas are classified as localized Stage IIB (T3b/T4a, N0, M0) and IIC (T4b, N0, M0) melanomas are classified as localized melanomas without nodal involvement but are a higher risk category compared to Stage I and IIA melanomas due to a relatively worse prognosis. Patients diagnosed with Stage IIB and IIC melanoma have 5-year melanoma-specific survival (MSS) reported as 87% and 82%, respectively. The corresponding 10-year survival rates have been reported as 82% and 75%, respectively.

The relatively similar prognosis of patients with Stage IIB/C melanoma and patients with Stage IIIB melanoma (5-year MSS for Stage IIIB = 83%), the poorer prognosis for these patients after relapse, underscore the need to prevent local or systemic relapses and provide support for the study of new systemic interventions to prevent metastases in the Stage IIB/C adjuvant melanoma setting.[2] The demonstrated efficacy and acceptable benefit-risk profiles of immunotherapies such as nivolumab in Stage III and IV unresectable/metastatic melanoma, as well as in resected Stage III and IV melanoma, make nivolumab a suitable candidate to study in Stage IIB/C melanoma where the tumor burden is lower and immunotherapies may have a better chance of curing the patient.

In this trial, the effect of re-introducing open-label nivolumab in the event of disease recurrence in participants undergoing initial treatment with nivolumab will be explored. Anecdotal evidence exists for the re-introduction of immunotherapy in similar settings. In addition, placebo participants will also be provided with the opportunity to crossover and receive active treatment in the event of disease recurrence. The open-label component may also increase understanding of the impact of treating with nivolumab in earlier stage disease on subsequent treatment with nivolumab when patients develop more advanced disease.

**Study Population:**

Male and female participants (≥ 12 years of age) with completely resected Stage IIB or IIC melanoma, with no evidence of disease (NED). Note: Where local regulations and/or institutional policies do not allow for participants < 18 years of age, the eligible participant population is ≥ 18 years of age.

Key Inclusion Criteria:

a) Participants must have been diagnosed with Stage IIB/C cutaneous melanoma (AJCC Staging, 8th edition) and have histologically confirmed melanoma that is completely surgically resected, with documented negative margins (per local standard) for disease on



Approved v4.0 930137673 4.0

resected specimens. All melanomas, except ocular and mucosal melanoma, regardless of primary site of disease will be allowed.

b) Complete resection with documented negative margins (per local standard) and sentinel lymph node assessment for presence/absence of disease, must be performed within 12 weeks prior to randomization. Note: In case of delays exceeding 12 weeks due to unforeseen circumstances, the eligibility should be discussed with the Medical Monitor or designee. Participants must have had a negative sentinel lymph node biopsy. Participants in whom a sentinel lymph node biopsy procedure could not be done or a sentinel lymph node was not detected are not eligible.

c) Participants must have disease-free status documented by a complete physical examination (within 14 days) and imaging studies within 4 weeks (28 days) prior to randomization. Imaging studies must include CT scans of the chest/abdomen/pelvis or CT scan of the chest and MRI scans of the abdomen and pelvis, and all known sites of resected disease (imaging of extremities for resected melanomas located in the extremities is not a requirement). The evaluation of extremities may be conducted, and documented, per local standard of care. Participants with signs and symptoms consistent with brain metastases should have imaging studies done to rule out the presence of brain metastases.

d) Participant has not been previously treated for melanoma beyond complete surgical resection of the melanoma lesion.

e) Participant has recovered adequately from toxicity and/or complications from surgery prior to study start.

f) ECOG performance status of 0 or 1 at the time of enrollment.

g) Tumor tissue (minimum of 15 unstained slides, preferably freshly cut, or 1 FFPE block to contain sufficient tissue for at least 15 sections) from the primary diagnostic biopsy must be shipped to the central laboratory prior to randomization. If the required tumor tissue content cannot be provided, the eligibility should be discussed with the Medical Monitor or designee.

Key Exclusion Criteria

1) Medical Conditions

a) History of ocular and mucosal melanoma.

b) Participants with active, known, or suspected autoimmune disease. Participants with type I diabetes mellitus, hypothyroidism only requiring hormone replacement, skin disorders (such as vitiligo, psoriasis, or alopecia) not requiring systemic treatment or conditions not expected to recur in the absence of an external trigger are permitted to enroll.

c) Prior malignancy active within the previous 3 years except for locally curable cancers that have been apparently cured, such as basal or squamous cell skin cancer, superficial bladder cancer, or carcinoma in situ of the prostate, cervix, or breast.

d) Participants with a condition requiring systemic treatment with either corticosteroids (> 10 mg daily prednisone equivalent) or other immunosuppressive medications within 14 days of randomization. Inhaled or topical steroids, and adrenal replacement steroid doses > 10 mg daily prednisone equivalent, are permitted in the absence of active autoimmune disease.

e) Women who are pregnant or breastfeeding.



Approved v1.0 930137673 4.0

f) Participants with serious or uncontrolled medical disorders. Additionally, in the case of prior severe acute respiratory syndrome coronavirus 2 (SARS-CoV-2) infection, symptoms must have completely resolved and based on investigator assessment in consultation with the BMS Medical Monitor or designee, there are no sequelae that would place the participant at a higher risk of receiving investigational treatment.

2) Prior/Concomitant Therapy
   a) Use of an investigational agent or an investigational device within 28 days before administration of first dose of study drug.
   b) Treatment directed against the resected melanoma (eg, chemotherapy, radiation therapy, targeted agents, biotherapy, or limb perfusion) that is administered after the complete resection.
   c) Prior treatment with an anti-PD-1, anti-PD-L1, anti-PD-L2, anti-CD137, anti-CTLA-4 antibody, or agents that target IL-2 pathway any other antibody or drug specifically targeting T-cell co-stimulation or checkpoint pathways. Exception: Prior adjuvant treatment with interferon (for melanoma other than study entry melanoma) is allowed if completed ≥ 6 months prior to randomization.
   d) Treatment with complementary medications (eg, herbal supplements or traditional Chinese medicines) to treat the disease under study within 2 weeks prior to randomization/treatment. Such medications are permitted if they are used as supportive care.
   e) Participants who have received a live / attenuated vaccine within 30 days of first treatment.

Male and female participants with disease recurrence within 3 years of discontinuation of blinded-study treatment (nivolumab or placebo) may be eligible for additional treatment with nivolumab monotherapy. In addition, for nivolumab participants, disease recurrence should be greater than 6 months following 1 year of treatment completion. Detailed eligibility criteria for enrollment to open-label nivolumab are in the main body of the protocol.

**Objectives and Endpoints:**

**Table 1: Objectives and Endpoints[a]**

| **Objectives** | **Endpoints** |
|---|---|
| **Primary** | |
| To compare the efficacy, as measured by recurrence-free survival (RFS), provided by nivolumab monotherapy versus placebo in participants with completely resected Stage IIB/C melanoma with no evidence of disease who are at high risk for recurrence. | RFS |
| **Secondary** | |
| To compare the overall survival (OS) provided by nivolumab monotherapy versus placebo in participants with completely resected Stage IIB/C | OS |



Approved v1.0 930137673 4.0

**Table 1: Objectives and Endpoints[a]**

| Objectives | Endpoints |
|---|---|
| melanoma with no evidence of disease who are at high risk for recurrence. | |
| To assess safety and toxicity of nivolumab monotherapy in participants with completely resected Stage IIB/C melanoma with no evidence of disease. | Occurrence and severity of AEs as defined by NCI CTCAE v5. |
| To evaluate distant metastases-free survival (DMFS) | DMFS |
| To evaluate investigator-assessed outcomes on next-line therapies. | • Objective response rates (if applicable)<br>• Duration of treatment on next-line therapies<br>• Progression-free survival through next-line therapy (PFS2) is defined as the time from randomization to recurrence/objective disease progression after the start of the next-line of systemic anti-cancer therapy, or to the start of a second next-line systemic therapy, or to death from any cause, whichever occurs first.<br>• End-of-next-line-treatment: To be used for situations where PFS2 cannot be reliably determined. Event defined as end or discontinuation of next-line treatment, second objective disease progression, or death from any cause, whichever occurs first. |
| **Exploratory** | |
| [redacted] | |



Approved v4.0 930137673 4.0

**Table 1: Objectives and Endpoints[a]**

| Objectives | Endpoints |
|---|---|
| [redacted] | |
| To characterize the pharmacokinetics (PK) and explore exposure-response relationships with respect to safety and efficacy. | PK parameters and exposure metrics calculated from serum concentration-time data and characterization of exposure-response relationships |
| To characterize the immunogenicity of nivolumab. | Detection of anti-nivolumab antibodies |

Abbreviations: AE, adverse events; [redacted] IgG, immunoglobulin G; [redacted] NCI CTCAE, National Cancer Institute Common Terminology for Adverse Events; OS, overall survival; PFS2, progression-free survival through next line therapy; PK, pharmacokinetics; [redacted]

[a] Objectives and endpoints for participants entering the optional open-label nivolumab are very similar to blinded nivolumab/placebo participants with some subtle differences in endpoints to account for the open-label non-randomized nature of treatment and for participants presenting with recurrent unresectable/metastatic disease in the event of recurrence. [redacted]

**Study Design:**

In this study, participants with completely resected Stage IIB/C melanoma with no evidence of disease, will be randomized (2:1), stratified by T stage, to receive nivolumab (480 mg IV every 4 weeks), or nivolumab-matched placebo until recurrence, unacceptability toxicity, withdrawal of consent, or a maximum of 12 months from the first dose of study treatment, which occurs first.

Following first recurrence (Placebo participants: within 3 years after the last dose; Nivolumab participants: greater than 6 months from completion of 12 months of nivolumab, but within 3 years after completing 12 months of treatment), participants may be eligible to receive optional open-label nivolumab for a maximum of 12 months (resectable disease) or 24 months (for unresectable/metastatic disease). The 12 or 24 months is calculated from the first dose of open-label study treatment.

The study design schematic is presented in Figure 1 below.



Approved v1.0 930137673 4.0

**Figure 1: Study Design Schematic (applicable To Adult and Pediatric Participants ≥ 12 years old)**

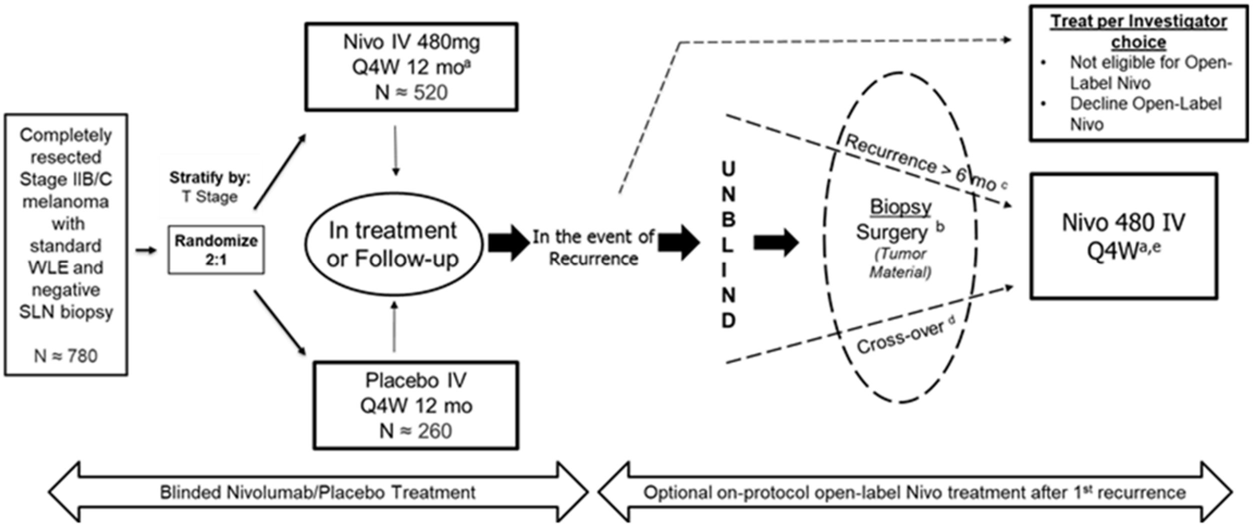


Abbreviations: IV, intravenous; Nivo, nivolumab; Q4W, every 4 weeks; SLN, sentinel lymph node; WLE, wide local excision.

[a] Pediatric dosing weight-based under 40 kg and for sites preferring weight-based dosing in pediatrics

[b] Tumor material obtained from surgery or biopsy

[c] Recurrence > 6 months after completion of 1 year of nivolumab treatment

[d] After recurrence, participants assigned to placebo arm will be offered to cross-over to nivolumab

[e] Resectable disease (Arm 1): Nivo 480 mg IV Q4W for 12 months
Unresectable disease (Arm 2): Nivo 480 mg IV Q4W for 24 months

During both blinded and open-label treatment, assessments of pharmacokinetics, biomarkers, patient-reported outcomes, and imaging will be conducted at the schedules defined in the full protocol. During follow-up of blinded-treatment, participants will continue in-person assessment visits through 1-year post treatment (12 months from Follow-Up Visit 2). Participants will continue to be followed for survival status until the OS final analysis.

**Number of Participants:**

Approximately 780 participants, stratified by AJCC 8th edition T-stage (T3b vs. T4a vs. T4b) will be randomized in a 2:1 ratio to nivolumab 480 mg intravenously (IV) every 4 weeks (Q4W) or nivolumab-matched placebo.

**Treatment Arms and Treatment Duration Details:**

All participants on nivolumab or placebo will be treated until recurrence, unacceptable toxicity, participant withdrawal of consent, or a maximum of 12 months from first dose of study treatment, whichever occurs first. Dosing details for nivolumab and placebo are provided below.



Approved v4.0 930137673 4.0

- **Arm A (Nivolumab):**
  - **Adult Dosing:**
    - ♦ Nivolumab 480 mg IV flat dose over approximately 30 minutes every 4 weeks (Q4W).
  - **Pediatric Dosing:**
    - ♦ 12-17 years of age and weighing ≥ 40 kgs: Nivolumab 480 mg IV Q4W.
    - ♦ 12-17 years of age and weighing < 40 kgs: 6 mg/kg Q4W up to a maximum of 240 mg.
    - ♦ For centers that do not prefer to use flat dosing: 6 mg/kg Q4W (maximum of 240 mg for weight < 40 kgs; maximum of 480 mg for weight ≥ 40 kgs).
    - ♦ All nivolumab infusions to be administered over approximately 30 minutes.
- **Arm B**: Adults and Pediatrics: Nivolumab matched placebo (0.9% Sodium Chloride for Injection/5% Dextrose for Injection) IV over approximately 30 minutes Q4W.

Optional open-label nivolumab will be administered for pediatrics and adults at the dosages described for blinded-treatment above (480 mg IV flat dose Q4 weeks). For centers that do not prefer to use flat dosing, weight-based dosing can be used as mentioned above. Participants will be treated until progression, unacceptable toxicity, withdrawal of consent or a maximum treatment duration defined as 12 months for participants with resectable disease, or 24 months for those with unresectable/metastatic disease (calculated from the first dose of open-label treatment) whichever is first.

**Study Treatments:**

| Medication | Potency | IP/Non-IP |
|---|---|---|
| BMS-936558 (Nivolumab) Solution for Injection | 10 mg/mL | IP |
| 0.9% Sodium Chloride for Injection | N/A | IP |
| 5% Dextrose for Injection | N/A | IP |

**Statistical Considerations:**

The sample size of the study (N ~ 780) is based on a comparison of the RFS distribution between participants randomized to nivolumab and participants randomized to placebo. Simulation models incorporating aspects of immunotherapy such as delayed separation (observed as late separation of survival curves between the experimental and placebo arms) and long-term survival benefits (observed as a long-lasting plateau towards the tail of the survival curve) were developed for sample size estimation. Sample size calculations for this study design were performed using EAST (v 6.4.1).

For this comparison of RFS between nivolumab and placebo in all randomized participants, approximately 154 RFS events would be required for a two-sided experiment-wise alpha = 0.05 log-rank test to show a statistically significant difference in RFS between the treatment arms with



Approved v4.0 930137673 4.0

at least 90% power when the average hazard ratio (HR) of the nivolumab arm to the placebo arm is 0.573 (HR = 1 for the first 6 months, HR = 0.537 after 6 months, HR = 1 from Year 10 [plateau effect]). Given an estimated accrual rate as described in Table 2 below, the accrual of 780 participants (ie, 520 participants in the nivolumab arm and 260 participants in the placebo arm) will take approximately 29.6 months. Under the assumptions for accrual, an assumed delayed treatment effect of 6 months as per the Sunbelt Melanoma Trial[3] and assumed HR as stated above, it would take approximately 68.1 months from the randomization of the first participant to observe the required number of RFS events. An observed HR of 0.707 or less would result in a statistically significant improvement at the final analysis of RFS.

**Table 2: Accrual Rate Assumption**

| Month | 0 | 1 | 2 | 3 | 4 | 5 | 6-9 | 10-14 | 15-17 | 18+ |
|---|---|---|---|---|---|---|---|---|---|---|
| Accrual Rate[a] | 2 | 10 | 13 | 21 | 30 | 10 | 0[b] | 6 | 28 | 50 |

[a] Assume 5% drop-out (lost to follow-up) rate in each arm by Month 50.

[b] 0.001 in East that doesn't allow 0 technically.

**Data Monitoring Committee:** Yes

## References:

1 Gershenwald J, Scolyer R, Hess K, et al. Melanoma Staging: Evidence-Based Changes in the American Joint Committee on Cancer Eighth Edition Cancer Staging Manual. CA Cancer J Clin. 2017;67(6):472–492.

2 Grob J, Garbe C, Ascierto P, et al. Adjuvant melanoma therapy with new drugs: should physicians continue to focus on metastatic disease or use it earlier in primary melanoma? Lancet Oncol. 2018;19:e720-25.

3 McMasters KM, Egger ME, Edwards MJ, et al. Final Results of the Sunbelt Melanoma Trial: A Multi-Institutional Prospective Randomized Phase III Study Evaluating the Role of Adjuvant High-Dose Interferon Alfa-2b and Completion Lymph Node Dissection for Patients Staged by Sentinel Lymph Node Biopsy. J Clin Oncol. 2016;34:1079-1086.



Approved v4.0 930137673 4.0

# 2 SCHEDULE OF ACTIVITIES

The following schedules of activities are provided:

Table 2-1: Screening Procedural Outline CA20976K (Applies to All Participants: Blinded or Open-label Treatment)

Table 2-2: On-Treatment Assessments CA20976K (Applies to All Participants: Blinded or Open-label Treatment)

Table 2-3: Long-Term Follow-Up CA20976K (Applies to All Participants: Blinded or Open-label Treatment)



Approved v1.0 930137673 4.0

**Table 2-1: Screening Procedural Outline CA20976K (Applies to All Participants: Blinded or Open-label Treatment)**

| **Procedure**[a] | **Screening Visit (Before Day 1, prior to Randomization)**[b] | **Notes** |
|---|---|---|
| **Eligibility Assessments** | | |
| Informed Consent | X | Must be obtained prior to performing any screening procedures.<br>Register in IRT to obtain the participant number (See Section 7.2)<br>If participant meets the criteria for re-enrollment (Section 6.4), the participant must be re-consented and assigned a new participant number from IRT.<br>**For optional open-label nivolumab treatment after first recurrence:**<br>Participant will maintain the same participant ID assigned for randomized blinded treatment, but the ID must be assigned again via IRT for open-label nivolumab treatment (See Section 7.2) |
| Inclusion/Exclusion Criteria | X | All inclusion/exclusion criteria (See Section 6.1 and 6.2) will be assessed during Screening (and reenrollment if applicable), and must be confirmed prior to randomization.[b] |
| Medical History | X | All medical history related to the disease under study.<br>• Include: Smoking history (including e-cigarettes), and alcohol history. |
| Concomitant Medication Collection | X | Within 14 days prior to randomization[b] |
| Review of Pathology Report | X | The pathology report must be reviewed, and the review documented by the investigator prior to randomization.[b]<br>Copies of de-identified pathology reports must be sent to the central laboratory vendor prior to randomization.[b]<br>• Refer to inclusion criteria in Section 6.1 and 6.2. |
| Tumor Tissue Samples | X | Prior to randomization[b]: Tumor tissue from the primary diagnostic biopsy (minimum 15 unstained slides, preferably freshly cut, or 1 |

**Table 2-1: Screening Procedural Outline CA20976K (Applies to All Participants: Blinded or Open-label Treatment)**

| **Procedure**[a] | **Screening Visit (Before Day 1, prior to Randomization)**[b] | **Notes** |
|---|---|---|
| | | FFPE block to contain sufficient tissue for at least 15 sections) must be shipped to central laboratory. Refer to Section 6.1 (Inclusion Criteria) and Section 9.8.2 (Tissue Specimens). |
| **Safety Assessments** | | |
| Complete Physical Examination | X | Include height and weight, within 14 days prior to randomization.[b] Pay special attention to skin and nodal examination. |
| Vital Signs, Oxygen Saturation | X | Include blood pressure (BP), heart rate, and temperature. Oxygen saturation by pulse oximetry at screening only. Perform assessments at the screening visit within 14 days prior to randomization.[b] |
| Performance Status (ECOG) | X | Within 14 days prior to randomization[b] |
| Serious Adverse Event Assessment | X | Collected from the time of consent, including all SAEs associated with SARS-CoV-2 infection. |
| Assessment of Signs and Symptoms | X | Within 14 days prior to randomization[b] |
| Electrocardiogram (ECG) | X | Within 14 days prior to randomization[b] |
| Laboratory Tests | X | On site/local laboratory testing to be completed within 14 days prior to randomization,[b] except for viral testing, which is to be completed within 28 days prior to randomization.[b] See Section 9.4.4 for a list of laboratory tests to be conducted. |
| Pregnancy Test | X | WOCBP only. Serum or urine to be done at screening visit and repeated within 24 hours prior to first dose of study therapy. For females under the age of 55, FSH > 40 mIU/mL is required to confirm menopause. See Appendix 4. |



Approved v4.0 930137673 4.0

**Table 2-1: Screening Procedural Outline CA20976K (Applies to All Participants: Blinded or Open-label Treatment)**

| Procedure[a] | Screening Visit (Before Day 1, prior to Randomization)[b] | Notes |
|---|---|---|
| **Efficacy assessment** | | |
| Body Imaging | X | Within 4 weeks (28 days) prior to randomization:[b] Contrast-enhanced (CE) CT of the chest, CE CT/MRI of the abdomen/pelvis, and all other known and/or suspected sites of disease (imaging of extremities for resected melanomas located in the extremities is not a requirement and may be conducted per local standard of care). See Section 9.1.1.1 for further details. |
| Brain Imaging | X | Within 4 weeks (28 days) prior to randomization[b]: MRI of the brain (brain CT allowable if MRI is contraindicated) without and with contrast, to be performed only if participant has signs and symptoms consistent with metastases to the brain. See Section 9.1.1.1 for further details. |
| **Other** | | |
| [redacted] | | |
| IRT Call/Assignment | X | |

Abbreviations: CT, computed tomography; eCRF, electronic Case Report Form; FFPE, formalin-fixed paraffin embedded; FSH, follicle-stimulating hormone; ID, [redacted] IRT, Interactive Response Technology; MRI, magnetic resonance imaging; SAE, serious adverse events; SAR-CoV-2, severe acute respiratory syndrome coronoavirus 2; WOCBP, women of childbearing potential.

[a] Some of the assessments referred to in this section may not be captured as data in the eCRF. They are intended to be used as safety monitoring by the treating physician. Additional testing or assessments may be performed as clinically necessary or where required by institutional or local regulations.

[b] For participants who opt for open-label on-protocol nivolumab treatment after first disease recurrence, Screening assessments will occur prior to assignment to optional on-protocol open-label nivolumab treatment.

**Table 2-2: On-Treatment Assessments CA20976K (Applies to All Participants: Blinded or Open-label Treatment)**

| Procedure[a,b] | Each cycle = 4 weeks)[c] | Notes |
|---|---|---|
| | **Day 1 of Each Cycle** | Cycle 1 is to be initiated within 3 calendar days (72 hours) of randomization.[d]<br>Subsequent cycles can be initiated within ± 3 days of the target visit date. See Section 7 (Treatment) for further details. |
| **<u>Safety Assessments</u>** | | |
| Targeted Physical Examination | X | To be performed within 72 hours prior to dosing. Pay special attention to skin and nodal examination. |
| Vital Signs | X | Include BP, heart rate, temperature. |
| Weight and Performance Status | X | Within 72 hours prior to dosing. |
| Adverse Event Assessment (including SAE assessment) | X | Continuously during treatment (including AEs/SAEs associated with SARS-CoV-2 infection). All AEs/SAEs are to be graded and managed per NCI CTCAE v5. |
| Review of concomitant medications | X | |
| Laboratory Tests | X | See full list of assessments in Section 9.4.4.<br>On site/local labs to be performed within 72 hours prior to dosing.<br>Note: Cycle 1 Day 1 laboratory tests are not required to be repeated if performed within 14 days prior to first dose.<br>Amylase, lipase, albumin, ACTH, and cortisol as clinically indicated. |
| Pregnancy Test | X | A negative pregnancy test should be documented within 24 hours prior to the initial administration of study drug. For subsequent cycles, a negative pregnancy test should be documented within 72 hours prior to dosing with study drug. Serum or Urine. |
| [redacted] | | |

**Table 2-2: On-Treatment Assessments CA20976K (Applies to All Participants: Blinded or Open-label Treatment)**

| **Procedure**[a,b] | **Each cycle = 4 weeks)**[c] | **Notes** |
|---|---|---|
| [redacted] | | |
| **Efficacy Assessments** | | |
| Body Imaging | See note | Contrast-enhanced (CE) CT of the chest, CE CT/MRI of the abdomen/pelvis, and all other known and/or suspected sites of disease. Imaging of extremities for resected melanomas located in the extremities is not a requirement and may be conducted per local standard of care. Extremity imaging should be conducted in the event of a suspected locoregional relapse that is not clinically unequivocal.<br><br>Participants with Stage IIB/C melanoma (Nivolumab or Placebo blinded portion):<br>• Every 26 weeks (± 14 days) during the treatment phase of 12 months.[e]<br>For optional open-label nivolumab treatment after first recurrence:<br>ARM 1 (Resectable disease)<br>Participants enrolled with recurrent Stage III/IV resected disease<br>• Every 12 weeks (± 7 days) from first dose of open-label study treatment through 12 months.[e]<br>ARM 2 (Unresectable/metastatic disease)<br>• Every 12 weeks (± 7 days) from first dose of open-label study treatment through 24 months.[e] |
| Brain Imaging | See note | Participants with signs or symptoms consistent with brain metastases should have an MRI of the brain (without and with contrast) as clinically indicated. CT of the brain without and with contrast can be performed if MRI is contraindicated. See Section 9.1.1.1 for further details. |
| **Pharmacokinetic /Immunogenicity Assessments** | | |
| PK and Immunogenicity samples | See note | Details regarding specific sample timing are specified in Table 9.5-1. |
| [redacted] | | |



Approved v4.0 930137673 4.0

**Table 2-2: On-Treatment Assessments CA20976K (Applies to All Participants: Blinded or Open-label Treatment)**

| Procedure[a,b] | Each cycle = 4 weeks)[c] | Notes |
|---|---|---|
| [redacted] | | |
| Biopsy of affected organs | See note | Optional. Upon occurrence of ≥ Grade 3 drug-related AE and/or lab abnormality regarded as a drug-related SAE. (See Section 9.8.2 and Table 2-3 for additional information). |
| **Clinical Drug Supplies** | | |
| IRT Randomization | X | Details regarding randomization of participants to receive nivolumab or placebo are described in Section 7.2.1.<br>For details on treatment assignment for participants in the open-label nivolumab monotherapy portion of the trial, see Section 7.2.2. |
| Dispense Study Treatment | X | Please use the IRT to dispense study treatment. First dose of study treatment to be administered within 3 calendar days (72 hours) of randomization.[d] Refer to Section 7.1.1 for details. |

Abbreviations: ACTH, adrenocorticotropic hormone; AE, adverse events, BP, blood pressure, CE, contrast enhanced; CT, computed tomography; eCRF, electronic Case Report Form; [redacted] FFPE, formalin-fixed paraffin embedded; FSH, follicle-stimulating hormone; [redacted] IRT, Interactive Response Technology; MRI, magnetic resonance imaging; NCI CTCAE v5, National Cancer Institute Common Terminology Criteria for Adverse Events version 5; PK, pharmacokinetics; SAE, serious adverse event; SARS-CoV-2, severe acute respiratory syndrome coronavirus 2; WOCBP, Women of childbearing potential.

a Some of the assessments referred to in this section may not be captured as data in the eCRF. They are intended to be used as safety monitoring by the treating physician. Additional testing or assessments may be performed as clinically necessary or where required by institutional or local regulations.

b If a dose is delayed, the procedures (except imaging procedures) scheduled for that same time point should also be delayed to coincide with when that time point's dosing actually occurs.

c Participants receiving blinded study treatment, and those receiving open-label nivolumab in Arm 1 (resectable disease) will be treated until unacceptable toxicity, withdrawal of consent, completion of 12 months of treatment, disease recurrence, or the study ends, whichever occurs first. Participants receiving open-label nivolumab after first recurrence in Arm 2 (unresectable/metastatic disease) will be treated until unacceptable toxicity, withdrawal of consent, completion of 24 months of treatment, disease recurrence, or the study ends, whichever occurs first.

d Prior to assignment to open-label nivolumab for participants who opt for open-label on-protocol nivolumab treatment after first disease recurrence.

e Timing is calculated from the first dose of blinded study treatment or open-label treatment, whichever is applicable.



Approved v4.0 930137673 4.0

**Table 2-3: Long-Term Follow-Up CA20976K (Applies to All Participants: Blinded or Open-label Treatment)**

<table>
<tr><th>Procedure</th><th>Follow-up Visit 1[a]</th><th>Follow-up Visit 2[a]</th><th>After Follow-up Visit 2</th><th>Notes</th></tr>
<tr><td colspan="5"><u>Safety Assessments</u></td></tr>
<tr><td>Targeted Physical Examination, Vital Signs, and Performance Status</td><td>X</td><td>X</td><td>X (see Note)</td><td><u>Only for participants with Stage IIB/C melanoma (Nivolumab or Placebo blinded portion):</u><br>After Follow-up Visit 2 (FU2), participants continue in-person assessment approximately every 12 weeks for 12 months (12 months calculated from FU2). Pay special attention to skin and nodal examination.<br>For participants initiating subsequent systemic anti-cancer therapy for a melanoma recurrence, other than participation in optional open-label nivolumab monotherapy or for a non-melanoma cancer, follow-up assessments can be done per local standard of care.</td></tr>
<tr><td>Adverse Event Assessment</td><td>X</td><td>X</td><td rowspan="2">X (see Note)</td><td rowspan="2">Please refer to Section 9.2 and Appendix 3 for further details regarding the collection and follow-up of AEs/SAEs (including AEs/SAEs associated with SARS-CoV-2-related infection) following study treatment discontinuation.<br><br>All AEs/SAEs are to be graded and managed per NCI CTCAE v5.<br><br>Only for participants with Stage IIB/C melanoma (Nivolumab or Placebo-blinded portion):<br><u>After FU2</u>, participants continue in-person assessment approximately every 12 weeks for an additional 12 months (12 months calculated from FU2) [b].</td></tr>
<tr><td>SAEs</td><td>X</td><td>X</td></tr>
<tr><td>Laboratory Tests</td><td>X</td><td>X</td><td></td><td>For Follow-up Visit 1 (FU1), onsite/local labs to include hematology and chemistry testing. See Section 9.4.4 for panel requirements. Repeat at Follow-up Visit 2 if study</td></tr>
</table>



Approved v4.0 930137673 4.0

**Table 2-3: Long-Term Follow-Up CA20976K (Applies to All Participants: Blinded or Open-label Treatment)**

| Procedure | Follow-up Visit 1[a] | Follow-up Visit 2[a] | After Follow-up Visit 2 | Notes |
|---|---|---|---|---|
| | | | | treatment-related toxicity persists, or as clinically indicated.<br>Laboratory toxicities (eg, suspected drug induced liver enzyme elevations) will be monitored during the follow-up phase, based on results from on-site/local labs, until all study drug related toxicities resolve, return to baseline, or are deemed irreversible. |
| Pregnancy Test | X | X | | Serum or urine.[b] Pregnancy testing is only required at FU1 and FU2, unless increased frequency and duration is required per local regulations. |
| Review of Concomitant Medication | X | X | | Long-term use of corticosteroids and other immune-modulating medications will be reported.[b] |
| [redacted] | | | | |



Approved v4.0 930137673 4.0

**Table 2-3: Long-Term Follow-Up CA20976K (Applies to All Participants: Blinded or Open-label Treatment)**

| Procedure | Follow-up Visit 1[a] | Follow-up Visit 2[a] | After Follow-up Visit 2 | Notes |
|---|---|---|---|---|
| **Efficacy Assessments** | | | | |
| Body Imaging | See note | | | Contrast-enhanced (CE) CT of the chest, CE CT/MRI of the abdomen/pelvis, and all other known and/or suspected sites of disease. Imaging of extremities for resected melanomas located in the extremities is not a requirement and may be conducted per local standard of care. Extremity imaging should be conducted in the event of a suspected locoregional relapse that is not clinically unequivocal.<br>**For participants with Stage IIB/C melanoma (Nivo or Placebo blinded portion):**<br>• Years 1, 2, and 3: Every 26 weeks (± 14 days)<br>• Years 4 and 5: Every 52 weeks (± 28 days)<br>• Participants who develop a loco-regional recurrence only, must be followed by surveillance imaging until the development of distant metastases.<br>• If a participant starts systemic anti-cancer therapy for melanoma recurrence after study drug discontinuation, other than optional open-label on-protocol nivolumab systemic therapy, or for a new non-melanoma tumor, follow-up scans can be done as per local standard of care.<br>**For optional open-label nivolumab treatment after first recurrence:**<br>**ARM 1 (Resectable Disease)[d]**<br>***Participants enrolled with recurrent Stage III/IV resected disease***<br>• Years 1, 2, and 3: Every 12 weeks (± 7 days)<br>• Years 4 and 5: Every 26 weeks (± 14 days)[c] |



Approved v4.0 930137673 4.0

**Table 2-3: Long-Term Follow-Up CA20976K (Applies to All Participants: Blinded or Open-label Treatment)**

| Procedure | Follow-up Visit 1[a] | Follow-up Visit 2[a] | After Follow-up Visit 2 | Notes |
|---|---|---|---|---|
| | | | | **ARM 2 (Unresectable/Metastatic Disease)**[e] Every 12 weeks (± 7 days) until disease progression, or treatment discontinuation, whichever occurs later. |
| Brain Imaging | MRI (brain CT allowable if MRI is contraindicated) of the brain without and with contrast, as clinically indicated. See Section 9.1.1. | | | |
| **Survival status** | | | | |
| Survival status | X | X | X | Every 12 weeks (± 14 days) from FU2 until the OS final analysis.[e] May be accomplished by visit or phone contact, to include subsequent anti-cancer therapy. BMS may request that survival data be collected on all treated participants outside of the 12-week window. At the time of this request each participant will be contacted to determine their survival status unless the participant has withdrawn consent for all contact. |
| **Pharmacokinetic and Immunogenicity Samples** | | | | |
| PK and Immunogenicity | Details regarding specific sample timing are specified in Table 9.5-1. | | | |

[redacted]



Approved v4.0 930137673 4.0

**Table 2-3: Long-Term Follow-Up CA20976K (Applies to All Participants: Blinded or Open-label Treatment)**

| Procedure | Follow-up Visit 1[a] | Follow-up Visit 2[a] | After Follow-up Visit 2 | Notes |
|---|---|---|---|---|
| Label Arm 1: resectable disease)/progression (Open-Label Arm 2: unresectable/metastatic disease) | | | | recurrence/progression (either surgery or biopsy) is strongly recommended for all participants except when risk of procedure to obtain such material is too high or not feasible. Refer to Section 9.8.2 [redacted] |
| Biopsy of affected organs | Upon occurrence of ≥ Grade 3 drug-related AE and/or lab abnormalities regarded as a drug- related SAE | | | Optional |

[redacted]

Abbreviations: AE, adverse event; BMS, Bristol-Myers Squibb; CR, complete response ; CT, computed tomography; [redacted] FU1, Follow-Up Visit 1; FU2, Follow-Up Visit 2; MRI, magnetic resonance imaging; NCI CTCAE v5, National Cancer Institute Common Terminology Criteria for Adverse Events version 5; OS, overall survival; PK, pharmacokinetics; PR, partial response; SAE, serious adverse event; [redacted]; SD, stable disease.

[a] Participants must be followed for at least 100 days after the last dose of study treatment. FU1 should occur 30 days from the last dose (± 7 days) or can be performed on the date of discontinuation if that date is greater than 37 days from the last dose. FU2 occurs approximately 100 days (± 7 days) from the last dose of study treatment. Both follow-up visits should be conducted in person.

[b] If a participant on the placebo arm experiences recurrence, and opts to receive open-label nivolumab monotherapy (Arm 1 or 2), participants may or may not have completed the two follow-up visits (FU1 and FU2) on blinded treatment. Assessments from FU1 and FU2, if conducted, may contribute to satisfying screening/Cycle 1 Day 1 requirements prior to treatment assignment (please check Screening Schedule of Activities requirements). If the participant (nivolumab- or placebo-treated) does not opt to receive open-label nivolumab treatment, the assessments/assessment frequency described in this table apply. **For optional open-label nivolumab treatment after first recurrence, visits after FU2, may be accomplished by visit (eg, examination, laboratory tests) OR by phone contact.**

[c] Timing is calculated from the date of the first dose of blinded study treatment, or open-label nivolumab treatment, whichever is applicable. [redacted]

[d] **Efficacy Assessments (Arm 1: Resectable disease):** If a participant starts systemic therapy for melanoma recurrence or for a new non-melanoma cancer after study drug discontinuation, follow-up scans can be done as per standard of care. Participants who did not have Stage IV resected disease on entry in the open-label treatment portion of the trial who develop a loco-regional recurrence only and have not initiated subsequent anticancer systemic therapy, must be followed by surveillance imaging until the development of distant metastases.

[e] **Efficacy Assessments (Arm 2: Unresectable/Metastatic disease):** For responders (CR, PR or SD) who received open-label nivolumab and remain on study beyond 2 years, an extension of the frequency of scans from every 12 weeks to every 26 weeks (± 14 days) until disease progression is permitted. Participants who discontinue study therapy for reasons other than progression should continue to be scanned until progression is documented. *Note: All imaging activities will continue until Year 5 (Year 5 is calculated from first dose of open-label on-protocol nivolumab study treatment).*



Approved v4.0 930137673 4.0

# 3 INTRODUCTION

## 3.1 Study Rationale

### *3.1.1 Overview*

CA20976K is a Phase 3, randomized, double-blind study designed to evaluate the use of adjuvant immunotherapy with nivolumab versus placebo after complete resection of Stage IIB/C melanoma in adults and pediatric participants ≥ 12 years old.

In this study, participants with resected Stage IIB/C melanoma and no evidence of disease will be randomized to treatment with either nivolumab or placebo for a duration of 12 months. Results from this study will help to define the role of nivolumab as an adjuvant immunotherapy in patients with resected Stage IIB and IIC cutaneous melanoma. In the event of disease recurrence, participants will have the option to receive on-study open-label nivolumab treatment. Placebo-treated participants who experience disease recurrence within 3 years after the last dose of placebo, and nivolumab-treated participants who experience recurrence greater than 6 months and within 3 years after completing treatment, will be eligible to participate in on study unblinded /open-label nivolumab treatment. Participants with resectable / recurrent disease will be offered nivolumab for a maximum duration of 12 months, whereas participants with unresectable or metastatic disease will be offered nivolumab monotherapy for a maximum of 24 months. Participants with disease recurrence who do not participate in the optional open-label nivolumab treatment portion of the study will be treated per standard of care as recommended by their treating physician.

### *3.1.2 Research Hypothesis*

Treatment with systemically administered nivolumab when compared to placebo will result in greater efficacy as measured by recurrence-free survival (RFS) and overall survival (OS) in participants with completely resected Stage IIB/C melanoma with no evidence of disease (NED).

## 3.2 Background

Melanoma accounts for less than 5% of all skin cancers; however, it causes the greatest number of skin cancer–related deaths worldwide.[1] Because earlier stage disease is prevalent, most deaths from melanoma occur in individuals who are initially diagnosed with low-thickness melanoma and not with advanced-stage disease, underscoring the importance of intervention in the early adjuvant (Stage I/II) setting.[2] Despite patients with early stage disease contributing the largest population to melanoma-specific mortality under the current treatment guidelines, these patients represent a population less likely to be treated in the early adjuvant setting.

In the United States, it is estimated that approximately 11,000 patients will be diagnosed with Stage II melanoma in 2019 half of whom will be diagnosed with Stage IIB/C melanoma. Worldwide (excluding the United States), approximately 44,000 patients will be diagnosed with Stage II disease in 2019, of which more than 50% are expected to be Stage IIB/C disease.[3] Moreover, unlike other solid tumors, melanoma mostly affects young and middle-aged individuals (median age at diagnosis, 57 years).[4] Between 1 to 4 percent of all melanomas occur in patients younger than 20 years of age.[5,6]



Approved v1.0 930137673 4.0

In contrast to patients who present with localized disease and primary tumors 1.0 mm or less, wherein a 5-year survival rate is achieved in more than 90% of patients, the 5-year survival rates for patients with localized melanomas more than 1.0 mm in thickness (T2-T4) range from 50% to 90% depending on tumor thickness, ulceration, and mitotic rate. The likelihood of regional nodal involvement upon relapse, increases with tumor thickness as well as the presence of ulceration and high mitotic rate.

Patients diagnosed with Stage IIB/C disease have thicker melanomas (> 2.0 mm). These patients have an unmet clinical need as there is no clear safe and effective systemic adjuvant therapy to prevent disease relapse once the primary tumor has been completely resected, which is the current standard of care for these patients. Approximately, 15% to 20% of patients diagnosed with Stage IIB and 30% of patients diagnosed with Stage IIC melanoma will have a relapse of their melanoma at 24 months.[7] Within 5 years of surgical resection, approximately 25% of patients with Stage IIB disease and 40% of patients with Stage IIC disease will have a relapse of their disease.[8] A SEER Registry review conducted by Landow et al. (2016) revealed that 7.4% (95% CI: 7.2-7.7) of patients diagnosed with invasive melanoma limited to the skin died within 10 years of diagnosis.[9] Relapse of disease portends a poor prognosis. Long-term survival in patients, once distant metastatic melanoma develops, has been less than 10%[9,10] or around 22% at 5-years from diagnosis.[8]

A greater understanding of the role of tumor burden as a modulating factor in the efficacy of anti-melanoma therapy is important. It is apparent that treatments for melanoma have greater odds of efficacy in patients with a smaller, more-limited disease burden. This has been observed with regional therapies (talimogene laherparepvic, isolated limb infusion or perfusion), targeted therapies (dabrafenib + trametinib, vemurafenib) and with immunotherapies (pembrolizumab, ipilimumab, nivolumab). Specifically, for anti-PD-1 immunotherapy, it has been reported that the ratio of T-cell invigoration to tumor burden may be associated with anti–PD-1 response.[11,12]

The introduction of therapies earlier in the course of the disease, when the tumor burden is much lower, to eradicate undetectable minimal residual disease prior to developing metastasis, therefore, has the potential for greatly increasing the chance of cure and substantially influencing melanoma-specific mortality. To extend the potential benefit of immunotherapy in the adjuvant setting to pediatric patients, pediatric patients diagnosed with Stage IIB/C melanoma ≥ 12 year old will also be included in this trial.

### 3.2.1 Pediatric Melanoma

Pediatric melanoma, a rare cancer, is usually defined as melanoma occurring in patients younger than 20 years, representing approximately 1 to 4 percent of all melanomas.[5,6] Melanoma is the second-most-common adult-type cancer in this age group (after thyroid cancer). Melanoma is rarer in younger children, with estimated annual incidence rates of one, two, three, and ten per million in the age groups 1 to 4, 5 to 9, 10 to 14, and 15 to 19, respectively.[8] The number of Stage IIB/C melanoma cases (at least one case) estimated in 2019 in patients less than 20 years of age by country, is displayed in the table below (Table 3.2.1-1).[13]



Approved v4.0 930137673 4.0

**Table 3.2.1-1: Estimated Number of Melanoma Cases in Patients Less than 20 years of Age by Country**

| Country | Estimated number of melanoma cases (males and females) in 2019 |
|---|---|
| Argentina | 4 |
| Australia | 4 |
| Belgium | 1 |
| Brazil | 41 |
| Canada | 1 |
| Chile | 2 |
| China - Rural | 74 |
| China - Urban | 2 |
| Colombia | 7 |
| Denmark | 1 |
| Egypt | 1 |
| France | 4 |
| Germany | 7 |
| Greece | 1 |
| India - Rural | 1 |
| Indonesia | 1 |
| Italy | 7 |
| Japan | 2 |
| Mexico | 7 |
| Netherlands | 2 |
| Nigeria | 7 |
| Portugal | 2 |
| Russia | 9 |
| South Africa | 1 |
| Spain | 6 |
| Sweden | 1 |
| Switzerland | 1 |
| Taiwan | 2 |
| Turkey | 5 |
| United Kingdom | 4 |
| United States | 16 |
| Venezuela | 3 |



Approved v4.0 930137673 4.0

Melanomas in children frequently are diagnosed as having thicker Breslow depths than in adults.[14] Children less than 10 years old are more likely to present with a higher stage disease, which may in part be due to delayed diagnosis of children with melanoma.[15] Similar to adult melanoma, tumor thickness, ulceration, lymph node status, and stage are found to be significant predictors of survival in patients with pediatric melanoma.[16] Despite the increasing incidence of melanoma in children and adolescents, there remains a paucity of research on this population. Currently, the management of pediatric and adolescent melanoma is based primarily on well-established practice guidelines used for adult patients. Additional studies are needed to evaluate outcomes as they relate to treatment and disease presentation in preadolescents and adolescents.[5]

### 3.2.2 The Adjuvant Treatment of Patients with Stage IIB/C Melanoma with Nivolumab is a Promising Strategy

At present, wide surgical excision of the primary melanoma with an offer to perform a sentinel lymph node biopsy is standard of care for patients with clinical Stage II melanoma of all substages. Patients who have a positive sentinel lymph node are upstaged to Stage III and can undergo either surveillance of the nodal basin with ultrasound or complete lymph node dissection. Per current guidelines, patients with node positive disease may be offered adjuvant nivolumab, pembrolizumab, or dabrafenib/trametinib (BRAF V600 activating mutation) or observation. Current treatment recommendations for patients with a negative sentinel lymph node (such as the CA20976K study population) or for patients in whom a sentinel lymph node biopsy was not conducted for any reason, is observation with periodic surveillance to detect disease recurrence, or treatment with high-dose interferon. For melanomas measuring more than 2 mm in thickness, which would include patients diagnosed with Stage IIB and IIC, the evaluation of the sentinel lymph node for disease involvement and a wide excision of the primary melanoma with 2-cm margins is highly recommended.[10,14]

There is an unmet need for adjuvant therapy in patients with earlier stage melanoma, such as Stage IIB and IIC. These patients do not have evidence of macroscopic or clinically detectable metastases but are at high risk of recurrence, which speaks to the presence of clinically occult metastases. Given the toxicity and limited efficacy of currently available adjuvant therapies such as high-dose interferon-alfa for early stage melanoma, most guidelines recommend observation for patients with Stage IIB and IIC melanoma who have had a wide excision of their melanomas and a negative sentinel lymph node biopsy.

The goal of adjuvant therapy is to cure patients with the lowest burden of disease (ie, micrometastatic disease after complete surgical resection). The mechanistic rationale for adjuvant therapy is that invasive and motile tumor cells enter circulation before the tumor is diagnosed. The disseminated tumor cells are not eradicated by the surgical removal of the primary tumor or the regional lymph nodes and may survive for years in a dormant state or as subclinical micrometastases in distant organs. After months or years of dormancy, some tumor cells may resume proliferation and form clinically detectable macrometastases.[17,18] The proven efficacy of immunotherapies (anti-CTLA-4 and anti-PD-1 agents) in the treatment of unresectable/metastatic melanoma and in the adjuvant Stage III melanoma setting, make such agents a natural choice to



Approved v4.0 930137673 4.0

study in patients diagnosed with Stage IIB/C melanoma, where the burden of disease is relatively low.

#### 3.2.2.1 Harnessing the Success of Immunotherapy in Patients with Late Stage Melanoma to Patients with Stage IIB/C Melanoma

As discussed above, the efficacy and acceptable safety profile of checkpoint-inhibitors in patients with more advanced melanoma make these agents a suitable choice to study in the earlier stage melanoma setting. The data obtained with these agents in later stage melanoma, is presented below and support the study of immunotherapies in Stage IIB/C melanoma. The efficacy and safety of checkpoint inhibitors and the experience with other types of systemic therapies is also briefly described. As therapies in the treatment of melanoma were first introduced in more advanced disease settings prior to being studied in earlier stage disease, the data will be presented accordingly, starting with the data from trials in unresectable/metastatic melanoma, followed by data from the Stage III and Stage II adjuvant melanoma setting. When available, the efficacy of these systemic therapies in relation to the tumor burden is also described. Collectively, these data form the basis for studying nivolumab in the earlier stages of melanoma on the current trial.

***Unresectable/Metastatic Melanoma***

An examination of immunotherapy and other trials for unresectable or metastatic melanoma points to the potential value of treating patients with a lower tumor burden. Until the approval of ipilimumab as an effective immunotherapy in 2011 by the United States Food and Drug Administration (US FDA), dacarbazine, an alkylating agent, was the standard of care due to the ineffectiveness of other single agents or combination therapies in improving the OS of patients.[10,19] Dacarbazine was approved by the FDA in 1974 after clinical trials reported a complete response rate in < 5% and 5-year survival in 2% to 6% of patients.

Since the earliest report in the 19th-century that cancer and the immune system were interlinked, based on the fact that tumors were frequently observed at sites of chronic inflammation, as well as the presence of immune cells in tumor tissues, efforts were made to harness immunotherapy as a promising treatment option for advanced melanoma. High-dose IL-2, a cytokine capable of expanding effector T-cells (Teffs) and regulatory T-cells (Tregs) was approved by the US FDA in 1998 to treat metastatic melanoma in first-line and second-line settings. Although response rates are modest (< 20%) with this agent, patients with a complete response (< 10%) tend to have extremely durable responses and high rates of long-term survival (> 5 years).[20,21,22] Although durable effects of immunotherapy were observed with IL-2 immunotherapy, high-dose IL-2 is associated with significant toxicities, and its use is restricted to institutions with medical staff experienced in the administration and management of these regimens.

To further bolster the immune response, studies combining chemotherapy and immunotherapy (biochemotherapy) were conducted on the basis that cytotoxic chemotherapy could stimulate the immune response via release of tumor antigens through chemotherapy induced cell lysis. Although a higher response rate and an improvement in PFS was noted with biochemotherapy over CVD alone, an improvement in OS was not observed[23] and biochemotherapy therapy was associated with severe toxicity.



Approved v4.0 930137673 4.0

The search for more efficacious and safer immunotherapy approaches was revolutionized by the discovery of inhibitors of the inhibitory immune checkpoint receptor, cytotoxic T-lymphocyte-associated protein 4 (CTLA-4). CTLA-4 blocks T-cell activation and induces immune tolerance. Ipilimumab, an anti-CTLA-4 antibody, blocks the inhibitory effect, enhancing pro-inflammatory T-cell cytokine production, and increasing clonal T-cell expansion and infiltration in responding tumors. Ipilimumab was approved by the US FDA for the treatment of advanced melanoma in 2011.[24] Results from 2 Phase 3 pivotal trials in Stage III/IV unresectable/metastatic melanoma supported the use of ipilimumab in previously treated (CA184002) and previously untreated (CA184024) metastatic melanoma. CA184002 randomly assigned patients to ipilimumab, gp100 peptide vaccine or ipilimumab plus gp100 peptide vaccine. CA184024 randomly assigned patients to dacarbazine plus ipilimumab or ipilimumab plus placebo. Results from these trials showed that ipilimumab was associated with improved response rates, response duration, PFS and OS,[25,26] with long-term survival in approximately 20% of patients on the ipilimumab arm.[27] High rates of Grade 3-4 AEs were observed in these trials, which utilized a higher dose of ipilimumab (10 mg/kg) and in combination with dacarbazine. Currently, ipilimumab is administered at 3 mg/kg in the unresectable/metastatic melanoma setting, and not in combination with dacarbazine.

The relationship of melanoma tumor burden to PFS and OS in an immunotherapy trial was demonstrated by Nishino et al (2014) in a Phase 1 trial of ipilimumab in combination with bevacizumab. The authors reported that patients with a median tumor diameter less than 38 mm had better median PFS and OS compared to patients with a median diameter of greater than 38 mm. (PFS of 27.5 months vs. 4.1 months; OS: Not reached vs. 12.6 months). The risk of progression and death increased by 14% and 18%, respectively, with every 5 mm increase in baseline diameter. The study underscores the importance of treating the disease when the tumor burden is much lower, which may be the case in earlier state diagnosis.[28]

Following the approval of ipilimumab, nivolumab, an anti-PD-1 monoclonal antibody, was approved by the United States FDA in 2014 for the treatment of patients with metastatic melanoma. The PD-1 receptor binds to PD-L1 and PD-L2, and acts as a T-cell co-inhibitory molecule, suppressing T-cell activation. Two Phase 3 clinical trials (CA209066 and CA209067) demonstrated the efficacy of nivolumab in previously untreated unresectable Stage III or IV melanoma. CA209066 was a randomized double-blind trial of nivolumab versus dacarbazine, where nivolumab demonstrated a significantly higher response rate (40% vs 14%), PFS (5.1 vs 2.2 months), and 1-year OS rate (73% vs. 42%).[29] CA209067 was a randomized double-blind trial where patients were assigned to nivolumab plus ipilimumab, nivolumab or ipilimumab. Response rates of 57%, 44% and 19% were observed in the nivolumab plus ipilimumab arm, nivolumab arm, and ipilimumab arm respectively. Median PFS was 11·5 months (95% CI 8.7−19.3) in the nivolumab plus ipilimumab group, 6.9 months (5.1−10.2) in the nivolumab group, and 2.9 months (2.8−3.2) in the ipilimumab group. At a minimum follow-up of 48 months, median OS was not reached (95% CI: 38.2−not reached) in the nivolumab plus ipilimumab group, 36·9 months (28·3−not reached) in the nivolumab group, and 19·9 months (16.9−24.6) in the ipilimumab group.[30,31] The potential benefit of treating with immunotherapy at a point that there is a lower



Approved v4.0 930137673 4.0

tumor burden is suggested by subgroup analyses of this trial demonstrating increasingly higher 3-year PFS as well as OS rates, with decreasing tumor burden (from > 97 mm to ≤ 31 mm), both in the nivolumab as well as nivolumab plus ipilimumab arms.[30]

Similarly, pembrolizumab monotherapy, another anti-PD-1 agent, has been shown to improve response and PFS compared to chemotherapy (KEYNOTE-002) or ipilimumab monotherapy (KEYNOTE-006) in unresectable Stage III or IV melanoma.[32,33] As noted with nivolumab, several authors have reported better efficacy outcomes with lower tumor burden with the use of pembrolizumab. Complete responses were noted in patients who had significantly smaller metastases (< 5 cm) or baseline sum of lesion diameters < 10.2 cm (median). Huang et al (2017) reported that the anti-PD-1 response may be associated with the ratio of T-cell invigoration to tumor burden.[11] The authors studied pre- and post-peripheral blood samples from 29 patients with Stage IV melanoma receiving pembrolizumab. The researchers noted that the ratio of exhausted CD8+ T cell phenotype to tumor burden distinguished clinical outcomes and predicted response. Therefore, the authors concluded that having T-cell invigoration was not adequate by itself; rather, it was the relative balance between the tumor burden and T-cell invigoration that contributed to response. This finding again suggests that better outcomes with anti-PD-1 agents may be achieved early during the course of the disease when the tumor burden is low.[11]

The growing interest in immunotherapy as a modality to treat metastatic melanoma became evident when the oncolytic virus, talimogene laherparepvic (T-VEC), a genetically modified nonpathogenic herpes simplex virus type 1 was approved by the FDA in 2015, in patients with refractory Stage IV or unresectable Stage III melanomas.[34] The virus after injection into a metastatic melanoma lesion, enters into both normal and malignant cells. The virus replicates in the melanoma cells, leading to tumor cell lysis and the release of tumor-specific antigens, which are recognized by antigen presenting cells, activating melanoma-specific T-cell responses.[19] In this study, 40% of patients had a complete response following T-VEC treatment, and patients with tumor burden less than the median (14.5 $cm^2$) had significantly improved durable and overall response rates, as well as improved OS.[35]

Other than immunotherapies, targeted therapies inhibiting either BRAF (intracellular signaling kinase in the MAPK pathway) or MEK (downstream target of BRAF) have been approved as monotherapies in unresectable/metastatic melanoma but will not be discussed in detail here. Interestingly, as seen above with the immunotherapies, disease burden was also seen to play an important role in predicting response to combination BRAF-MEK inhibition in advanced melanoma. A pooled analysis of Phase 3 dabrafenib plus trametinib trials conducted by Schadendorf et al. (2017) showed that lower tumor burden, as characterized by normal LDH levels, less than three metastatic organ sites, and a sum of lesion diameters less than 66 mm identified the best prognostic group of those receiving combination therapy, with a 3-year PFS rate of 42%.[36]

The durable responses seen with immunotherapies in the advanced melanoma setting led to the further investigation of these agents in the adjuvant melanoma setting. In addition, the observation of increased efficacy in subgroups with a lower tumor burden is particularly important in an



Approved v1.0 930137673 4.0

adjuvant setting, when tumor burden is at a microscopic level where immunotherapy may provide the greatest opportunity for cure.

***Adjuvant melanoma (Stage III/IV resected)***

A number of controlled trials with adjuvant cytotoxic chemotherapy in patients diagnosed with Stage II and III melanoma did not demonstrate any therapeutic advantage; therefore adjuvant systemic chemotherapy is not indicated in melanoma.[14,37] As a result, efforts have been focused on other adjuvant systemic treatment modalities such as immunotherapy with interferons (IFNs), targeted therapies, and checkpoint inhibitors, with most of the experience in the Stage III adjuvant setting. Systemic therapies that have had a notable influence in the Stage III adjuvant setting, and that could potentially be of value in earlier Stage IIB/C melanoma due to their established efficacy are briefly discussed below.

The earliest investigations with immunotherapies in adjuvant melanoma started with IFNs, cytokines secreted by leukocytes that play an important role in the immunomodulatory, antiangiogenic, antiproliferative, and antitumor activities. High-dose IFN $\alpha$-2b was approved by the US FDA and in Europe in 1996 as an adjuvant therapy for the treatment of resected Stage IIB/III melanoma based on results of the ECOG 1684, a trial that showed a benefit on RFS and OS.[38] Although the hazard ratio for RFS was maintained at a median follow-up of 12.6 years, the continued benefit with OS was not maintained.[39] Adjuvant PEG-IFN was approved by the US FDA in 2011 for the treatment of patients with resected Stage III melanoma (EORTC 18991) based on a favorable RFS (HR = 0.82, P = 0.01) seen at a median follow-up of 3.8 years.[40] The toxicity profile of high-dose IFN spurred the study of low to intermediate dose IFNs. While improvement in relapse-free survival was noted in a few prospective randomized trials, no improvement in OS was demonstrated. As such, high-dose IFN with a planned duration of up to a year or pegylated-interferon with a planned duration of up to 5 years, is an option for patients with completely resected Stage III disease. As very few patients with completely resected in-transit disease were included in these IFN trials, guidelines do allow for the use of IFN in this patient population, but with a weaker recommendation.[41]

Similar to research in the unresectable and metastatic melanoma setting, an attempt was made to improve upon the efficacy of immunotherapy by combining with chemotherapy in the adjuvant melanoma setting. The SWOG S0008 trial established the role of biochemotherapy in completely resected Stage III melanoma except for Stage IIIA-N1a (non-ulcerated primary tumor with micrometastases in one sentinel lymph node).[42] An improved median RFS of 4.0 years was noted in the biochemotherapy arm (cisplatin, vinblastine and dacarbazine, plus interleukin-2 and interferon) versus 1.9 years in the high-dose interferon-alfa-2b arm after a median follow-up of 7.2 years. There was no significant difference in the median OS as well as the 5-year OS rate between the 2 groups.

Based on the efficacy of ipilimumab in metastatic melanoma, a Phase 3 double-blind randomized multicenter trial, EORTC 18071 (CA209029) was designed. The trial compared adjuvant high-dose ipilimumab (10 mg/kg) to placebo in patients with completely resected Stage III melanoma



Approved v4.0 930137673 4.0

(Stage IIIA disease [if N1a, at least one metastases > 1 mm] or with Stage IIIB-C disease but no in-transit metastases). The ipilimumab arm demonstrated an improvement in RFS (HR = 0.75, P = 0.013) versus placebo at a median follow-up of 2.74 years, resulting in approval by the US FDA in 2015.[43] The benefit in RFS was maintained at a median follow-up of 5.3 years (HR = 0.76, P < 0.001). In addition, a benefit in OS was also observed (HR = 0.72, P = 0.001).[44] As a result, ipilimumab became a standard therapy in Stage III adjuvant melanoma.

Following the success of nivolumab in metastatic melanoma, and on the basis of a more favorable safety profile than ipilimumab, nivolumab 3 mg/kg every 2 weeks for 1 year was studied in a Phase 3 randomized, double-blind, multicenter international trial (CA209238) in completely resected Stage IIIB/C and Stage IV participants. Ipilimumab, the active comparator was dosed at 10 mg/kg every 3 weeks for four doses, followed by dosing every 12 weeks, for a maximum of one year. With a minimum follow-up of 18 months, nivolumab demonstrated a statistically significant and clinically meaningful improvement in RFS (HR = 0.65, P < 0.001) and distant metastases-free survival (DMFS) (HR = 0.73) versus ipilimumab; the observed separation of the RFS and DMFS curves was maintained over 24 months.[45,46] Notably, the activity of nivolumab was observed regardless of BRAF mutation status. Nivolumab received US FDA and European Medicines Agency approval in December 2017 and July 2018, respectively, for resected Stage III and IV melanoma. Similarly, a Phase 3 trial (KEYNOTE -054) in Stage III resected patients was conducted with pembrolizumab 200 mg every 3 weeks for a maximum of one year versus placebo. The active therapy arm was associated with significantly longer RFS.[47] As with nivolumab, the benefit of treatment with pembrolizumab was observed regardless of BRAF mutation status. The safety profile of the anti-PD-1 agents was generally consistent with the safety profile observed with these agents in the metastatic setting. Due to the superior efficacy and favorable adverse event profile of nivolumab and pembrolizumab, these agents have become the preferred immunotherapy in Stage III completely resected patients.[41]

In addition to immunotherapies, modern combination targeted therapies such as BRAF-MEK inhibitors have also been evaluated as adjuvant treatments in Stage III melanoma on the basis of their efficacy in the advanced melanoma setting. The combination of dabrafenib plus trametinib was approved by the US FDA and the European Commission as adjuvant therapy for patients with completely resected BRAF V600E/K–mutant Stage III melanoma based on the RFS benefit to this combination seen in the Phase 3 COMBI-AD trial. Subgroup analysis of RFS demonstrated similar treatment benefit regardless of baseline factors, including disease stage, nodal metastatic burden, and ulceration.[48]

From the above discussion, the concept of moving immunotherapies from a more advanced setting where their efficacy has been well established, into a setting of occult or microscopic residual disease is well supported by the documented safety and efficacy data of these agents in the adjuvant setting. The efficacy of anti-PD-1 agents in the adjuvant setting was demonstrated independent of BRAF mutation status. These data support the study of checkpoint inhibitors in Stage IIB/C melanomas, where there exists compelling unmet clinical need.



Approved v1.0 930137673 4.0

***Adjuvant Melanoma (Stage II)***

As discussed earlier, for patients presenting with Stage IIB/C melanoma, the general recommendation, globally, is to perform a wide local excision with a 2 cm margin and offer a sentinel lymph node biopsy to detect regional lymph node involvement, followed by observation for recurrence of melanoma. Adjuvant therapies were generally not recommended for these participants, other than immunotherapies with interferons. Stage IIB/C melanomas are considered high-risk melanomas due to their relatively higher tumor thicknesses (2 mm to > 4 mm), and the presence of ulceration, all recognized high-risk features. Systemic therapies studied in the adjuvant Stage II setting will be briefly described below.

In recognition of the risk of recurrence, the NCCN 2018 guidelines (but not the 2019 guidelines) included the option of offering high-dose interferon-alfa (IFN-α), a category 2B recommendation, to patients diagnosed with Stage IIB/C melanoma.[10] In addition, adjuvant IFN is offered in some European countries for high-risk resected Stage II or III melanoma on the basis of reduction in RFS, but has not been universally adopted because of the small OS benefit and the significant toxicity.[14] A RFS benefit to INF-α was seen in ECOG 1690, a trial that compared high and low-dose adjuvant INF-α in patients diagnosed with Stage IIB/III melanoma.[49] The RFS benefit [hazard ratio (HR) = 0.75, P = 0.035] and a trend towards improved OS (HR = 0.72, P = 0.059) seen in a French trial conducted in patients with Stage II melanomas thicker than 1.5 mm, and clinically undetectable node metastases, formed the basis for approval of low-dose INF-α in Europe.[50] Although efficacy in RFS, and in some cases OS, has been observed with INF-α, the toxicity profile is substantial, especially, at high doses, greatly limiting its use in this early stage patient population.[42,47,51,52]

Based on the success of BRAF-targeted therapy in more advanced settings, BRAF-targeted therapy has been studied in fully resected Stage IIC BRAF-mutated melanoma. The BRIM-8 trial was a Phase 3, double-blind, randomized, placebo controlled study that compared adjuvant vemurafenib monotherapy versus placebo in resected Stage IIC patients, pooled with patients with resected Stage III disease, designated as cohort 1.[53] A separate cohort of Stage IIIC patients, designated as cohort 2 was also enrolled. In cohort 1 (patients with Stage IIC–IIIA–IIIB disease), after a median follow-up of 30.8 months, median disease-free survival was not reached (95% CI not estimable) in the vemurafenib group versus 36·9 months (21.4–not estimable) in the placebo group (HR 0.54 [95% CI 0.37–0.78]; log-rank P = 0·0010); however, the result was not significant because of the pre-specified hierarchical prerequisite for the primary disease-free survival analysis of cohort 2 to show a significant disease-free survival benefit.

In addition to the clinical benefits gained by curing localized melanoma, curing patients early of melanoma may also be very desirable to decrease direct melanoma-related costs, which tend to be higher as the disease advances. Buja et al (2018) built a highly detailed decision-making model to estimate the direct melanoma-related costs by disease stage and by phase of diagnosis and treatment according to clinical guidelines. The mean per-patient cost of the whole melanoma pathway (including one year of follow-up) ranged from €149 for stage 0 disease to €66,950 for Stage IV disease. The cost for Stage IIB and IIC participants was estimated to be €5,009 and



Approved v4.0 930137673 4.0

€5,989 respectively. This cost doubles to €10,210 once a patient develops Stage III disease, and increases almost 12 times to €66,950 for a patient who develops Stage IV disease.[54] Given the fact that durable responses have been observed in more advanced settings with anti-CTLA-4 and anti-PD-1 immunotherapies, successful treatment of patients in Stage IIB/C melanoma may also translate to a positive socioeconomic impact.

Due to the success of checkpoint-inhibitors in metastatic melanomas as well as in Stage III adjuvant melanomas, these agents are now being evaluated in the treatment of patients with completely resected Stage IIB/C melanoma. Pembrolizumab, 200 mg every 3 weeks, for a maximum of 1 year of therapy is being evaluated in patients with completely resected Stage IIB/C melanoma who have a negative sentinel lymph node biopsy, in a double-blind, randomized, international trial (KEYNOTE -716, NCT03553836). As observation is the generally accepted standard of care in this setting, the comparator arm is a placebo, and patients are randomized in a 1:1 ratio. A single arm Phase 2 trial in the same study population, using nivolumab, is also currently enrolling (NCT03405155). Other clinical trials with immunotherapies such as peptide vaccines (NCT03617328) are also being conducted in earlier phases that include resected Stage II participants, underscoring the belief that immunotherapies could be more effective when introduced earlier in the course of the disease.

In conclusion, there is an unmet need for effective adjuvant therapies in adult and pediatric patients with Stage IIB/C melanoma. The robust clinical activity of adjuvant nivolumab in participants with more advanced melanoma and the potential for increased benefit in patients with lower burden of disease warrant the further investigation of nivolumab in this population. We hypothesize that adjuvant treatment with nivolumab has the potential to significantly impact clinical outcomes such as RFS, DMFS, and OS in patients diagnosed with Stage IIB/C melanoma.

### 3.2.3 Nivolumab Mechanism of Action

Cancer immunotherapy rests on the premise that tumors can be recognized as foreign rather than as self and can be effectively attacked by an activated immune system. An effective immune response in this setting is thought to rely on immune surveillance of tumor antigens expressed on cancer cells that ultimately results in an adaptive immune response and cancer cell death. Meanwhile, tumor progression may depend on acquisition of traits that allow cancer cells to evade immunosurveillance and escape effective innate and adaptive immune responses.[45,46,55] Current immunotherapy efforts attempt to break the apparent tolerance of the immune system to tumor cells and antigens by either introducing cancer antigens by therapeutic vaccination or by modulating regulatory checkpoints of the immune system. T-cell stimulation is a complex process involving the integration of numerous positive as well as negative co-stimulatory signals in addition to antigen recognition by the T-cell receptor (TCR).[56] Collectively, these signals govern the balance between T-cell activation and tolerance.

PD-1 is a member of the CD28 family of T-cell co-stimulatory receptors that also includes CD28, CTLA-4, ICOS, and BTLA.[57] PD-1 signaling has been shown to inhibit CD-28-mediated upregulation of IL-2, IL-10, IL-13, interferon-γ (IFN-γ) and Bcl-xL. PD-1 expression also been noted to inhibit T-cell activation, and expansion of previously activated cells. Evidence for a



Approved v4.0 930137673 4.0

negative regulatory role of PD-1 comes from studies of PD-1 deficient mice, which develop a variety of autoimmune phenotypes.[58] These results suggest that PD-1 blockade has the potential to activate anti-self T-cell responses, but these responses are variable and dependent upon various host genetic factors. Thus, PD-1 deficiency or inhibition is not accompanied by a universal loss of tolerance to self-antigens.

In vitro, nivolumab binds to PD-1 with high affinity (EC50 0.39-2.62 nM), and inhibits the binding of PD-1 to its ligands PD-L1 and PD-L2 (IC50 ± 1 nM). Nivolumab binds specifically to PD-1 and not to related members of the CD28 family such as CD28, ICOS, CTLA-4 and BTLA. Blockade of the PD-1 pathway by nivolumab results in a reproducible enhancement of both proliferation and IFN-γ release in the mixed lymphocyte reaction (MLR). Using a cytomegalovirus (CMV) re stimulation assay with human peripheral blood mononuclear cells (PBMC), the effect of nivolumab on antigen specific recall response indicates that nivolumab augmented IFN-γ secretion from CMV specific memory T-cells in a dose-dependent manner versus isotype-matched control. In vivo blockade of PD-1 by a murine analog of nivolumab enhances the anti-tumor immune response and results in tumor rejection in several immunocompetent mouse tumor models (MC38, SA1/N, and PAN02).[59]

## 3.3 Benefit/Risk Assessment

Stage IIB/C, localized, resected melanoma represents a population of high unmet need due to the potential for locoregional, nodal or systemic relapse, which dramatically impacts post-recurrence survival, especially in the case of systemic relapses. The 5-year survival of patients dramatically drops from 98.4% for localized disease to 63.6% for disease that has spread regionally, and to 22.5% for distant metastatic disease.[8] The current recommended standard of care for patients diagnosed with Stage IIB/C melanoma is observation. Even though interferon-alfa, which has been shown to improve disease-free survival and OS in a small subset of patients, and could be used as an adjuvant treatment in Stage IIC patients, the toxicity profile precludes most physicians from using the agent as adjuvant treatment in this patient population. In fact, the most recent version of the NCCN guidelines (Jan 2019) does not recommend interferon-alfa in resected Stage IIB and IIC patients. [41]

Anti-PD1 antibodies such as nivolumab and pembrolizumab have demonstrated activity in the metastatic setting, as well as in resected Stage III/IV (nivolumab) and Stage III (pembrolizumab) melanoma. Nivolumab has been shown to have an acceptable safety profile, described in detail in the current Investigator Brochure (IB) for nivolumab. A pattern of immune-related AEs has been defined, for which management algorithms have been developed; these are provided in Appendix 5. Most high-grade events were manageable with the use of corticosteroids or hormone replacement therapy (endocrinopathies) as instructed in these algorithms. Additional details on the safety profile of nivolumab, including results from other clinical studies, are also available in the nivolumab Investigator Brochure (IB).

This study to investigate adjuvant treatment after a complete surgical resection of Stage IIB and IIC melanoma allows the enrollment of minors who are 12 years and older. There is no predicted



Approved v4.0 930137673 4.0

higher risk for toxicity for these pediatric patients, who are often seen by medical oncologists for cancer care versus pediatric oncologists given divergent expertise amongst these specialties.

The robust clinical activity demonstrated by nivolumab in participants with advanced melanoma, the manageable safety profile, and the lack of standard of care for participants who are at high risk for recurrence after a complete surgical resection of Stage IIB and IIC melanoma supports the further development of nivolumab in this population of participants.

It is the hypothesis of the trial that there will be improvements in RFS and OS in patients who have complete resections but are at high risk for recurrence of their melanoma. Since the median age of patients diagnosed with melanoma with localized disease is younger than that of other cancer patients, an improvement in RFS and OS is certainly meaningful, with the potential for years of improved quality of life and productivity, which on balance outweighs the risks of nivolumab treatment.

# 4 OBJECTIVES AND ENDPOINTS

**Table 4-1: Objectives and Endpoints[a]**

| Objectives | Endpoints |
|---|---|
| **Primary** | |
| To compare the efficacy, as measured by recurrence-free survival (RFS), provided by nivolumab monotherapy versus placebo in participants with completely resected Stage IIB/C melanoma with no evidence of disease who are at high risk for recurrence. | RFS |
| **Secondary** | |
| To compare the overall survival (OS) provided by nivolumab monotherapy versus placebo in participants with completely resected Stage IIB/C melanoma with no evidence of disease, who are at high risk for recurrence. | OS |
| To assess safety and toxicity of nivolumab monotherapy in participants with completely resected Stage IIB/C melanoma with no evidence of disease. | Occurrence and severity of AEs as defined by NCI CTCAE v5. |
| To evaluate distant metastases-free survival (DMFS) | DMFS |
| To evaluate investigator-assessed outcomes on next-line therapies. | • Objective response rates (if applicable)<br>• Duration of treatment on next-line therapies<br>• Progression-free survival through next-line therapy (PFS2) is defined as the time from randomization to recurrence/objective disease progression after the start of the next-line of systemic anti-cancer therapy, or to the start of a second next-line systemic therapy, or to death from any cause, whichever occurs first.<br>• End-of-next-line-treatment: To be used for situations where PFS2 cannot be reliably determined. Event defined as end or discontinuation of next-line |



Approved v4.0 930137673 4.0

**Table 4-1: Objectives and Endpoints[a]**

| Objectives | Endpoints |
|---|---|
| | treatment, second objective disease progression, or death from any cause, whichever occurs first. |
| **Exploratory** | |
| [redacted] | |
| To characterize the pharmacokinetics (PK) and explore exposure-response relationships with respect to safety and efficacy. | PK parameters and exposure metrics calculated from serum concentration-time data and characterization of exposure-response relationships |
| To characterize the immunogenicity of nivolumab. | Detection of anti-nivolumab antibodies |

Abbreviations: AE, adverse events; [redacted]



Approved v4.0 930137673 4.0

[redacted] NCI CTCAE, National Cancer Institute Common Terminology for Adverse Events; OS, overall survival; [redacted] PFS2, progression-free survival through next-line therapy; PK, pharmacokinetics; [redacted]

[a] Objectives and endpoints for participants entering the optional open-label nivolumab are very similar to blinded nivolumab/placebo participants with some subtle differences in endpoints to account for the open-label non-randomized nature of treatment and for participants presenting with recurrent unresectable/metastatic disease in the event of recurrence. [redacted]

# 5 STUDY DESIGN

## 5.1 Overall Design

Please refer to the Study Rationale Section (Section 3.1.1) for an overview of the study design.

The study design schematic is presented in Figure 5.1-1:

**Figure 5.1-1: Study Design Schematic (Applicable to Adult and Pediatric Participants ≥ 12 years old)**

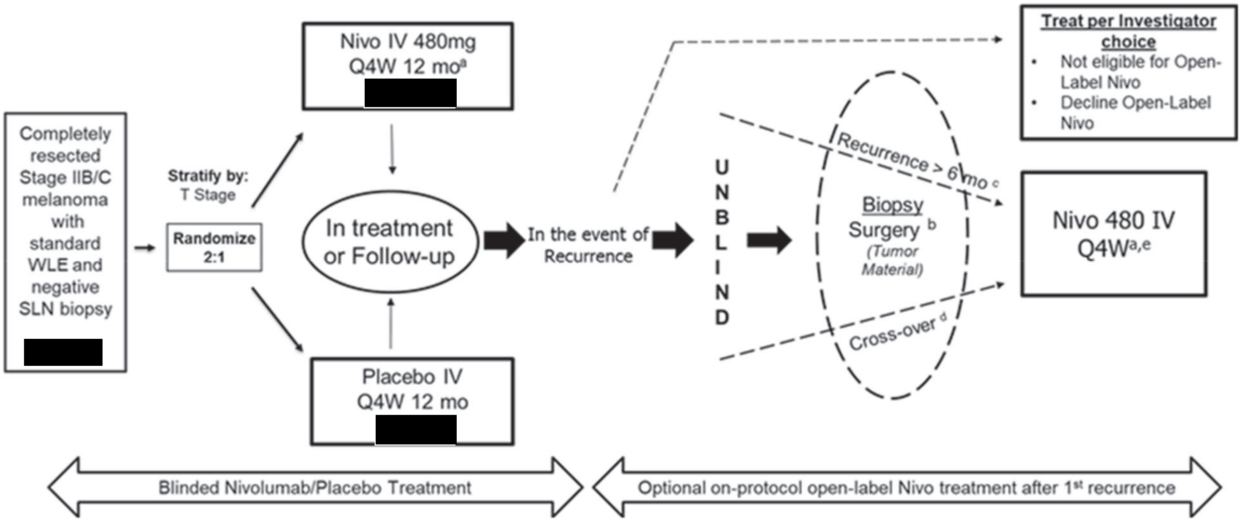


Abbreviations: IV, intravenous; Nivo, nivolumab; Q4W, every 4 weeks; SLN, sentinel lymph node; WLE, wide local excision.

[a] Pediatric dosing weight-based under 40 kg and for sites preferring weight-based dosing in pediatrics

[b] Tumor material obtained from surgery or biopsy

[c] Recurrence > 6 months after completion of 1 year of nivolumab treatment

[d] After recurrence, participant assigned to placebo arm will be offered to cross-over to nivolumab

[e] Resectable disease (Arm 1): Nivo 480 mg IV Q4W for 12 months
Unresectable disease (Arm 2): Nivo 480 mg IV Q4W for 24 months

### *5.1.1 Nivolumab or Placebo Blinded Treatment*

The Nivolumab or Placebo Blinded portion will consist of: screening, treatment, and follow-up, outlined below. **For a complete list of study required procedures, please refer to Section 2 (Schedule of Activities) and Section 9 (Study Assessment and Procedures).**



Approved v4.0 930137673 4.0

**Screening:**

- Establish participant's initial (pre-screen) eligibility, sign informed consent form (ICF). Record any hereditary risk factors for melanoma, if available, in the participant's medical history. Pay special attention to the skin and nodal examination.
- Register participant for screening using the IRT.
- Assess for complete study eligibility within the required timeframe found in Table 2-1.
- Ship tumor tissue as specified in Section 6.1 (Inclusion Criteria) and Section 9.8.2 (Tissue Specimens) to the central laboratory prior to randomization. Confirmation from central laboratory on receipt of Screening tumor tissue is not required for randomization in the IRT.
- WOCBP: Document negative pregnancy test within 24 hours prior to first study treatment.
- Submit screening/baseline imaging to the radiology vendor. Participant management/study enrollment is based on the investigator's assessment of disease-free status.
- Laboratory and other tests may be repeated as needed. Eligibility is assessed by most current result prior to randomization (see Section 6.1).

**Treatment:**

- Following eligibility confirmation, participant will be randomly assigned (2:1), within IRT, to either nivolumab (Arm A) or placebo (Arm B).
- On-study laboratory assessments: Draw within 72 hours prior to dosing. WOCBP: Pregnancy test every cycle prior to dosing.
- Physical examination: Within 72 hours prior to dosing. Pay special attention to skin and nodal examination.
- Within 3 calendar days (72 hours) from randomization, participant must receive the first dose of study medication.
- [redacted]
- Adverse event assessments: Document at each clinic visit.
- PK, immunogenicity, [redacted] Collect per Table 9.5-1, [redacted]
- Tumor surveillance assessments: Every 26 weeks (± 14 days), starting from the first dose of study treatment, throughout the 12 months of treatment. No central imaging assessments are planned; submit scans to a central imaging vendor for holding.

***Treatment Arms and Treatment Duration Details***

All participants on nivolumab or placebo will be treated until recurrence, unacceptable toxicity, participant withdrawal of consent, or a maximum of 12 months from first dose of study treatment, whichever occurs first. Dosing details for the randomized blinded treatment are provided below.



Approved v1.0 930137673 4.0

- ***Arm A (Nivolumab):***
  - *Adult Dosing***:**
    - ♦ Nivolumab 480 mg IV flat dose over approximately 30 minutes every 4 weeks (Q4W).
  - *Pediatric Dosing***:**
    - ♦ 12-17 years of age and weighing ≥ 40 kgs: Nivolumab 480 mg IV Q4W.
    - ♦ 12-17 years of age and weighing < 40 kgs: 6 mg/kg Q4W up to a maximum of 240 mg.
    - ♦ For centers that do not prefer to use flat dosing: 6 mg/kg Q4W (maximum of 240 mg for weight < 40 kgs; maximum of 480 mg for weight ≥ 40 kgs).
    - ♦ All nivolumab infusions to be administered over approximately 30 minutes.
- ***Arm B***: Adults and Pediatrics: Nivolumab matched placebo (0.9% Sodium Chloride for Injection/5% Dextrose for Injection) IV over approximately 30 minutes Q4W.

**Follow-Up:**

- Begins after 12 months of treatment or when the decision is made to discontinue a participant from study therapy.

***Safety Assessments***

- Two in-person follow-up safety assessment visits, 30 days (FU1) and 100 days (FU2) after the last dose of study treatment.
- Following Visits 1 and 2, conduct additional in-person safety assessments approximately every 12 weeks for 12 months (1 year). These assessments can be conducted along with the additional physical examinations described below in the section "Monitoring for Disease Recurrence". Please refer to the Schedule of Activities, Table 2-3 for additional details regarding safety follow-up after discontinuation of study treatment.

***Monitoring for Disease Recurrence***

- After Follow-up visit 2: Participants will visit the clinic for a physical examination approximately every 12 weeks for 12 months (1 year), with special attention to skin and nodal examination. For participants initiating subsequent systemic anti-cancer therapy for a melanoma recurrence, other than receiving optional open-label nivolumab in this study, or for a new non-melanoma cancer, follow-up assessments can be done per local standard of care.
- Tumor surveillance assessments: Every 26 weeks ± 14 days during the first, second and third year; every 52 weeks ± 28 days from 36 months to 60 months (all timepoints relative to first dose). Participants who develop a loco-regional recurrence only, must be followed by surveillance imaging until the development of distant metastases. Note: If a participant starts systemic therapy for melanoma recurrence after study drug discontinuation, other than optional open-label nivolumab, or for a new non-melanoma cancer, follow-up scans can be done as per standard of care.



Approved v1.0 930137673 4.0

[redacted] ***PK,*** [redacted]

- PK/immunogenicity samples: See Table 9.5-1.

[redacted]

***Survival Status Follow-Up (in-person or by telephone)***

- Every 12 weeks (± 14 days) following the second follow-up visit, calculated from first dose of study treatment until the OS final analysis.

***In the event of disease recurrence***

- Treatment unblinding will occur if knowledge of the treatment assignment is necessary to determine subsequent systemic anti-cancer therapy (including participation in the optional open-label nivolumab treatment). Please refer to Section 7.3.1 (Unblinding: emergency, Accidental, and In the Event of Disease Recurrence) for details regarding unblinding.
- Participants agreeing to optional open-label nivolumab treatment: Follow assessments described in detail in Section 2 (Schedule of Activities).
- Participants opting to not participate in open-label nivolumab treatment: Treat per investigator's choice; continue to follow the Schedule of Activities (Table 2-3).
- Submit biopsy tissue at recurrence and de-identified pathology reports of biopsies confirming recurrence to the central vendor.

### 5.1.2 Optional On-Protocol Open-Label Nivolumab Treatment After First Recurrence

In the event of disease recurrence, participants on the blinded nivolumab or placebo portion will be offered the option to receive open-label on-protocol nivolumab treatment. The primary objective of the overall study is to compare the RFS of nivolumab versus placebo. The inclusion of open-label nivolumab provides an opportunity for patients entering the randomized blinded study to gain access to nivolumab, if clinically indicated, in the event of recurrence.

The optional nivolumab treatment portion of the study will consist of screening, treatment, and follow-up. Key study elements are outlined briefly below. **For a comprehensive list of study required procedures, please refer to Section 2 (Schedule of Activities) and Section 9.1 (Study Assessment and Procedures).**

**Screening:**

- Participants sign a separate optional informed consent form (ICF). For all other screening requirements, please refer to the Schedule of Activities table (Table 2-1) and eligibility criteria (Section 6).
- Submit images to central imaging vendor. Participant management/study enrollment is based on the investigator's assessment of disease status.



Approved v1.0 930137673 4.0

- After meeting all eligibility criteria, participant is assigned to the appropriate arm for open-label nivolumab using the IRT.

**Treatment:**

For safety, efficacy, [redacted] PK, immunogenicity [redacted] assessments required during treatment, please refer to the Schedule of Activities table (Table 2-2).

- Submit images to central imaging vendor. Participant management during treatment is based on the investigator's assessment of disease recurrence/progression.
- Within 3 calendar days (72 hours) of assignment to open-label treatment with nivolumab, the participant must receive the first dose of study medication (Day 1 of Cycle 1).

***Treatment Arms and Duration:***

- Participants will be treated until recurrence/progression, unacceptable toxicity, participant withdrawal of consent, or for a maximum of 12 months (*Arm 1: resectable disease*) or 24 months (*Arm 2: unresectable/metastatic disease*) from the first dose of open-label study treatment, whichever occurs first. Dosing details for nivolumab are as described in Section 5.1 under "Treatment".

**Follow-Up of Participants Receiving Open-Label Nivolumab**

- Begins after 1 year (12 months) of treatment for Arm 1 participants and after 2 years (24 months) of treatment or when the decision is made to discontinue a participant from study therapy.

***Monitoring for Disease Recurrence/Progression***

- Submit scans to central imaging vendor. Tumor surveillance assessments per Schedule of Activities (Table 2-3).
- Submit de-identified pathology reports of biopsies confirming recurrence/progression to the central vendor.

***Safety Assessments, [redacted] PK, Immunogenicity, [redacted] and Survival Status Follow-Up***

- Please refer to Schedule of Activities (Table 2-3).

### 5.1.3 Data Monitoring Committee and Other External Committees

A Data Monitoring Committee (DMC) will be established to provide oversight of safety and efficacy considerations in Protocol CA20976K. Additionally, the DMC will provide advice to the sponsor regarding actions the committee deems necessary for the continuing protection of



Approved v1.0 930137673 4.0

participants enrolled in the study. The DMC will be charged with assessing such actions in light of an acceptable benefit/risk profile for nivolumab in both blinded treatment, and in the optional open-label nivolumab treatment after first recurrence portion of the trial. The DMC will act in an advisory capacity to BMS and will monitor participant safety and evaluate the available efficacy data for the study. The oncology therapeutic area of BMS has primary responsibility for design and conduct of the study.

Details of the DMC responsibilities and procedures will be specified in the DMC charter.

A Study Steering Committee (SSC), consisting of Investigators and personnel members representing the Sponsor of the study, will be established to obtain scientific guidance and advice for the protocol and conduct of the study. Details of the SSC responsibilities and procedures will be specified in the SSC charter.

## 5.2 Number of Participants

Approximately [redacted] participants diagnosed with Stage IIB/C melanoma that has been completely resected and who have no evidence of disease will be randomized (2:1) to nivolumab monotherapy or placebo.

## 5.3 End of Study Definition

The start of the trial is defined as first patient first visit. End of trial/study is defined as the completion of the the last visit or scheduled procedure shown in the SCHEDULE OF ACTIVITIES for the last participant, or the final date on which data for the primary endpoint was or is expected to be collected, if this is not the same. Additional information regarding post-study treatment follow-up can be found in Section 8.1.1.

## 5.4 Scientific Rationale for Study Design

CA20976K (CA20976K: CHECKpoint pathway and nivoluMAb clinical Trial Evaluation 76K) is a Phase 3 randomized, double blind study of nivolumab monotherapy versus placebo in participants with completely resected Stage IIB and IIC melanoma with no evidence of disease. The current standard of care for patients presenting with Stage IIB/C is a wide local excision followed by a sentinel lymph node biopsy. Following surgery, observation is the current gold standard. Interferon-alfa is the only adjuvant therapy that is offered as a possible treatment option in this patient population due to benefit seen in relapse/disease-free survival, with an unclear benefit in OS. Given the unexceptional benefit and high toxicity profile in a patient population that is free of disease, it is controversial whether interferon can be considered standard of care for Stage IIB/C melanoma. As a result, most patients are generally observed until recurrence of disease, at which point subsequent treatment is initiated depending on the site and extent of the recurrence. A placebo infusion was therefore chosen as the comparator arm on this trial.

Nivolumab (OPDIVO®) as a single agent was chosen as the active arm as it has demonstrated clinical benefit in participants with unresectable or metastatic melanoma, as well as in Stage III and IV resected melanoma. It has been approved for use in multiple countries, including the US and EU, and has been approved for several other indications (eg, metastatic NSCLC, advanced RCC, cHL). There is a reasonable expectation that nivolumab may be effective in resected Stage



Approved v4.0 930137673 4.0

IIB/C melanoma based on the demonstrated effectiveness of nivolumab first proven in unresectable/metastatic melanoma and subsequently as an adjuvant therapy in Stage III/IV completely resected melanoma.

Based on nivolumab's efficacy in more advanced melanoma settings (Stage III/IV adjuvant and Stage III/IV unresectable/metastatic), the 2:1 randomization provides an opportunity to offer this therapy to more Stage IIB/C patients who have a prognosis similar to Stage IIIB patients (per American Joint Committee on Cancer [AJCC] 8th edition). Direct evidence of nivolumab's benefit in this setting, while previously limited, is evolving,[60] and we hypothesize that participants assigned to the nivolumab arm may derive greater benefit versus participants in the placebo arm. The 2:1 randomization facilitates gaining additional experience, including additional safety data on nivolumab, to characterize and better inform the risk-benefit profile in this earlier Stage IIB/C melanoma study population, in which observation following wide local excision and negative sentinel lymph node involvement for melanoma is the generally accepted current standard of care.

[redacted]

### 5.4.1 Rationale for Treatment with Blinded Nivolumab

There is currently an unmet medical need for effective adjuvant therapies for patients diagnosed with Stage IIB and IIC cutaneous melanoma. Results from this study will help to define the role of nivolumab as an adjuvant immunotherapy in patients with Stage IIB and IIC cutaneous melanoma.

Cutaneous malignant melanomas are staged according to the AJCC (see Appendix 1 for List of Abbreviations) tumor/node/metastasis (TNM) classification and staging system. The AJCC 8th edition tumor staging for patients presenting with Stage II cutaneous malignant melanomas is shown below.[61]

| When T is… | And N is… | And M is…. | The pathological Stage is…. |
|---|---|---|---|
| T2b/T3a | N0 | M0 | IIA |
| T3b/T4a | N0 | M0 | IIB |
| T4b | N0 | M0 | IIC |
| T2: >1.0–2.0 mm; T3: >2.0–4.0 mm; T4: >4.0 mm; a: without ulceration; b: with ulceration; N0: No regional metastases detected; M0: No evidence of distant metastasis | | | |

Stage IIB (T3b/T4a, N0, M0) and IIC (T4b, N0, M0) melanomas are classified as localized melanomas without nodal involvement but are a higher risk category compared to Stage I and IIA melanomas due to a relatively worse prognosis. Patients diagnosed with Stage IIB and IIC melanoma have 5-year melanoma-specific survival (MSS) reported as 87% and 82%, respectively. The corresponding 10-year survival rates have been reported as 82% and 75%, respectively. Initial relapses are reported most frequently as local/in-transit after resection of Stage IIB melanoma (47%), while systemic relapses are more frequent after resection of Stage IIC melanoma (52%).[61]



Approved v1.0 930137673 4.0

Lung is the most frequent site of systemic relapse reported for both subtypes, with the second most frequent site being the liver for patients diagnosed with Stage IIB and brain for patients diagnosed with Stage IIC melanoma.[62] Once disease recurs, the site of initial relapse is the primary determinant of post-recurrence survival. Patients with systemic relapses have historically had a 5-year survival rate of 7% followed by 22, 33 and 57% for nodal, in-transit and local relapses respectively. [63] These poor prognoses in patients with both systemic and local relapse highlight the importance of controlling localized disease to prevent melanoma metastases.[62]

The rationale for adjuvant treatment for patients diagnosed with localized melanoma stems from the fact that most deaths from melanoma will occur in patients initially diagnosed with localized early stage disease without lymph node involvement.[64] The relatively similar prognosis of patients with Stage IIB/C melanoma and patients with Stage IIIB melanoma (5-year MSS for Stage IIIB = 83%), the poorer prognosis for these patients after relapse, underscore the need to prevent local or systemic relapses and provide support for the study of new systemic interventions to prevent metastases in the Stage IIB/C adjuvant melanoma setting.

Nivolumab (BMS-936558) is a fully human, IgG4 (kappa) isotype monoclonal antibody (mAb) that binds programmed death-1 (PD-1) on activated immune cells and disrupts engagement of the receptor with its ligands programmed death-ligand 1 (PD-L1) (B7 H1/CD274) and programmed death-ligand 2 (PD-L2) (B7-DC/CD273), thereby abrogating inhibitory signals and augmenting the host antitumor response. In clinical trials, nivolumab has demonstrated activity in several tumor types, including melanoma, renal cell carcinoma (RCC), and non-small cell lung cancer (NSCLC).

Nivolumab, with or without ipilimumab, is approved in the United States (US), European Union, and other countries for multiple indications. Nivolumab, as a monotherapy, and in combination with ipilimumab, is approved in Stage III/IV unresectable/metastatic melanoma.[65] Recently, nivolumab monotherapy has been approved as an adjuvant therapy in patients with completely resected Stage III and IV melanoma. The established safety and efficacy of nivolumab in patients with unresectable and metastatic melanoma, as well as in patients with resected Stage III melanoma, makes nivolumab an ideal candidate to study in patients with earlier Stage IIB/C melanoma, characterized by a lower disease burden, where immunotherapy may be more effective.

[redacted]



Approved v4.0 930137673 4.0

[redacted]



Approved v 1.0 930137673 4.0

[redacted]



Approved v4.0 930137673 4.0

[redacted]

### 5.4.5 Rationale for Blinding (Nivolumab Versus Placebo)

The study will be double-blinded in order to minimize bias arising from differences in thresholds for classification of recurrence between the arms, which could subsequently affect treatment duration between the arms and have an impact on the primary endpoint of RFS. In addition, blinding will reduce bias in reporting, classification, and management of adverse events. Participants with recurrence who discontinue treatment may require knowledge of which treatment arm they were assigned to in order to appropriately select any post-recurrence subsequent therapy (including treatment of participants in the optional open-label nivolumab portion of the trial). Therefore, in the event of recurrence of disease and treatment discontinuation of each participant, the participant's treatment assignment will be unblinded via the Interactive Response Technology (IRT) to inform the appropriate subsequent treatment.

[redacted]



Approved v4.0 930137673 4.0

[redacted]



Approved v 1.0 930137673 4.0

[redacted]



Approved v4.0 930137673 4.0

[redacted]



Approved v4.0 930137673 4.0

[redacted]



Approved v4.0 930137673 4.0

[redacted]



Approved v4.0 930137673 4.0

[redacted]

## 5.5 Justification for Dose

### *5.5.1 Rationale for 480 mg Flat Dose Every 4 Weeks*

A nivolumab dose of 480 mg given every 4 weeks (Q4W) was selected for this study based on available PK, safety, and efficacy data.



Approved v4.0 930137673 4.0

Nivolumab PK has been extensively studied in multiple tumor types, including melanoma, NSCLC, RCC, cHL, SCCHN, CRC, and urothelial carcinoma and has been safely administered at doses up to 10 mg/kg Q2W. Nivolumab monotherapy was originally approved as a body-weight based dose of 3 mg/kg Q2W, and was recently updated to 240 mg Q2W or 480 mg Q4W in multiple indications.[101] Less frequent 480 mg Q4W dosing regimens can reduce the burden to patients of frequent, lengthy IV treatments and allow combination of nivolumab with other agents using alternative dosing regimens.

The benefit-risk profiles of nivolumab 240 mg Q2W and 480 mg Q4W are predicted to be comparable to 3 mg/kg Q2W. This assessment is based on a comprehensive characterization of nivolumab PK, safety, efficacy, and exposure-response relationships across indications. Population PK (PPK) analyses have shown that the PK of nivolumab is linear with proportional exposures over a dose range of 0.1 to 10 mg/kg; no clinically meaningful differences in PK across ethnicities and tumor types were observed. Using the PPK model, the exposures following administration of several dosing regimens of nivolumab administered as a flat dose were simulated, including 240 mg Q2W and 480 mg Q4W. The simulated average serum concentration at steady state [Cavgss] following administration of nivolumab 480 mg Q4W are predicted to be similar to those following administration of nivolumab 240 mg Q2W and nivolumab 3 mg/kg Q2W administered to participants over a wide body weight range (34-180 kg) across tumor types.

Extensive exposure-response (E-R) analyses of multiple PK measures (maximum serum concentration at Day 1[Cmax1], average serum concentration at Day 28 [Cavg28], and trough serum concentration at Day 28 [Cmin28]) and efficacy and safety endpoints indicated that the efficacy of the flat-dose 480 mg IV regimen are similar to that of 3 mg/kg Q2W IV regimen. In E-R efficacy analyses for OS and ORR conducted in melanoma, RCC, and NSCLC using Cavg28 as the exposure measure, probabilities of achieving a response and survival probabilities at 1 year and 2 years for IV 480 mg Q4W were similar to that of IV 3 mg/kg Q2W. In E-R safety analyses, it was demonstrated that the exposure margins for safety are maintained following nivolumab 480 mg Q4W, and the predicted risks of discontinuations due to AEs or death, AE Grade 3+, and immune-mediated AEs (IMAEs) Grade 2+ are similar following nivolumab 480 mg Q4W relative to nivolumab 3 mg/kg Q2W across tumor types. In addition, nivolumab exposures with 240 mg Q2W and 480 mg Q4W flat-dose IV regimens across tumor types are maintained well below the corresponding exposures observed with the well-tolerated 10 mg/kg IV nivolumab Q2W dose regimen.

Additional details on nivolumab safety and risk-benefit can be found in the investigator brochure.

[redacted]



Approved v1.0 930137673 4.0

[redacted]

### 5.5.3 Rationale for Nivolumab 30-Minute Infusion Duration

Nivolumab is currently approved using a 30-minute infusion as a monotherapy.[64]

# 6 STUDY POPULATION

For entry into the study, the following criteria MUST be met.

## 6.1 Inclusion Criteria

### 6.1.1 Inclusion Criteria - Blinded Nivolumab or Placebo Treatment

1) **Signed Written Informed Consent**
   a) Participants must have signed and dated an IRB/IEC approved written informed consent form in accordance with regulatory and institutional guidelines. This must be obtained before the performance of any protocol related procedures that are not part of normal participant care.
      i) For participants unable to give their written consent, in accordance with local regulations, one or both parents, a guardian, or a legally acceptable representative must be informed of the study procedures and must document permission by signing the ICF approved for the study prior to clinical study participation.
      ii) For minors: Each participant must be informed about the nature of the study to the extent compatible with his or her understanding. Should a participant become capable or reach the age of majority, his or her consent should be obtained as soon as possible. The explicit wish of a participant who is a minor or is unable to provide his or her written consent but who is capable of forming an opinion and assessing information to refuse participation in or to be withdrawn from the clinical study at any time should be considered by the investigator.
      iii) Minors who are judged to be of an age of reason as determined by local requirements should also give their assent. The assent should be documented based on local requirements. Continued assent should be documented when important new information becomes available that is relevant to the participant’s assent.
   b) Participants must be willing and able to comply with scheduled visits, treatment schedule, and laboratory testing.
2) **Type of Participant and Target Disease Characteristics**
   a) Participants must have been diagnosed with Stage IIB/C cutaneous melanoma (AJCC Staging, 8th edition; see Appendix 8) and have histologically confirmed melanoma that is completely surgically resected, with documented negative margins (per local standard) for



Approved v1.0 930137673 4.0

disease on resected specimens. All melanomas, except ocular/uveal and mucosal melanoma, regardless of primary site of disease will be allowed. Participants with Stage IIB/C melanoma who present with synchronous or metachronous Stage < 2 melanomas that have been completely resected will be allowed.

***NOTE: A copy of the de-identified pathology report(s) confirming Stage IIB or IIC disease and negative margins on the resected specimen must be sent to the Central Laboratory vendor prior to randomization.***

a) Complete resection with documented negative margins (per local standard) and sentinel lymph node assessment for presence/absence of disease, must be performed within 12 weeks prior to randomization. Note: In case of delays exceeding 12 weeks due to unforeseen circumstances, the eligibility should be discussed with the Medical Monitor or designee.
b) Participants must have a negative sentinel lymph node biopsy. Participants in whom a sentinel lymph node biopsy procedure could not be done or a sentinel lymph node was not detected are not eligible.

***NOTE: A copy of the de-identified pathology report confirming sentinel lymph node negativity must be sent to the Central Laboratory vendor prior to randomization.***

c) The pathology report for Stage IIB, IIC, and sentinel lymph node biopsy must be reviewed, and review documented by the investigator.
d) Participants must have disease-free status documented by a complete physical examination (within 14 days) and imaging studies within 4 weeks (28 days) prior to randomization. Imaging studies must include CT scans of the chest/abdomen/pelvis or CT scan of the chest and MRI scans of the abdomen and pelvis, and all known sites of resected disease (imaging of extremities for resected melanomas located in the extremities is not a requirement and may be conducted per local standard of care). Participants with signs and symptoms consistent with brain metastases should have imaging studies done to rule out the presence of brain metastases.
    i) Participants with equivocal lymph nodes on imaging (≥ 10 mm and < 15 mm in a short axis) may be eligible if confirmation with histology/cytology is available. If risk of biopsy is too high or biopsy is not feasible, two sequential CT or MRI scans (as listed above) should be available and showing no progressive and measurable (lymph nodes ≥ 15 mm in short axis) disease or PET/CT demonstrating no FDG uptake. The second scan should occur at least 4 weeks after the initial scan. Participants with incidental findings (eg, indeterminate nodules noted on imaging) reported during screening may be eligible if confirmation of disease-free status can be documented by histology/cytology, imaging studies, or in certain situations by discussing with the Medical Monitor or designee. Note: Participants who have had a history of metastatic melanoma and have had their metastatic melanoma resected are not eligible.
e) The complete set of baseline images must be available before randomization.
f) Participant has not been previously treated for melanoma beyond complete surgical resection of the melanoma lesion.
g) Participant has recovered adequately from toxicity and/or complications from surgery prior to study start.



Approved v1.0 930137673 4.0

h) ECOG performance status of 0 or 1 at the time of enrollment (Appendix 6)

i) Tumor tissue from the primary diagnostic biopsy (minimum of 15 unstained slides, preferably freshly cut, or 1 FFPE block to contain sufficient tissue for at least 15 sections) must be shipped to the central laboratory prior to randomization (see Section 9.8.2 for details). If the required tumor tissue content cannot be provided, the eligibility should be discussed with the Medical Monitor or designee.

j) Participants must be able and willing to comply with the study visit schedule and study procedures.

**3) Age and Reproductive Status**

a) Males and Females, ≥ 12 years of age

**i) Except: where local regulations and/or institutional policies do not allow for participants < 18 years of age (pediatric population) to participate. For those sites, the eligible participant population is ≥ 18 years of age**

b) Women of childbearing potential (WOCBP) must have a negative serum or urine pregnancy test (minimum sensitivity 25 IU/L or equivalent units of HCG) within 24 hours prior to the start of study treatment. An extension up to 72 hours prior to the start of study treatment is permissible in situations where results cannot be obtained within the standard 24-hour window.

c) Women of childbearing potential (WOCBP) must agree to follow instructions for method(s) of contraception for the duration of treatment with study treatment(s) plus 5 half-lives of study treatment plus 30 days (duration of ovulatory cycle) for a total of 5 months after the last dose of study treatment.

4) NOTE: As per Protocol Amendment 02, nivolumab IB v19, male study participants will not be required to use contraceptive measures and/or a latex or other synthetic condom during sexual activity with a WOCBP partner. WOCBP who are continuously not heterosexually active are also exempt from contraceptive requirements, and still must undergo pregnancy testing as described in this section.

a) **Not applicable as per Protocol Amendment 02.** Males who are sexually active with WOCBP must agree to follow instructions for method(s) of contraception for a total of 7 months post-treatment completion [ie, 90 days (duration of sperm turnover) plus the time required for study drug to undergo approximately five half-lives]. In addition, male participants must be willing to refrain from sperm donation during this time.

b) **Not applicable as per Protocol Amendment 02.** Azoospermic males are exempt from contraceptive requirements unless the potential exists for fetal toxicity due to study drug being present in seminal fluid, even if the participant has undergone a successful vasectomy or if the partner is pregnant. WOCBP who are continuously not heterosexually active are also exempt from contraceptive requirements, and still must undergo pregnancy testing as described in this section.

Investigators shall counsel WOCBP on the importance of pregnancy prevention and the implications of an unexpected pregnancy and when applicable, the potential of fetal toxicity occurring due to transmission of study drug to a developing fetus. Investigators shall advise on the



Approved v4.0 930137673 4.0

use of highly effective methods of contraception (Appendix 4), which have a failure rate of < 1% when used consistently and correctly.

### 6.1.2 Inclusion Criteria for Participants Receiving Treatment after Recurrence

1) **Signed Written Informed Consent**
   a) Participants must have signed and dated a separate IRB/IEC approved written informed consent form for open-label treatment with nivolumab monotherapy in accordance with regulatory and institutional guidelines. All other informed consent related details discussed in Section 6.1.1 for participants receiving blinded treatment apply to participants receiving treatment after recurrence.

2) **Type of Participant and Target Disease Characteristics**
   a) Participant must have been randomized, treated and have documented recurrence (per Section 9.1.2.1) of melanoma on blinded nivolumab or placebo treatment
   b) Recurrent disease must have been diagnosed within 3 years of last dose of blinded study. Participants assigned to blinded nivolumab treatment must have completed 1 year of therapy and have experienced disease recurrence more than 6 months following the last dose.
   c) Participant must not have received any other systemic anticancer therapy (including investigational anticancer therapy) or loco-regional anticancer therapy (other than surgery for complete resection of the recurrence and radiation therapy administered with a palliative intent) between the last dose of blinded study treatment and the first dose of open-label study treatment.

      ***NOTE: Prior adjuvant radiation therapy (RT) after neurosurgical resection for central nervous system (CNS) lesions or prior RT to CNS metastases is allowed.***

   d) Palliative/definitive radiotherapy, if administered, must have been completed at least 2 weeks prior to study drug administration
   e) ***Arm 1 (resectable disease only):*** Complete documented resection of recurrent disease (preferably within 12 weeks prior to assignment to open-label treatment) with margins negative (per local standard) for disease. For CNS lesion(s), documentation indicating that there has been complete resection of CNS lesion(s) will suffice as confirmation of negative margins.

      ***NOTE: A copy of the de-identified pathology report(s) confirming negative margins on the resected specimen must be sent to the Central Laboratory vendor prior to assignment to open-label treatment.***

   f) ***Arm 1 (resectable disease only):*** Participants must have disease-free status documented by a complete physical examination and imaging studies within 4 weeks (28 days) prior to assignment to open-label treatment. See Table 2-1 (Screening Schedule of Activities) for imaging related details.
   g) ***Arm 2: (unresectable/metastatic disease only):*** Participants with brain metastases are eligible if brain metastases have been treated and there is no MRI evidence - except where contraindicated in which CT scan is acceptable of progression for at least 8 weeks after treatment is complete and within 28 days prior to first dose of open-label study drug



Approved v1.0 930137673 4.0

administration. Cases should be discussed with the Medical Monitor or designee. There must also be no requirement for immunosuppressive doses of systemic corticosteroids (> 10 mg/day prednisone equivalents) for at least 2 weeks prior to study drug administration.

h) Participant has recovered adequately from toxicity and/or complications from prior blinded study treatment and surgery or palliative radiotherapy prior to assignment.

i) ECOG performance status of 0 or 1 at the time of enrollment (Refer to Appendix 6)

j) Tumor tissue from the recurrent site of disease per Section 9.8.2.

3) **Age and Reproductive Status** (All age and reproductive status related details discussed in Section 6.1.1 for participants receiving blinded therapy apply to participants opting to receive open-label nivolumab)

## 6.2 Exclusion Criteria

### *6.2.1 Exclusion Criteria - Blinded Nivolumab or Placebo Treatment*

1) **Medical Conditions**

a) History of ocular or mucosal melanoma.

b) Participants with active, known, or suspected autoimmune disease. Participants with type I diabetes mellitus, hypothyroidism only requiring hormone replacement, skin disorders (such as vitiligo, psoriasis, or alopecia) not requiring systemic treatment or conditions not expected to recur in the absence of an external trigger are permitted to enroll.

c) Prior malignancy active within the previous 3 years except for locally curable cancers that have been apparently cured, such as basal or squamous cell skin cancer, superficial bladder cancer, or carcinoma in situ of the prostate, cervix, or breast.

d) Participants with a condition requiring systemic treatment with either corticosteroids (> 10 mg daily prednisone equivalent) or other immunosuppressive medications within 14 days of randomization. Inhaled or topical steroids, and adrenal replacement steroid doses > 10 mg daily prednisone equivalent, are permitted in the absence of active autoimmune disease.

e) Women who are pregnant or breastfeeding.

f) Participants with serious or uncontrolled medical disorders. Additionally, in the case of prior SARS-CoV-2 infection, symptoms must have completely resolved and based on investigator assessment in consultation with the BMS Medical Monitor or designee, there are no sequelae that would place the participant at a higher risk of receiving investigational treatment.

2) **Prior/Concomitant Therapy**

a) Use of an investigational agent or an investigational device within 28 days before administration of first dose of study drug.

b) Treatment directed against the resected melanoma (eg, chemotherapy, radiation therapy, targeted agents, biotherapy, or limb perfusion) that is administered after the complete resection.

c) Prior treatment with an anti-PD-1, anti-PD-L1, anti-PD-L2, anti-CD137, anti-CTLA-4 antibody, or agents that target IL-2 pathway any other antibody or drug specifically targeting T-cell co-stimulation or checkpoint pathways. Exception: Prior adjuvant



Approved v1.0 930137673 4.0

treatment with interferon (for melanoma other than study entry melanoma) is allowed if completed ≥ 6 months prior to randomization.

d) Treatment with complementary medications (eg, herbal supplements or traditional Chinese medicines) to treat the disease under study within 2 weeks prior to randomization/treatment. Such medications are permitted if they are used as supportive care. Refer to Section 7.7.1 for prohibited therapies.

e) Participants who have received a live / attenuated vaccine within 30 days of first treatment.

f) The concomitant use of topical Toll-like receptor 7 (TLR7) agonists (eg, imiquimod), calcineurin inhibitors (eg, tacrolimus), or topical immunotherapy/contact sensitizer preparations, eg, but not limited to 2,4-dinitrochlorobenzene (DNCB), squaric acid dibutylester (SADBE), and diphencyprone (DPCP) at or near the primary tumor site, are not specifically excluded, but consultation with the Medical Monitor or designee is highly recommended prior to enrolling participants using these agents.

g) Participants currently in other interventional trials, including those for coronavirus disease 2019 (COVID-19), may not participate in BMS clinical trials until the protocol-specific washout period is achieved. If a study participant has received an investigational COVID-19 vaccine prior to Screening, enrollment must be delayed until the full dosing schedule of the vaccine has been completed and the biologic impact of the vaccine is stabilized, unless a delay would compromise the participant's health or suitability for enrollment, as determined by discussion between the Investigator and the BMS Medical Monitor or designee.

3) **Physical and Laboratory Test Findings**

a) WBC < 2000/μL

b) Neutrophils < 1500/μL

c) Platelets < $100 \times 10^3$/μL

d) Hemoglobin < 9.0 g/dL

e) Serum creatinine > 1.5 x ULN, unless creatinine clearance ≥ 40 mL/min (measured or calculated using the Cockroft-Gault formula)

Female CrCl = [(140 - age in years) x weight in kg x 0.85] ÷ [72 x serum creatinine in mg/dL]

Male CrCl = [(140 - age in years) x weight in kg x 1.00] ÷ [72 x serum creatinine in mg/dL]

f) AST/ALT: > 3.0 x ULN

g) Total bilirubin > 1.5 x ULN (except participants with Gilbert Syndrome who must have a total bilirubin level of < 3.0 x ULN)

h) Any positive test result for hepatitis B virus or hepatitis C virus indicating presence of virus, eg, Hepatitis B surface antigen (HBsAg, Australia antigen) positive, or Hepatitis C antibody (anti-HCV) positive (except if HCV-RNA negative).

i) Known history of positive test for human immunodeficiency virus (HIV) or known acquired immunodeficiency syndrome (AIDS). Note: Testing for HIV must be performed at sites where mandated locally.

4) **Allergies and Adverse Drug Reaction**

a) Known history of allergy or hypersensitivity to study drug components.



Approved v1.0 930137673 4.0

b) Known history of severe hypersensitivity reaction (Grade ≥ 3) to any monoclonal antibody.

5) **Other Exclusion Criteria**

a) Prisoners or participants who are involuntarily incarcerated. (Note: under certain specific circumstances, and only in countries where local regulations permit, a person who has been imprisoned may be included or permitted to continue as a participant. Strict conditions apply and Bristol-Myers Squibb approval is required.

b) Participants who are compulsorily detained for treatment of either a psychiatric or physical (eg, infectious disease) illness.

### *6.2.2 Exclusion Criteria for Participants Receiving Treatment After Recurrence*

1) **Medical Conditions**

a) Recurrent disease diagnosed greater than (>) 3 years after last dose of blinded study treatment.

b) Participants who experience a second recurrence or progression before enrollment into the open-label treatment portion of the trial.

NOTE: All other exclusion criteria described in Section 6.2.1 for participants receiving blinded study treatment apply to participants opting to receive open-label treatment after first recurrence. To address exclusion 2c, prior treatment with nivolumab, an anti-PD-1 agent, due to participation in the blinded-treatment on this trial, is permitted. Prior adjuvant radiation therapy (RT) after neurosurgical resection for central nervous system (CNS) lesions or prior RT to CNS metastases is allowed.

## 6.3 Lifestyle Restrictions

Not applicable. No restrictions are required.

## 6.4 Screen Failures

Screen failures are defined as participants who consent to participate in the clinical study but who are not subsequently randomized (blinded-treatment participants) or enrolled (open-label nivolumab treatment after first recurrence) in the study/included in the analysis population. A minimal set of screen failure information is required to ensure transparent reporting of screen failure participants, to meet the Consolidated Standards of Reporting Trials (CONSORT) publishing requirements, as applicable, and to respond to queries from regulatory authorities. Minimal information includes date of consent, demography, screen failure details, eligibility criteria, and any serious AEs.

### *6.4.1 Retesting During Screening or Lead-In Period*

Participant Re-enrollment: This study permits the re-enrollment of a participant that has discontinued the study as a pre-treatment failure (ie, participant has not been randomized / has not



Approved v1.0 930137673 4.0

been treated). If re-enrolled, the participant must be re-consented and assigned a new participant number from IRT.

Retesting of laboratory parameters and/or other assessments within any single screening period will be permitted (in addition to any parameters that require a confirmatory value).

The most current result prior to randomization is the value by which study inclusion will be assessed, as it represents the participant's most current, clinical state.

Laboratory parameters and/or assessments that are included in Section 2, Screening Procedural Outline may be repeated in an effort to find all possible well-qualified participants. Consultation with the Medical Monitor may be needed to identify whether repeat testing of any particular parameter is clinically relevant.

Testing for asymptomatic COVID-19, for example, by reverse transcription polymerase chain reaction (RT-PCR) or viral antigen is not required. However, some participants may develop suspected or confirmed symptomatic COVID-19 infection or be discovered to have asymptomatic COVID-19 infection during the Screening period.[104] In such cases, participants may be considered eligible for the study after meeting all inclusion/exclusion criteria related to serious or uncontrolled medical disorders, and after meeting the following criteria:

- At least 10 days (20 days for severe/critical illness) have passed since symptoms first appeared or positive test result, and
- At least 24 hours have passed since last fever without the use of fever-reducing medications, and
- Symptoms (eg, cough, shortness of breath) have resolved, and
- In the opinion of the Investigator, there are no COVID-19 sequelae that may place the participant at a higher risk of receiving investigational treatment, and
- Negative follow-up RT-PCR or viral antigen test for COVID-19 based on institutional, local, or regional guidelines

# 7 TREATMENT

Study treatment is defined as any investigational treatment(s), marketed product(s), placebo or medical device intended to be administered to a study participant according to the study randomization or treatment allocation

Study treatment includes both Investigational [Medicinal] Product (IP/IMP) and Non-investigational [Medicinal] Product (Non-IP/Non-IMP) and can consist of the following:

An investigational product, also known as investigational medicinal product in some regions, is defined as a pharmaceutical form of an active substance or placebo being tested or used as a reference in a clinical study, including products already with a marketing authorization but used or assembled (formulated or packaged) differently than the authorized form, or used for an unauthorized indication, or when used to gain further information about the authorized form.



Approved v4.0 930137673 4.0

Other medications used as support or escape medication for preventative, diagnostic, or therapeutic reasons, as components of the standard of care for a given diagnosis, may be considered as non-investigational products.

The investigational products are nivolumab and 0.9% Sodium Chloride for Injection/5% Dextrose for Injection (Table 7-1). Solutions used as placebo (ie, 0.9% Sodium Chloride Injection or 5% Dextrose Injection) should also be sourced by investigative sites if available and permitted by local regulations.

Premedications or medications used to treat infusion-related reactions should be sourced by the investigative sites if available and permitted by local regulations.



Approved v4.0 930137673 4.0

**Table 7-1: Study Treatments for CA20976K**

| Product Description / Class and Dosage Form | Potency | IP/Non-IP | Blinded- or Open-Label | Packaging / Appearance | Storage Conditions (per label) |
|---|---|---|---|---|---|
| BMS-936558 (Nivolumab) Solution for Injection | 10 mg/mL | IP | Open-label[a] | Vials | Refer to the label on container and/or Pharmacy Manual |
| 0.9% Sodium Chloride for Injection | N/A | IP | Open-label[a] | Various (local commercial product) | As per package insert |
| 5% Dextrose for Injection | N/A | IP | Open-label[a] | Various (local commercial product) | As per package insert |

Abbreviations: IP, Investigational Product; Non-IP, Non-Investigational Product.

[a] The term “open-label” refers to the medication as it is upon receipt at the pharmacy. The blinded-treatment will be conducted in a double-blinded fashion.



Approved v4.0 930137673 4.0

## 7.1 Treatments Administered

### *7.1.1 Treatment with Nivolumab or Placebo*

Blinded Nivolumab/Placebo Treated Participants

Adult participants should receive nivolumab (or matching placebo) at a dose of 480 mg as an approximately 30-minute infusion on Day 1 of each treatment cycle until unacceptable toxicity, withdrawal of consent, completion of 12 months of treatment (from first dose of study treatment), disease recurrence, or the study ends, whichever occurs first. Participants should begin study treatment (Cycle 1) within 3 calendar days of randomization. Subsequent cycles could be initiated within ± 3 days of the target visit date. When the initiation of a subsequent cycle is delayed (eg, due to an AE in the preceding cycle), the target visit date of all future cycles should be calculated from the day the participant was actually administered the study treatment in the delayed subsequent cycle.

Pediatric participants (≥ 12 years old) who weigh ≥ 40 kg should receive nivolumab (or matching placebo) at a dose of 480 mg as an approximately 30-minute infusion on Day 1 of each treatment cycle every 4 weeks, until unacceptable toxicity, withdrawal of consent, completion of 12 months of treatment (from first dose of study treatment), disease recurrence, or the study ends, whichever occurs first. Participants should begin study treatment within 3 calendar days of randomization. The timing of initiation of subsequent cycles is the same as in adults.

For pediatric participants who weigh < 40 kg, nivolumab (or matching placebo) should be dosed at 6 mg/kg (maximum 240 mg, Q4W) as an approximately 30-minute infusion.

Weight based dosing (6 mg/kg up to a maximum of 240 mg for participants weighing < 40 kgs and a maximum of 480 mg for participants weighing ≥ 40 kgs) can be used for centers that do not prefer to dose nivolumab as a flat dose.

Pediatric dosing calculations should be based on the body weight assessed at baseline. It is not necessary to re-calculate subsequent doses if the participant weight is within 10% of the weight used to calculate the previous dose. All doses should be rounded up or to the nearest milligram per institutional standard.

Open-Label Nivolumab Treatment

Treatment details for adults and adolescent are as in Section 7.1.1 for blinded study treatment, except that the use of a matching placebo is not applicable to the open-label nivolumab portion of the trial. In addition, nivolumab 480 mg will be administered Day 1 of each treatment cycle until unacceptable toxicity, withdrawal of consent, completion of 12 months (Arm 1: resectable disease) or 24 months (Arm 2: unresectable/metastatic disease) of treatment (from first dose of open-label nivolumab), disease progression, or the study ends, whichever occurs first.

### *7.1.2 General Treatment Administration Considerations*

There will be no dose escalations or reductions of nivolumab allowed. Doses of nivolumab may be interrupted, delayed, or discontinued depending on how well the participant tolerates the treatment. Dosing visits are not skipped, only delayed.



Approved v1.0 930137673 4.0

For Q4W dosing cycles, participants may be dosed within a ± 3 day window. Premedications are not recommended for the first dose of nivolumab. Participants should be carefully monitored for infusion reactions during nivolumab administration. If an acute infusion reaction is noted, participants should be managed according to Section 7.4.2.

Nivolumab infusions are compatible with polyvinyl chloride (PVC) or polyolefin containers and infusion sets, and glass bottles.

Please see the nivolumab IB and pharmacy manual for specific infusion preparation, and infusion set/infusion bag compatibility recommendations.

## 7.2 Method of Treatment Assignment

The exact procedures for using the IRT will be detailed in the IRT manual.

### *7.2.1 Blinded Nivolumab or Placebo Treatment*

After the participant's initial eligibility is established and informed consent has been obtained, the participant must be enrolled into the study through IRT website to obtain the participant number. Every participant who signs the informed consent form must be assigned a participant number in IRT. Specific instructions for using IRT will be provided to the investigational site in a separate document. The investigator or designee will register the participant for enrollment by following the enrollment procedures established by BMS. The following information is required for enrollment:

- Date that informed consent was obtained
- Date of birth (Year of birth, if local regulations do not allow use of entire date of birth)
- Gender at birth

Once enrolled in IRT, enrolled participants who have met all eligibility criteria will be ready to be randomized through the IRT. The sponsor, participants, investigator, and site staff will be blinded to the study drug administered during the study. Each investigative site must assign an unblinded pharmacist/designee, and an unblinded site monitor will be assigned by sponsor to provide oversight of drug supply and other unblinded study documentation. The randomization call may be performed by the blinded study coordinator or another suitable individual designated by the investigator; a notification of the actual treatment assignment will be sent by the IRT to the unblinded pharmacy site staff. The following information is required for participant randomization:

- Participant number
- Date of birth (Year of birth, if local regulations do not allow use of entire date of birth)
- Participants will be stratified by:
  - − AJCC T-Stage, 8th edition (T3b vs. T4a vs. T4b)
- Participants will be treated with one of the following:



Approved v1.0 930137673 4.0

− Arm A: nivolumab 480 mg IV Q4W (for a maximum of 12 months from first dose of study treatment)

♦ Pediatric dosing as per Section 7.1.1

− Arm B: nivolumab matched placebo IV Q4W (for a maximum of 12 months from first dose of study treatment)

### 7.2.2 Optional Open-Label Nivolumab Treatment After First Recurrence

In the event of recurrence on blinded study treatment, participants may be eligible to receive open-label nivolumab monotherapy. The open-label nivolumab portion of the trial is comprised of two arms, without stratification or randomization. If participants agree to participate in the open-label nivolumab portion of this trial, the participant's treatment assignment in the randomized portion of the trial will be unblinded. Depending on the resectability of the recurrence (resectable versus not), as determined by the investigator/treating physician, participants will be assigned to either Arm 1 (resectable disease) or Arm 2 (unresectable/metastatic disease). The same participant ID assigned in the randomized, blinded portion of the trial will be carried forward in open-label nivolumab treatment phase.

Participants on Arm 1 and Arm 2 will be treated on a nivolumab regimen (480 mg IV Q4W) for a maximum of 12 and 24 months respectively.

Please see Section 7.1.1 further treatment details, including dosing flexibility in pediatrics.

Participants who have received blinded study treatment, experiencing recurrence, but who do not wish to participate in the open-label nivolumab portion of the trial, can be treated per standard of care by the investigator/treating physician and will continue to be followed per follow-up procedures outlined in the Schedule of Activities (Section 2).

For participants receiving open-label nivolumab monotherapy after recurrence, the information collected by the IRT will be similar to that collected prior to randomization, minus the stratification information.

## 7.3 Blinding

This is a randomized double-blind trial. For the optional open-label nivolumab portion, each participant will be assigned to the specific arm (Arm 1 or Arm 2) using an Interactive Response Technology (IRT). Access to treatment codes will be restricted from all participants, and site and BMS personnel prior to database lock, with exception as specified below.

Designated staff of Bristol-Myers Squibb Research & Development may be unblinded (obtain the randomization codes) prior to database lock to facilitate the bioanalytical analysis of pharmacokinetic samples and immunogenicity. A bioanalytical scientist in the Bioanalytical Sciences department of Bristol-Myers Squibb Research & Development (or a designee in the external central bioanalytical laboratory) will be unblinded to (may obtain) the randomized treatment assignments in order to minimize unnecessary bioanalytical analysis of samples.



Approved v1.0 930137673 4.0

### 7.3.1 Unblinding: Emergency, Accidental, and in the Event of Disease Recurrence

Blinding of treatment assignment is critical to the integrity of this clinical study. However, in the event of a medical emergency or pregnancy in an individual participant in which knowledge of the investigational product is critical to the participant's management, the blind for that participant may be broken by the investigator. The participant's safety takes priority over any other considerations in determining if a treatment assignment should be unblinded. Before breaking the blind of an individual participant's treatment, the investigator should determine that the unblinded information is necessary, ie, that it will alter the participant's immediate management. In many cases, particularly when the emergency is clearly not related to the investigational product, the problem may be properly managed by assuming that the participant is receiving active product. It is highly desirable that the decision to unblind treatment assignment be discussed with the Medical Monitor or designee, but the investigator always has ultimate authority for the decision to unblind. The actual task of unblinding can be delegated by the Investigator to a designee assigned the task on the Delegation of Authority. The Principal Investigator or appointed designee should only call in for emergency unblinding AFTER the decision to unblind the participant has been documented.

For this study, the method of unblinding for emergency purposes is IRT.

In cases of accidental unblinding, contact the unblinded Clinical Trial Monitor and ensure every attempt is made to preserve the blind.

Any request to unblind a participant for non-emergency purposes should be discussed with the Medical Monitor or designee.

In case of an emergency, the investigator(s) has unrestricted access to randomization information via the Interactive Response Technology (IRT) and is capable of breaking the blind through the IRT system without prior approval from sponsor. Following the unblinding the Investigator shall notify the Medical Monitor or designee.

### 7.3.2 Unblinding in the Event of Disease Recurrence

Unblinding participants in the event of disease recurrence is necessary for participants who opt to participate in the optional open-label nivolumab component. Unblinding may be requested for election of subsequent systemic therapy (other than participation in optional open-label nivolumab), if the investigator feels that the selection of subsequent systemic therapy may be influenced by knowledge of the treatment assignment.

At the time of recurrence, if the participant's treatment assignment is required to assign the next-line systemic therapy, an unblinding request should be made by the site investigator/staff to the BMS site monitor or a member of the BMS study trial management team, who will gain approval from the Medical Monitor or designee to proceed with unblinding of the treatment arm. The BMS site monitor or a member of the BMS study trial management team will work with the site to unblind the treatment arm through the IRT.



Approved v4.0 930137673 4.0

## 7.4 Dosage Modification

Dose reductions or dose escalations are not permitted. All dose modification rules apply to all arms given the blinded nature of this study.

### 7.4.1 *Criteria to Delay, Resume, or Discontinue Nivolumab or Nivolumab-Placebo*

Dose delay criteria apply for all study drug-related adverse events (AEs) regardless of whether or not the event is attributed to nivolumab or placebo given the blinded nature of this study. All study drugs must be delayed until treatment can resume. AEs will be reported and managed per National Cancer Institute (NCI) Common Terminology Criteria for Adverse Events version 5.0 (CTCAE, v5). Please refer to Table 7.4.1-1 below for AE criteria to delay, resume, or discontinue nivolumab or nivolumab-placebo. For additional criteria not covered in Table 7.4.1-1, please refer to the following sections for additional details: Section 7.4.1.1 for dose delay, Section 7.4.1.2 for dose resumption, and Section 7.4.1.3 for discontinuation from study treatment.

**Table 7.4.1-1: Adverse Event Criteria to Delay, Resume, or Discontinue Study Treatment**

| Drug-Related Adverse Event (AE) per CTCAE V5 | Severity | Action Taken | Clarifications, Exceptions, and Resume Criteria |
|---|---|---|---|
| **Gastrointestinal** | | | |
| Colitis or Diarrhea | Grade 2 or 3 | Delay dose | Dosing may resume when AE resolves to baseline |
| | Grade 4 | Permanently discontinue | |
| **Renal** | | | |
| Serum Creatinine Increased | Grade 2 or 3 | Delay dose | Dosing may resume when AE resolves to Grade ≤ 1 or baseline value |
| | Grade 4 | Permanently discontinue | |
| **Pulmonary** | | | |
| Pneumonitis | Grade 2 | Delay dose | Dosing may resume after pneumonitis has resolved to Grade ≤ 1. |
| | Grade 3 or 4 | Permanently discontinue | |
| **Hepatic** | | | |
| Aspartate aminotransferase (AST), alanine aminotransferase (ALT), or total bilirubin (T.bili) increased | AST or ALT > 3x and ≤ 5x upper limit of normal (ULN) or T.bili > 1.5x and ≤ 3x ULN, | Delay dose | Dosing may resume when laboratory values return to baseline. |



Approved v1.0 930137673 4.0

**Table 7.4.1-1: Adverse Event Criteria to Delay, Resume, or Discontinue Study Treatment**

| Drug-Related Adverse Event (AE) per CTCAE V5 | Severity | Action Taken | Clarifications, Exceptions, and Resume Criteria |
|---|---|---|---|
| | regardless of baseline value | | |
| | AST or ALT > 5x ULN or T.bili > 3x ULN, regardless of baseline value | Delay dose or permanently discontinue | In most cases of AST or ALT > 5x ULN, study treatment will be permanently discontinued. If the investigator determines a possible favorable benefit/risk ratio that warrants continuation of study treatment, a discussion between the investigator and the Medical Monitor/ designee must occur and approval from Medical Monitor prior to resuming therapy. |
| | Concurrent AST or ALT > 3x ULN and T.bili > 2x ULN, regardless of baseline value | Permanently discontinue | |
| **Endocrinopathy** | | | |
| Adrenal Insufficiency | Grade 2 adrenal insufficiency | Delay dose | Dosing may resume after adequately controlled with hormone replacement. |
| | Grade 3 or 4 adrenal insufficiency or adrenal crisis | Delay dose or permanently discontinue | Mandatory discussion with and approval from the Medical Monitor needed prior to resuming therapy. If adrenal insufficiency resolves or is adequately controlled with physiologic hormone replacement, participant may not require discontinuation of study drug. |
| Hyperglycemia | Hyperglycemia requiring initiation or change in daily management (Grade 2 or 3) | Delay dose | Dosing may resume if hyperglycemia resolves to Grade ≤ 1 or baseline value, or is adequately controlled with glucose-controlling agents. |
| | Grade 4 | Delay dose or permanently discontinue | Mandatory discussion with and approval from the Medical Monitor needed prior to resuming therapy. If hyperglycemia resolves, or is adequately controlled with glucose-controlling agents, participant may |



Approved v1.0 930137673 4.0

**Table 7.4.1-1: Adverse Event Criteria to Delay, Resume, or Discontinue Study Treatment**

| Drug-Related Adverse Event (AE) per CTCAE V5 | Severity | Action Taken | Clarifications, Exceptions, and Resume Criteria |
|---|---|---|---|
| | | | not require discontinuation of study drug. |
| Hypophysitis/Hypopituitarism | Symptomatic Grade 1-3 that is also associated with corresponding abnormal lab and/or pituitary scan | Delay dose | Dosing may resume if endocrinopathy resolves to be asymptomatic, or is adequately controlled with only physiologic hormone replacement. |
| | Grade 4 | Delay dose or permanently discontinue | Mandatory discussion with and approval from the Medical Monitor needed prior to resuming therapy. If endocrinopathy resolves or is adequately controlled with physiologic hormone replacement, participant may not require discontinuation of study drug. |
| Hyperthyroidism or Hypothyroidism | Grade 2 or 3 | Delay dose | Dosing may resume if endocrinopathy resolves to be asymptomatic, or is adequately controlled with only physiologic hormone replacement or other medical management. |
| | Grade 4 | Delay dose or permanently discontinue | Mandatory discussion with and approval from the Medical Monitor needed prior to resuming therapy. If endocrinopathy resolves or is adequately controlled with physiologic hormone replacement or other medical management, participant may not require discontinuation of study drug. |
| **Skin** | | | |
| Rash | Grade 2 rash covering > 30% body surface area or Grade 3 rash | Delay dose | Dosing may resume when rash reduces to ≤ 10% body surface area. |
| | Suspected Stevens-Johnson syndrome (SJS), toxic epidermal necrolysis | Delay dose | Dosing may resume if SJS, TEN, or DRESS is ruled out and rash reduces to ≤ 10% body surface area. |

**Table 7.4.1-1: Adverse Event Criteria to Delay, Resume, or Discontinue Study Treatment**

| Drug-Related Adverse Event (AE) per CTCAE V5 | Severity | Action Taken | Clarifications, Exceptions, and Resume Criteria |
|---|---|---|---|
| | (TEN) or drug reaction with eosinophilia and systemic symptoms (DRESS) | | |
| | Grade 4 rash or confirmed SJS, TEN, or DRESS | Permanently discontinue | |
| **Neurological** | | | |
| Guillain-Barre Syndrome (GBS) | Any Grade | Permanently discontinue | |
| Myasthenia Gravis (MG) | Any Grade | Permanently discontinue | |
| Encephalitis | Any Grade encephalitis | Delay dose | After workup for differential diagnosis, (ie, infection, tumor-related), if encephalitis is not drug related, then dosing may resume when AE resolves. |
| | Any Grade drug-related encephalitis | Permanently discontinue | |
| Myelitis | Any Grade myelitis | Delay dose | After workup for differential diagnosis, (ie, infection, tumor-related), if myelitis is not drug related, then dosing may resume when AE resolves. |
| | Any Grade drug-related myelitis | Permanently discontinue | |
| Neurological (other than GBS, MG, encephalitis, or myelitis) | Grade 2 | Delay dose | Dosing may resume when AE resolves to baseline. |
| | Grade 3 or 4 | Permanently discontinue | |
| Myocarditis | | | |
| Myocarditis | Symptoms induced from mild to moderate activity or exertion | Delay dose | Dosing may resume after myocarditis has resolved. |



Approved v1.0 930137673 4.0

**Table 7.4.1-1: Adverse Event Criteria to Delay, Resume, or Discontinue Study Treatment**

| Drug-Related Adverse Event (AE) per CTCAE V5 | Severity | Action Taken | Clarifications, Exceptions, and Resume Criteria |
|---|---|---|---|
| | Severe or life threatening, with symptoms at rest or with minimal activity or exertion, and/or where intervention indicated | Permanently discontinue | |
| **Other Clinical AE** | | | |
| Pancreatitis:<br>Amylase or Lipase Increased | Grade 3 with symptoms | Delay dose | Note: Grade 3 increased amylase or lipase without signs or symptoms of pancreatitis does not require dose delay.<br>Dosing may resume when patient becomes asymptomatic. |
| | Grade 4 | Permanently discontinue | |
| Uveitis | Grade 2 uveitis | Delay dose | Dosing may resume if uveitis responds to topical therapy (eye drops) and after uveitis resolves to Grade ≤ 1 or baseline. If patient requires oral steroids for uveitis, then permanently discontinue study drug. |
| | Grade 3 or 4 uveitis | Permanently discontinue | |
| Other Drug-Related AE (not listed above) | Grade 2 non-skin AE, except fatigue | Delay dose | Dosing may resume when AE resolves to Grade ≤ 1 or baseline value. |
| | Grade 3 AE - First occurrence lasting ≤ 7 days | Delay dose | Dosing may resume when AE resolves to Grade ≤ 1 or baseline value. |
| | Grade 3 AE - First occurrence lasting > 7 days | Permanently discontinue | |
| | Recurrence of Grade 3 AE of any duration | Permanently discontinue | |



Approved v4.0 930137673 4.0

**Table 7.4.1-1: Adverse Event Criteria to Delay, Resume, or Discontinue Study Treatment**

| Drug-Related Adverse Event (AE) per CTCAE V5 | Severity | Action Taken | Clarifications, Exceptions, and Resume Criteria |
|---|---|---|---|
| | Grade 4 or life-threatening adverse reaction | Permanently discontinue | |
| **Other Lab abnormalities** | | | |
| Other Drug-Related lab abnormality (not listed above) | Grade 3 | Delay dose | Exceptions:<br>No delay required for: Grade 3 lymphopenia.<br>Permanent discontinuation for: Grade 3 thrombocytopenia > 7 days or associated with bleeding. |
| | Grade 4 | Permanently discontinue | Exceptions: The following events do not require discontinuation of study drug:<br>• Grade 4 neutropenia ≤ 7 days.<br>• Grade 4 lymphopenia or leukopenia.<br>• Grade 4 isolated electrolyte imbalances/abnormalities that are not associated with clinical sequelae and are responding to supplementation/appropriate management within 72 hours of their onset. |
| **Infusion Reactions (manifested by fever, chills, rigors, headache, rash, pruritus, arthralgia, hypotension, hypertension, bronchospasm, or other allergic-like reactions.)** | | | |
| Hypersensitivity Reaction or Infusion Reaction | Grade 3 or 4 | Permanently discontinue | Refer to Section 7.4.2 on Treatment of Related Infusion Reactions. |

#### 7.4.1.1 Additional Dose Delay Criteria/Details

- Any AE, laboratory abnormality, or intercurrent illness which, in the judgment of the Investigator, warrants delaying the dose of study medication.
- SARS-CoV-2 infection either confirmed or suspected.

Participants who require delay of study drug administration should be re-evaluated weekly or more frequently if clinically indicated and resume dosing when re-treatment criteria are met.



Approved v1.0 930137673 4.0

#### 7.4.1.2 Additional Dose Resumption Criteria/Details

- Participants may resume treatment with study drug if they have completed AE management (ie, corticosteroid taper) or are on ≤ 10 mg prednisone or equivalent, and meet the requirements per Table 7.4.1-1.
- Participants with SARS-CoV-2 infection (either confirmed or suspected) may resume treatment after all of the following criteria are met:
- Symptomatic Participants: At least 10 days (20 days for severe/critical illness) have passed since symptoms first appeared. Asymptomatic participants: At least 10 days have passed since positive test result (eg, RT-PCR or viral antigen).
- Resolution of symptoms (including at least 24 hours has passed since last fever without fever-reducing medications).
- Evaluation by the Investigator with confirmation that there are no sequelae that would place the participant at a higher risk of receiving investigational treatment.
- Consultation by the BMS Medical Monitor or designee. For suspected cases, treatment may also resume if SARS-CoV-2 infection is ruled-out and other criteria to resume treatment are met.
- Prior to re-initiating on-study treatment in a participant with a dosing delay lasting > 8 weeks due to COVID-19, the BMS Medical Monitor or designee must be consulted (see Section 8.1).
- If treatment is withheld > 8 weeks from the last dose, the participant must be permanently discontinued from study therapy, except as specified in Section 7.4.1.3.

#### 7.4.1.3 Additional Dose Discontinuation Criteria/Details

- Any event that leads to delay in dosing lasting > 8 weeks from the previous dose requires discontinuation, with the following exceptions:
  - Dosing delays to allow for prolonged steroid tapers to manage drug-related AEs are allowed.
  - Dosing delays lasting > 8 weeks from the previous dose that occur for **non-drug-related reasons (including due to COVID-19)** may be allowed if approved by the BMS Medical Monitor or designee.
- Any AE, laboratory abnormality, or intercurrent illness which, in the judgment of the Investigator, presents a substantial clinical risk to the participant with continued nivolumab dosing, may require discontinuation.
- Prior to re-initiating treatment in a participant with a dosing delay lasting > 8 weeks, the BMS Medical Monitor or designee must be consulted. Tumor assessments should continue as per protocol even if dosing is delayed. Periodic study visits to assess safety and laboratory studies should also continue every 6 weeks or more frequently if clinically indicated during such dosing delays.
- Study treatment should be discontinued for disease recurrence (local, regional, or distant). Please refer to Section 7.8.1 for continuation of treatment beyond disease recurrence/progression exceptions.



Approved v4.0 930137673 4.0

Refer to the Schedule of Activities (Section 2) for data to be collected at the time of treatment discontinuation and follow-up and for any further evaluations that can be completed.

In the case of pregnancy, the investigator must immediately, within 24 hours of awareness of the pregnancy, notify the BMS Medical Monitor or designee of this event. In most cases, the study treatment will be permanently discontinued in an appropriate manner (eg, dose tapering if necessary, for participant safety). Refer to Section 9.2.5, Pregnancy.

All participants who discontinue study treatment should comply with protocol-specified follow-up procedures as outlined in Section 2. The only exception to this requirement is when a participant withdraws consent for all study procedures including post-treatment study follow-up or loses the ability to consent freely (ie, is imprisoned or involuntarily incarcerated for the treatment of either a psychiatric or physical illness).

If study treatment is discontinued prior to the participant's completion of the study, the reason for the discontinuation must be documented in the participant's medical records and entered on the appropriate case report form (CRF) page.

Please refer to Section 8.1 for full details of dose discontinuation criteria not covered here.

### 7.4.2 Treatment of Nivolumab-Related Infusion Reactions

Since nivolumab contains only human immunoglobulin protein sequences, it is unlikely to be immunogenic and induce infusion or hypersensitivity reactions. However, if such a reaction were to occur, it might manifest with fever, chills, rigors, headache, rash, pruritus, arthralgia, hypo- or hypertension, bronchospasm, or other symptoms. All Grade 3 or 4 infusion reactions should be reported within 24 hours as an SAE if criteria are met.

Treatment recommendations are provided below based on CTCAE v5 grading definitions and may be modified based on local treatment standards and guidelines, as appropriate:

For Grade 1 symptoms: (mild reaction; infusion interruption not indicated; intervention not indicated)

- Remain at bedside and monitor participant until recovery from symptoms. The following prophylactic premedications are recommended for future infusions: diphenhydramine 50 mg (or equivalent) and/or acetaminophen/paracetamol 325 to 1000 mg at least 30 minutes before additional nivolumab administrations.

For Grade 2 symptoms: (moderate reaction; Therapy or infusion interruption indicated but responds promptly to symptomatic treatment [eg, antihistamines, non-steroidal anti-inflammatory drugs, narcotics, corticosteroids, bronchodilators, IV fluids]; prophylactic medications indicated for ≤ 24 hours).

- Stop the nivolumab infusion, begin an IV infusion of normal saline, and treat the participant with diphenhydramine 50 mg IV (or equivalent) and/or acetaminophen/paracetamol 325 to 1000 mg; remain at bedside and monitor participant until resolution of symptoms. Corticosteroid and/or bronchodilator therapy may also be administered as appropriate. If the infusion is interrupted, then restart the infusion at 50% of the original infusion rate when



Approved v4.0 930137673 4.0

symptoms resolve; if no further complications ensue after 30 minutes, the rate may be increased to 100% of the original infusion rate. (Note: If the infusion rate cannot be safely increased to the original infusion rate, the reduced infusion rate of 50% of the original infusion rate, or a reduced rate as determined per local standard, can be adopted for future infusions). Monitor participant closely. If symptoms recur, then no further nivolumab will be administered at that visit. Administer diphenhydramine 50 mg IV, and remain at bedside and monitor the participant until resolution of symptoms. The amount of study drug infused must be recorded on the CRF.

- For future infusions, the following prophylactic premedications are recommended: diphenhydramine 50 mg (or equivalent) and/or acetaminophen/paracetamol 325 to 1000 mg should be administered at least 30 minutes before nivolumab. If necessary, corticosteroids (up to 25 mg of IV hydrocortisone [SoluCortef] or equivalent) may be used.

For Grade 3 or 4 symptoms: (severe reaction, Grade 3: prolonged [ie, not rapidly responsive to symptomatic medication and/or brief interruption of infusion]; recurrence of symptoms following initial improvement; hospitalization indicated for other clinical sequelae [eg, renal impairment, pulmonary infiltrates]. Grade 4: Life-threatening consequences; urgent intervention indicated).

- Immediately discontinue infusion of nivolumab. Begin an IV infusion of normal saline and treat the participant as follows: Recommend bronchodilators, epinephrine 0.2 to 1 mg of a 1:1000 solution for subcutaneous administration or 0.1 to 0.25 mg of a 1:10,000 solution injected slowly for IV administration, and/or diphenhydramine 50 mg IV with methylprednisolone 100 mg IV (or equivalent), as needed. Participant should be monitored until the investigator is comfortable that the symptoms will not recur. Nivolumab will be permanently discontinued. Investigators should follow their institutional guidelines for the treatment of anaphylaxis. Remain at bedside and monitor participant until recovery of the symptoms.

In case of late-occurring hypersensitivity symptoms (eg, appearance of a localized or generalized pruritus within 1 week after treatment), symptomatic treatment may be given (eg, oral antihistamine or corticosteroids).

## 7.5 Preparation/Handling/Storage/Accountability

The investigational product should be stored in a secure area according to local regulations. It is the responsibility of the investigator to ensure that investigational product is only dispensed to study participants. The investigational product must be dispensed only from official study sites by authorized personnel according to local regulations.

The product storage manager should ensure that the study treatment is stored in accordance with the environmental conditions (temperature, light, and humidity) as determined by BMS. If concerns regarding the quality or appearance of the study treatment arise, the study treatment should not be dispensed, and the unblinded BMS Clinical Trial Monitor should be contacted immediately.



Approved v4.0 930137673 4.0

Study treatment not supplied by BMS will be stored in accordance with the package insert.

Investigational product documentation (whether supplied by BMS or not) must be maintained that includes all processes required to ensure drug is accurately administered. This includes documentation of drug storage, administration and, as applicable, storage temperatures, reconstitution, and use of required processes (eg, required diluents, administration sets).

Further guidance and information for final disposition of unused study treatment are provided in Appendix 2.

Please refer to the current version of the Investigator Brochure and/or pharmacy manual for complete preparation, storage, and handling information for nivolumab.

### *7.5.1 Retained Samples for Bioavailability / Bioequivalence / Biocomparability*

Not applicable.

## 7.6 Treatment Compliance

Study treatment compliance will be periodically monitored by drug accountability as well as the participant's medical record and CRF. Study drug will be administered in the clinic by trained personnel.

## 7.7 Concomitant Therapy

Non-live COVID-19 vaccination is considered a simple concomitant medication within the study. However, the efficacy and safety of non-live vaccines (including non-live COVID-19 vaccines) in participants receiving nivolumab is unknown.

### *7.7.1 Prohibited and/or Restricted Treatments*

The following medications are prohibited during the study (unless utilized to treat a drug-related adverse event):

- Immunosuppressive agents
- Immunosuppressive doses of systemic corticosteroids > 10 mg daily prednisone equivalent (except as stated in Section 6.2- Exclusion Criteria) only until treatment discontinuation. See Section 7.7.4 for permitted corticosteroid therapy.
- Any additional, concurrent anti-neoplastic therapy (ie, chemotherapy, hormonal therapy, immunotherapy, extensive non-palliative radiation therapy, or standard or investigational agents for treatment of melanoma) outside of study treatment. Participants who develop a new non-melanoma fully resectable malignancy (examples include but are not limited to: in situ bladder cancer, in situ gastric cancer, or in situ colon cancers; in situ cervical cancers/dysplasia; or breast carcinoma in situ; or prostate carcinoma) during the study may continue receiving study drugs if the only therapy required is hormonal therapy, surgery and/or radiation (and the surgery or radiation site does not overlap with a previous primary melanoma or melanoma metastasis location). Consultation with the Medical Monitor or designee is required once a new malignancy is detected.
- Any complementary medications (eg, herbal supplements or traditional Chinese medicines) intended to treat the disease under study. Such medications are permitted if they are used as



Approved v1.0 930137673 4.0

supportive care. Use of marijuana and its derivatives for treatment of symptoms related to cancer or cancer treatment are permitted if obtained by medical prescription or if its use (even without a medical prescription) has been legalized locally.

- Consultation with the Medical Monitor or designee is required prior to the use of topical Toll-like receptor 7 (TLR7) agonists (eg, imiquimod), calcineurin inhibitors (eg, tacrolimus), or topical immunotherapy/contact sensitizer preparations, eg, but not limited to 2,4-dinitrochlorobenzene (DNCB), squaric acid dibutylester (SADBE), and diphencyprone (DPCP) at or near the primary tumor site.
- Participants who, in the assessment by the investigator, require the use of any of the aforementioned treatments for clinical management should be removed from the study.
- Participants may receive other medications that the investigator deems to be medically necessary.
- Any live / attenuated vaccine (eg, varicella, zoster, yellow fever, rotavirus, oral polio and measles, mumps, rubella [MMR]) during treatment and until 100 days post last dose.

### 7.7.2 Other Restrictions and Precautions

Participants with a condition requiring systemic treatment with either corticosteroids (> 10 mg daily prednisone equivalent) or other immunosuppressive medications within 14 days of randomization are excluded. Inhaled or topical steroids, and adrenal replacement steroid doses greater than 10 mg daily prednisone equivalent, are permitted in the absence of active autoimmune disease.

### 7.7.3 Imaging Restriction and Precautions

It is the local imaging facility's responsibility to determine, based on participant attributes (eg, allergy history, diabetic history, and renal status), the appropriate imaging modality and contrast regimen per imaging study. Imaging contraindications and contrast risks are to be considered in this assessment. Participants with renal insufficiency are to be assessed as to whether or not they should receive contrast and if so, which contrast agent and dose is appropriate. Specific to MRI, participants with severe renal insufficiency (ie, estimated glomerular filtration rate (eGFR) < 30 mL/min/1.73 $m^2$) are at increased risk of nephrogenic systemic fibrosis, therefore MRI contrast is contraindicated. In addition, participants may be excluded from MRI if they have tattoos, metallic implants, pacemakers, etc. This will be outlined in the image acquisition manual.

Gentle hydration before and after IV contrast should follow local standard of care. The ultimate decision to perform MRI in an individual participant in this study rests with the site radiologist, the investigator, and standards set by the local Ethics Committee.

### 7.7.4 Permitted Therapy

Participants are permitted the use of topical, ocular, intra-articular, intranasal, and inhalational corticosteroids (with minimal systemic absorption). Adrenal replacement steroid doses > 10 mg daily prednisone are permitted. A brief (less than 3 weeks) course of corticosteroids for prophylaxis (eg, contrast dye allergy) or for treatment of non-autoimmune conditions (eg, delayed-type hypersensitivity reaction caused by a contact allergen) is permitted.



Approved v4.0 930137673 4.0

## 7.8 Treatment After the End of the Study

At the end of the study, BMS will not continue to provide BMS supplied study treatment to participants/investigators unless BMS chooses to extend the study. The investigator should ensure that the participant receives appropriate standard of care to treat the condition under study. BMS reserves the right to terminate access to BMS supplied study treatment if any of the following occur: a) the study is terminated due to safety concerns; b) the development of nivolumab is terminated for other reasons, including but not limited to lack of efficacy and/or not meeting the study objectives; c) the participant can obtain medication from a government sponsored or private health program. In all cases BMS will follow local regulations.

### *7.8.1 Treatment Beyond Disease Progression*

#### *7.8.1.1 Participants Receiving Optional Open-Label Nivolumab with Unresectable/Metastatic Disease (Arm 2)*

Accumulating evidence indicates a minority of participants treated with immunotherapy may derive clinical benefit despite initial evidence of Progressive Disease (PD). Participants will be permitted to continue treatment beyond initial RECIST 1.1 defined PD (Appendix 7), up to a maximum of 2 years treatment, as long as they meet the following criteria:

- Investigator-assessed clinical benefit
- Participant is tolerating study drug
- Stable performance status
- Treatment beyond progression will not delay an imminent intervention to prevent serious complications of disease progression (eg, CNS metastases)
- Participant provides written informed consent prior to receiving additional study medication. All other elements of the main informed consent including the description of reasonably foreseeable risks or discomforts, or other alternative treatment options will still apply.

A radiographic assessment/scan should be performed within 6 weeks of initial investigator-assessed progression to determine whether there has been a decrease in the tumor size or continued PD. The assessment of clinical benefit should be balanced by clinical judgment as to whether the participant is clinically deteriorating and unlikely to receive any benefit from continued treatment with nivolumab.

If the investigator feels that the participant continues to achieve clinical benefit by continuing treatment, the participant should remain on the trial and continue to receive monitoring according to the Schedule of Activities in Section 2.

For the participants who continue study therapy beyond progression, further progression is defined as an additional 10% increase in tumor burden with a minimum of 5 mm absolute increase from time of initial PD. This includes an increase in the sum of diameters of all target lesions and/ or the diameters of new measurable lesions compared to the time of initial PD. Treatment with study medication should be discontinued permanently upon documentation of further progression.



Approved v1.0 930137673 4.0

New lesions are considered measureable at the time of initial progression if the longest diameter is at least 10 mm (except for pathological lymph nodes which must have a short axis of at least 15 mm). Any new lesion considered non-measureable at the time of initial progression may become measureable and therefore included in the tumor burden if the longest diameter increases to at least 10 mm (except for pathological lymph nodes which must have a short axis of at least 15 mm). In situations where the relative increase in total tumor burden by 10% is solely due to inclusion of new lesions which become measurable, these new lesions must demonstrate an absolute increase of at least 5 mm.

# 8 DISCONTINUATION CRITERIA

## 8.1 Discontinuation from Study Treatment

Please refer to Table 7.4.1-1 for details regarding discontinuation of nivolumab or nivolumab-placebo.

Participants MUST discontinue investigational product (and non-investigational product at the discretion of the investigator) for any of the following reasons:

- Disease recurrence (local, regional, or distant). Please refer to Section 7.7.1 for continuation of treatment beyond disease recurrence/progression exceptions.
- Participant's request to stop study treatment. Participants who request to discontinue study treatment will remain in the study and must continue to be followed for protocol specified follow-up procedures. The only exception to this is when a participant specifically withdraws consent for any further contact with him/her or persons previously authorized by participant to provide this information.
- Any clinical adverse event (AE), laboratory abnormality or intercurrent illness which, in the opinion of the investigator, indicates that continued participation in the study is not in the best interest of the participant.
- Termination of the study by Bristol-Myers Squibb (BMS).
- Loss of ability to freely provide consent through imprisonment or involuntarily incarceration for treatment of either a psychiatric or physical (eg, infectious disease) illness. (Note: Under specific circumstances, and only in countries where local regulations permit, a participant who has been imprisoned may be permitted to continue as a participant. Strict conditions apply and BMS approval is required) .

In the case of pregnancy, the Investigator must immediately notify the Sponsor or designee of this event. In most cases, the study drug will be permanently discontinued in an appropriate manner (eg, dose tapering if necessary for participant safety). Please contact the Sponsor or designee within 24 hours of awareness of the pregnancy. If the Investigator determines a possible favorable benefit/risk ratio that warrants continuation of study drug, a discussion between the investigator and the Sponsor or designee must occur. All participants who discontinue study treatment should comply with protocol specified follow-up procedures as outlined in Section 2. The only exception to this requirement is when a participant withdraws consent for all study procedures including post-treatment study follow-up or loses the ability to consent freely (ie, is imprisoned or involuntarily incarcerated for the treatment of either a psychiatric or physical illness).



Approved v4.0 930137673 4.0

If study treatment is discontinued prior to the participant's completion of the study, the reason for the discontinuation must be documented in the participant's medical records and entered on the appropriate case report form (CRF) page.

#### *8.1.1 Post Study Treatment Study Follow-up*

In this study, RFS and OS are key endpoints of the study. Post study follow-up is of critical importance and is essential to preserving participant safety and the integrity of the study. Participants who discontinue study treatment must continue to be followed (**in this study or a rollover study**) for collection of tumor surveillance assessments and survival follow-up data as required and in line with Section 2 and Section 5 until the OS final analysis.

BMS may request that survival data be collected on all treated/randomized participants outside of the protocol-defined window (see Schedule of Activities - Section 2). At the time of this request, each participant will be contacted to determine their survival status unless the participant has withdrawn consent for all contacts or is lost to follow-up.

Participants who discontinue study treatment may continue to be followed.

### 8.2 Discontinuation from the Study

Participants who request to discontinue study treatment will remain in the study and must continue to be followed for protocol specified follow-up procedures. The only exception to this is when a participant specifically withdraws consent for any further contact with him/her or persons previously authorized by participant to provide this information.

- Participants should notify the investigator of the decision to withdraw consent from future follow-up **in writing**, whenever possible.
- The withdrawal of consent should be explained in detail in the medical records by the investigator, as to whether the withdrawal is from further treatment with study treatment only or also from study procedures and/or post treatment study follow-up, and entered on the appropriate CRF page.
- In the event that vital status (whether the participant is alive or dead) is being measured, publicly available information should be used to determine vital status only as appropriately directed in accordance with local law.
- If the participant withdraws consent for disclosure of future information, the sponsor may retain and continue to use any data collected before such a withdrawal of consent.

### 8.3 Lost to Follow-Up

- All reasonable efforts must be made to locate participants to determine and report their ongoing status. This includes follow-up with persons authorized by the participant.
- Lost to follow-up is defined by the inability to reach the participant after a minimum of **three** documented phone calls, faxes, or emails as well as lack of response by participant to one registered mail letter. All attempts should be documented in the participant's medical records.
- If it is determined that the participant has died, the site will use permissible local methods to obtain date and cause of death.



Approved v1.0 930137673 4.0

- If investigator's use of third-party representative to assist in the follow-up portion of the study has been included in the participant's informed consent, then the investigator may use a Sponsor retained third-party representative to assist site staff with obtaining participant's contact information or other public vital status data necessary to complete the follow-up portion of the study.
- The site staff and representative will consult publicly available sources, such as public health registries and databases, in order to obtain updated contact information.
- If after all attempts, the participant remains lost to follow-up, then the last known alive date as determined by the investigator should be reported and documented in the participant's medical records.

# 9 STUDY ASSESSMENTS AND PROCEDURES

Study procedures and timing are summarized in the Schedule of Activities (Section 2).

- Protocol waivers or exemptions are not allowed.
- All immediate safety concerns must be discussed with the Sponsor immediately upon occurrence or awareness to determine if the participant should continue or discontinue treatment.
- Adherence to the study design requirements, including those specified in the Schedule of Activities, is essential and required for study conduct.
- All screening evaluations must be completed and reviewed to confirm that potential participants meet all eligibility criteria before randomization. The investigator will maintain a screening log to record details of all participants screened and to confirm eligibility or record reasons for screening failure, as applicable.
- Procedures conducted as part of the participant's routine clinical management (eg, blood count) and obtained before signing of informed consent may be utilized for screening or baseline purposes provided the procedure meets the protocol-defined criteria and has been performed within the timeframe defined in the Schedule of Activities.
- Additional measures, including non-study required laboratory tests, should be performed as clinically indicated or to comply with local regulations. Laboratory toxicities (eg, suspected drug induced liver enzyme evaluations) will be monitored during the follow-up phase via on-site/local labs until all study drug-related toxicities resolve, return to baseline, or are deemed irreversible.
- If a participant shows pulmonary-related signs (hypoxia, fever) or symptoms (eg. dyspnea, cough, fever) consistent with possible pulmonary adverse events, the participant should be immediately evaluated to rule out pulmonary toxicity, according to the suspected pulmonary toxicity management algorithm in the BMS-936558 (nivolumab) Investigator Brochure.
- Some of the assessments referred to in this section may not be captured as data in the CRF. They are intended to be used as safety monitoring by the treating physician. Additional testing or assessments may be performed as clinically necessary or where required by institutional or local regulations.



Approved v1.0 930137673 4.0

## 9.1 Efficacy Assessments

### *9.1.1 Imaging Assessment for the Study*

Images will be submitted to a central imaging vendor and may undergo blinded independent central review (BICR) at any time during the study. Prior to scanning first participant, sites should be qualified and understand the image acquisition guidelines and submission process as outlined in the CA20976K Imaging Manual provided by the central imaging vendor.

Screening and on study images should be acquired as outlined in Section 2, Schedule of Activities.

Tumor assessments at other timepoints may be performed if clinically indicated and should be submitted to the central imaging vendor as soon as possible. Unscheduled CT/MRI should be submitted to central imaging vendor. X-rays and bone scans that clearly demonstrate interval progression of disease, for example most commonly as unequivocal lesions that are unmistakably new since the prior CT/MRI, should be submitted to central imaging vendor. Otherwise, they do not need to be submitted centrally.

#### *9.1.1.1 Methods of Measurement*

Contrast-enhanced CT of the chest, abdomen, pelvis, and all other known and/or suspected sites of disease (imaging of extremities for resected melanomas located in the extremities is not a requirement and may be conducted per local standard of care. Extremity imaging should be conducted in the event of a suspected locoregional relapse that is not clinically unequivocal) should be performed for tumor assessments. Images should be acquired with slice thickness of 5 mm or less with no intervening gap (contiguous). Every attempt should be made to image each participant using an identical acquisition protocol on the same scanner for all imaging timepoints.

If a participant has a contraindication for CT intravenous contrast, then a non-contrast CT of the chest and a contrast-enhanced MRI of the abdomen, pelvis, and other known/suspected sites of disease should be obtained.

If a participant has a contraindication for both MRI and CT intravenous contrasts, then a non-contrast CT of the chest and a non-contrast MRI of the abdomen, pelvis, and other known/suspected sites of disease should be obtained.

If a participant has a contraindication for MRI (eg, incompatible pacemaker) in addition to contraindication to CT intravenous contrast, then a non-contrast CT of the chest, abdomen, pelvis, and other known/suspected sites of disease is acceptable.

Use of CT component of a PET-CT scanner: Combined modality scanning such as with PET CT is increasingly used in clinical care, and is a modality/technology that is in rapid evolution; therefore, the recommendations outlined here may change rather quickly with time. At present, low dose or attenuation correction CT portions of a combined PET-CT are of limited use in anatomically-based efficacy assessments and it is therefore suggested that they should not be substituted for dedicated diagnostic contrast enhanced CT scans. However, if a site can document that the CT performed as part of a PET-CT is of identical diagnostic quality to a diagnostic CT (with IV and oral contrast), then the CT portion of the PET-CT can be used. Note, however, that



Approved v4.0 930137673 4.0

the PET portion of the CT introduces additional data which may bias an investigator if it is not routinely or serially performed.

Bone scans may be collected per local standards, as clinically indicated.

MRI of brain (with and without contrast) should be acquired as outlined in the Schedule of Activities for Arm 2 Participants. CT of the Brain (without and with contrast) can be performed if MRI is contraindicated.

***For Arm 2 (Unresectable/Metastatic Disease) only:***

Tumor measurements should be made by the same investigator or radiologist for each assessment, whenever possible. Changes in tumor measurements and tumor response to guide ongoing study treatment decisions will be assessed by the investigator using the RECIST v1.1 criteria (Appendix 7).

Bone scan or PET scan are not adequate for assessment of RECIST 1.1 response in target lesions. In selected circumstances where such modalities are the sole modality used to assess certain nontarget organs, those non-target organs may be evaluated less frequently. For example, bone scans may need to be repeated only when complete response is identified in target disease or when progression in bone is suspected.

### 9.1.2 Investigator Assessment of Baseline Disease Status

Participant eligibility must be confirmed by investigator prior to randomization.

#### 9.1.2.1 General Considerations for Determining Recurrence

Cytology and/or histology are mandatory to confirm recurrence in solitary or in doubtful lesions, cutaneous, subcutaneous, or lymph node lesions. Histological or cytological evidence of recurrence should be attempted in all cases except when the biopsy poses significant risks to the participant and/or may not be clinically feasible. An example when obtaining a biopsy to confirm recurrence may not be safe and clinically feasible is brain metastases.

- Clinically detected new lesions:
  - Superficial cutaneous lesions: the neoplastic nature must be confirmed by cytology/histology.
  - Deep subcutaneous lesions and lymph node lesions should be documented by ultrasound and histological/cytological evidence should be attempted. In absence of a pathology report, lesion recurrence will be documented with a CT scan/MRI.
- Tumor markers or auto-antibodies alone cannot be used to assess recurrence.
- The goal is to identify lesions suspicious for recurrence of melanoma. If it is believed that a lesion is NOT malignant in nature (eg, infection, trauma), it should be noted in the medical records. The specified minimum size criteria should be combined with radiographic appearance consistent with recurrent tumor in the assessment of all suspicious lesions.
- Non-melanoma primary cancer will be censored.
- Equivocal recurrence is upgraded to unequivocal recurrence (except in cases of central nervous system [CNS] recurrence) by one or more of the following:



Approved v4.0 930137673 4.0

  - A subsequent scan not earlier than 4 weeks from the time when recurrence was first suspected demonstrates that the lesion size is ≥ 5 mm over the size previously recorded, or the radiographic appearance of the lesion has become consistent with tumor recurrence. If this occurs, the date of recurrence will be the date when the lesion was first suspected.
  - Positive histology/cytology
- Appearance of multiple new lesions in the same time point generally constitutes unequivocal recurrence, even though they may be from different organs (eg, one liver lesion, one lung lesion, and one enlarged lymph node).

#### 9.1.2.2 Investigator Assessment of Recurrence (Nivolumab/Placebo-Blinded Treatment and Open-Label Nivolumab - Arm 1)

The same method of assessment used at Screening should be used for on-study timepoints. Post-baseline assessments will be performed at the time points described below until disease recurrence confirmed by Investigator, death, or withdrawal from the study.

- Tumor assessments for ongoing study treatment decisions will be completed by the Investigator.
- Additional imaging of potential disease sites should be performed whenever disease recurrence or occurrence of a secondary malignancy is suspected.
- Brain CT or MRI with and without contrast or bone imaging during on-study treatment and follow-up periods should be obtained if clinically indicated.

Criteria for Diagnosing Recurrences is presented in Table 9.1.2.2-1.

**Table 9.1.2.2-1: Criteria For Diagnosing Recurrence**

| Anatomic Sites | Criteria |
|---|---|
| Non-Nodal Soft Tissue | Equivocal lesions include:<br>• Solitary lesion measuring ≤ 10 mm in LD or with radiographic appearance equivocal for tumor recurrence.<br>Unequivocal lesions include:<br>• One or more new lesions > 10 mm in LD with radiographic appearance consistent with tumor recurrence.<br>• Positive histology/cytology |
| Bone Lesions | Equivocal lesions include:<br>• Solitary lesion.<br>• Lesions identified on radionuclide bone scan. Findings on radionuclide bone scan must be confirmed by CT, MRI, or plain films in order to be upgraded to unequivocal.<br>Unequivocal lesions include:<br>• Two or more new lesions consistent with tumor recurrence.<br>• Positive histology/cytology |
| Lymph Nodes[a] | Normal lymph nodes are defined as < 10 mm in the short-axis diameter (SAD). |



Approved v1.0 930137673 4.0

**Table 9.1.2.2-1: Criteria For Diagnosing Recurrence**

| Anatomic Sites | Criteria |
|---|---|
| | Equivocal lymph nodes include<br>• Lymph nodes measuring 10 – 14 mm SAD with radiographic appearance consistent with recurrence.<br>• Lymph nodes ≥ 15 mm SAD without radiographic appearance consistent with recurrence.<br>Unequivocal proof of nodal recurrence includes:<br>• One or more previously normal or equivocal lymph nodes that enlarge to ≥ 15 mm SAD and with radiographic appearance consistent with recurrence.<br>• Positive histology/cytology |
| Fluid Collections<br>(eg, ascites, pleural/pericardial effusions) | • Presence of fluid alone, without pathological confirmation, does not constitute equivocal or unequivocal recurrence.<br>• Unequivocal proof of recurrence is positive pathology of malignant cells from fluid(s). |
| CNS | Unequivocal recurrence is defined as any new CNS lesion of any size on CT or MRI with a radiographic appearance consistent with tumor recurrence. |

[a] For lymph node with short axis 10-15 mm, consider biopsy when lymph node is progressively enlarged as evidenced.

### *9.1.2.3 Definitions*

Recurrence is defined as the appearance of one or more new melanoma lesions, which can be local, regional, or distant in location from the primary resected site.

**Local Cutaneous Recurrence:**

Local cutaneous recurrence after adequate excision of the primary melanoma is associated with aggressive tumor biologic features and is frequently a harbinger of metastases.

**Regional Lymphatic and Nodal Recurrences:**

The neoplastic nature of the regional recurrences should be attempted and confirmed by histology/cytology.

**In Transit Metastases:**

In transit metastases represents the clinical manifestations of small tumor emboli trapped within the dermis and subdermal lymphatics between the site of the primary tumor and the regional lymph node drainage basin(s). In extremities, in transit metastases can also occur distal to the site of the primary lesion as a result of reversed lymphatic flow. In transit metastases occur in 10% to 15% in patients with Stage III disease. Although previous staging systems distinguished between the small satellitosis (within 2 cm of the primary tumor), pathophysiologically, these two events represent different points on a continuum of the same biologic process. When present, in transit metastases are usually multiple, evolve over time, and, as previously stated, are often the harbinger of subsequent systemic disease.



Approved v1.0 930137673 4.0

**Regional Node Recurrences:**

Regional node failure in a previously dissected basin is usually found at the periphery of the prior surgical procedure.

**Patterns of Metastases:**

Melanoma is well-known for its ability to metastasize to virtually any organ or tissue. The most common initial sites of distance metastases are the non-visceral (skin, subcutaneous tissue, and lymph nodes), which are recurrence sites for 42% to 59% of participants in various studies. Visceral locations are the lung, brain, liver, gastrointestinal tract, and bone; the visceral sites are the initial sites of relapse in approximately 25% of all patients diagnosed with melanoma who experience recurrence.

#### *9.1.2.4 Date of Recurrence*

The first date when recurrence was observed is taken into account regardless of the method of assessment. Therefore, recurrence will be declared for any lesion when:

- Only imaging was performed and recurrence confirmed.
- Only pathology was done and malignancy confirmed (in solitary or in doubtful lesions, cutaneous, subcutaneous, or lymph node lesions).
- Both pathology and imaging were done and recurrence/malignancy confirmed. In this case, the date of whichever examination comes first is considered the date of recurrence.

Pathology reports of biopsies confirming recurrence should be sent to a central vendor.

Note: For documentation, the date of recurrence is the date that the pathology and/or imaging confirms recurrence, not the date that the information was communicated to the participant.

#### *9.1.2.5 Investigator Assessment of Progression for Participants Receiving Open-Label Nivolumab (Arm 2: Unresectable/Metastatic Disease)*

Tumor assessments should continue even if dosing is delayed or discontinued. Changes in tumor measurements and tumor responses will be assessed as per the protocol specified schedule by the same investigator or designee using RECIST 1.1 criteria. Investigators will report the number and size of new lesions that appear while on study. The time points of tumor assessments will be reported on the CRF based on the investigator's assessment using RECIST 1.1 criteria (See Appendix 7 for specifics of RECIST 1.1 criteria to be used in this study). Assessments of PR and CR must be confirmed at least 4 weeks (28 days) after initial response. A BOR of SD requires a minimum of 78 days on study (date of first dose to the earliest date outlined in the Schedule of Activities for the first imaging assessment).

[redacted]



Approved v4.0 930137673 4.0

[redacted]



Approved v4.0 930137673 4.0

[redacted]

## 9.2 Adverse Events

The definitions of an AE or serious adverse event (SAE) can be found in Appendix 3.

AEs will be reported by the participant (or, when appropriate, by a caregiver, surrogate, or the participant's legally authorized representative).

The investigator and any designees are responsible for detecting, documenting, and reporting events that meet the definition of an AE or SAE and remain responsible for following up AEs that are serious, considered related to the study treatment or the study, or that caused the participant to discontinue before completing the study.

**Contacts for SAE reporting specified in Appendix 3.**

Immune-mediated adverse events are AEs consistent with an immune-mediated mechanism or immune-mediated component for which non-inflammatory etiologies (eg, infection or tumor progression) have been ruled out. IMAEs can include events with an alternate etiology which were exacerbated by the induction of autoimmunity. Information supporting the assessment will be collected on the participant's case report form.

### *9.2.1 Time Period and Frequency for Collecting AE and SAE Information*

Sections 5.6.1 and 5.6.2 in the Investigator Brochure (IB) represent the Reference Safety Information to determine expectedness of serious adverse events for expedited reporting.

All AEs/SAEs are to be graded and managed per CTCAE v5.

**(A) For Participants with Stage IIB/C Melanoma (Nivolumab or Placebo-blinded Portion)**

For participants in the blinded portion of the trial, all AEs (serious and nonserious) will be collected for a minimum of 15 months (including Follow-Up Visits 1 and 2) following discontinuation of study treatment. The details are as follows:

**Nonserious AEs:** All nonserious AEs (not only those deemed to be treatment-related) must be collected continuously during the treatment period, starting from the initiation of study treatment, and until 100 days from the last dose of blinded study treatment. Beyond 100 days of discontinuation of blinded study treatment, only new-onset treatment-related nonserious AEs need to be reported. These events will continue to be collected for a minimum of 15 months (including Follow-Up Visits 1 and 2) following discontinuation of blinded study treatment. Nonserious



Approved v4.0 930137673 4.0

treatment-related AEs with an onset date beyond 100 days of discontinuation of blinded study treatment do not need to be reported, if the non-serious treatment-related AE has an onset date on or after the initiation of subsequent anti-cancer therapy.

**SAEs:** All SAEs (not only those deemed to be treatment-related) must be collected continuously during the treatment period, starting from the time of signing the consent, including those thought to be associated with protocol-specified procedures, and until 100 days from the last dose of blinded study treatment. Beyond 100 days of discontinuation of blinded study treatment, only new-onset treatment-related SAEs need to be reported. These events will continue to be collected for a minimum of 15 months (including Follow-Up Visits 1 and 2) following discontinuation of blinded study treatment. Serious blinded study treatment-related or study procedure-related AEs with an onset date beyond 100 days of discontinuation of blinded study treatment must be reported, if the onset of the serious study treatment/procedure-related AE has an onset date on or after the initiation of subsequent anti-cancer therapy.

**Serious and nonserious AEs (SARS-CoV-2-associated):** All serious and nonserious SARS-CoV-2 infection (confirmed or suspected)-associated AEs must be collected from the time of signing the consent until 100 days following discontinuation of dosing.

**(B) For Participants in the Open-Label Portion**

**Nonserious AEs:** All nonserious AEs (not only those deemed to be treatment-related) must be collected continuously during the treatment period, starting from the initiation of study treatment, and until 100 days from the last dose of study treatment. Beyond 100 days of discontinuation of dosing, new-onset nonserious AEs do not need to be reported.

**SAEs:** All SAEs (not only those deemed to be treatment-related) must be collected from the time of signing the open-label consent, including those thought to be associated with protocol-specified procedures and within 100 days of discontinuation of dosing. Beyond 100 days of discontinuation of dosing, new-onset SAEs do not need to be reported if subsequent anti-cancer therapy is initiated, unless the event is felt to be related to the CA20976K study treatment.

**Serious and nonserious AEs (SARS-CoV-2-associated):** All serious and nonserious SARS-CoV-2 infection (confirmed or suspected)-associated AEs need to be collected from the time of signing the consent until 100 days following discontinuation of dosing.

For participants randomized and never treated with study drug, SAEs should be collected for 30 days from the date of randomization.

**The Investigator must report any SAE that occurs after these time periods and that is believed to be related to study drug or protocol-specified procedure**, (eg, a follow-up skin biopsy).

- Medical occurrences that begin before the start of study treatment but after obtaining informed consent will be recorded on the appropriate section of the CRF module.
- All SAEs will be recorded and reported to Sponsor or designee within 24 hours, as indicated in Appendix 3.



Approved v4.0 930137673 4.0

- The Investigator will submit any updated SAE data to the Sponsor or designee within 24 hours of updated information being available.

Investigators are not obligated to actively seek AEs or SAEs in former study participants. However, if the Investigator learns of any SAE, including a death, at any time after a participant has been discharged from the study, and he/she considers the event reasonably related to the study treatment or study participation, the investigator must promptly notify the Sponsor.

The method of evaluating and assessing AE and SAE causalities and the procedures for completing, reporting, and/or transmitting SAE reports are provided in Appendix 3.

### 9.2.2 Method of Detecting AEs and SAEs

AEs can be spontaneously reported or elicited during open-ended questioning, examination, or evaluation of a participant. Care should be taken not to introduce bias when collecting AE and/or SAEs. Inquiry about specific AEs should be guided by clinical judgement in the context of known AEs, when appropriate for the program or protocol.

Every AE must be assessed by the Investigator with regard to whether it is considered immune-mediated. For events which are potentially immune-mediated, additional information will be collected on the participant's case report form.

### 9.2.3 Follow-up of AEs and SAEs

- Nonserious AEs (including confirmed or suspected SARS-CoV-2 infection-associated nonserious AEs) should be followed to resolution or stabilization, or reported as SAEs if they become serious (see Appendix 3).
- Follow-up is also required for nonserious AEs (including confirmed or suspected SARS-CoV-2 infection-associated nonserious AEs) that cause interruption or discontinuation of study treatment and for those present at the end of study treatment as appropriate.
- All identified nonserious AEs (including confirmed or suspected SARS-CoV-2 infection-associated nonserious AEs) must be recorded and described on the nonserious AE page of the CRF (paper or electronic). Completion of supplemental CRFs may be requested for AEs and/or laboratory abnormalities that are reported/identified during the course of the study.

All SAEs (including confirmed or suspected SARS-CoV-2 associated SAEs) will be followed until resolution, until the condition stabilizes, until the event is otherwise explained, or until the participant is lost to follow-up (as defined in Section 8.3), or for suspected cases, until SARS-CoV-2 infection is ruled-out based on additional medical evaluation.

Further information on follow-up procedures is given in Appendix 3.

### 9.2.4 Regulatory Reporting Requirements for SAEs

- Prompt notification by the investigator to the Sponsor of SAEs is essential so that legal obligations and ethical responsibilities towards the safety of participants and the safety of a product under clinical investigation are met.



Approved v1.0 930137673 4.0

- An investigator who receives an investigator safety report describing SAEs or other specific safety information (eg, summary or listing of SAEs) from the Sponsor will file it along with the Investigator's Brochure and will notify the IRB/IEC, if appropriate according to local requirements.

Sponsor or designee will be reporting adverse events to regulatory authorities and ethics committees according to local applicable laws including European Directive 2001/20/EC and FDA Code of Federal Regulations 21 CFR Parts 312 and 320. A SUSAR (Suspected, Unexpected Serious Adverse Reaction) is a subset of SAEs and will be reported to the appropriate regulatory authorities and investigators following local and global guidelines and requirements.

### 9.2.5 Pregnancy

If, following initiation of the study treatment, it is subsequently discovered that a participant is pregnant or may have been pregnant at the time of study exposure, including during at least 5 half-lives after product administration, the investigator must immediately notify the BMS Medical Monitor or designee of this event and complete and forward a Pregnancy Surveillance Form to BMS Designee within 24 hours of awareness of the event and in accordance with SAE reporting procedures described in Appendix 3.

If the investigator determines a possible favorable benefit/risk ratio that warrants continuation of study treatment, or re-initiation of study treatment, a discussion between the investigator and the BMS Medical Monitor or designee must occur. If, for whatever reason, the pregnancy has ended, confirmed by negative serum pregnancy test, treatment may be resumed (at least 3 weeks and not greater than 6 weeks after the pregnancy has ended), following approvals of participant /Sponsor /IRB/EC, as applicable.

Follow-up information regarding the course of the pregnancy, including perinatal and neonatal outcome and, where applicable, offspring information must be reported on the Pregnancy Surveillance Form.

Any pregnancy that occurs in a female partner of a male study participant should be reported to Sponsor or designee. In order for Sponsor or designee to collect any pregnancy surveillance information from the female partner, the female partner must sign an informed consent form for disclosure of this information. Information on this pregnancy will be collected on the Pregnancy Surveillance Form.

In cases where a study drug can be present in seminal fluid, at exposures sufficient to potentially cause fetal toxicity, and if any sexual activity (e.g. vaginal, anal, oral) has occurred between a male participant and a pregnant WOCBP partner(s), the information should be reported to the Sponsor or designee, even if the male participant has undergone a successful vasectomy. In order for Sponsor or designee to collect any pregnancy surveillance information from the female partner, the female partner(s) must sign an informed consent form for disclosure of this information. Information on the pregnancy will be collected on the Pregnancy Surveillance Form.



Approved v1.0 930137673 4.0

### 9.2.6 Laboratory Test Result Abnormalities

The following laboratory test result abnormalities should be captured on the nonserious AE CRF page or SAE Report Form electronic, as appropriate. Paper forms are only intended as a back-up option when the electronic system is not functioning.

- Any laboratory test result that is clinically significant or meets the definition of an SAE
- Any laboratory test result abnormality that required the participant to have study treatment discontinued or interrupted
- Any laboratory test result abnormality that required the participant to receive specific corrective therapy

It is expected that wherever possible, the clinical rather than laboratory term would be used by the reporting investigator (eg, anemia versus low hemoglobin value).

### 9.2.7 Potential Drug Induced Liver Injury (DILI)

Wherever possible, timely confirmation of initial liver-related laboratory abnormalities should occur prior to the reporting of a potential DILI event. All occurrences of potential DILIs, meeting the defined criteria, must be reported as SAEs (see Section 9.2.1 and Appendix 3 for reporting details).

Potential drug induced liver injury is defined as:

1) AT (ALT or AST) elevation > 3 times upper limit of normal (ULN)
2) AND
3) Total bilirubin > 2 times ULN, without initial findings of cholestasis (elevated serum alkaline phosphatase),
4) AND
5) No other immediately apparent possible causes of AT elevation and hyperbilirubinemia, including, but not limited to, viral hepatitis, pre-existing chronic or acute liver disease, or the administration of other drug(s) known to be hepatotoxic.

### 9.2.8 Other Safety Considerations

Any significant worsening noted during interim or final physical examinations, electrocardiogram, x-ray filming, any other potential safety assessment required or not required by protocol should also be recorded as a nonserious or serious AE, as appropriate, and reported accordingly.

## 9.3 Overdose

An overdose is defined as the accidental or intentional administration of any dose of a product that is considered both excessive and medically important. Overdoses that meet the regulatory definition of SAE will be reported as an SAE (see Appendix 3).

## 9.4 Safety

Planned time points for all safety assessments are listed in the Schedule of Activities.



Approved v1.0 930137673 4.0

Participants will be evaluated for safety if they have received any study drug. Toxicity assessments will be continuous during the treatment phase. During the long-term follow-up phase (Table 2-3), toxicity assessments should be done in person for Follow-Up Visits 1 and 2. Following Visits 1 and 2, additional in-person safety assessments will be conducted approximately every 12 weeks through 12 months (12 months counted from Follow-Up Visit 2) in randomized participants who received blinded therapy. For participants receiving open-label nivolumab monotherapy after recurrence, additional safety visits will be conducted approximately every 12 weeks through 12 months (12 months counted from Follow-Up Visit 2). These additional safety assessments in participants being treated after recurrence (per protocol) may be accomplished by an in-person visit (eg, examination, laboratory tests) or by phone contact. Please refer to the Schedule of Activities, Table 2-3 for additional details regarding safety follow-up after discontinuation of study treatment.

Once participants reach the long-term follow-up phase, either in person (eg, during surveillance assessments) or documented telephone calls to assess the participant's vital status are acceptable.

Adverse events, including laboratory abnormalities, will be reported and managed per NCI CTCAE v5. Some of the assessments may not be captured as data in the CRF. They are intended to be used as safety monitoring by the treating physician. Additional testing or assessments may be performed as clinically necessary or where required by institutional or local regulations.

### 9.4.1 Physical Examinations

Refer to Schedule of Activities (See Section 2).

### 9.4.2 Vital signs

Refer to Schedule of Activities (See Section 2).

### 9.4.3 Electrocardiograms

Refer to Schedule of Activities (See Section 2).

### 9.4.4 Clinical Safety Laboratory Assessments

Investigators must document their review of each laboratory safety report. See the Schedule of Activities (Section 2) for specific assessments and time points.

| **Hematology - CBC** | |
|---|---|
| Hemoglobin | |
| Hematocrit | |
| Total leukocyte count, including differential | |
| Platelet count | |
| **Chemistry** | |
| Aspartate aminotransferase (AST)<br>Alanine aminotransferase (ALT)<br>Total bilirubin<br>Alkaline phosphatase<br>Lactate dehydrogenase (LDH) | Albumin at screening and as clinically indicated<br>Sodium<br>Potassium<br>Chloride |



Approved v4.0 930137673 4.0

| | |
|---|---|
| Creatinine<br>Blood urea nitrogen (BUN) or serum urea<br>Glucose<br>Amylase at screening and as clinically indicated<br>Lipase at screening and as clinically indicated | Calcium<br>Phosphorus or phosphate<br>TSH, free T3 and free T4 - screening<br>TSH, with reflexive fT3 and fT4 if TSH is abnormal - on treatment<br>Magnesium<br>Creatine kinase<br>Creatinine clearance (CLcr) - screening only<br>ACTH at screening and as clinically indicated<br>Cortisol at screening and as clinically indicated |
| **Serology** | |
| Serum for hepatitis C antibody, hepatitis B surface antigen, HIV-1 and -2 antibody (screening only)<br>Hepatitis B/C (HBsAg, HCV antibody, or HCV RNA) - screening only<br>Note: Testing for HIV must be performed at sites where mandated locally. | |
| **Other Analyses** | |
| Pregnancy test (WOCBP only: minimum sensitivity 25 IU/L or equivalent units of HCG as indicated in Section 2). | |
| Follicle-stimulating hormone (FSH) (screening - only required to confirm menopause in women < age 55) | |

### 9.4.5 *Imaging Safety Assessment*

Any incidental findings of potential clinical relevance that are not directly associated with the objectives of the protocol should be evaluated and handled by the Study Investigator as per standard medical/clinical judgment.

## 9.5 Pharmacokinetic and Immunogenicity Assessments

Samples for PK and immunogenicity assessments will be collected for all participants receiving nivolumab or placebo, and for participants receiving open-label nivolumab monotherapy after first recurrence as described in Table 9.5-1. Placebo PK and Anti-Drug Antibody (ADA) samples will not be analyzed. All timepoints are relative to the start of study drug administration. All on-treatment timepoints are intended to align with days on which study drug is administered. If it is known that a dose is going to be delayed, then the predose sample should be collected just prior to the delayed dose. However, if a predose sample is collected but the dose is subsequently delayed, an additional predose sample should not be collected. Further details of sample collection, processing, and shipment will be provided in the laboratory procedures manual.

Blood samples should be drawn from a site other than the infusion site (ie, contralateral arm) on days of infusion for all pre-dose and end of infusion-PK (EOI-PK) samples. **Exception: On sampling days where EOI-PK samples are NOT drawn, to minimize the number of needle sticks, the pre-dose sample can be drawn immediately from the same arm intended for study drug infusion from the catheter prior to IV insertion and prior to any saline or fluid flush**



Approved v1.0 930137673 4.0

**into the port to avoid diluting the blood sample.** Nivolumab infusion is specified in the protocol for 30 minutes and is typically programmed for 30 minutes on the infusion pumps. Due to the nature by which the infusion pumps operate, there could be some residual dead volume in the IV tubing, and will be variable depending on the pump and IV tubing used. The EOI-PK sample is drawn with the intent of accurately estimating the Cmax, and as such should be drawn when all the study drug has been infused. If the site believes that all this drug was infused within the 30 minutes, then the EOI-PK sample occurs within approximately 2 minutes of this 30-minute infusion. If a flush needs to be administered to clear the IV lines of the drug and deliver all drug, then the EOI-PK sample should be drawn approximately 2 minutes prior to end of the flush.

If the infusion was interrupted, the interruption details will also be documented on the CRF. Blood samples will be processed to collect serum and stored preferably at -70°C (samples may be stored at -20°C up to 2 months). Further details of pharmacokinetic sample collection and processing will be provided to the site in the lab manual. The serum samples will be analyzed for drug (nivolumab) and ADA (anti-nivolumab antibodies) by validated immunoassays. Samples with a positive ADA response may also be analyzed for neutralizing ADA response to nivolumab.

In addition, selected serum samples may be analyzed by an exploratory method that measures nivolumab, or detect anti-drug antibodies for technology exploration purposes; exploratory results will not be reported. The corresponding serum samples designated for either PK, immunogenicity [redacted] assessments may also be used for any of those analyses, if required (eg, insufficient sample volume to complete testing or to follow-up on suspected immunogenicity related AE).



Approved v4.0 930137673 4.0

**Table 9.5-1: Pharmacokinetic (PK) and Immunogenicity Sampling Collections for Blinded Nivolumab or Placebo-Treated Participants or Participants Treated with Open-Label Nivolumab After First Recurrence**

| Study Day[a] (1 Cycle = 4 weeks) | Time (Relative To Dosing) Hour | Time (Relative To Dosing) Hour: Min | Pharmacokinetic Serum Sample for Nivolumab | Immunogenicity Serum for Nivolumab |
|---|---|---|---|---|
| Cycle 1 Day 1 | Predose[b] | 0:00 | X | X |
| | End of Infusion[c] | 0:30 | X | |
| Cycle 2 Day 1 | Predose[b] | 0:00 | X | X |
| Cycle 3 Day 1 | Predose[b] | 0:00 | X | X |
| Cycle 6 Day 1 | Predose[b] | 0:00 | X | X |
| | End of Infusion[c] | 0:30 | X | |
| Cycle 9 Day 1 | Predose[b] | 0:00 | X | X |
| FU1<br>First 2 Follow-up visits (30 ± 7 days from the discontinuation of study drug) | N/A | N/A | X | X |
| FU2<br>First 2 Follow-up visits (100 ± 7 days from the discontinuation of study drug) | N/A | N/A | X | X |

Abbreviation: FU1, Follow-Up Visit 1; FU2, Follow-Up Visit 2; Min, minutes.

[a] If a participant discontinues study drug treatment during the treatment/sampling period, they will move to sampling at the follow-up visits.

[b] Predose samples should be collected immediately before the administration of the drug (preferably within 30 minutes). If it is known that a dose is going to be delayed, then the predose sample should be collected immediately prior to the delayed dose. However, if a predose sample is collected but the dose is subsequently delayed, an additional predose sample should not be collected.

[c] The end of infusion-PK sampling (EOI-PK) occurs when the entire nivolumab dose in the infusion bag is administered to the patient. If diluent will not be administered to flush the IV lines, this sample should be taken immediately prior to stopping the IV infusion (preferably within 2 minutes prior to the end of infusion). If diluent is used to flush the dose remaining in the infusion line, then the EOI-PK sample will occur when there is no dose remaining in the infusion line after a flush. If the end of IV infusion is delayed to beyond the nominal infusion duration, the collection of this sample should also be delayed accordingly.



Approved v4.0 930137673 4.0

## 9.6 Pharmacodynamics

[redacted]

## 9.7 Pharmacogenomics

[redacted]



Approved v4.0 930137673 4.0

[redacted]

[redacted]



Approved v4.0 930137673 4.0

[redacted]

### *9.8.1 Additional Research Collection*

This protocol will include residual sample storage for additional research (AR).

**For All US sites:**

Additional research is required for all study participants, except where prohibited by IRBs/ethics committees or academic/institutional requirements. Where one or more of these exceptions occurs, participation in the additional research should be encouraged but will not be a condition of overall study participation.

If the IRB/ethics committees and site agree to the mandatory additional research retention and/or collection, then the study participant must agree to the mandatory additional research as a requirement for inclusion in the study.

If optional participation is permitted and approved, then the study participants may opt out of the additional research retention and/or collection.

**For non-US Sites**

Additional research is optional for all study participants, except where retention and/or collection is prohibited by local laws or regulations, ethics committees, or institutional requirements.

This collection for additional research is intended to expand the translational R&D capability at Bristol-Myers Squibb, and will support as yet undefined research aims that will advance our understanding of disease and options for treatment. It may also be used to support health authority requests for analysis, and advancement of pharmacodiagnostic development to better target drugs to the right patients. This may also include genetic/genomic exploration aimed at exploring disease pathways, progression and response to treatment etc.



Approved v1.0 930137673 4.0

**Sample Collection and Storage**

All requests for access to samples or data for additional research will be vetted through a diverse committee of the study Sponsor's senior leaders in Research and Development (or designee) to ensure the research supports appropriate and well-defined scientific research activities.

Residual P [redacted] will also be retained for additional research purposes.

Samples kept for future research will be stored at the BMS Biorepository in [redacted] USA or an independent, BMS-approved storage vendor.

The manager of these samples will ensure they are properly used throughout their usable life and will destroy the samples at the end of the scheduled storage period, no longer than fifteen (15) years after the end of the study or the maximum allowed by applicable law.

Transfers of samples by research sponsor to third parties will be subject to the recipient's agreement to establish similar storage procedures.

Samples will be stored in a coded fashion, and no researcher will have access to the key. The key is securely held by the Investigator at the clinical site, so there is no direct ability for a researcher to connect a sample to a specific individual.

Further details of sample collection and processing will be provided to the site in the procedure manual**.**

**Table 9.8.1-1: Residual Sample Retention for Additional Research Schedule**

| Sample Type | Timepoints for which residual samples will be retained |
|---|---|
| PK Serum | All |
| [redacted] | |

Abbreviations: [redacted] PK, pharmacokinetics.

### *9.8.2 Tissue Specimens*

Pre-treatment tumor tissue specimens, not previously irradiated ("not previously irradiated" applies to participants with unresectable/metastatic disease recurrence entering open-label nivolumab), in the form of a paraffin embedded block to contain sufficient tissue for at least 15 sections or a minimum of 15 unstained slides, preferably freshly cut, from the primary diagnostic biopsy must be shipped to the central lab prior to randomization or treatment assignment, for

blinded or open-label nivolumab treatment, respectively. If a minimum of 15 unstained slides or 1 FFPE block cannot be provided, the eligibility should be discussed with the Medical Monitor or designee. This minimum quantity applies to tissue obtained prior to randomization/assignment to the open label portion as well as tissue obtained upon disease recurrence or progression.

The following tissue attributes for the samples prior to randomization of participants with Stage IIB/C melanoma are not mandatory, but considered desirable to optimize downstream [redacted] analyses. The tissue biopsy samples should be from the primary diagnostic material (obtained for example by biopsy punch, shave, excision, incision, saucerization). If tissue from the primary diagnostic biopsy is insufficient, it may be supplemented by tissue from subsequent surgeries, provided at least 3 mm of tumor in smallest dimension is present. A dermatopathologist's review/input is encouraged. Pathology samples acquisition guidelines and submission process will be outlined in the study Laboratory Manual.

[redacted]



Approved v1.0 930137673 4.0

[redacted]



Approved v4.0 930137673 4.0

[redacted]



Approved v4.0 930137673 4.0

## 9.9 Medical Resource Utilization and Health Economics

Healthcare utilization data, associated with medical encounters, will be collected in the CRF by the investigator and study-site personnel for all participants throughout the study. Protocol-mandated procedures, tests, and encounters are excluded. The data collected may be used to conduct exploratory economic analyses and will include:

- Number and duration of medical care encounters, including surgeries, and other selected procedures (inpatient and outpatient)
- Duration of hospitalization (total days length of stay, including duration by wards; eg, intensive care unit)
- Number and character of diagnostic and therapeutic tests and procedures
- Outpatient medical encounters and treatments (including physician or emergency room visits, tests and procedures, and medications).

## 9.10 Other Assessments

Not applicable.

# 10 STATISTICAL CONSIDERATIONS

## 10.1 Sample Size Determination

### *10.1.1 RFS*

The sample size of the study is based on a comparison of the RFS distribution between participants randomized to nivolumab and participants randomized to placebo. Simulation models incorporating aspects of immunotherapy such as delayed separation (observed as late separation of survival curves between the experimental and placebo arms) and long-term survival benefits (observed as a long-lasting plateau towards the tail of the survival curve) were developed for sample size estimation. Sample size calculations for this study design were done using EAST (v 6.4.1).

For this comparison of RFS between nivolumab and placebo in all randomized participants, approximately 154 RFS events would be required for a two-sided experiment-wise alpha = 0.05 log-rank test, to show a statistically significant difference in RFS between the treatment arms with at least 90% power when the average hazard ratio (HR) of the nivolumab arm to the placebo arm is 0.573. Given an estimated accrual rate as described in Table 10.1.1-1 below, the accrual of 780 participants (ie, 520 participants in the nivolumab arm and 260 participants in the placebo arm) will take approximately 29.6 months. Under the assumptions for accrual, an assumed delayed treatment effect of 6 months as per the Sunbelt Melanoma Trial[110] and assumed HR as stated above (HR = 1 for the first 6 months, HR = 0.537 after 6 months, HR = 1 from Year 10 [plateau effect]), it would take approximately 68.1 months from the randomization of the first participant to observe the required number of RFS events. An observed HR of 0.707 or less would result in a statistically significant improvement at the final analysis of RFS.



Approved v4.0 930137673 4.0

**Table 10.1.1-1: Accrual Rate Assumption**

| Month | 0 | 1 | 2 | 3 | 4 | 5 | 6-9 | 10-14 | 15-17 | 18+ |
|---|---|---|---|---|---|---|---|---|---|---|
| Accrual Rate[a] | 2 | 10 | 13 | 21 | 30 | 10 | 0[b] | 6 | 28 | 50 |

[a] Assume 5% drop-out (lost to follow-up) rate in each arm by Month 50.

[b] 0.001 in East that doesn't allow 0 technically.

Final analysis of RFS will be conducted when approximately 154 RFS events have occurred. In case the events occur slower than anticipated, final analysis of RFS will be conducted when approximately 139 events (90% of planned number of events for the final analysis) have been observed. In that case, the power would be at least 86% (and the critical HR would be 0.692).

One interim analysis of RFS will be conducted when approximately 123 RFS events (80% information fraction) have been reached among all randomized participants. We estimate this would occur when all participants have a minimum follow-up of approximately 23.7 months from the randomization of the last participant. The estimated timing for this interim analysis is at 53.3 months. The stopping boundaries at the interim and final analyses will be derived based on the exact number of RFS events using Lan-DeMets alpha spending function with O'Brien-Fleming boundaries. With an interim RFS analysis at approximately 123 RFS events, the type I error would be 0.024 (two-sided), the power 62.8%, and an observed HR of 0.65 or less would result in a statistically significant improvement. The type I error to be used for final RFS analysis would be 0.043 (two-sided).

### *10.1.2 Overall Survival*

OS will be formally compared only if the null hypothesis for the primary endpoint (RFS) is rejected.

For the comparison of OS between nivolumab and placebo in all randomized participants, approximately 277 deaths would be required for a two-sided experiment-wise alpha = 0.05 log-rank test, to show a statistically significant difference in OS with at least 79.6% power when the average hazard ratio (HR) of the nivolumab arm to the placebo arm is 0.7. It is projected that an observed HR of 0.777 or less would result in a statistically significant improvement at the final analysis of OS.

No formal OS interim analysis is planned at the time of interim analysis (IA) and final analysis (FA) for RFS due to anticipated immaturity of the OS data.

Although formal OS testing is planned at OS FA, descriptive statistics for OS will be prepared at RFS IA and/or RFS FA upon regulatory requests. If OS results (beyond the frequency of deaths per arm) including Kaplan-Meier (KM) curves are requested, an administrative alpha of 0.0001 will be spent as alpha penalty. Should such analyses be conducted, only a BMS restricted team will have access to OS descriptive results.



Approved v4.0 930137673 4.0

The projected number of deaths that will have occurred at the time of interim or final RFS analysis is 53 deaths (or 19% of the 277 final deaths) and 87 deaths (or 31% of the 277 final deaths), respectively.

To ensure sufficient maturity of the OS data at the time formal analysis is performed, one formal OS interim analysis will be conducted when approximately 166 deaths (60% information fraction) have been reached among all randomized participants, which is expected to occur after the final analysis of RFS.

It is estimated this would occur when all participants have a minimum follow-up of approximately 63 months from the randomization of the last participant. The estimated timing for this interim analysis is at 93 months after first patient first treatment. The stopping boundaries at the interim and final analyses will be derived based on the exact number of OS events using Lan-DeMets alpha spending function with O'Brien-Fleming boundaries. With an interim OS analysis at approximately 166 deaths, the type I error would be 0.008 (two-sided), the power 30.7%, and an observed HR of 0.644 or less would result in a statistically significant improvement. The type I error to be used for final OS analysis would be 0.048 (two-sided).

**Table 10.1.2-1: Overview of Timing of Analyses**

| Analysis | When | Description |
|---|---|---|
| IA for RFS | Approximately 123 RFS events have occurred (80% IF)<br>Expected time from FPFT:53.3 m<br>Expected min FU: 23.7 m | Alpha spent: 0.024, power: 62.8%, cHR: 0.65 |
| FA for RFS | Approximately 154 RFS events have occurred<br>Expected time from FPFT: 68.1 m [a]<br>Expected min FU: 38.5 m [a] | Alpha spent: 0.043, Cum alpha spent: 0.05, power: 90.2%, cHR: 0.707 |
| IA for OS | Approximately 166 OS events have occurred (60% IF)<br>Expected time from FPFT: 93 m<br>Expected min FU: 63 m | Alpha spent: 0.008, power: 30.7%, cHR: 0.644 |
| FA for OS | Approximately 277 OS events have occurred<br>Expected time from FPFT: 133 m [b]<br>Expected min FU: 103 m [b] | Alpha spent: 0.048, Cum alpha spent: 0.05, power: 79.6%, cHR: 0.777 |

Abbreviations: cHR, critical hazard ratio; cum, cumulative; FA, final analysis; FPFT, first participant first treatment; FU, follow-up; IA, interim analysis; IF, information fraction; OS, overall survival; RFS, recurrence-free survival.

[a] In case RFS events occur too slowly, FA may be triggered when at least 90% IF of RFS events is observed (power at least 86%, cum alpha spent ≤ 0.05)

[b] In case OS events occur too slowly, FA may be triggered when a minimum follow-up of 9 years (ie, 108 months) is reached (expected in October 2030).



Approved v4.0 930137673 4.0

## 10.2 Populations for Analyses

For purposes of analysis, the following populations are defined:

| Population | Description |
|---|---|
| All enrolled participants | All participants who signed an informed consent form and were registered into the IRT. |
| All randomized participants | All participants who were randomized to any treatment arm in the study. |
| All treated participants | All participants who received at least one dose of study drug. |
| PK participants | All treated participants with available serum time-concentration data. |
| Immunogenicity evaluable participants:<br>Nivolumab ADA evaluable participants | <br>All treated participants with baseline and at least 1 post baseline pre-infusion nivolumab immunogenicity assessment |

[redacted]

## 10.3 Statistical Analyses

The statistical analysis plan (SAP) will be developed and finalized before the first database lock for formal interim efficacy analysis, and will describe the selection of participants to be included in the analyses, and procedures for accounting for missing, unused, and spurious data. Below is a summary of planned statistical analyses of the primary and secondary endpoints.

A description of the participant population will be included in a statistical output report, including subgroups of age, gender and race.

### *10.3.1 Efficacy Analyses*

#### *10.3.1.1 Primary Endpoint Analysis*

The primary endpoint is RFS. The primary endpoint of RFS will be programmatically determined based on the disease recurrence date provided by the investigator and is defined as the time between the date of randomization and the date of first recurrence (local, regional or distant metastasis), new primary melanoma (including melanoma in situ), or death (whatever the cause), whichever occurs first. For participants who remain alive and whose disease has not recurred or did not die, RFS will be censored on the date of last evaluable disease assessment. For those participants who remained alive and had no recorded post-randomization tumor assessment, RFS will be censored on the day of randomization. Censoring rules for the primary analysis of RFS are presented in Table 10.3.1.1-1.



Approved v1.0 930137673 4.0

**Table 10.3.1.1-1: Censoring Scheme for Primary Definition of RFS**

| Situation | Date of Event or Censoring | Outcome |
|---|---|---|
| Recurrence (local, regional, distant, new primary melanoma (including melanoma in situ)) | Date of first recurrence | Event |
| Death without recurrence | Date of death | Event |
| No baseline disease assessment | Date of randomization | Censored |
| No on-study disease assessments and no death | Date of randomization | Censored |
| No recurrence and no death | Date of last evaluable disease assessment | Censored |
| New anticancer therapy, tumor-directed radiotherapy, or tumor-directed surgery received without recurrence reported prior to or on the same day of disease assessment | Date of last evaluable disease assessment prior to or on the same date of initiation of subsequent therapy | Censored |
| Second non-melanoma primary cancer reported prior or on the same day of disease assessment | Date of last evaluable disease assessment prior to or on the same date of diagnosis of second non-melanoma primary cancer | Censored |

RFS distributions will be compared between treatment groups (nivolumab vs placebo) using a two-sided log-rank test stratified AJCC T-Stage at Screening at the overall significance level of 5% (two-sided) in all randomized participants.

The HR and corresponding 100x (1-adjusted $\alpha$)% confidence intervals (CIs) will be estimated using a Cox proportional hazards model, with treatment group as a single covariate, stratified by the above factor.

RFS curves will be estimated using Kaplan-Meier (KM) product-limit methodology. Median RFS with two-sided 95% CIs using the log-log transformation will be computed. In addition, RFS rates at 6, 12, 18, 24, 30, and 36 months (and yearly after depending on follow-up) with two-sided 95% CIs using the log-log transformation will be computed.

The proportional hazards assumption will be assessed at the time of analysis and appropriate methods will be employed in case of non-proportional hazards.

#### *10.3.1.2 Secondary Endpoint Analyses*

**Overall Survival (OS)**

OS is defined as the time between the date of randomization and the date of death. For participants without documentation of death, OS will be censored on the last date the participant was known to be alive. OS will be followed continuously while participants are on the study drug and every 12 weeks via in-person or phone contact after participants discontinue the study drug.

OS distributions will be compared in hierarchical testing order (order defined in Section 10.3.4) between treatment groups (nivolumab vs placebo) using a two-sided log-rank test stratified by AJCC T-Stage at Screening at the overall significance level of 5% (two-sided) in all randomized



Approved v1.0 930137673 4.0

participants. The hazard ratio and corresponding two-sided 100x (1-adjusted α)% CIs will be estimated using a Cox proportional hazards model, with treatment group as a single covariate, stratified by the above factors.

OS curves will be estimated using Kaplan-Meier (KM) product-limit methodology. Median OS with two-sided 95% CIs using the log-log transformation will be computed. In addition, OS rates at 6, 12, 18, 24, 30 and 36 months (and yearly after depending on follow-up) with two-sided 95% CIs using the log-log transformation will be computed.

**Distant Metastasis-Free Survival (DMFS)**

DMFS is defined as the time between the date of randomization and the date of first distant metastasis or date of death (whatever the cause), whichever occurs first.

Censoring rules for the analysis of DMFS are presented in Table 10.3.1.2-1.

**Table 10.3.1.2-1: Censoring Scheme for Primary Definition of DMFS**

| Situation | Date of Event or Censoring | Outcome |
|---|---|---|
| Distant metastasis | Date of first distant metastasis | Event |
| Death without distant metastasis | Date of death | Event |
| No baseline disease assessment | Date of randomization | Censored |
| No on-study disease assessments and no death | Date of randomization | Censored |
| No distant metastasis and no death | Date of last evaluable disease assessment | Censored |

DMFS will be analyzed using similar analyses methods as for RFS. No multiplicity adjustment will be applied.

**Investigator-Assessed Outcomes on Next-Line Therapies**

PFS2 is defined as the time from randomization to recurrence/objective disease progression after the start of the next-line of systemic anti-cancer therapy, or to the start of a second next-line systemic therapy, or to death from any cause, whichever occurs first.

In case PFS2 cannot be reliably determined, we will analyze the time from randomization to end or discontinuation of next-line treatment, second objective disease progression, or death from any cause, whichever occurs first.

The hazard ratio and corresponding 95% confidence intervals (CIs) will be estimated using a Cox proportional hazards model, with treatment group as a single covariate.

Objective response rates and duration of treatment on next-line therapies will be summarized.

### *10.3.2 Safety Analyses*

Safety and tolerability of nivolumab and placebo will be measured by the incidence of adverse events, serious adverse events, deaths, and laboratory abnormalities.



Approved v1.0 930137673 4.0

Safety analyses will be performed in all treated participants. Descriptive statistics of safety will be presented using NCI CTCAE v5 by treatment group (for the nivolumab and placebo arms). All on-study AEs, treatment-related AEs, SAEs, and treatment-related SAEs will be tabulated using worst grade per NCI CTCAE v5 criteria by system organ class and preferred term. On-study lab parameters including hematology, chemistry, liver function, and renal function will be summarized using worst grade NCI CTCAE v5 criteria.

### 10.3.3 Other Analyses

[redacted]

#### 10.3.3.3 Pharmacokinetic Analyses

The nivolumab concentration data obtained in this study will be combined with data from other studies in the clinical development program to develop a population PK model. This model will

be used to evaluate the effects of intrinsic and extrinsic covariates on the PK of nivolumab. In addition, exposure-response analyses with selected efficacy and safety endpoints may be conducted. Results of population PK and exposure response-analyses will be reported separately.

[redacted]

#### *10.3.3.5 Immunogenicity Analyses*

Methodology for analysis of immunogenicity will be described in the statistical analysis plan.

#### *10.3.3.6 Analyses of Follow-Up Data from Participants Receiving Optional Open-Label Nivolumab Treatment*

Methodology for analysis of follow-up data from participants receiving optional open-label nivolumab treatment will be described in the statistical analysis plan.

### *10.3.4 Interim Analyses*

One formal interim analysis of RFS will be conducted when approximately 123 RFS events (80% information fraction) have been reached among all randomized participants. The stopping boundaries at the interim and final analyses will be derived based on the exact number of RFS events using Lan-DeMets alpha spending function with O'Brien-Fleming boundaries. With an interim RFS analysis at approximately 123 RFS events, the type I error would be 0.024 (two-sided). The type I error to be used for final RFS analysis would be 0.043 (two-sided).

RFS and OS will be tested using a hierarchical procedure: RFS will be compared first in all randomized participants with an alpha allocation of 0.05 (two-sided). If significant, OS will be compared in all randomized participants with an alpha allocation of 0.05 (two-sided).

One formal OS interim analysis will be conducted when approximately 166 deaths (60% information fraction) have been reached among all randomized participants, which is expected to occur after the final analysis of RFS.

It is estimated this would occur when all participants have a minimum follow-up of approximately 63 months from the randomization of the last participant. The estimated timing for this interim analysis is at 93 months. The stopping boundaries at the interim and final analyses will be derived based on the exact number of OS events using Lan-DeMets alpha spending function with O'Brien-Fleming boundaries. With an interim OS analysis at approximately 166 deaths, the type I error would be 0.008 (two-sided), the power 30.7%, and an observed HR of 0.644 or less would result in a statistically significant improvement. The type I error to be used for final OS analysis would be 0.048 (two-sided).

The DMC will have access to periodic unblinded interim reports of efficacy and safety to allow a risk/benefit assessment. Details will be included in the DMC charter.

The Statistical Analysis Plan will further describe the planned interim analyses.



Approved v1.0 930137673 4.0

# 11 REFERENCES

1 Ossio R, Marin-Roldan R, Martinez-Said H, et al. Melanoma: a global perspective. Nature Reviews – Cancer. 2017;17:393-394.

2 Gershenwald J, Scolyer R, Hess K, et al. Melanoma Staging: Evidence-Based Changes in the American Joint Committee on Cancer Eighth Edition Cancer Staging Manual. CA Cancer J Clin. 2017;67(6):472–492.

3 BMS Internal Communication, data on file.

4 Leonardi G, Falzone L, Salemi R, et al. Cutaneous melanoma: From pathogenesis to therapy (Review). Int J of Oncol. 2018;52:1071-1080.

5 Austin MT, Xing Y, Hayes-Jordan AA, et al. Melanoma incidence rises for children and adolescents: an epidemiologic review of pediatric melanoma in the United States. J Pediatr Surg. 2013 Nov;48(11):2207-13.

6 Strouse JJ, Fears TR, Tucker MA, et al. Pediatric melanoma: risk factor and survival analysis of the surveillance, epidemiology and end results database. J Clin Oncol. 2005;23(21):4735.

7 Egger M, Bhutiani N, Farmer R, et al. Prognostic factors in melanoma patients with tumor-negative sentinel lymph nodes. Surgery. 2016;159:1412-21.

8 Surveillance, Epidemiology, and End Results (SEER) Program (www.seer.cancer.gov) Cancer Statistics Review (CSR), 1975-2015.

9 Landow S, Gjelsvik A, Weinstock M. Mortality burden and prognosis of thin melanomas overall and by subcategory of thickness, SEER registry data, 1992-2013. J Am Acad Dermatol. 2016;76(2):258-263.

10 NCCN Clinical Practice Guidelines in Oncology-Melanoma, Version 3.2018, July 12, 2018.

11 Huang AC, Postow MA, Orlowski RJ, et al. T-cell invigoration to tumor burden ratio associated with anti-PD-1 response. Nature. 2017;545:60-5.

12 Poklepovic A, Carvajal R. Prognostic Value of Low Tumor Burden in Patients With Melanoma. Oncology. 2018;32(9):e90-e96

13 BMS Internal Communication, data on file.

14 Garbe C, Peris K, Hauschild A, et al. Diagnosis and treatment of melanoma. European consensus-based interdisciplinary guideline - Update 2016. Eur J Cancer. 2016;63:201-217.

15 Saiyed F, Hamilton E, Austin M. Pediatric melanoma: incidence, treatment, and prognosis. Pediatric Health, Medicine and Therapeutics. 2017;8:39–45.



Approved v4.0 930137673 4.0

16 Averbook B, Lee S, Delman K, et al. Pediatric Melanoma: Analysis of an International Registry. Cancer. 2013;119(22):4012-4019.

17 Massagué J, Obenauf AC. Metastatic colonization by circulating tumour cells. Nature. 2016;529:298–306.

18 Haas L, Wiesner T, Obenauf AC. A new era of proactive melanoma therapy: hit hard, hit early. The British Journal of Dermatology. 2018;178(4):817-820.

19 Domingues B, Lopes JM, Soares P, et al. Melanoma treatment in review. Immunotargets Ther. 2018;7:35-49.

20 Smith F, Downey S, Klapper J, et al. Treatment of metastatic melanoma using interleukin-2 alone or in conjunction with vaccines. Clin Cancer Res. 2008;14:5610-5618.

21 Rosenberg S, Yang J, Topalian S, et al. Treatment of 283 consecutive patients with metastatic melanoma or renal cell cancer using high-dose interleukin 2. JAMA. 1994;271:907-913.

22 Atkins MB, Kunkel L, Sznol M, et al. High-dose recombinant interleukin-2 therapy in patients with metastatic melanoma: long-term survival update. Cancer J Sci Am. 2000;6 Suppl 1:S11-14.

23 Atkins M, Hsu J, Lee S, et al. Phase III trial comparing concurrent biochemotherapy with cisplatin, vinblastine, dacarbazine, interleukin-2, and interferon alfa-2b with cisplatin, vinblastine, and dacarbazine alone in patients with metastatic malignant melanoma (E3695): a trial coordinated by the Eastern Cooperative Oncology Group. J Clin Oncol. 2008;26(35):5748-5754.

24 Ipilimumab US Package Insert; revised July 2018.

25 Hodi F, O'Day S, McDermott D, et al. Improved survival with ipilimumab in patients with metastatic melanoma. N Engl J Med. 2010;363:711-723.

26 Robert C, Thomas L, Bondarenko I, et al. Ipilimumab plus dacarbazine for previously untreated metastatic melanoma. N Engl J Med. 2011;364:2517-2526.

27 Maio M, Grob J, Aamdal S, et al. Five-year survival rates for Treatment-Naive Patients With Advanced Melanoma Who Received Ipilimumab Plus Dacarbazine in a Phase III Trial. J Clin Oncol. 2015;33:1191-1196.

28 Nishino M, Giobbie-Hurder A, Ramaiya NH, et al. Response assessment in metastatic melanoma treated with ipilimumab and bevacizumab: CT tumor size and density as markers for response and outcome. J Immunother Cancer. 2014;2:40.

29 Robert C, Long G, Brady B, et al. Nivolumab in previously untreated melanoma without BRAF mutation. N Engl J Med. 2015;372:320-330.

30 Hodi F, Chiarion-Sileni V, Gonzalez R, et al. Nivolumab plus ipilimumab or nivolumab alone versus ipilimumab alone in advanced melanoma (CheckMate 067): 4-year outcomes of a multicentre, randomised, phase 3 trial. Lancet Oncol. 2018;19:1480–92.



Approved v1.0 930137673 4.0

31 Wolchok J, Chiarion-Sileni V, Gonzalez R, et al. Overall Survival with Combined Nivolumab and Ipilimumab in Advanced Melanoma. N Engl J Med. 2017;377:1345-56.

32 Ribas A, Puzanov I, Dummer R, et al. Pembrolizumab versus investigator-choice chemotherapy for ipilimumab-refractory melanoma (KEYNOTE-002): a randomized, controlled, phase 2 trial. Lancet Oncol. 2015;16:908-918.

33 Robert C, Schachter J, Long G, et al. Pembrolizumab versus ipilimumab in Advanced Melanoma. N Engl J Med. 2015;372:2521-2532.

34 T-VEC (Imlygic) Package Insert, Revised 12/2018

35 Kaufman H, Amatruda T, Nemunaitis JJ, et al. Tumor size and clinical outcomes in melanoma patients (MEL pts) treated with talimogene laherparepvec (T-VEC). J Clin Oncol. 2015;33:9074.

36 Schadendorf D, Long GV, Stroiakovski D, et al. Three-year pooled analysis of factors associated with clinical outcomes across dabrafenib and trametinib combination therapy phase 3 randomised trials. Eur J Cancer. 2017;82:45-55.

37 Flaherty L, Othus M, Atkins M, et al. Southwest Oncology Group S0008: a phase III trial of high-dose interferon Alfa-2b versus cisplatin, vinblastine, and dacarbazine, plus interleukin-2 and interferon in patients with high-risk melanoma--an intergroup study of cancer and leukemia Group B, Children's Oncology Group, Eastern Cooperative Oncology Group, and Southwest Oncology Group. J Clin Oncol. 2014 Nov 20;32(33):3771-8.

38 Kirkwood J, Strawderman M, Ernstoff M, et al. Interferon Alfa-2b Adjuvant Therapy of High-Risk Resected Cutaneous Melanoma: The Eastern Cooperative Oncology Group Trial EST 1684. J Clin Oncol. 1996;14:7-17

39 Kirkwood J, Manola J, Ibrahim J, et al. A Pooled Analysis of Eastern Cooperative Oncology Group and Intergroup Trials of Adjuvant High-Dose Interferon for Melanoma. Clin Cancer Res. 2004;10:1670-1677.

40 Eggermont A, Suciu S, Santinami M, et al. Adjuvant therapy with pegylated interferon alfa-2b versus observation alone in resected stage III melanoma: final results of EORTC 18991, a randomised phase III trial. Lancet. 2008;372:117–26.

41 NCCN Clinical Practice Guidelines in Oncology-Melanoma, Version 2, 2019.

42 Lens M, Dawes M. Interferon Alfa Therapy for Malignant Melanoma: A Systematic Review of Randomized Controlled Trials. J Clin Oncol. 2002;20:1818-1825.

43 Weber J, Mandala M, Del Vecchio M, et al. Adjuvant Nivolumab versus Ipilimumab in Resected Stage III or IV Melanoma. N Engl J Med. 2017;377(19):1824-1835.

44 Eggermont A, Chiario-Sileni V, Grob J, et al. Prolonged Survival in Stage III Melanoma with Ipilimumab Adjuvant Therapy. N Engl J Med. 2016;375(19):1845–1855.

45 Pardoll D. Does the immune system see tumors as foreign or self? Annu Rev Immunol. 2003;21:807-39.



Approved v1.0 930137673 4.0

46 Zitvogel L, Tesniere A, Kroemer G. Cancer despite immunosurveillance: immunoselection and immunosubversion. Nat Rev Immunol. 2006;6:715-27.

47 Wheatley K, Ives N, Hancock B, et al. Does adjuvant interferon-a for high-risk melanoma provide a worthwhile benefit? A meta-analysis of the randomised trials. Cancer Treatment Reviews. 2003;29:241–252.

48 Hauschild A, Dummer R, Schadendorf D, et al. Longer Follow-Up Confirms Relapse-Free Survival Benefit With Adjuvant Dabrafenib Plus Trametinib in Patients With Resected BRAF V600–Mutant Stage III Melanoma. J Clin Oncol. 2018;36:1-9.

49 Kirkwood J, Ibrahim J, Sondak V, et al. High- and Low-Dose Interferon Alfa-2b in High-Risk Melanoma: First Analysis of Intergroup Trial E1690/S9111/C9190. J Clin Oncol. 2000;18:2444-2458.

50 Grob JJ, Dreno B, de la Salmoniere P, et al. Randomized trial of interferon α-2a as adjuvant therapy in resected primary melanoma thicker than 1·5 mm without clinically detectable node metastases. Lancet. 1998;351:1905-10.

51 Mocellin S, Lens MB, Pasquali S, et al. Interferon alpha for the adjuvant treatment of cutaneous melanoma. Cochrane Database of Systematic Reviews 2013, Issue 6. Art. No.: CD008955.

52 Ives N, Suciu S, Eggermont A, et al. Adjuvant interferon-a for the treatment of high-risk melanoma: An individual patient data meta-analysis. Ives et al. Eur J Cancer. 2017;82:171-183.

53 Maio M, Lewis K, Demidov L, et al. Adjuvant vemurafenib in resected, BRAFV600 mutation-positive melanoma (BRIM8): a randomised, double-blind, placebo-controlled, multicentre, phase 3 trial, Lancet Oncol. 2018;19:510–20.

54 Buja A, Sartor G, Scioni M, et al. Estimation of Direct Melanoma-related Costs by Disease Stage and by Phase of Diagnosis and Treatment According to Clinical Guidelines. Acta Derm Venereol. 2018;98:218–224.

55 Dunn GP, Bruce AT, Ikeda H, et al. Cancer immunoediting: from immunosurveillance to tumor escape. Nat Immunol. 2002;3:991-8.

56 Greenwald RJ, Freeman GH, Sharpe AH. The B7 family revisited. Annu Rev Immunol. 2004;23:515-48.

57 Freeman FJ, Long AJ, Iwai Y, et al. Engagement of the PD-1 immunohibitory receptor by a novel B7 family member leads to negative regulation of lymphocyte activation. J Exp Med. 2000;192(7):1027-34.

58 Sharpe AH, Wherry EJ, Ahmed R, et al. The function of programmed cell death 1 and its ligands in regulating autoimmunity and infection. Nature Immunol. 2007;8:237-45.

59 Wolchok JD, Hoos A, O'Day S, et al. Guidelines for the evaluation of immune therapy activity in solid tumors: immune-related response criteria. Clin Cancer Res. 2009;15:7412-20.



Approved v4.0 930137673 4.0

[60] Wilson M, Geskin L, Carvajal R, et al. Adjuvant nivolumab in high-risk stage IIb/IIc melanoma patients: Results from investigator initiated clinical trial. J Clin Oncol. 2021;39(15_suppl):9583.

[61] Amin MB, Edge SB, Greene FL, et al, eds. AJCC Cancer Staging Manual. 8th ed. New York: Springer; 2017.

[62] Lee A, Droppelmann N, Panageas K, et al. Patterns and Timing of Initial Relapse in Pathologic Stage II Melanoma Patients. Ann Surg Oncol. 2017;24(4):939-946.

[63] Dalal KM, Patel A, Brady MS, et al. Patterns of First-Recurrence and Post-recurrence Survival in Patients with Primary Cutaneous Melanoma After Sentinel Lymph Node Biopsy. Ann Surg Oncol. 2007;14(6):1934-1942.

[64] Grob J, Garbe C, Ascierto P, et al. Adjuvant melanoma therapy with new drugs: should physicians continue to focus on metastatic disease or use it earlier in primary melanoma? Lancet Oncol. 2018;19:e720-25.

[65] OPDIVO (nivolumab) US Prescribing Information. Bristol-Myers Squibb, Princeton, NJ. February 2018.

[redacted]



Approved v4.0 930137673 4.0

[redacted]



Approved v4.0 930137673 4.0

[redacted]

101 OPDIVO. Annex I Summary of Product Characteristics. Bristol-Myers Squibb Company. 2018.

[redacted]



Approved v1.0 930137673 4.0

[104] Centers for Disease Control (CDC), Criteria for Return to Work for Healthcare Personnel with Suspected or Confirmed COVID-19 (Interim Guidance) Updated May 5, 2020 https://www.cdc.gov/coronavirus/2019-ncov/hcp/return-to-work.html

[redacted]



Approved v4.0 930137673 4.0

# 12 APPENDICES



Approved v4.0 930137673 4.0

## APPENDIX 1 ABBREVIATIONS AND TRADEMARKS

| Term | Definition |
|---|---|
| ACTH | adrenocorticotropic hormone |
| ADA | anti-drug antibody |
| AE | adverse event |
| AIDS | acquired immunodeficiency syndrome |
| AJCC | American Joint Committee on Cancer |
| ALT | alanine aminotransferase |
| ANC | absolute neutrophil count |
| ANOVA | analysis of variance |
| aPTT | activated partial thromboplastin time |
| AR | additional research |
| AST | aspartate aminotransferase |
| AT | aminotransaminases |
| BICR | Blinded Independent Central Review |
| BMI | body mass index |
| BMS | Bristol-Myers Squibb |
| BP | blood pressure |
| BUN | blood urea nitrogen |
| CBC | complete blood count |
| CE | contrast enhanced |
| CFR | Code of Federal Regulations |
| cHL | Classical Hodgkin's Lymphoma |
| cHR | critical hazard ratio |
| CI | confidence interval |
| CIOMS | Council for International Organizations of Medical Sciences |
| CLcr | creatinine clearance |
| CMV | cytomegalovirus |
| CNS | central nervous system |
| COVID-19 | coronavirus disease 2019 |
| CRC | colorectal cancer |
| CRF | case report form |



Approved v4.0 930137673 4.0

| Term | Definition |
|---|---|
| CSR | clinical study report |
| CT | computed tomography |
| CTAg | Clinical Trial Agreement |
| [redacted] | |
| CTLA-4 | cytotoxic T-lymphocyte-associated protein-4 |
| cum | cumulative |
| DILI | drug-induced liver injury |
| DMC | Data Monitoring Committee |
| [redacted] | |
| DRESS | drug reaction with eosinophilia and systemic symptoms |
| ECG | electrocardiogram |
| ECOG | Eastern Cooperative Oncology Group |
| EDC | electronic data capture |
| eCRF | electronic Case Report Form |
| eGFR | estimated glomerular filtration rate |
| EHR | electronic health record |
| EMA | European Medicines Agency |
| EOI | end of infusion |
| EOI-PK | end of infusion-pharmacokinetics |
| [redacted] | |
| EU | European Union |
| FA | final analysis |
| [redacted] | |
| FDA | Food and Drug Administration |
| FFPE | formalin-fixed paraffin embedded |
| [redacted] | |
| FPFT | first participant first treatment |
| FSH | follicle-stimulating hormone |
| FU1 | Follow-Up Visit 1 |



Approved v1.0 930137673 4.0

| Term | Definition |
|---|---|
| FU2 | Follow-Up Visit 2 |
| GBS | Guillain-Barre syndrome |
| GCP | Good Clinical Practice |
| GFR | glomerular filtration rate |
| [redacted] | |
| HBsAg | hepatitis B surface antigen |
| HBV | hepatitis B virus |
| HCV | hepatitis C virus |
| HIPAA | Health Insurance Portability and Accountability Act |
| HIV | human immunodeficiency virus |
| HR | hazard ratio |
| HRT | hormone replacement therapy |
| [redacted] | |
| IA | interim analysis |
| IB | Investigator Brochure |
| ICD | International Classification of Diseases |
| ICF | informed consent form |
| ICH | International Conference on Harmonisation |
| ICMJE | International Committee of Medical Journal Editors |
| ie | id est (that is) |
| IEC | Independent Ethics Committee |
| IF | information fraction |
| IFN | interferon |
| IgG | Immunoglobin G |
| [redacted] | |
| IMP | Investigational Medicinal Products |
| IP | Investigational Product |
| IRB | Institutional Review Board |
| IRT | Interactive Response Technology |
| IUS | intrauterine hormone-releasing system |
| IV | intravenous |



Approved v1.0 930137673 4.0

| Term | Definition |
|---|---|
| KM | Kaplan-Meier |
| LAM | lactational amenorrhea method |
| LDH | lactate dehydrogenase |
| mAb | monoclonal antibody |
| Mg++ | magnesium |
| MG | myasthenia gravis |
| MID | minimally important difference |
| min | minute |
| MLR | mixed lymphocyte reaction |
| mmHg | millimeters of mercury |
| MMIS | malignant melanoma in situ |
| MMR | mismatch repair |
| [redacted] | |
| MRI | magnetic resonance imaging |
| mRNA | messenger ribonucleic acid |
| MRT | mean residence time |
| MSI-H | microsatellite instability-high |
| NCCN | National Comprehensive Cancer Network |
| NCI CTCAE | National Cancer Institute Common Terminology Criteria for Adverse Events |
| NCI-SEER | National Cancer Institute Surveillance, Epidemiology, and End Results Program |
| NED | no evidence of disease |
| ng | nanogram |
| [redacted] | |
| NSAID | nonsteroidal anti-inflammatory drug |
| NSCLC | non-small cell lung cancer |
| OS | overall survival |
| pAUCe | extrapolated partial AUC from last quantifiable concentration to infinity |
| Pb | percent of bound drug |



Approved v1.0 930137673 4.0

| Term | Definition |
|---|---|
| PBMC | peripheral blood mononuclear cell |
| [redacted] | |
| PD | pharmacodynamics |
| PD-L1 | programmed cell death ligand-1 |
| PEG-IFN | pegylated interferon |
| PET | positron emission tomography |
| PFS | progression-free survival |
| PFS2 | Progression-free survival through next-line therapy |
| PK | pharmacokinetics |
| PPK | population pharmacokinetics |
| [redacted] | |
| PT | prothrombin time |
| PTT | partial thromboplastin time |
| PVC | polyvinyl chloride |
| QD, qd | quaque die, once daily |
| [redacted] | |
| R2 | coefficient of determination |
| RBC | red blood cell |
| RCC | renal cell carcinoma |
| RFS | recurrence-free survival |
| [redacted] | |
| RT-PCR | reverse transcription polymerase chain reaction |
| SAD | short-axis diameter |
| SAE | serious adverse event |
| SARS-CoV-2 | severe acute respiratory syndrome coronavirus 2 |
| SCCHN | squamous cell carcinoma of the head and neck |
| SD | stable disease |
| SDV | source data verification |
| SEER | Surveillance, Epidemiology and End Results |
| SJS | Stevens-Johnson syndrome |



Approved v1.0 930137673 4.0

| Term | Definition |
|---|---|
| SLN | sentinel lymph node |
| SSC | Study Steering Committee |
| SUSAR | suspected unexpected serious adverse reaction |
| T.bili | total bilirubin |
| TCR | T-cell receptor |
| TEN | toxic epidermal necrolysis |
| [redacted] | |
| UK | United Kingdom |
| ULN | upper limit of normal |
| US | United States |
| VAS | visual analog scale |
| WBC | white blood cell |
| [redacted] | |
| WHO | World Health Organization |
| WLE | wide local excision |
| WNOCBP | women **not** of childbearing potential |
| WOCBP | women of childbearing potential |



Approved v1.0 930137673 4.0

# APPENDIX 2 STUDY GOVERNANCE CONSIDERATIONS

The terms "participant" and "subject" refer to a person who has consented to participate in the clinical research study. Typically, the term "participant" is used in the protocol and the term "subject" is used in the Case Report Form (CRF).

## REGULATORY AND ETHICAL CONSIDERATIONS

This study will be conducted in accordance with:

- Consensus ethical principles derived from international guidelines, including the Declaration of Helsinki and Council for International Organizations of Medical Sciences (CIOMS) International Ethical Guidelines
- Applicable International Council for Harmonisation (ICH) Good Clinical Practice (GCP) Guidelines
- Applicable laws, regulations, and requirements

The study will be conducted in compliance with the protocol. The protocol, any revisions/amendments, and the participant informed consent form (ICF) will receive approval/favorable opinion by Institutional Review Board/Independent Ethics Committee (IRB/IEC), and regulatory authorities according to applicable regulations prior to initiation of the study.

All potential serious breaches must be reported to the Sponsor or designee immediately. A potential serious breach is defined as a Quality Issue (eg, protocol deviation) that is likely to affect, to a significant degree, one or more of the following: (1) the rights, physical safety or mental integrity of one or more participants; (2) the scientific value of the clinical trial (eg, reliability and robustness of generated data). Items (1) or (2) can be associated with either GCP regulation(s) or trial protocol(s).

Personnel involved in conducting this study will be qualified by education, training, and experience to perform their respective tasks.

This study will not use the services of study personnel where sanctions have been invoked or where there has been scientific misconduct or fraud (eg, loss of medical licensure, debarment).

## INSTITUTIONAL REVIEW BOARD/INDEPENDENT ETHICS COMMITTEE

Before study initiation, the investigator must have written and dated approval/favorable opinion from the IRB/IEC for the protocol, Investigator's Brochure, product labeling information, ICF, participant recruitment materials (eg, advertisements), and any other written information to be provided to participants.

The investigator, Sponsor, or designee should provide the IRB/IEC with reports, updates, and other information (eg, expedited safety reports, amendments, administrative letters) annually, or more frequently, in accordance with regulatory requirements or institution procedures.

The investigator is responsible for providing oversight of the conduct of the study at the site and adherence to requirements of the following where applicable:



Approved v4.0 930137673 4.0

- ICH guidelines
- United States Code of Federal Regulations, Title 21, Part 50 (21CFR50)
- European Union Directive 2001/20/EC; or
- European Regulation 536/2014 for clinical studies (if applicable),
- European Medical Device Regulation 2017/745 for clinical device research (if applicable),
- the IRB/IEC
- and all other applicable local regulations

## COMPLIANCE WITH THE PROTOCOL AND PROTOCOL REVISIONS

The investigator should not implement any deviation or change to the protocol without prior review and documented approval/favorable opinion of an amendment from the IRB/IEC (and, if applicable, also by the local Health Authority), except where necessary to eliminate an immediate hazard(s) to study participants.

If a deviation or change to a protocol is implemented to eliminate an immediate hazard(s) prior to obtaining relevant approval/favorable opinion(s), the deviation or change will be submitted as soon as possible to:

- IRB/IEC
- Regulatory authority(ies), if applicable by local regulations (per national requirements)

Documentation of approval/favorable opinion signed by the chairperson or designee of the IRB(s)/IEC(s) and, if applicable, also by the local Health Authority, must be sent to Bristol-Myers Squibb (BMS).

If an amendment substantially alters the study design or increases the potential risk to the participant: (1) the ICF must be revised and submitted to the IRB(s)/IEC(s) for review and approval/favorable opinion; (2) the revised form must be used to obtain consent from participants currently enrolled in the study if they are affected by the amendment; and (3) the new form must be used to obtain consent from new participants prior to enrollment.

## FINANCIAL DISCLOSURE

Investigators and sub-investigators will provide the Sponsor with sufficient, accurate financial information, in accordance with regulations, to allow the Sponsor to submit complete and accurate financial certification or disclosure statements to the appropriate Health Authorities. Investigators are responsible for providing information on financial interests during the course of the study and for 1 year after completion of the study.

## INFORMED CONSENT PROCESS

Investigators must ensure that participants are clearly and fully informed about the purpose, potential risks, and other critical issues regarding clinical studies in which they volunteer to participate.



Approved v4.0 930137673 4.0

The Sponsor or designee will provide the investigator with an appropriate sample ICF, which will include all elements required by the ICH GCP, and applicable regulatory requirements. The sample ICF will adhere to the ethical principles that have their origin in the Declaration of Helsinki.

The investigator or his/her representative must:

- Obtain IRB/IEC written approval/favorable opinion of the written ICF and any other information to be provided to the participant prior to the beginning of the study and after any revisions are completed for new information.
- Provide a copy of the ICF and written information about the study in the language in which the participant is proficient prior to clinical study participation. The language must be nontechnical and easily understood.
- Explain the nature of the study to the participant or his/her legally acceptable representative and answer all questions regarding the study.
- Inform participant that his/her participation is voluntary. Participant or his/her legally acceptable representative will be required to sign a statement of informed consent that meets the requirements of 21 CFR 50, local regulations, ICH guidelines, Health Insurance Portability and Accountability Act (HIPAA) requirements, where applicable, and the IRB/IEC or study center.
- Allow time necessary for participant or his/her legally acceptable representative to inquire about the details of the study.

Obtain an ICF signed and personally dated by participant or his/her legally acceptable representative and by the person who conducted the informed consent discussion.

- Include a statement in participant's medical record that written informed consent was obtained before participant was enrolled in the study and the date the written consent was obtained. The authorized person obtaining the informed consent must also sign the ICF.
- Re-consent participant to the most current version of the ICF(s) during his/her participation in the study, as applicable.

Revise the ICF whenever important new information becomes available that is relevant to the participant's consent. The investigator, or a person designated by the investigator, should fully inform the participant or his/her legally acceptable representative of all pertinent aspects of the study and of any new information relevant to the participant's willingness to continue participation in the study. This communication should be documented.

The confidentiality of records that could identify participants must be protected, respecting the privacy and confidentiality rules applicable to regulatory requirements, the participant's signed ICF, and, in the US, the participant's signed HIPAA Authorization.

The ICF must also include a statement that BMS and local and foreign regulatory authorities have direct access to participant records.

In situations where consent cannot be given by participants, their legally acceptable representatives (as per country regulation) are clearly and fully informed about the purpose, potential risks, and other critical issues regarding clinical studies in which the participant volunteers to participate.



Approved v1.0 930137673 4.0

If informed consent is initially given by a participant's legally acceptable representative or legal guardian and the participant subsequently becomes capable of making and communicating his or her informed consent during the study, consent must additionally be obtained from the participant.

For minors, according to local legislation, one or both parents or a legally acceptable representative must be informed of the study procedures and must sign the ICF approved for the study prior to clinical study participation. The explicit wish of a minor, who is capable of forming an opinion and assessing this information to refuse participation in, or to be withdrawn from, the clinical study at any time, should be considered by the investigator.

Minors who are judged to be of an age of reason must also give their written consent. Minors who reach the age of majority (legal adulthood) during the clinical trial must give their written consent.

Participant unable to give their written informed consent (eg, stroke or participants with severe dementia) may only be enrolled in the study with the consent of a legally acceptable representative. The participant must also be informed about the nature of the study to the extent compatible with his or her understanding, and should this participant become capable, he or she should personally sign and date the ICF as soon as possible. The explicit wish of a participant who is unable to give his or her written consent, but who is capable of forming an opinion and assessing information to refuse participation in, or to be withdrawn from, the clinical study at any time, should be considered by the investigator.

The rights, safety, and well-being of the study participants are the most important considerations and should prevail over interests of science and society.

## SOURCE DOCUMENTS

Source documents provide evidence for the existence of the participant and substantiate the integrity of the data collected. Source documents are filed at the investigator's site.

Data reported on the CRF or entered in the electronic CRF (eCRF) that are transcribed from source documents must be consistent with the source documents or the discrepancies must be explained.

- The investigator may need to request previous medical records or transfer records, depending on the study. Also, current medical records must be available.
- Definitions of what constitutes source data can be found in the site procedure and document form.

The investigator is responsible for ensuring that the source data are accurate, legible, contemporaneous, original, and attributable, whether the data are handwritten on paper or entered electronically. If source data are created (first entered), modified, maintained, archived, retrieved, or transmitted electronically via computerized systems (and/or any other kind of electronic devices) as part of regulated clinical trial activities, such systems must be compliant with all applicable laws and regulations governing use of electronic records and/or electronic signatures. Such systems may include, but are not limited to, electronic medical records/electronic health records, adverse event (AE) tracking/reporting, protocol-required assessments, and/or drug accountability records.



Approved v4.0 930137673 4.0

When paper records from such systems are used in place of an electronic format to perform regulated activities, such paper records should be certified copies. A certified copy consists of a copy of original information that has been verified, as indicated by a dated signature, as an exact copy having all of the same attributes and information as the original.

## STUDY INTERVENTION RECORDS

Records for study intervention (whether supplied by BMS, its vendors, or the site) must substantiate study intervention integrity and traceability from receipt, preparation, administration, and through destruction or return. Records must be made available for review at the request of BMS/designee or a Health Authority.

| If | Then |
|---|---|
| Supplied by BMS (or its vendors): | Records or logs must comply with applicable regulations and guidelines and should include:<br>• amount received and placed in storage area<br>• amount currently in storage area<br>• label identification number or batch number<br>• amount dispensed to and returned by each participant, including unique participant identifiers<br>• amount transferred to another area/site for dispensing or storage<br>• nonstudy disposition (eg, lost, wasted)<br>• amount destroyed at study site, if applicable<br>• amount returned to BMS<br>• retain samples for bioavailability/bioequivalence/biocomparability, if applicable<br>• dates and initials of person responsible for Investigational Product dispensing/accountability, as per the Delegation of Authority Form |
| Sourced by site and not supplied by BMS or its vendors (examples include IP sourced from the sites stock or commercial supply or a specialty pharmacy) | The investigator or designee accepts responsibility for documenting traceability and study treatment integrity in accordance with requirements applicable under law and the standard operating procedures/standards of the sourcing pharmacy |

BMS or its designee will provide forms to facilitate inventory control if the investigational site does not have an established system that meets these requirements.



Approved v1.0 930137673 4.0

## CASE REPORT FORMS

An investigator is required to prepare and maintain adequate and accurate case histories designed to record all observations and other data pertinent to the investigation on each individual treated or entered as a control in the investigation. Data that are derived from source documents and reported on the CRF must be consistent with the source documents, or the discrepancies must be explained. Additional clinical information may be collected and analyzed in an effort to enhance understanding of product safety. CRFs may be requested for AEs and/or laboratory abnormalities that are reported or identified during the course of the study.

For sites using the Sponsor or designee electronic data capture (EDC) tool, eCRFs will be prepared for all data collection fields except for fields specific to SAEs and pregnancy, which will be reported on the electronic SAE form and Pregnancy Surveillance Form, respectively. If the electronic SAE form is not available, a paper SAE form can be used. Spaces may be left blank only in those circumstances permitted by study-specific CRF completion guidelines provided by the Sponsor or designee.

The confidentiality of records that could identify participants must be protected, respecting the privacy and confidentiality rules in accordance with the applicable regulatory requirement(s).

The investigator will maintain a signature sheet to document signatures and initials of all persons authorized to make entries and/or corrections on CRFs.

The completed CRF and SAE/pregnancy CRFs must be promptly reviewed, signed, and dated by the investigator or qualified physician who is a sub-investigator and who is delegated this task on the Delegation of Authority Form. Sub-investigators in Japan may not be delegated the CRF approval task. For eCRFs, review and approval/signature is completed electronically through the BMS EDC tool. The investigator must retain a copy of the CRFs, including records of the changes and corrections.

Each individual electronically signing eCRFs must meet Sponsor or designee training requirements and must only access the BMS EDC tool using the unique user account provided by the Sponsor or designee. User accounts are not to be shared or reassigned to other individuals.

## MONITORING

Monitoring details describing strategy, including definition of study critical data items and processes (eg, risk-based initiatives in operations and quality such as risk management and mitigation strategies and analytical risk-based monitoring), methods, responsibilities, and requirements, including handling of noncompliance issues and monitoring techniques (central, remote, or on-site monitoring) are provided in the monitoring plan.

Representatives of BMS must be allowed to visit all study site locations periodically to assess the data quality and study integrity. In those instances where BMS staff is restricted from visiting the site, remote source data verification (SDV) is allowed where access to electronic health records (EHRs) is permitted. On site, they will review study records and directly compare them with source documents, discuss the conduct of the study with the investigator, and verify that the facilities remain acceptable.



Approved v4.0 930137673 4.0

Certain CRF pages and/or electronic files may serve as the source documents.

In addition, the study may be evaluated by the Sponsor or designee internal auditors and government inspectors who must be allowed access to CRFs, source documents, other study files, and study facilities. BMS audit reports will be kept confidential.

The investigator must notify BMS promptly of any inspections scheduled by regulatory authorities and promptly forward copies of inspection reports to the Sponsor or designee.

## RECORDS RETENTION

The investigator (or head of the study site in Japan) must retain all study records and source documents for the maximum period required by applicable regulations and guidelines, or institution procedures, or for the period specified by BMS or its designee, whichever is longer. The investigator (or head of the study site in Japan) must contact BMS prior to destroying any records associated with the study.

BMS or its designee will notify the investigator (or head of the study site in Japan) when the study records are no longer needed.

If the investigator withdraws from the study (eg, relocation, retirement), the records shall be transferred to a mutually agreed-upon designee (eg, another investigator, study site, IRB). Notice of such transfer will be given in writing to BMS or its designee.

## RETURN OF STUDY TREATMENT

For this study, study treatments (those supplied by BMS or a vendor or sourced by the investigator), such as partially used study treatment containers, vials, and syringes, may be destroyed on site.

| If | Then |
|---|---|
| Study treatments supplied by BMS (including its vendors) | Any unused study interventions supplied by BMS can only be destroyed after being inspected and reconciled by the responsible Study Monitor, unless study treatments containers must be immediately destroyed as required for safety, or to meet local regulations (eg, cytotoxics or biologics).<br><br>Partially used study interventions and/or empty containers may be destroyed after proper reconciliation and documentation. But unused IMP must be reconciled by site monitor/Clinical Research Associate prior to destruction. |



Approved v1.0 930137673 4.0

| If | Then |
|---|---|
| | If study treatments will be returned, the return will be arranged by the responsible Study Monitor. |
| Study treatments sourced by site, not supplied by BMS (or its vendors; eg, study treatments sourced from the site's stock or commercial supply or a specialty pharmacy) | It is the investigator's or designee's responsibility to dispose of all containers according to the institutional guidelines and procedures. |

It is the investigator's or designee's responsibility to arrange for disposal of study interventions, provided that procedures for proper disposal have been established according to applicable federal, state, local, and institutional guidelines and procedures, and provided that appropriate records of disposal are kept. The following minimal standards must be met:

- On-site disposal practices must not expose humans to risks from the drug.
- On-site disposal practices and procedures are in agreement with applicable laws and regulations, including any special requirements for controlled or hazardous substances.
- Written procedures for on-site disposal are available and followed. The procedures must be filed with the site's standard operating procedures and a copy provided to BMS upon request.
- Records are maintained that allow for traceability of each container, including the date disposed of, quantity disposed, and identification of the person disposing the containers. The method of disposal (eg, incinerator, licensed sanitary landfill, or licensed waste-disposal vendor) must be documented.
- Accountability and disposal records are complete, up-to-date, and available for the Study Monitor to review throughout the clinical trial period.

It is the investigator's or designee's responsibility to arrange for disposal of all empty containers.

If conditions for destruction cannot be met, the responsible Study Monitor will make arrangements for return of study treatments provided by BMS (or its vendors). Destruction of non-study treatments sourced by the site, not supplied by BMS, is solely the responsibility of the investigator or designee.

## STUDY AND SITE START AND CLOSURE

The Sponsor/designee reserves the right to close the study site or to terminate the study at any time for any reason at the sole discretion of the Sponsor. Study sites will be closed upon study completion. A study site is considered closed when all required documents and study supplies have been collected and a study-site closure visit has been performed.

The investigator may initiate study-site closure at any time, provided there is reasonable cause and sufficient notice is given in advance of the intended termination.



Approved v1.0 930137673 4.0

Reasons for the early closure of a study site by the Sponsor or investigator may include, but are not limited to:

- Failure of the investigator to comply with the protocol, the requirements of the IRB/IEC or local Health Authorities, the Sponsor's procedures, or GCP guidelines
- Inadequate recruitment of participants by the investigator
- Discontinuation of further study intervention development

If the study is prematurely terminated or suspended, the Sponsor shall promptly inform the investigators, the IECs/IRBs, the regulatory authorities, and any contract research organization(s) used in the study of the reason for termination or suspension, as specified by the applicable regulatory requirements. The investigator shall promptly inform the participant and should assure appropriate participant therapy and/or follow-up.

## DISSEMINATION OF CLINICAL STUDY DATA

In order to benefit potential study participants, patients, healthcare providers and researchers, and to help BMS honor its commitments to study participants, BMS will make information about clinical research studies and a summary of their results available to the public as per regulatory and BMS requirements. BMS will post study information on local, national or regional databases in compliance with national and international standards for disclosure. BMS may also voluntarily disclose information to applicable databases.

In the European Union (EU), the summary of results and summary for laypersons will be submitted within 1 year (6 months if pediatric participants are enrolled) of the end of trial in EU/European Economic Area and third countries.

## CLINICAL STUDY REPORT

A Signatory Investigator must be selected to sign the Clinical Study Report (CSR).

For each CSR related to this protocol, the following criteria will be used to select the Signatory Investigator:

- External Principal Investigator designated at protocol development

## SCIENTIFIC PUBLICATIONS

The data collected during this study are confidential and proprietary to the Sponsor or designee. Any publications or abstracts arising from this study must adhere to the publication requirements set forth in the Clinical Trial Agreement (CTAg) governing [study site or investigator] participation in the study. These requirements include, but are not limited to, submitting proposed publications to the Sponsor or designee at the earliest practicable time prior to submission or presentation and otherwise within the time period set forth in the CTAg.

Scientific publications (such as abstracts, congress podium presentations and posters, and manuscripts) of the study results will be a collaborative effort between the study Sponsor and the



Approved v1.0 930137673 4.0

external authors. No public presentation or publication of any interim results may be made by any Principal Investigator, sub-investigator, or any other member of the study staff without the prior written consent of the Sponsor.

Authorship of publications at BMS is aligned with the criteria of the International Committee of Medical Journal Editors (ICMJE, www.icmje.org). Authorship selection is based upon significant contributions to the study (ie, ICMJE criterion #1). Authors must meet all 4 ICMJE criteria for authorship:

1) Substantial intellectual contribution to the conception or design of the work; or the acquisition of data (ie, evaluable participants with quality data), analysis, or interpretation of data for the work (eg, problem solving, advice, evaluation, insights and conclusion); AND
2) Drafting the work or revising it critically for important intellectual content; AND
3) Final approval of the version to be published; AND
4) Agreement to be accountable for all aspects of the work in ensuring that questions related to the accuracy or integrity of any part of the work are appropriately investigated and resolved.

Those who make the most significant contributions, as defined above, will be considered by BMS for authorship of the primary publication. Sub-investigators will generally not be considered for authorship in the primary publication. Geographic representation will also be considered.

Authors will be listed by order of significant contributions (highest to lowest), with the exception of the last author. Authors in first and last position have provided the most significant contributions to the work.

For secondary analyses and related publications, author list and author order may vary from primary to reflect additional contributions.



Approved v4.0 930137673 4.0

# APPENDIX 3 ADVERSE EVENTS AND SERIOUS ADVERSE EVENTS: DEFINITIONS AND PROCEDURES FOR RECORDING, EVALUATING, FOLLOW-UP, AND REPORTING

## ADVERSE EVENTS

| **Adverse Event Definition:** |
|---|
| An adverse event (AE) is defined as any new untoward medical occurrence or worsening of a pre-existing medical condition in a clinical investigation participant administered study treatment that does not necessarily have a causal relationship with this treatment. |
| An AE can therefore be any unfavorable and unintended sign (such as an abnormal laboratory finding), symptom, or disease temporally associated with the use of study treatment, whether or not considered related to the study treatment. |
| **Events Meeting the AE Definition** |
| • Any abnormal laboratory test results (hematology, clinical chemistry, or urinalysis) or results from other safety assessments (eg, electrocardiograms, radiological scans, vital signs measurements), including those that worsen from baseline, considered clinically significant in the medical and scientific judgment of the investigator. Note that abnormal lab tests or other safety assessments should only be reported as AEs if the final diagnosis is not available. Once the final diagnosis is known, the reported term should be updated to be the diagnosis.<br>• Exacerbation of a chronic or intermittent pre-existing condition, including either an increase in frequency and/or intensity of the condition.<br>• New conditions detected or diagnosed after study intervention administration, even though it may have been present before the start of the study.<br>• Signs, symptoms, or the clinical sequelae of a suspected drug-drug interaction.<br>• Signs, symptoms, or the clinical sequelae of a suspected overdose of either study intervention or a concomitant medication. Overdose, as a verbatim term (as reported by the investigator), should not be reported as an AE/serious adverse event (SAE) unless it is an intentional overdose taken with possible suicidal/self-harming intent. Such overdoses should be reported regardless of sequelae and should specify “intentional overdose” as the verbatim term. |
| **Events NOT Meeting the AE Definition** |
| • Medical or surgical procedure (eg, endoscopy, appendectomy); the condition that leads to the procedure is the AE.<br>• Situations in which an untoward medical occurrence did not occur (social and/or convenience admission to a hospital). |

## DEFINITION OF SAE

If an event is not an AE per definition above, then it cannot be an SAE, even if serious conditions are met.



Approved v4.0 930137673 4.0

## SERIOUS ADVERSE EVENTS

| **A serious adverse event (SAE) is defined as any untoward medical occurrence that, at any dose:** |
|---|
| Results in death. |
| Is life-threatening (defined as an event in which the participant was at risk of death at the time of the event; it does not refer to an event which hypothetically might have caused death if it were more severe). |
| Requires inpatient hospitalization or causes prolongation of existing hospitalization (see NOTE below).<br>NOTE:<br>The following hospitalizations are not considered SAEs in Bristol-Myers Squibb (BMS) clinical studies:<br>• A visit to the emergency room or other hospital department < 24 hours that does not result in admission (unless considered an important medical or life-threatening event).<br>• Elective surgery, planned prior to signing consent.<br>• Admissions as per protocol for a planned medical/surgical procedure.<br>• Routine health assessment requiring admission for baseline/trending of health status (eg, routine colonoscopy).<br>• Medical/surgical admission other than to remedy ill health and planned prior to entry into the study. Appropriate documentation is required in these cases.<br>• Admission encountered for another life circumstance that carries no bearing on health status and requires no medical/surgical intervention (eg, lack of housing, economic inadequacy, caregiver respite, family circumstances, administrative reason).<br>• Admission for administration of anticancer therapy in the absence of any other SAEs (applies to oncology protocols). |
| Results in persistent or significant disability/incapacity. |
| Is a congenital anomaly/birth defect. |
| Is an important medical event (defined as a medical event[s] that may not be immediately life-threatening or result in death or hospitalization but, based upon appropriate medical and scientific judgment, may jeopardize the participant or may require intervention [eg, medical, surgical] to prevent one of the other serious outcomes listed in the definition above). Examples of such events include, but are not limited to, intensive treatment in an emergency room or at home for allergic bronchospasm and blood dyscrasias or convulsions that do not result in hospitalization. Potential drug-induced liver injury (DILI) is also considered an important medical event. (See Section 9.2.7 for the definition of potential DILI.) |



Approved v1.0 930137673 4.0

Pregnancy and DILI must follow the same transmission timing and processes to BMS as used for SAEs. (See Section 9.2.5 for reporting pregnancies.)

Any component of a study endpoint that is considered related to study therapy should be reported as an SAE (eg, death is an endpoint; if death occurred due to anaphylaxis, anaphylaxis must be reported).

## EVALUATING AES AND SAES

| **Assessment of Causality** |
|---|
| • The investigator is obligated to assess the relationship between study intervention and each occurrence of each AE/SAE.<br>• A "reasonable possibility" of a relationship conveys that there are facts, evidence, and/or arguments to suggest a causal relationship, rather than a relationship cannot be ruled out.<br>• The investigator will use clinical judgment to determine the relationship.<br>• Alternative causes, such as underlying disease(s), concomitant therapy, and other risk factors, as well as the temporal relationship of the event to study intervention administration, will be considered and investigated.<br>• The investigator will also consult the Investigator's Brochure and/or product information for marketed products in his/her assessment.<br>• For each AE/SAE, the investigator must document in the medical notes that he/she has reviewed the AE/SAE and has provided an assessment of causality.<br>• There may be situations in which an SAE has occurred and the investigator has minimal information to include in the initial report to the Sponsor. However, it is very important that the investigator always make an assessment of causality for every event before the initial transmission of the SAE data to the Sponsor.<br>• The investigator may change his/her opinion of causality in light of follow-up information and send an SAE follow-up report with the updated causality assessment.<br>• The causality assessment is one of the criteria used when determining regulatory reporting requirements. |



Approved v4.0 930137673 4.0

| • **Assessment of Intensity** |
|---|
| The investigator will make an assessment of intensity for each AE and SAE reported during the study and assign it to 1 of the following categories:<br>• Mild: An event that is easily tolerated by the participant, causing minimal discomfort and not interfering with everyday activities.<br>• Moderate: An event that causes sufficient discomfort and interferes with normal everyday activities.<br>• Severe: An event that prevents normal everyday activities. An AE that is assessed as severe should not be confused with an SAE. Severe is a category utilized for rating the intensity of an event, and both AEs and SAEs can be assessed as severe.<br><br>An event is defined as "serious" when it meets at least 1 of the predefined outcomes as described in the definition of an SAE, NOT when it is rated as severe. |

| **Follow-up of AEs and SAEs** |
|---|
| If only limited information is initially available, follow-up reports are required. (Note: Follow-up SAE reports must include the same investigator term[s] initially reported.)<br>If an ongoing SAE changes in its intensity or relationship to study treatment or if new information becomes available, the SAE report must be updated and submitted within 24 hours to BMS (or designee) using the same procedure used for transmitting the initial SAE report.<br>All SAEs must be followed to resolution or stabilization. |



Approved v4.0 930137673 4.0

## REPORTING OF SAES TO SPONSOR OR DESIGNEE

- SAEs, whether related or not related to study treatment, and pregnancies must be reported to BMS (or designee) immediately within 24 hours of awareness of the event.
- SAEs must be recorded on the SAE Report Form.
  - The required method for SAE data reporting is through the electronic case report form (eCRF).
  - The paper SAE Report Form is intended only as a back-up option when the electronic data capture system is unavailable/not functioning for transmission of the eCRF to BMS (or designee).
    - ♦ In this case, the paper form is transmitted via email or confirmed facsimile transmission.
    - ♦ When paper forms are used, the original paper forms are to remain on site.
- Pregnancies must be recorded on paper Pregnancy Surveillance Forms and transmitted via email or confirmed facsimile transmission.

**SAE Email Address:** [redacted]

**SAE Facsimile Number:** Refer to Contact Information list.

**SAE Telephone Contact** (required for SAE and pregnancy reporting): Refer to Contact Information list.



Approved v4.0 930137673 4.0

# APPENDIX 4 WOMEN OF CHILDBEARING POTENTIAL DEFINITIONS AND METHODS OF CONTRACEPTION

Appendix 4 provides general information and definitions related to Woman of Childbearing Potential and methods of contraception that can be applied to most clinical trials. For information specific to this study regarding acceptable contraception requirements for female and male participants, refer to Section 6.1 of the protocol. Only the contraception methods as described in Section 6.1 are acceptable for this study.

## DEFINITIONS

**Woman of Childbearing Potential (WOCBP)**

A woman is considered fertile following menarche and until becoming postmenopausal unless permanently sterile. Permanent sterilization methods include hysterectomy, bilateral salpingectomy, and bilateral oophorectomy.

**Women in the following categories are not considered WOCBP:**

- Premenarchal
- Premenopausal female with 1 of the following:
  - Documented hysterectomy
  - Documented bilateral salpingectomy
  - Documented bilateral oophorectomy

Note: Documentation can come from the site personnel's review of the participant's medical records, medical examination, or medical history interview.

- Postmenopausal female
  - A postmenopausal state is defined as 12 months of amenorrhea in a woman over age 45 years in the absence of other biological or physiological causes. In addition, females under the age of 55 years must have a serum follicle-stimulating hormone (FSH) level > 40 mIU/mL to confirm menopause.

Note: Females treated with hormone replacement therapy (HRT) are likely to have artificially suppressed FSH levels and may require a washout period in order to obtain a physiologic FSH level. The duration of the washout period is a function of the type of HRT used. Suggested guidelines for the duration of the washout periods for HRT types are presented below. Investigators should use their judgement in checking serum FSH levels.

- 1-week minimum for vaginal hormonal products (rings, creams, gels)
- 4-week minimum for transdermal products
- 8-week minimum for oral products



Approved v1.0 930137673 4.0

Other parenteral products may require washout periods as long as 6 months. If the serum FSH level is > 40 mIU/mL at any time during the washout period, the woman can be considered postmenopausal.

**End of Relevant Systemic Exposure**

End of relevant systemic exposure is the timepoint where the Investigational Medicinal Product (IMP) or any active major metabolites have decreased to a concentration that is no longer considered to be relevant for human teratogenicity or fetotoxicity. This should be evaluated in context of safety margins from the no-observed-adverse-effect level or the time required for 5 half-lives of the IMP to pass.

## METHODS OF CONTRACEPTION

Local laws and regulations may require use of alternative and/or additional contraception methods.

| |
|---|
| **Highly Effective Contraceptive Methods That Are User Dependent**<br>*Failure rate of < 1% per year when used consistently and correctly.*[a] |
| • Combined (estrogen- and progestogen-containing) hormonal contraception associated with inhibition of ovulation and/or implantation. (This method of contraception can only be used by WOCBP participants in studies where hormonal contraception is permitted by the study protocol.)[b]<br>– Oral (birth control pills)<br>– Intravaginal (rings)<br>– Transdermal<br>• Combined (estrogen- and progestogen-containing) hormonal contraception must begin at least 30 days prior to initiation of study therapy. |
| • Progestogen-only hormonal contraception associated with inhibition of ovulation. (This method of contraception can only be used by WOCBP participants in studies where hormonal contraception is permitted by the study protocol.)[b]<br>– Oral<br>– Injectable<br>• Progestogen-only hormonal contraception must begin at least 30 days prior to initiation of study therapy. |
| **Highly Effective Methods That Are User Independent** |
| • Implantable progestogen-only hormonal contraception associated with inhibition of ovulation and/or implantation. (This method of contraception can only be used by WOCBP participants in studies where hormonal contraception is permitted by the study protocol.)[b]<br>• Intrauterine device. |



Approved v1.0 930137673 4.0

| |
|---|
| • Intrauterine hormone-releasing system (IUS). (This method of contraception can only be used by WOCBP participants in studies where hormonal contraception is permitted by the study protocol.)[b,c]<br>• Bilateral tubal occlusion. |
| • Vasectomized partner<br>A vasectomy is a highly effective contraception method provided that the participant is the sole male sexual partner of the WOCBP and the absence of sperm has been confirmed. If not, an additional highly effective method of contraception should be used. |
| • Sexual abstinence.<br>*Sexual abstinence is considered a highly effective method only if defined as refraining from heterosexual intercourse during the entire period of risk associated with the study treatment. The reliability of sexual abstinence needs to be evaluated in relation to the duration of the study and the preferred and usual lifestyle of the participant.*<br>• Continuous abstinence must begin at least 30 days prior to initiation of study therapy.<br>• It is not necessary to use any other method of contraception when complete abstinence is elected.<br>• WOCBP participants who choose complete abstinence must continue to have pregnancy tests, as specified in Section 2.<br>• Acceptable alternate methods of highly effective contraception must be discussed in the event that the WOCBP participant chooses to forego complete abstinence.<br>• Periodic abstinence (including, but not limited to, calendar, symptothermal, postovulation methods), withdrawal (coitus interruptus), spermicides only, and lactational amenorrhea method (LAM) are not acceptable methods of contraception for this study. |
| NOTES:<br>[a] Typical use failure rates may differ from failure rates when contraceptive methods are used consistently and correctly. Use should be consistent with local regulations regarding the use of contraceptive methods for participants in clinical studies.<br>[b] Hormonal contraception may be susceptible to interaction with the study treatment, which may reduce the efficacy of the contraceptive method. Hormonal contraception is permissible only when there is sufficient evidence that the IMP and other study medications will not alter hormonal exposures such that contraception would be ineffective or result in increased exposures that could be potentially hazardous. In this case, alternative methods of contraception should be utilized. For information specific to this study regarding permissibility of hormonal contraception, refer to Sections 6.1 INCLUSION CRITERIA and 7.7.1 PROHIBITED AND/OR RESTRICTED TREATMENTS of the protocol.<br>[c] IUSs are acceptable methods of contraception in the absence of definitive drug interaction studies when hormone exposures from intrauterine devices do not alter contraception effectiveness. For information specific to this study regarding permissibility of hormonal contraception, refer to Sections 6.1 INCLUSION CRITERIA and 7.7.1 PROHIBITED AND/OR RESTRICTED TREATMENTS of the protocol. |



Approved v1.0 930137673 4.0

**Less Than Highly Effective Contraceptive Methods That Are User Dependent**

*Failure rate of > 1% per year when used consistently and correctly.*

- Male or female condom with or without spermicide. Male and female condoms cannot be used simultaneously.
- Diaphragm with spermicide.
- Cervical cap with spermicide.
- Vaginal sponge with spermicide.
- Progestogen-only oral hormonal contraception, where inhibition of ovulation is not the primary mechanism of action. (This method of contraception cannot be used by WOCBP participants in studies where hormonal contraception is prohibited.)

**Unacceptable Methods of Contraception**

- Periodic abstinence (calendar, symptothermal, postovulation methods).
- Withdrawal (coitus interruptus).
- Spermicide only.
- LAM.

## COLLECTION OF PREGNANCY INFORMATION

Guidance for collection of pregnancy information and outcome of pregnancy on the Pregnancy Surveillance Form is provided in Section 9.2.5 and Appendix 3.



Approved v1.0 930137673 4.0

# APPENDIX 5 MANAGEMENT ALGORITHMS FOR STUDIES UNDER CTCAE VERSION 5.0

These general guidelines constitute guidance to the investigator and may be supplemented by discussions with the Medical Monitor representing the Sponsor. The guidance applies to all immuno-oncology agents and regimens.

A general principle is that differential diagnoses should be diligently evaluated according to standard medical practice. Non-inflammatory etiologies should be considered and appropriately treated.

Corticosteroids are a primary therapy for immuno-oncology drug-related adverse events. The oral equivalent of the recommended IV doses may be considered for ambulatory patients with low grade toxicity. The lower bioavailability of oral corticosteroids should be taken into account when switching to the equivalent dose of oral corticosteroids.

Consultation with a medical or surgical specialist, especially prior to an invasive diagnostic or procedure, is recommended.

The frequency and severity of the related adverse events covered by these algorithms will depend on the immuno-oncology agent or regimen being used.



Approved v1.0 930137673 4.0

# GI Adverse Event Management Algorithm

Rule out non-inflammatory causes. If non-inflammatory cause is identified, treat accordingly and continue I-O therapy.
Opiates/narcotics may mask symptoms of perforation. Infliximab should not be used in cases of perforation or sepsis.

| Grade of Diarrhea/ Colitis (NCI CTCAE v5) | Management | Follow-up |
|---|---|---|
| **Grade 1**<br>Diarrhea: < 4 stools/day over baseline<br>Colitis: asymptomatic | • Continue I-O therapy per protocol<br>• Symptomatic treatment | • Close monitoring for worsening symptoms<br>• Educate patient to report worsening immediately<br>If worsens:<br>• Treat as Grade 2 or Grade 3-4 |
| **Grade 2**<br>Diarrhea: 4-6 stools per day over baseline;<br>IV fluids indicated < 24 hrs;<br>Limiting instrumental ADL<br>Colitis: abdominal pain; blood in stool | • Delay I-O therapy per protocol<br>• Symptomatic treatment | If improves to Grade 1:<br>• Resume I-O therapy per protocol<br>If persists > 5-7 days or recurs:<br>• 0.5-1 mg/kg/day methylprednisolone or oral equivalent<br>• When symptoms improve to Grade 1, taper steroids over at least 1 month, consider prophylactic antibiotics for opportunistic infections, and resume I-O therapy per protocol.<br>If worsens or persists > 3-5 days with oral steroids:<br>• Treat as Grade 3-4 |
| **Grade 3-4**<br>Diarrhea (G3): ≥ 7 stools per day over baseline;<br>IV fluids ≥24 hrs;<br>limiting self care ADL<br>Colitis (G3): severe abdominal pain, peritoneal signs<br>G4: life-threatening, perforation | • Discontinue or delay I-O therapy per protocol*<br>• 1 to 2 mg/kg/day methylprednisolone IV or IV equivalent<br>• Add prophylactic antibiotics for opportunistic infections<br>• Consider lower endoscopy | If improves:<br>• Continue steroids until grade 1, then taper over at least 1 month<br>If persists > 3-5 days, or recurs after improvement:<br>• Add infliximab 5 mg/kg (if no contraindication) or other immunosuppressants.<br>• Consider testing CMV for steroid refractory colitis<br>Note: Infliximab should not be used in cases of perforation or sepsis |

Patients on IV steroids may be switched to an equivalent dose of oral corticosteroids (eg, prednisone) at start of tapering or earlier, after sustained clinical improvement is observed. Lower bioavailability of oral corticosteroids should be taken into account when switching to the equivalent dose of oral corticosteroids.

* Discontinue for Grade 4 diarrhea or colitis. For Grade 3 diarrhea or colitis, 1) Nivolumab monotherapy: Nivolumab can be delayed. 2) Nivolumab+ Ipilimumab combination: Ipilimumab should be discontinued while nivolumab can be delayed. Nivolumab monotherapy can be resumed when symptoms improve to Grade 1. Please refer to protocol for dose delay and discontinue criteria for other combinations.

28-Sep-2020



Approved v4.0 930137673 4.0

# Renal Adverse Event Management Algorithm

Rule out non-inflammatory causes. If non-inflammatory cause, treat accordingly and continue I-O therapy.

| Grade of Creatinine Elevation (NCI CTCAE v5) | Management | Follow-up |
|---|---|---|
| **Grade 1**<br>Creatinine > ULN to ≤ 1.5x ULN | • Continue I-O therapy per protocol<br>• Monitor creatinine weekly | If returns to baseline:<br>•Resume routine creatinine monitoring per protocol<br>If worsens:<br>• Treat as Grade 2 to Grade 4 |
| **Grade 2-3**<br>Creatinine > 1.5x baseline to ≤ 6x ULN | • Delay I-O therapy per protocol<br>• Monitor creatinine every 2-3 days<br>• 0.5 to 1 mg/kg/day methylprednisolone IV or oral equivalent<br>• Consider renal biopsy with nephrology consult | If returns to Grade 1:<br>•Taper steroids over at least 1 month, consider prophylactic antibiotics for opportunistic infections, and resume I-O therapy and routine creatinine monitoring per protocol<br>If elevations persist > 7 days or worsen:<br>•Treat as Grade 4 |
| **Grade 4**<br>Creatinine > 6x ULN | • Discontinue I-O therapy per protocol<br>• Monitor creatinine daily<br>• 1-2 mg/kg/day methylprednisolone IV or IV equivalent<br>• Consult nephrologist<br>• Consider renal biopsy | If returns to Grade 1: Taper steroids over at least 1 month and add prophylactic antibiotics for opportunistic infections<br><br>If no improvement or worsening, add additional immunosuppression. |

Patients on IV steroids may be switched to an equivalent dose of oral corticosteroids (eg, prednisone) at start of tapering or earlier, after sustained clinical improvement is observed. Lower bioavailability of oral corticosteroids should be taken into account when switching to the equivalent dose of oral corticosteroids.

28-Sep-2020

# Pulmonary Adverse Event Management Algorithm

Rule out non-inflammatory causes. If non-inflammatory cause, treat accordingly and continue I-O therapy.
Evaluate with imaging and pulmonary consultation.

| Grade of Pneumonitis (NCI CTCAE v5) | Management | Follow-up |
|---|---|---|
| **Grade 1**<br>Radiographic changes only | • Consider delay of I-O therapy<br>• Monitor for symptoms every 2-3 days<br>• Consider Pulmonary and ID consults | • Re-image at least every 3 weeks<br><br>If worsens:<br>• Treat as Grade 2 or 3-4 |
| **Grade 2**<br>Mild to moderate new symptoms | • Delay I-O therapy per protocol<br>• Pulmonary and ID consults<br>• Monitor symptoms daily, consider hospitalization<br>• 1 mg/kg/day methyl prednisolone IV or oral equivalent<br>• Consider bronchoscopy, lung biopsy | If improves to Grade 1:<br>• Resume I-O therapy per protocol<br>If persists > 5-7 days or recur:<br>• 0.5-1 mg/kg/day methylprednisolone or oral equivalent<br>• When symptoms improve to Grade 1, taper steroids over at least 1 month, consider prophylactic antibiotics for opportunistic infections, and resume I-O therapy per protocol.<br>If worsens or persists > 3-5 days with oral steroids:<br>• Treat as Grade 3-4 |
| **Grade 3-4**<br>Severe new symptoms; New/worsening hypoxia; Life-threatening | • Discontinue I-O therapy per protocol<br>• Hospitalize<br>• Pulmonary and ID consults<br>• 2-4 mg/kg/day methyl prednisolone IV or IV equivalent<br>• Add prophylactic antibiotics for opportunistic infections<br>• Consider bronchoscopy, lung biopsy | If improves to baseline:<br>• Taper steroids over at least 6 weeks<br><br>If not improving after 48 hours or worsening:<br>• Add additional immunosuppression |

Patients on IV steroids may be switched to an equivalent dose of oral corticosteroids (eg, prednisone) at start of tapering or earlier, after sustained clinical improvement is observed. Lower bioavailability of oral corticosteroids should be taken into account when switching to the equivalent dose of oral corticosteroids.

28-Sep-2020

# Hepatic Adverse Event Management Algorithm

Rule out non-inflammatory causes. If non-inflammatory cause, treat accordingly and continue I-O therapy.
Consider imaging for obstruction.

| Liver Function Test | Management | Follow-up |
| --- | --- | --- |
| AST or ALT > ULN to 3.0 x ULN and/or T. bili > ULN to 1.5 x ULN | • Continue I-O therapy per protocol<br>• Consider increasing frequency of monitoring | • Continue LFT monitoring per protocol<br><br>If worsens:<br>• Treat as below |
| AST or ALT > 3.0 to ≤ 5 x ULN and/or T. bili > 1.5 to ≤ 3 x ULN | • Delay I-O therapy per protocol<br>• Increase frequency of monitoring to every 3 days | If returns to baseline:<br>•Resume routine monitoring, resume I-O therapy per protocol<br><br>If elevations persist > 5-7 days or worsen:<br>• 0.5-1 mg/kg/day methylprednisolone or oral equivalent and when and AST ALT returns to <3X ULN and bili <1.5X ULN, taper steroids over at least 1 month, consider prophylactic antibiotics for opportunistic infections, and resume I-O therapy per protocol |
| AST or ALT > 5 x ULN or T.bili >3 x ULN | • Delay or Discontinue I-O therapy per protocol^<br>• Increase frequency of monitoring to every 1-2 days<br>• 1 to 2 mg/kg/day methylprednisolone IV or IV equivalent*<br>• Add prophylactic antibiotics for opportunistic infections<br>• Consult gastroenterologist or hepatologist | If AST and ALT returns to <5X ULN and bili <3X ULN,<br>• Taper steroids over at least 1 month<br><br>If does not improve in >3-5 days, worsens, or rebounds:<br>• Add mycophenolate mofetil 1 g BID<br>• If no response within an additional 3-5 days, consider tacrolimus or other immunosuppressants per local guidelines<br>Note: Avoid infliximab due to potential risk of liver failure |

Patients on IV steroids may be switched to an equivalent dose of oral corticosteroids (e.g. prednisone) at start of tapering or earlier, after sustained clinical improvement is observed. Lower bioavailability of oral corticosteroids should be taken into account when switching to the equivalent dose of oral corticosteroids.
^ Please refer to protocol dose delay and discontinue criteria for specific details.
*The recommended starting dose for AST or ALT > 20 x ULN or bilirubin >10 x ULN is 2 mg/kg/day methylprednisolone IV.

28-Sep-2020

# Endocrinopathy Adverse Event Management Algorithm

Rule out non-inflammatory causes. If non-inflammatory cause, treat accordingly and continue I-O therapy.
Consider visual field testing, endocrinology consultation, and imaging.

| Endocrinopathy | Management | Follow-up |
|---|---|---|
| Asymptomatic TSH abnormality<br>or<br>Hyperglycemia requiring no medical intervention | • Continue I-O therapy per protocol<br>• If TSH < 0.5 x LLN, or TSH > 2 x ULN, or consistently out of range in 2 subsequent measurements:<br>○ include fT4 at subsequent cycles as clinically indicated;<br>○ consider endocrinology consult | • Monitor TSH as per protocol<br>• Monitor blood sugar<br>• If symptomatic or blood sugar requiring initiation of treatment or change in management, manage as below |
| Symptomatic endocrinopathy<br>or<br>hyperglycemia requiring initiation of treatment or change in daily management or work up | • Evaluate endocrine function; consider endocrinology consult<br>• Consider pituitary scan<br>Symptomatic with abnormal lab/pituitary scan:<br>• Delay I-O therapy per protocol<br>• 1-2 mg/kg/day methylprednisolone IV or PO equivalent for hypophysitis<br>• Initiate appropriate hormone/medical therapy<br>No abnormal lab/pituitary MRI scan but symptoms persist:<br>• Continue IO therapy. Repeat labs in 1-3 weeks /MRI in 1 month<br>Hyperglycemia:<br>Screen for Diabetic Ketoacidosis (DKA). If negative, continue IO and treat diabetes. If DKA hold IO, admit, manage DKA. Steroids not recommended for DKA/hyperglycemia | If improves (with or without hormone replacement):<br>• Taper steroids over at least 1 month and consider prophylactic antibiotics for opportunistic infections<br>• Resume I-O therapy per protocol<br>• Patients with adrenal insufficiency may need to continue steroids with mineralocorticoid component |
| Suspicion of adrenal crisis (eg, severe dehydration, hypotension, shock out of proportion to current illness) | • Delay or discontinue I-O therapy per protocol<br>• Rule out sepsis<br>• Stress dose of IV steroids with mineralocorticoid activity<br>• IV fluids<br>• Consult endocrinologist | • Patients with adrenal insufficiency may need to continue steroids with mineralocorticoid component<br>• If adrenal crisis resolves and patient is adequately controlled with physiologic hormone replacement, patient can resume IO therapy per protocol. |

Patients on IV steroids may be switched to an equivalent dose of oral corticosteroids (eg, prednisone) at start of tapering or earlier, after sustained clinical improvement is observed. Lower bioavailability of oral corticosteroids should be taken into account when switching to the equivalent dose of oral corticosteroids.

28-Sep-2020



Approved v4.0 930137673 4.0

# Skin Adverse Event Management Algorithm

Rule out non-inflammatory causes. If non-inflammatory cause, treat accordingly and continue I-O therapy.

| Rash | Management | Follow-up |
|---|---|---|
| Covering ≤ 30% BSA* | • Symptomatic therapy (eg, antihistamines, topical steroids)<br>• Continue I-O therapy per protocol | <u>If persists > 1-2 weeks or recurs:</u><br>• Consider skin biopsy<br>• Delay I-O therapy per protocol<br>• Consider 0.5-1.0 mg/kg/day methylprednisolone IV or oral equivalent. Once improving , taper steroids over at least 1 month, consider prophylactic antibiotics for opportunistic infections, and resume I-O therapy per protocol<br><u>If worsens:</u><br>• Treat as >30% BSA |
| Covering > 30% BSA (with or without symptoms)<br><br>Life threatening consequences*^ | • Delay or discontinue I-O therapy per protocol<br>• Consider skin biopsy<br>• Dermatology consult<br>• 1.0 -2.0 mg/kg/day IV methylprednisolone IV or oral equivalent | <u>If improves to < 10% BSA:</u><br>• Taper steroids over at least 1 month and add prophylactic antibiotics for opportunistic infections<br>• Resume I-O therapy per protocol |

Patients on IV steroids may be switched to an equivalent dose of oral corticosteroids (e.g. prednisone) at start of tapering or earlier, after sustained clinical improvement is observed. Lower bioavailability of oral corticosteroids should be taken into account when switching to the equivalent dose of oral corticosteroids.

*Refer to NCI CTCAE v5 for term-specific grading criteria.

^If Steven-Johnson Syndrome (SJS),toxic epidermal necrosis (TEN), Drug Reaction with Eosinophilia and Systemic Symptoms (DRESS) is suspected, withhold I-O therapy and refer patient for specialized care for assessment and treatment. If SJS, TEN, or DRESS is diagnosed, permanently discontinue I-O therapy.

28-Sep-2020

# Neurological Adverse Event Management Algorithm

Rule out non-inflammatory causes. If non-inflammatory cause, treat accordingly and continue I-O therapy.

| Grade of Neurological Toxicity (NCI CTCAE v5) | Management | Follow-up |
|---|---|---|
| **Grade 1**<br>Asymptomatic or mild symptoms;<br>Intervention not indicated | • Continue I-O therapy per protocol<br>• Discontinue I-O for select AEs^ | • Continue to monitor per protocol.<br>If worsens:<br>• Treat as Grade 2 or Grade 3-4 |
| **Grade 2**<br>Moderate symptoms;<br>Limiting instrumental ADL | • Delay I-O therapy per protocol<br>• Discontinue I-O for select AEs^<br>• Consider neurology consult<br>• Treat symptoms per local guidelines<br>• Consider 0.5 to 1 mg/kg/day methylprednisolone IV or PO equivalent | If returns to baseline:<br>• Resume I-O therapy per protocol when improved to baseline If worsens:<br>• Treat as Grade 3-4 |
| **Grade 3-4**<br>Severe symptoms;<br>Limiting self-care ADL;<br>Life-threatening | • Discontinue I-O therapy per protocol<br>• Obtain neurology consult<br>• Treat symptoms per local guidelines<br>• 1-2 mg/kg/day IV methylprednisolone IV or IV equivalent<br>• Add prophylactic antibiotics for opportunistic infections | If improves to Grade 2:<br>• Taper steroids over at least 1 month<br><br>If worsens or atypical presentation:<br>• Consider IVIG or other immunosuppressive therapies per local guidelines |

Patients on IV steroids may be switched to an equivalent dose of oral corticosteroids (eg. prednisone) at start of tapering or earlier, after sustained clinical improvement is observed. Lower bioavailability of oral corticosteroids should be taken into account when switching to the equivalent dose of oral corticosteroids.

^Discontinue for any grade myasthenia gravis, Guillain-Barre syndrome, treatment-related myelitis, or encephalitis.

28-Sep-2020

# Myocarditis Adverse Event Management Algorithm

Rule out non-inflammatory causes. If non-inflammatory cause, treat accordingly and continue I-O therapy.

| Myocarditis | Management | Follow-up |
|---|---|---|
| Symptoms with mild to moderate activity or exertion | • Delay I-O therapy; hospitalization with cardiac monitoring<br>• Urgent cardiology consultation for evaluation and management<br>  ○ Troponin and BNP<br>  ○ ECG ± continuous cardiac monitoring<br>  ○ Echocardiogram<br>  ○ Cardiac MRI<br>• Prompt initiation of 2 mg/kg/day methylprednisolone IV or equivalent | • If worsens, intensify treatment according to grade<br>• Upon recovery, taper steroids over at least 1 month with close monitoring of troponin and BNP. as well as for new symptoms<br>• Repeat cardiac MRI for post treatment assessment and cardiology follow-up<br>• Retreatment may be considered after recovery and completion of steroid taper |
| Severe with symptoms at rest or with minimal activity or exertion; intervention indicated<br><br>Life-threatening consequences; urgent intervention indicated (e.g., continuous IV therapy or mechanical hemodynamic support) | • Permanently discontinue I-O therapy<br>• Hospitalize to intensive cardiac monitoring<br>• Cardiac evaluation to include:<br>  ○ Troponin and B-type natriuretic peptide monitoring<br>  ○ ECG ± continuous cardiac monitoring<br>  ○ Echocardiogram<br>  ○ Cardiac MRI<br>  ○ Myocardial biopsy if feasible<br>• Immediate initiation of 2 mg/kg/day methylprednisolone IV or 1 g IV bolus<br>• Consider adding a second immunosuppressive agent<br>Additionally, for life threatening myocarditis:<br>• Hospitalize/transfer to institution with expertise in intensive cardiac monitoring<br>• Consider anti-thymocyte globulin as second agent given its immediate effect | • If no improvement, consider additional immunosuppression<br>• Upon recovery, taper steroids over at least 1 month with close monitoring of troponin and BNP as well as for new symptoms<br>• Repeat cardiac MRI for post treatment assessments and cardiology follow-up |

Patients on IV steroids may be switched to an equivalent dose of oral corticosteroids (eg, prednisone) at start of tapering or earlier, after sustained clinical improvement is observed. Lower bioavailability of oral corticosteroids should be taken into account when switching to the equivalent dose of oral corticosteroids.
Prophylactic antibiotics should be considered in the setting of ongoing immunosuppression.

28-Sep-2020

## APPENDIX 6 ECOG PERFORMANCE STATUS

These scales are used by doctors and researchers to assess how a patient's disease is progressing, assess how the disease affects the daily living abilities of the patient, and determine appropriate treatment and prognosis. They are included here for healthcare professionals to assess.

| ECOG PERFORMANCE STATUS | |
|---|---|
| 0 | Fully active, able to carry on all pre-disease performance without restriction |
| 1 | Restricted in physically strenuous activity but ambulatory and able to carry out work of a light or sedentary nature, eg, light house work, office work |
| 2 | Ambulatory and capable of all selfcare but unable to carry out any work activities. Up and about more than 50% of waking hours |
| 3 | Capable of only limited selfcare, confined to bed or chair more than 50% of waking hours |
| 4 | Completely disabled. Cannot carry on any selfcare. Totally confined to bed or chair |
| 5 | Dead |

Toxicity and Response Criteria of the Eastern Cooperative Oncology Group.


Approved v4.0 930137673 4.0

# APPENDIX 7 RESPONSE EVALUATION CRITERIA IN SOLID TUMORS GUIDELINES (VERSION 1.1) WITH BMS MODIFICATIONS

## 1 EVALUATION OF LESIONS

Solid tumors will be evaluated using Response Evaluation Criteria In Solid Tumors version 1.1 (RECIST 1.1) guideline with BMS modifications.[1]

At baseline, tumor lesions/lymph nodes will be categorized as measurable or non-measurable as follows:

### 1.1 Measurable

**Tumor lesions:** Must be accurately measured in at least one dimension (longest diameter in the plane of measurement is to be recorded) with a minimum size of:

- 10 mm by CT/MRI scan (scan slice thickness no greater than 5 mm), or ≥ 2x slice thickness if greater than 5mm.

**Malignant lymph nodes:** To be considered pathologically enlarged and measurable, a lymph node must be ≥ 15 mm in short axis when assessed by CT/MRI scan (scan slice thickness recommended to be no greater than 5 mm).

Lymph nodes merit special mention since they are normal anatomical structures which may be visible by imaging even if not involved by tumor. Pathological nodes which are defined as measurable and may be identified as target lesions must meet the criterion of a short axis of ≥ 15 mm by CT/MRI scan. Only the short axis of these nodes will contribute to the baseline sum. The short axis of the node is the diameter normally used by radiologists to judge if a node is involved by solid tumor. Nodal size is normally reported as two dimensions in the plane in which the image is obtained (for CT scan this is almost always the axial plane; for MRI the plane of acquisition may be axial, sagittal or coronal). The smaller of these measures is the short axis. For example, an abdominal node which is reported as being 20 mm x 30 mm has a short axis of 20 mm and qualifies as a malignant, measurable node. In this example, 20 mm should be recorded as the node measurement. All other pathological nodes (those with short axis ≥ 10 mm but < 15 mm) should be considered non-target lesions. Nodes that have a short axis < 10 mm are considered non-pathological and should not be recorded or followed.

Note: Lesions on X-Ray are not to be selected as Target or Non-Target Lesions.

### 1.2 Non-Measurable

All other lesions are considered non-measurable, including small lesions (longest diameter < 10mm or pathological lymph nodes with ≥ 10 to < 15 mm short axis) as well as truly non-measurable lesions. Lesions considered truly non-measurable include: leptomeningeal disease, inflammatory breast disease, lymphangitic involvement of skin or lung, abdominal masses/abdominal organomegaly identified by physical exam that is not measurable by reproducible imaging techniques.



Approved v4.0 930137673 4.0

Note: Lesions on X-Ray are not to be selected as Target or Non-Target Lesions.

### 1.3 Special considerations regarding lesion measurability

#### *1.3.1 Bone lesions*

- Bone scan, PET scan and plain films are ***not*** considered adequate imaging techniques to measure bone lesions. However, these techniques can be used to confirm the presence or disappearance of bone lesions.
- Lytic bone lesions or mixed lytic-blastic lesions, with *identifiable soft tissue components*, that can be evaluated by cross sectional imaging techniques such as CT or MRI can be considered as measurable lesions if the *soft tissue component* meets the definition of measurability described above.
- Blastic bone lesions are non-measurable.

### 1.4 Baseline Documentation Of 'Target' And 'Non-Target' Lesions

When more than one measurable lesion is present at baseline all lesions up to a maximum of five lesions total (and a maximum of two lesions per organ) representative of all involved organs should be identified as target lesions and will be recorded and measured at baseline (this means in instances where patients have only one or two organ sites involved a maximum of two and four lesions respectively will be recorded).

Note: A maximum of two lesions can be selected per organ system. For example, a maximum of two lung lesions can be selected (selected from one lung or one lesion from each). A maximum of two lymph nodes can be selected at baseline, as the lymphatic system is considered one organ.

Target lesions should be selected on the basis of their size (lesions with the longest diameter), be representative of all involved organs, but in addition should be those that lend themselves to reproducible repeated measurements. It may be the case that, on occasion, the largest lesion does not lend itself to reproducible measurement in which circumstance the next largest lesion which can be measured reproducibly should be selected.

A sum of the diameters (longest for non-nodal lesions, short axis for nodal lesions) for all target lesions will be calculated and reported as the baseline sum diameters. If lymph nodes are to be included in the sum, then as noted above, only the short axis is added into the sum. The baseline sum diameters will be used as reference to further characterize any objective tumor regression in the measurable dimension of the disease.

All other lesions (or sites of disease) including pathological lymph nodes should be identified as non-target lesions and should also be recorded at baseline. Measurements are not required and these lesions should be followed as 'present', 'absent', or in rare cases 'unequivocal progression' (more details to follow). In addition, it is possible to record multiple non-target lesions involving the same organ as a single item on the case record form (eg, 'multiple enlarged pelvic lymph nodes' or 'multiple liver metastases').



Approved v1.0 930137673 4.0

# 2 RESPONSE CRITERIA

## 2.1 Evaluation of Target Lesions

- **Complete Response (CR):** Disappearance of all target lesions. Any pathological lymph nodes (whether target or non-target) must have reduction in short axis to < 10 mm.
- **Partial Response (PR):** At least a 30% decrease in the sum of diameters of target lesions, taking as reference the baseline sum diameters.
- **Progressive Disease (PD):** At least a 20% increase in the sum of diameters of target lesions, taking as reference the smallest sum on study (this includes the baseline sum if that is the smallest on study). In addition to the relative increase of 20%, the sum must also demonstrate an absolute increase of at least 5 mm. (Note: the appearance of one or more new lesions is also considered progression).
- **Stable Disease (SD):** Neither sufficient shrinkage to qualify for PR nor sufficient increase to qualify for PD, taking as reference the smallest sum diameters while on study.
- **Not Evaluable (NE):** If one or more target lesions cannot be measured or adequately assessed as either fully resolved or too small to measure (due to missing or poor quality images), and the sum of diameters of the remaining measured target lesions (if any) has not increased sufficiently to meet Progressive Disease as defined above.

### *2.1.1 Special Notes on the Assessment of Target Lesions*

#### *2.1.1.1 Lymph nodes*

Lymph nodes identified as target lesions should always have the actual short axis measurement recorded (measured in the same anatomical plane as the baseline examination), even if the nodes regress to below 10 mm on study. This means that when lymph nodes are included as target lesions, the 'sum' of lesions may not be zero even if complete response criteria are met, since a normal lymph node is defined as having a short axis of < 10 mm. Case report forms or other data collection methods may therefore be designed to have target nodal lesions recorded in a separate section where, in order to qualify for CR, each node must achieve a short axis < 10 mm. For PR, SD and PD, the actual short axis measurement of the nodes is to be included in the sum of target lesions.

#### *2.1.1.2 Target lesions that become 'too small to measure'*

While on study, all lesions (nodal and non-nodal) recorded at baseline should have their actual measurements recorded at each subsequent evaluation, even when very small (eg, 2 mm). However, sometimes lesions or lymph nodes which are recorded as target lesions at baseline become so faint on CT scan that the radiologist may not feel comfortable assigning an exact measure and may report them as being 'too small to measure'. When this occurs it is important that a value be recorded on the case report form. If it is the opinion of the radiologist that the lesion has likely disappeared, the measurement should be recorded as 0 mm. If the lesion is believed to be present and is faintly seen but too small to measure, a default value of 5 mm should be assigned as the reference diameter. (Note: It is less likely that this rule will be used for lymph nodes since they usually have a definable size when normal and are frequently surrounded by fat such as in the retroperitoneum; however, if a lymph node is believed to be present and is faintly seen but too


Approved v1.0 930137673 4.0

small to measure, a default value of 5 mm should be assigned in this circumstance as well). This default value is derived from the 5 mm CT slice thickness (but should not be changed with varying CT slice thickness). The measurement of these lesions is potentially non-reproducible, therefore providing this default value will prevent false responses or progressions based upon measurement error. To reiterate, however, if the radiologist is able to provide an actual measure, that should be recorded, even if it is below 5 mm.

#### *2.1.1.3 Lesions that split or coalesce on treatment*

When non-nodal lesions 'fragment', the longest diameters of the fragmented portions should be added together to calculate the target lesion sum. Similarly, as lesions coalesce, a plane between them may be maintained that would aid in obtaining maximal diameter measurements of each individual lesion. If the lesions have truly coalesced such that they are no longer separable, the vector of the longest diameter in this instance should be the maximal longest diameter for the 'coalesced lesion'.

## 2.2 Evaluation of Non-Target Lesions

This section provides the definitions of the criteria used to determine the tumor response for the group of non-target lesions. While some non-target lesions may actually be measurable, they need not be measured and instead should be assessed only qualitatively at the time points specified in the protocol.

- **Complete Response (CR):** Disappearance of all non-target lesions. All lymph nodes must be non-pathological in size (< 10mm short axis).
- **Non-CR/Non-PD:** Persistence of one or more non-target lesion(s)
- **Progressive Disease (PD):** Unequivocal progression of existing non-target lesions.

### *2.2.1 Special Notes on Assessment of Progression of Non-Target Disease*

The concept of progression of non-target disease requires additional explanation as follows:

#### *2.2.1.1 When the patient also has measurable disease*

In this setting, to achieve 'unequivocal progression' on the basis of the non-target disease, there must be an overall level of substantial worsening in non-target disease such that, even in presence of SD or PR in target disease, the overall tumor burden has increased sufficiently to merit discontinuation of therapy A modest 'increase' in the size of one or more non-target lesions is usually not sufficient to qualify for unequivocal progression status. Pleural effusions, pericardial effusions and ascites will not be followed as target or non-target lesions and will not contribute to response or progression. The designation of overall progression solely on the basis of change in non-target disease in the face of SD or PR of target disease will therefore be extremely rare.

#### *2.2.1.2 When the patient has only non-measurable disease*

This circumstance arises in some trials when it is not a criterion of study entry to have measurable disease. The same general concepts apply here as noted above, however, in this instance there is no measurable disease assessment to factor into the interpretation of an increase in non-measurable



Approved v1.0 930137673 4.0

disease burden. Because worsening in non-target disease cannot be easily quantified (by definition: if all lesions are truly non-measurable) a useful test that can be applied when assessing patients for unequivocal progression is to consider if the increase in overall disease burden based on the change in non-measurable disease is comparable in magnitude to the increase that would be required to declare PD for measurable disease: ie, an increase in tumor burden representing an additional 73% increase in 'volume' (which is equivalent to a 20% increase diameter in a measurable lesion). Examples include, an increase in lymphangitic disease from localized to widespread, or may be described as 'sufficient to require a change in therapy'. If 'unequivocal progression' is seen, the patient should be considered to have had overall PD at that point. While it would be ideal to have objective criteria to apply to non-measurable disease, the very nature of that disease makes it impossible to do so; therefore the increase must be substantial.

### 2.2.2 New Lesions

The appearance of new malignant lesions denotes disease progression; therefore, some comments on detection of new lesions are important. There are no specific criteria for the identification of new radiographic lesions; however, the finding of a new lesion should be unequivocal: ie, not attributable to differences in scanning technique, change in imaging modality or findings thought to represent something other than tumor (for example, some 'new' bone lesions may be simply healing or flare of pre-existing lesions). This is particularly important when the patient's baseline lesions show partial or complete response. For example, necrosis of a liver lesion may be reported on a CT scan report as a 'new' cystic lesion, which it is not.

NOTE: Fluid collections (pleural effusions, pericardial effusions, and ascites) will not be considered new lesions and will not contribute to response or progression. In the event a new fluid collection is seen on a post-baseline imaging exam, a comment may be made, but the appearance of a new fluid collection alone should not result in an assessment of Progressive Disease (PD). A lesion identified on a follow-up study in an anatomical location that was not scanned at baseline is considered a new lesion and will indicate disease progression. An example of this is the patient who has visceral disease at baseline and while on study has a CT or MRI brain ordered which reveals metastases. The patient's brain metastases are considered to be evidence of PD even if he/she did not have brain imaging at baseline. A lesion identified on Chest X-Ray that was not present in prior CT can be considered a new lesion and will result in Progressive Disease (PD).

If a new lesion is equivocal, for example because of its small size, continued follow-up evaluation will clarify if it represents truly new disease. If repeat scans confirm there is definitely a new lesion, then progression should be declared using the date of the initial scan. While FDG-PET response assessments need additional study, it is sometimes reasonable to incorporate the use of FDG-PET scanning to complement CT scanning in assessment of progression (particularly possible 'new' disease). New lesions on the basis of FDG-PET imaging can be identified according to the following algorithm:



Approved v4.0 930137673 4.0

1. Negative FDG-PET at baseline, with a positive FDG-PET at follow-up is a sign of PD based on a new lesion.
2. No FDG-PET at baseline and a positive FDG-PET at follow-up: If the positive FDG-PET at follow-up corresponds to a new site of disease confirmed by CT, this is PD. If the positive FDG-PET at follow-up is not confirmed as a new site of disease on CT, additional follow-up CT scans are needed to determine if there is truly progression occurring at that site (if so, the date of PD will be the date of the initial abnormal FDG-PET scan). If the positive FDG-PET at follow-up corresponds to a pre-existing site of disease on CT that is not progressing on the basis of the anatomic images, this is not PD.

## 2.3 Response Assessment

### *2.3.1 Evaluation of Best Overall Response*

The best overall response is the best response recorded from the start of the study treatment until disease progression or the last response recorded, taking into account any requirement for confirmation and censoring rules regarding subsequent therapy. The patient's best overall response assignment will depend on the findings of both target and non-target disease and will also take into consideration the appearance of new lesions. Furthermore, depending on the nature of the study and the protocol requirements, it may also require confirmatory measurement.

### *2.3.2 Time Point Response*

At each protocol specified time point, a response assessment occurs. Table 2.3.2-1 provides a summary of the overall response status calculation at each time point for patients who have measurable disease at baseline. When patients have non-measurable (therefore non-target) disease only, Table 2.3.2-2 is to be used.

**Table 2.3.2-1: Time Point Response: Patients With Target (± Non-Target) Disease**

| Target Lesions | Non-Target Lesions | New Lesions | Overall Response |
|---|---|---|---|
| CR | CR | No | CR |
| CR | Non-CR/non-PD | No | PR |
| CR | Not evaluated | No | PR |
| PR | Non-PD or not all evaluated | No | PR |
| SD | Non-PD or not all evaluated | No | SD |
| Not all evaluated | Non-PD | No | NE |
| PD | Any | Yes or No | PD |
| Any | PD | Yes or No | PD |
| Any | Any | Yes | PD |

CR = complete response, PR = partial response, SD = stable disease, PD = progressive disease and NE = inevaluable



Approved v1.0 930137673 4.0

| Table 2.3.2-2: Time Point Response: Patients with Non-target Disease Only | | |
|---|---|---|
| **Non-Target Lesions** | **New Lesions** | **Overall Response** |
| CR | No | CR |
| Non-CR/non-PD | No | Non-CR/non-PD[a] |
| Not all evaluated | No | NE |
| Unequivocal PD | Yes or No | PD |
| Any | Yes | PD |
| CR = complete response, PD = progressive disease and NE = inevaluable | | |

[a] Non-CR/non-PD is preferred over SD for non-target disease since SD is increasingly used as endpoint for assessment of efficacy in some trials so to assign this category when no lesions can be measured is not advised.

### 2.3.3 Best Overall Response

Best response determination of complete or partial response requires confirmation: Complete or partial responses may be claimed only if the criteria for each are met at a subsequent time point of ≥ 4 weeks (28 days) later. In this circumstance, the best overall response can be interpreted as in Table 2.3.3-1. When SD is believed to be best response, it must meet the protocol specified minimum time from the date of first treatment or randomization date.

For example, if the first scheduled follow-up imaging visit is Week 6 (± 7 days) for a particular protocol, a Best Response of SD can only be made after the participant is on-study for a minimum of 6 weeks (42 days) minus 7 days, for an absolute minimum time on-study of 35 days from the reference start date (reference date is considered Day 1 on study). If the participant is not on-study for at least this amount of time, any tumor assessment indicating stable disease before this time period will have a Best Response of NE unless PD is identified.

**Special note on response assessment:** When nodal disease is included in the sum of target lesions and the nodes decrease to 'normal' size (< 10 mm), they may still have a measurement reported on scans. This measurement should be recorded even though the nodes are normal in order not to overstate progression should it be based on increase in size of the nodes. As noted earlier, this means that patients with CR may not have a total sum of 'zero' on the case report form (CRF).

| Table 2.3.3-1: Best Overall Response (Confirmation of CR and PR Required) | | |
|---|---|---|
| **Overall Response First Time Point** | **Overall Response Subsequent Time Point** | **Best Overall Response** |
| CR | CR | CR |
| CR | PR | SD, PD OR PR[a] |
| CR | SD | SD provided minimum criteria for SD duration met, otherwise, PD |
| CR | PD | SD provided minimum criteria for SD duration met, otherwise, PD |



Approved v1.0 930137673 4.0

| Table 2.3.3-1: Best Overall Response (Confirmation of CR and PR Required) | | |
|---|---|---|
| **Overall Response First Time Point** | **Overall Response Subsequent Time Point** | **Best Overall Response** |
| CR | NE | SD provided minimum criteria for SD duration met, otherwise, NE |
| PR | CR | PR |
| PR | PR | PR |
| PR | SD | SD |
| PR | PD | SD provided minimum criteria for SD duration met, otherwise, PD |
| PR | NE | SD provided minimum criteria for SD duration met, otherwise, NE |
| NE | NE | NE |
| CR = complete response, PR = partial response, SD = stable disease, PD = progressive disease, and NE = inevaluable | | |

[a] If a CR is truly met at first time point, then any disease seen at a subsequent time point, even disease meeting PR criteria relative to baseline, makes the disease PD at that point (since disease must have reappeared after CR). Best response would depend on whether minimum duration for SD was met. However, sometimes 'CR' may be claimed when subsequent scans suggest small lesions were likely still present and in fact the patient had PR, not CR at the first time point. Under these circumstances, the original CR should be changed to PR and the best response is PR.

### *2.3.4 Confirmation Scans*

**Verification of Response:** To be assigned a status of CR or PR, changes in tumor measurements must be confirmed by consecutive or subsequent repeat assessments that should be performed no less than 28 days after the criteria for response are first met. Subsequent documentation of a CR may provide confirmation of a previously identified CR even with an intervening NE or PR (eg, CR NE CR or CR PR CR). Subsequent documentation of a PR may provide confirmation of a previously identified PR even with an intervening NE or SD (eg, PR NE PR or PR SD PR). However, only one (1) intervening time point will be allowed between PR/CRs for confirmation.

**Verification of Progression**: Progression of disease should be verified in cases where progression is equivocal. If repeat scans confirm PD, then progression should be declared using the date of the initial scan. If repeat scans do not confirm PD, then the participant is considered to not have progressive disease.

## REFERENCES

1 Eisenhauer EA, Therasse P, Bogaerts J, et al. New response evaluation criteria in solid tumors: revised RECIST guideline (version 1.1). Eur J Cancer 2009; 45: 228-47.



Approved v1.0 930137673 4.0

# APPENDIX 8 AJCC MELANOMA STAGING

[From AJCC Cancer Staging Manual, 8th Edition (pages 577 & 578)]

## Definition of Primary Tumor (T)

| T Category | Thickness | Ulceration Status |
|---|---|---|
| **TX:** primary tumor thickness cannot be assessed (e.g., diagnosis by curettage) | Not applicable | Not applicable |
| **T0:** no evidence of primary tumor (e.g., unknown primary or completely regressed melanoma) | Not applicable | Not applicable |
| **Tis** (melanoma *in situ*) | Not applicable | Not applicable |
| **T1** | ≤1.0 mm | Unknown or unspecified |
| T1a | <0.8 mm | Without ulceration |
| T1b | <0.8 mm<br>0.8-1.0 mm | With ulceration<br>With or without ulceration |
| **T2** | >1.0-2.0 mm | Unknown or unspecified |
| T2a | >1.0-2.0 mm | Without ulceration |
| T2b | >1.0-2.0 mm | With ulceration |
| **T3** | >2.0-4.0 mm | Unknown or unspecified |
| T3a | >2.0-4.0 mm | Without ulceration |
| T3b | >2.0-4.0 mm | With ulceration |
| **T4** | >4.0 mm | Unknown or unspecified |
| T4a | >4.0 mm | Without ulceration |
| T4b | >4.0 mm | With ulceration |

## Definition of Distant Metastasis (M)

| M Category | Anatomic site | LDH level |
|---|---|---|
| **M0** | No evidence of distant metastasis | Not applicable |
| **M1** | Evidence of distant metastasis | See below |
| **M1a** | Distant metastasis to skin, soft tissue including muscle, and/or non-regional lymph node | Not recorded or unspecified |
| M1a(0) | | Not elevated |
| M1a(1) | | Elevated |
| **M1b** | Distant metastasis to lung with or without M1a sites of disease | Not recorded or unspecified |
| M1b(0) | | Not elevated |
| M1b(1) | | Elevated |
| **M1c** | Distant metastasis to non-CNS visceral sites with or without M1a or M1b sites of disease | Not recorded or unspecified |
| M1c(0) | | Not elevated |
| M1c(1) | | Elevated |
| **M1d** | Distant metastasis to CNS with or without M1a, M1b, or M1c sites of disease | Not recorded or unspecified |
| M1d(0) | | Not elevated |
| M1d(1) | | Elevated |
| Suffixes for M category: (0) LDH not elevated; (1) LDH elevated. No suffix is used if LDH is not recorded or is unspecified. | | |



Approved v1.0 930137673 4.0

## Definition of Regional Lymph Node (N)

| N Category | Number of tumor-involved regional lymph nodes | Presence of in-transit, satellite, or microsatellite metastases |
|---|---|---|
| **NX** | Regional nodes not assessed (e.g., SLN biopsy not performed, regional nodes previously removed for another reason)<br>**Exception**: pathological N category is not required for T1 melanomas, use cN | No |
| **N0** | No regional metastases detected | No |
| **N1** | One tumor-involved node or in-transit, satellite, and/or microsatellite metastases with no tumor-involved nodes | |
| N1a | One clinically occult (i.e., detected by SLN biopsy) | No |
| N1b | One clinically detected | No |
| N1c | No regional lymph node disease | Yes |
| **N2** | Two or three tumor-involved nodes or in-transit, satellite, and/or microsatellite metastases with one tumor-involved node | |
| N2a | Two or three clinically occult (i.e., detected by SLN biopsy) | No |
| N2b | Two or three, at least one of which was clinically detected | No |
| N2c | One clinically occult or clinically detected | Yes |
| **N3** | Four or more tumor-involved nodes or in-transit, satellite, or microsatellite metastases with two or more tumor-involved nodes, or any number of matted nodes with or without in-transit, satellite, and/or microsatellite metastases | |
| N3a | Four or more clinically occult (i.e., detected by SLN biopsy) | No |
| N3b | Four or more, at least one of which was clinically detected, or presence any number of matted nodes | No |
| N3c | Two or more clinically occult or clinically detected, and/or presence any number of matted nodes | Yes |



Approved v4.0 930137673 4.0

## AJCC Prognostic Stage Groups

Clinical (cTNM)

Clinical stage includes microstaging of the primary melanoma and clinical/radiologic/biopsy evaluation for metastases. By convention, clinical staging should be used after biopsy of the primary melanoma, with clinical assessment for regional and distant metastases. Note that pathological assessment of the primary melanoma is used for both clinical and pathological classification. Diagnostic biopsies to evaluate possible regional and/or distant metastasis also are included. Note there is only one stage group for clinical Stage III melanoma.

| When T is.... | And N is..... | And M is.... | The clinical stage is... |
|---|---|---|---|
| Tis | N0 | M0 | 0 |
| T1a | N0 | M0 | IA |
| T1b | N0 | M0 | IB |
| T2a | N0 | M0 | IB |
| T2b | N0 | M0 | IIA |
| T3a | N0 | M0 | IIA |
| T3b | N0 | M0 | IIB |
| T4a | N0 | M0 | IIB |
| T4b | N0 | M0 | IIC |
| Any T, Tis | ≥N1 | M0 | III |
| Any T | Any N | M1 | IV |



Approved v1.0 930137673 4.0

## PATHOLOGICAL (PTNM)

Pathological staging includes microstaging of the primary melanoma, including any additional staging information from the wide excision (surgical) specimen that constitutes primary tumor surgical treatment and pathological information about the regional lymph nodes after SLN biopsy or therapeutic lymph node dissection for clinically evident regional lymph node disease.

| When T is.... | And N is..... | And M is.... | The pathological stage is... |
|---|---|---|---|
| Tis | N0 | M0 | 0 |
| T1a | N0 | M0 | IA |
| T1b | N0 | M0 | IA |
| T2a | N0 | M0 | IB |
| T2b | N0 | M0 | IIA |
| T3a | N0 | M0 | IIA |
| T3b | N0 | M0 | IIB |
| T4a | N0 | M0 | IIB |
| T4b | N0 | M0 | IIC |
| T0 | N1b, N1c | M0 | IIIB |
| T0 | N2b, N2c, N3b or N3c | M0 | IIIC |
| T1a/b-T2a | N1a-N2a | M0 | IIIA |
| T1a/b-T2a | N1b/c or N2b | M0 | IIIB |
| T2b/T3a | N1a-N2b | M0 | IIIB |
| T1a-T3a | N2c or N3a/b/c | M0 | IIIC |
| T3b/T4a | Any N ≥N1 | M0 | IIIC |
| T4b | N1a-N2c | M0 | IIIC |
| T4b | N3a/b/c | M0 | IIID |
| Ant T, Tis | Any N | M1 | IV |
| Pathological Stage 0 (melanoma *in situ*) and T1 do not require pathological evaluation of lymph nodes to complete pathological staging; use cN information to assign their pathological stage. | | | |



Approved v1.0 930137673 4.0

# APPENDIX 9 COUNTRY SPECIFIC REQUIREMENTS

Criteria for exclusion of HIV-positive participants in Argentina, Czech Republic, Germany, and any other countries where exclusion of HIV-positive participants is locally mandated.

| Protocol Section | Revised Protocol Text |
|---|---|
| Table 2-1 Screening Procedural Outline (CA20976K) | Add "HIV testing" to list of laboratory tests. |
| Section 6.2 Exclusion Criteria, 1) Medical Conditions g) | Replace "Known history of testing positive for human immunodeficiency virus (HIV) or known acquired immunodeficiency syndrome (AIDS). Testing for HIV must be performed at sites mandated by local requirements" with "Positive test for HIV" |
| Section 9.4.4 Clinical Safety Laboratory Assessments | Replace "Testing for HIV-1 and HIV-2 must be performed at sites where mandated by local requirements" with "Testing for HIV-1 and HIV-2 must be performed." |



Approved v1.0 930137673 4.0

## APPENDIX 10 PROTOCOL AMENDMENT SUMMARY OF CHANGE HISTORY

**Overall Rationale for the Revised Protocol 02, 15-Oct-2021**

The protocol has been modified to align the management of adverse events (AEs) in trial participants, as well as the reporting of such AEs, per the Common Terminology Criteria for Adverse Events (CTCAE) version 5.0. The modification has been made since AE management in the previous version of the protocol (Revised Protocol 01, dated 16-Oct-2020) based on CTCAE v4 has been updated to CTCAE v5.

[redacted]

Other clarifications and editorial updates have been made throughout the protocol to improve clarity and readability and to keep consistency throughout the document.

This protocol amendment applies to all participants.

| **Summary of key changes for Protocol Amendment 02** | | |
|---|---|---|
| **Section Number and Title** | **Description of Change** | **Brief Rationale** |
| Title Page | Updated Medical Monitor information. | Administrative update. |
| Synopsis: Key Inclusion Criteria | • Updated criterion b) to indicate that in case 12 weeks between complete resection and randomization is exceeded due to unforeseen circumstances, the eligibility should be discussed with the Sponsor.<br>• Updated criterion g) to indicate that the eligibility should be discussed with the Sponsor in case the required tumor tissue content cannot be provided. | Additional text inserted to align these key criteria with criteria in the main body. |

| Summary of key changes for Protocol Amendment 02 | | |
|---|---|---|
| **Section Number and Title** | **Description of Change** | **Brief Rationale** |
| Synopsis: Key Exclusion Criteria - Prior/Concomitant Therapy<br>Section 6.2.1: Exclusion Criteria - Blinded Nivolumab or Placebo Treatment | In criterion 2)b), added "radiation therapy" against the resected melanoma, as an example of an excluded treatment, amongst other excluded therapies. | Radiation therapy directed against the resected melanoma could confound the evaluation of recurrence. |
| | Updated criterion 2)d) to (1) replace the terminology of "botanical preparations" with "complementary medications", and (2) indicate that complementary medications are permitted if they are used as supportive care. | Terminology introduced to reflect the wider array of products that may be in use by trial participants. In addition, the use of such products is only a concern when used to treat the study entry melanoma, and not for supportive care purposes. |
| Table 2-1: Screening Procedural Outline CA20976K (Applies to All Participants: Blinded or Open-label Treatment)<br>Section 5.1.2: Optional On-Protocol Open-Label Nivolumab Treatment After First Recurrence, Monitoring for Disease Recurrence/Progression | "Review of Pathology Report" row in Table 2-1 and "Monitoring for Disease Recurrence/ Progression" subsection in Section 5.1.2, clarified that pathology reports sent to the central laboratory must be de-identified. | Clarification inserted to emphasize the importance of protecting participant privacy. |
| Table 2-2: On-Treatment Assessments CA20976K (Applies to All Participants: Blinded or Open-label Treatment)<br>Table 2-3: Long-Term Follow-Up CA20976K | In the "Adverse Event Assessment" row, edited text to clarify that the management and reporting of all AEs/serious adverse events (SAEs) is per CTCAE v5. | AE management in the previous version of the protocol based on CTCAE v4 has been updated to CTCAE v5. |



Approved v1.0 930137673 4.0

| Summary of key changes for Protocol Amendment 02 | | |
|---|---|---|
| **Section Number and Title** | **Description of Change** | **Brief Rationale** |
| (Applies to All Participants: Blinded or Open-label Treatment) | | |
| Table 2-3: Long-Term Follow-up CA20976K (Applies to All Participants: Blinded or Open-label Treatment) | Edited footnote "a" to indicate that Follow-up Visit 1 can be performed on the date of discontinuation if that date is greater than 37 days, instead of 42 days, from the last dose. | To correct the 42 days inserted in error. |
| Section 5.1.1: Nivolumab or Placebo Blinded Treatment | Clarified that 3 days was 72 hours in the "Treatment" section.<br>In the "In the event of disease recurrence" section, clarified that pathology reports of biopsies confirming recurrence submitted to the central vendor need to be de-identified. | To improve clarity.<br>Clarification inserted to emphasize the importance of protecting participant privacy. |
| Section 5.4: Scientific Rationale for Study Design | Updated text on the rationale for the 2:1 randomization. | Provided additional rationale (specifically, additional safety information on the experimental arm) for the 2:1 randomization design implemented in the trial. |
| Section 6.1.1: Inclusion Criteria - Blinded Nivolumab or Placebo Treatment | Added text to criterion 2)e)i) on addressing incidental findings (eg, indeterminate nodules noted on imaging).<br>Added text to criterion 3)b) that allows additional time, up to 72 hours prior to start of study treatment, to obtain a negative serum or urine pregnancy test. | Text inserted to provide sites with direction on handling incidental findings noted during the screening process.<br>Provide sites with additional flexibility especially where results cannot be obtained within the standard 24-hour window. |



Approved v1.0 930137673 4.0

| Summary of key changes for Protocol Amendment 02 | | |
|---|---|---|
| **Section Number and Title** | **Description of Change** | **Brief Rationale** |
| | Criteria 3)c)-3)e): Deleted the requirement for male participants to use contraceptive measures. | Based on fetal and maternal exposure studies in rabbit and cynomolgus monkey models, excretion of monoclonal antibodies (mAbs) into semen, and male-mediated mAb drug transfer via seminal fluid does not present a health risk to a female partner, and mAbs present in seminal fluid are not bioavailable to the developing conceptus. Studies in healthy male volunteers demonstrated that the risk to a fetus exposed to a mAb via seminal fluid transfer to a pregnant partner is negligible and any theoretical fetal exposure that might occur is highly unlikely to be pharmacologically relevant. |
| Section 6.2.1: Exclusion Criteria - Blinded Nivolumab or Placebo Treatment | In criterion 2)g), inserted clarification that the full dosing schedule of coronavirus disease 2019 (COVID-19) vaccines should be completed, unless a delay would compromise the participant's health or suitability for enrollment. | It is preferable for participants to complete the full dosing schedule of COVID-19 inactivated vaccines to allow for resolution of any vaccine-induced side effects prior to initiating study treatment. New limited safety data on inactivated mRNA vaccines (eg, BNT162b2) indicate no new immune-mediated side effects or exacerbations of existing immune-related side effects were observed in participants receiving concomitant checkpoint inhibitors and the BNT162b2 vaccine; therefore, allowance is made to accommodate participants who may not have completed their full COVID-19 vaccine schedule, if it is felt to be in the participant's best interest, after discussion with the Sponsor and the investigator. |
| Section 7: Treatment | Deleted text that placebo should be sourced by investigative sites if | Redundant, as this text already included in another paragraph. |

| Summary of key changes for Protocol Amendment 02 | | |
|---|---|---|
| **Section Number and Title** | **Description of Change** | **Brief Rationale** |
| | available and permitted by local regulations. | |
| Section 7.1.1: Treatment with Nivolumab or Placebo | Clarified that study treatment can be administered over approximately 30 minutes. | To align with infusion duration text in Section 5.1.1: Nivolumab or Placebo Blinded Treatment |
| | Inserted text to indicate that the target interval of 28 days for initiation of a subsequent cycle is always based on the actual date the study treatment was administered in the preceding cycle, unless the subsequent cycle needed to be delayed for safety reasons. | To provide clarity to sites that there should always be an interval of 28 days (± 3 days) between actual treatments, regardless of subsequent target visit dates that may have been charted out on calendar (for planning purposes) prior to or around the time of initiating the first study treatment. |
| Section 7.3.1: Unblinding: Emergency, Accidental, and in the Event of Disease Recurrence | Inserted text that in the case of accidental unblinding, the unblinded Clinical Trial Monitor assigned to the site should be contacted. | To prevent the inadvertent unblinding of blinded trial personnel (eg, the Medical Monitor or designee [Clinical Scientist]). |
| Section 7.3.2: Unblinding in the Event of Disease Recurrence | Inserted text that a member of the BMS study trial management team, in addition to a BMS site monitor, could assist the site with instructions related to unblinding the treatment arm through the Interactive Response Technology (IRT), in case of disease recurrence. | Added additional BMS support to ensure that sites can unblind a participant's treatment arm in a timely manner in the event of disease recurrence. |
| Section 7.4.1: Criteria to Delay, Resume, and Discontinue Nivolumab or Nivolumab-Placebo Criteria | Updated the section name from "Dose Delay Criteria" to "Criteria to Delay, Resume, and Discontinue Nivolumab or Nivolumab-Placebo". | The change was made since the criteria to delay, resume, and discontinue treatment were consolidated and reformatted into 1 section (Table 7.4.1-1) for better readability. |



Approved v1.0 930137673 4.0

| Summary of key changes for Protocol Amendment 02 | | |
|---|---|---|
| **Section Number and Title** | **Description of Change** | **Brief Rationale** |
| | Revised Protocol 01 Sections 7.4.3 (Criteria to Resume Treatment) and 8.1.1 (Discontinuation of Nivolumab or Nivolumab-Placebo) were moved to Section 7.4.1. | |
| | Updated text to clarify that the management and reporting of AEs will be based on CTCAE v5. | AE management in the previous version of the protocol based on CTCAE v4 was updated to CTCAE v5. |
| Section 7.4.2: Treatment of Nivolumab-Related Infusion Reactions | Deleted text that Grade 3 or 4 infusion reactions need to be reported within 24 hours to the BMS Medical Monitor/Study Director. | Redundant text, as sites are already directed to report Grade 3 or 4 infusion reactions within 24 hours as an SAE, if reporting criteria are met. |
| | Edited text to clarify that the management and reporting of infusion reactions is per CTCAE v5. | AE management in the previous version of the protocol based on CTCAE v4 was updated to CTCAE v5. |
| | Inserted a note that in the case of Grade 2 infusion reaction symptoms, if the infusion rate cannot be safely increased to the original infusion rate, then a reduced infusion rate of 50% of the original infusion rate or a reduced rate per local standards can be adopted for future infusions. | Flexibility provided to enable sites to manage subsequent infusion rates in participants who experience infusion reactions, in accordance with local practices. |
| Section 7.5: Preparation/Handling/Storage/Accountability | Updated text to clarify that the unblinded Clinical Trial Monitor assigned to the site should be contacted if there are concerns about the quality | To prevent the inadvertent unblinding of blinded trial personnel (eg, the Medical Monitor, Clinical Scientist, blinded site monitor, etc). |



Approved v4.0 930137673 4.0

| Summary of key changes for Protocol Amendment 02 | | |
|---|---|---|
| **Section Number and Title** | **Description of Change** | **Brief Rationale** |
| | or appearance of the study treatment. | |
| Section 7.6: Treatment Compliance | Deleted the following text: "Drug accountability should be reviewed by the site study staff at each visit." | Redundant text. |
| Section 7.7: Concomitant Therapy | Inserted text that non-live COVID-19 vaccination is considered a simple concomitant medication and that the safety and efficacy of non-live vaccines (including COVID-19 vaccines) in participants receiving nivolumab is unknown. | Non-live COVID-19 vaccines are considered a simple concomitant medication since a significant interaction between these vaccines and the study treatment is not anticipated. The safety and efficacy of non-live vaccines (including COVID-19 vaccines) in participants receiving nivolumab continues to evolve. |
| Section 7.7.1: Prohibited and/or Restricted Treatments | Updated text to (1) replace the terminology of "botanical preparations" with "complementary medications", and (2) indicate that complementary medications are permitted if they are used as supportive care. | Terminology introduced to reflect the wider array of products that may be in use by trial participants. In addition, the use of such products is only a concern when used to treat the study entry melanoma, and not for supportive care purposes. |
| Section 8.1: Discontinuation from Study Treatment | Inserted a cross-reference to Section 7.4.1 for details regarding discontinuation of nivolumab or nivolumab-placebo. | Reference to Section 7.4.1 inserted to direct the reader to Table 7.4.1-1 due to the reformatting of criteria to delay, resume, and discontinue study treatment. |
| Section 9.2.1: Time Period and Frequency for Collecting AE and SAE Information<br>Section 9.4: Safety | Edited text to clarify that the management and reporting of all AEs/SAEs is per CTCAE v5. | AE management in the previous version of the protocol based on CTCAE v4 was updated to CTCAE v5. |



Approved v1.0 930137673 4.0

| Summary of key changes for Protocol Amendment 02 | | |
|---|---|---|
| **Section Number and Title** | **Description of Change** | **Brief Rationale** |
| Section 9.4.4: Clinical Safety Laboratory Assessments | Added serum urea and phosphate as alternative laboratory assessments to blood urea nitrogen (BUN) and phosphorus, respectively. | Change made to accommodate local laboratory testing practices. |
| Table 9.5-1: Pharmacokinetic (PK) and Immunogenicity Sampling Collections for Blinded Nivolumab or Placebo-Treated Participants or Participants Treated with Open-Label Nivolumab After First Recurrence | Added ± 7-day window to Follow-up Visits 1 and 2. | A window of ± 7 days was added to be consistent with the ± 7-day window for these visits. |

[redacted]



Approved v1.0 930137673 4.0

| Summary of key changes for Protocol Amendment 02 | | |
|---|---|---|
| **Section Number and Title** | **Description of Change** | **Brief Rationale** |
| | [redacted] | |
| Section 10.1.2: Overall Survival | Clarified that a formal interim OS analysis is not planned at the time of RFS analysis (interim or final).<br>Inserted text to indicate that descriptive OS data will be provided at the time of significant RFS data (interim or final). In addition, the projected number of deaths at the time of the interim and final RFS analysis has been introduced. | Anticipated immaturity of OS data at the time of RFS analysis.<br>[redacted] |
| Section 10.3.4: Interim Analyses | Type I error updated for the interim and final RFS analyses. | Correction made to be consistent with Section 10.1.1: RFS. |
| Section 11: References | List was updated. | Based on updates/additions as per Protocol Amendment 02. |
| Appendix 1: Abbreviations and Trademarks | Abbreviations list was updated. | For consistency within the protocol. |
| Appendix 2: Study Governance Considerations | Appendix was updated with standard language. | To align with current standards. |
| Appendix 3: Adverse Events and Serious Adverse Events: | Appendix was updated with standard language. | To align with current standards. |



Approved v1.0 930137673 4.0

| Summary of key changes for Protocol Amendment 02 | | |
|---|---|---|
| **Section Number and Title** | **Description of Change** | **Brief Rationale** |
| Definitions and Procedures for Recording, Evaluating, Follow-up, and Reporting | | |
| Appendix 4: Women of Childbearing Potential Definitions and Methods of Contraception | Appendix was updated with standard language. | To align with current standards. |
| Appendix 5: Management Algorithms for Studies Under CTCAE Version 5.0 | Algorithms were updated. | To align with most current version. |
| Throughout | Removed mention of "Study Director" | This title is no longer used. |



Approved v1.0 930137673 4.0

## Overall Rationale for the Revised Protocol 01, 16-Oct-2020

[redacted] The protocol has been modified to incorporate this and other changes described below.

| Summary of key changes for Revised Protocol 01 | | |
|---|---|---|
| **Section Number and Title** | **Description of Change** | **Brief Rationale** |
| Synopsis: Key Inclusion Criteria | Made a clarification in inclusion criterion "b" to include wording for timing of sentinel lymph node negativity assessment as it relates to the 12-week window post complete resection with negative margins. | Due to the potential for geographic/institutional differences in the timing of sentinel lymph node assessment in relation to the wide local excision (WLE), the inclusion criterion was clarified that regardless of when the sentinel lymph node assessment was performed in relation to the surgery that documents the patient has negative margins, the 12-week clock is calculated from the time of this surgery to randomization. The clarification was provided to potentially reduce any bias in estimation of the RFS endpoint due to variability in sentinel lymph node assessment timing. |
| | Clarification was made to inclusion criterion "c" to indicate that imaging of extremities for resected melanomas located in the extremities is not a requirement and may be conducted per local standard of care. | The current protocol requires baseline body imaging of chest, abdomen, pelvis (CT-chest/abdomen/pelvis or CT-chest and MRI-abdomen/pelvis), and all known sites of resected disease (lymph nodes ≥ 15 mm in short axis). Since the imaging of extremities by CT/MRI for primary extremity melanomas that have been resected is not generally done per standard of care in this patient population, and not recommended by major international guidelines, the criterion was clarified to indicate that the imaging of extremities was not mandatory and that the baseline evaluation could be conducted and |



Approved v1.0 930137673 4.0

| Summary of key changes for Revised Protocol 01 | | |
|---|---|---|
| **Section Number and Title** | **Description of Change** | **Brief Rationale** |
| | | documented by the local standard of care. |
| | [redacted] | |
| Synopsis: Key Exclusion Criteria | To exclusion criterion "1.f", additional direction regarding the evaluation of participants with prior SARS-CoV-2 infection was added. | Exclusion criterion "1.f" excludes randomizing participants with serious or uncontrolled medical disorders. The text regarding the evaluation of participants with prior SARS-CoV-2 infection was added to provide further clarity to sites on the handling of these situations. |
| | To exclusion criterion "2.c" an exception was added that prior adjuvant treatment with interferon (for melanoma other than study entry melanoma) is allowed if completed ≥ 6 months prior to randomization. | Prior treatment with interferon is not anticipated in this study population. In some countries, low-dose adjuvant treatment with interferon is approved in melanomas above 1.5 mm, although, the use of this agent is anticipated to be rare. The current protocol does not allow for the use of any systemic anti-cancer therapy beyond surgical |



Approved v4.0 930137673 4.0

| Summary of key changes for Revised Protocol 01 | | |
|---|---|---|
| **Section Number and Title** | **Description of Change** | **Brief Rationale** |
| | | resection for the study entry melanoma. The prior use of this agent for a melanoma resected in the remote past is anticipated to have minimal to no impact on the evaluation of outcomes for the study entry melanoma. |
| Synopsis, Section 4: Objectives and Endpoints | The “Endpoint” for the Secondary Objective regarding the safety and toxicity of nivolumab monotherapy was updated to “Occurrence and severity of AEs as defined by NCI CTCAE v5.” | Clarified endpoint language to be reflective of the broader scope of safety and toxicity evaluation. |
| | [redacted] | |
| Synopsis: Study Design | Clarified language regarding timeframe for participation of placebo and nivolumab treatment-assigned patients to participate in the open-label portion of the trial in the event of recurrence on the blinded portion of the trial. | Editorially clarified the language to be consistent with text in the main body of the protocol. |
| | [redacted] | |



Approved v4.0 930137673 4.0

| Summary of key changes for Revised Protocol 01 | | |
|---|---|---|
| **Section Number and Title** | **Description of Change** | **Brief Rationale** |
| Synopsis: Number of Participants | Number of participants was changed from [redacted] to [redacted] | Updated to allow adequate minimum follow-up time at the interim analysis of primary endpoint. |
| Synopsis: Study Treatments | Table was updated to list the nivolumab potency that will be used. | Text updated to reflect the intent of the column, which is to list the nivolumab potency, and not the presentation. |
| Synopsis: Statistical Considerations | Statistical section was updated. | [redacted] |
| Synopsis: References | References were updated. | Omitted in error. |
| [redacted] | | |



Approved v1.0 930137673 4.0

**Summary of key changes for Revised Protocol 01**

| **Section Number and Title** | **Description of Change** | **Brief Rationale** |
|---|---|---|
| | [redacted] | [redacted] |
| | "Serious Adverse Event Assessment" row, inserted text that all AEs (SAEs or non-serious AEs) associated with SARS-CoV-2 infection will be collected from time of consent. | Text inserted to document, at Baseline, AEs attributed specifically to SARS-CoV-2 infection, thereby, facilitating the analysis and interpretation of downstream safety data during treatment and in follow-up. |
| | "Body Imaging" row added clarification in the "Notes" column that "imaging of extremities for resected melanomas located in the extremities is not a requirement and may be conducted per local standard of care." | The current protocol requires baseline body imaging of chest, abdomen, pelvis (CT-chest/abdomen/pelvis or CT-chest and MRI-abdomen/pelvis), and all known sites of resected disease (lymph nodes ≥ 15 mm in short axis). Since the imaging of extremities by CT/MRI for primary extremity melanomas that have been resected is not generally done per standard of care in this patient population, and is not recommended by major international guidelines, the criterion was clarified to indicate that the imaging of extremities was not mandatory and that the baseline evaluation could be conducted and documented by the local standard of care. |
| | [redacted] | [redacted] |
| Section 2, Table 2-2: On-Treatment Assessments CA20976K (Applies to All | "Targeted Physical Examination" row added "Pay special attention to skin and nodal examination." in the "Notes" column. | Emphasis on nodal examination, in addition to skin examination, was added to align with the intent of identifying local/regional recurrences per recommendations made by national guidelines (as stated in Section 5.4.10 |

| Summary of key changes for Revised Protocol 01 | | |
|---|---|---|
| **Section Number and Title** | **Description of Change** | **Brief Rationale** |
| Participants: Blinded or Open-label Treatment) | | "Rationale for Follow-up Clinic Visit Frequency"). |
| | "Adverse Events Assessment" row, inserted text that all AEs (SAEs or non-serious AEs associated with SARS-CoV-2 infection) will be collected continuously during treatment. | Text inserted to document, during treatment, AEs attributed specifically to SARS-CoV-2 infection, thereby, facilitating the analysis and interpretation of the overall safety data |
| | Introduced text that for reporting purposes, all AEs/SAEs are to be graded using CTCAE v5. However, for management, events must be re-graded using CTCAE v4. | The current toxicity management guidelines in the protocol are based on CTCAE v4 and will be updated in the future to CTCAE v5. |
| | "Body Imaging" row added clarification in the "Notes" column that "imaging of extremities for resected melanomas located in the extremities is not a requirement and may be conducted per local standard of care. Extremity imaging should be conducted in the event of a suspected locoregional relapse that is not clinically unequivocal." | The current protocol requires baseline body imaging of chest, abdomen, pelvis (CT-chest/abdomen/pelvis or CT-chest and MRI-abdomen/pelvis), and all known sites of resected disease (lymph nodes ≥ 15 mm in short axis). Since the imaging of extremities by CT/MRI for primary extremity melanomas that have been resected is not generally done per standard of care in this patient population, and is not recommended by major international guidelines, the criterion was clarified to indicate that the imaging of extremities was not mandatory and that the baseline evaluation could be conducted and documented by the local standard of care. In addition, the need to image the extremity was added to follow-up on a suspected locoregional relapse that is clinically equivocal. |
| | Also from "Body Imaging" row "Notes" column, deleted text regarding imaging in the open-label resectable disease arm (Arm 1) for participants in the | Stage IIB/C participants in the blinded portion of the trial who relapse will most likely have recurrences that are most advanced in Stage (III/IV), therefore, the text referring to |



Approved v4.0 930137673 4.0

| Summary of key changes for Revised Protocol 01 | | |
|---|---|---|
| **Section Number and Title** | **Description of Change** | **Brief Rationale** |
| | blinded portion of the trial who have recurrent disease other than Stage III/IV. | recurrences other than Stage III/IV was deleted. |
| | [redacted] | |
| Section 2, Table 2-3: Long-Term Follow-Up CA20976K (Applies to All Participants: Blinded or Open-label Treatment) | Introduced text that for reporting purposes, all AEs/SAEs are to be graded using CTCAE v5. However, for management, events must be re-graded using CTCAE v4.<br><br>Streamlined the AE/SAE section text. | The current toxicity management guidelines in the protocol are based on CTCAE v4 and will be updated in the future to CTCAE v5.<br><br>Text in this section was rearranged and streamlined to refer the reader to Section 9.2 (Adverse Events), where AE/SAE management (including details for AE/SAE handling for SARS-CoV-2 related infection) is described in greater detail. |
| | "Pregnancy Test" row, in the "Notes" column option to have increased pregnancy testing beyond Follow-Up Visits 1 and 2 was added. | Option added to comply with applicable local regulatory requirements for increased frequency of pregnancy testing. |
| | "Body Imaging" row added clarification in the "Notes" column that "imaging of extremities for resected melanomas located in the extremities is not a requirement and may be conducted per local standard of care. Extremity imaging should be conducted in the event of a suspected locoregional relapse that is not clinically unequivocal." | The current protocol requires baseline body imaging of chest, abdomen, pelvis (CT-chest/abdomen/pelvis or CT-chest and MRI-abdomen/pelvis), and all known sites of resected disease (lymph nodes ≥ 15 mm in short axis). Since the imaging of extremities by CT/MRI for primary extremity melanomas that have been resected is not generally done per standard of care in this patient population, and is not recommended by major international guidelines, the criterion was clarified to indicate that |



Approved v4.0 930137673 4.0

| Summary of key changes for Revised Protocol 01 | | |
|---|---|---|
| **Section Number and Title** | **Description of Change** | **Brief Rationale** |
| | | the imaging of extremities was not mandatory and that the baseline evaluation could be conducted and documented by the local standard of care. In addition, the need to image the extremity was added to follow-up on a suspected locoregional relapse that is clinically equivocal. |
| | Also from "Body Imaging" row "Notes" column, deleted text regarding imaging in the open-label resectable disease arm (Arm 1) for participants in the blinded portion of the trial who have recurrent disease other than Stage III/IV. | Stage IIB/C participants in the blinded portion of the trial who relapse will most likely have recurrences that are most advanced in Stage (III/IV), therefore, the text referring to recurrences other than Stage III/IV was deleted. |
| | [redacted] | |
| Section 5.1: Overall Design | Updated schema to reflect sample size change from [redacted] to [redacted] and accordingly adjusted the nivolumab arm | Change made to align with revised sample size. |

| Summary of key changes for Revised Protocol 01 | | |
|---|---|---|
| **Section Number and Title** | **Description of Change** | **Brief Rationale** |
| | sample size from [redacted] to [redacted] and placebo arm sample size from [redacted] to [redacted] | |
| Section 5.1.1: Nivolumab or Placebo Blinded Treatment | In “Screening”, clarified that tumor tissue should be shipped to central lab prior to randomization and removed language stating that “receipt of minimum tissue requirement will be assessed by central lab prior to randomization.” | Deleted requirement for the central lab to confirm the receipt of Screening tumor tissue prior to randomization to eliminate delays in randomization since the information evaluated by the central lab was not used for stratification purposes. |
| | In “Screening”, deleted text that participants should not have received any systemic therapy after the date that the submitted tumor tissue was obtained. | Redundant text that is already covered in the eligibility criteria. |
| | In “Treatment”, added text on “nodal examination” in addition to skin examination. | Emphasis on nodal examination, in addition to skin examination, was added to align with the intent of identifying local/regional recurrences per recommendations made by national guidelines (as stated in Section 5.4.10 “Rationale for Follow-up Clinic Visit Frequency”). |
| Section 5.1.2: Optional On-Protocol Open-Label Nivolumab Treatment After First Recurrence | [redacted] | |
| Section 5.1.3: Data Monitoring Committee and Other External Committees | Added text that a Study Steering Committee (SSC) has been established. | A SSC was selected to better guide the Sponsor on the scientific aspects of conducting the trial. |



Approved v1.0 930137673 4.0

| Summary of key changes for Revised Protocol 01 | | |
|---|---|---|
| **Section Number and Title** | **Description of Change** | **Brief Rationale** |
| Section 5.2: Number of Participants | Changed number of participants from [redacted] to [redacted] | Change made to align with revised sample size, allowing higher information fraction and adequate minimum follow-up at interim analysis of primary endpoint. |
| Section 5.4.11: Rationale for Continued Adjuvant Therapy for Patients who are Diagnosed with Malignant Melanoma in Situ During Study Treatment | New section added with language for continued study treatment for participants who develop a malignant melanoma in situ (MMIS) during treatment. | Added to continue study treatment without unblinding in the event the participant develops MMIS lesions due to the excellent prognosis of this lesion with appropriate treatment per standard of care. |
| Section 6.1.1: Inclusion Criteria - Blinded Nivolumab or Placebo Treatment | In criterion "2.a", inserted text that patients with Stage IIB/C melanoma who present with synchronous or metachronous Stage < 2 melanomas that have been completely resected will be allowed. | The inclusion criteria did not specifically address synchronous or metachronous melanomas. Patients with synchronous or metachronous melanomas that are below Stage II have an excellent 5-year melanoma-specific survival of greater than 95%, and the risk for melanoma recurrence is greater with the study entry Stage IIB/C melanoma; therefore, patients with synchronous or metachronous melanomas less than Stage II will be allowed on this trial. |
| | Clarified inclusion criterion "2.b" to include wording for timing of sentinel lymph node negativity assessment as it relates to the 12-week window post complete resection with negative margins. | Due to the potential for geographic/institutional differences in the timing of sentinel lymph node assessment in relation to the wide local excision (WLE), the inclusion criterion was clarified that regardless of when the sentinel lymph node assessment was performed in relation to the surgery that documents the patient has negative margins, the 12-week clock is calculated from the time of this surgery to randomization. The clarification was provided to potentially reduce any bias |

| Summary of key changes for Revised Protocol 01 | | |
|---|---|---|
| **Section Number and Title** | **Description of Change** | **Brief Rationale** |
| | | in estimation of the RFS endpoint due to variability in sentinel lymph node assessment timing. |
| | Clarified inclusion criterion "2.e" to indicate that imaging of extremities for resected melanomas located in the extremities is not a requirement and may be conducted per local standard of care. | The current protocol requires baseline body imaging of chest, abdomen, pelvis (CT-chest/abdomen/pelvis or CT-chest and MRI-abdomen/pelvis), and all known sites of resected disease (lymph nodes ≥ 15 mm in short axis). Since the imaging of extremities by CT/MRI for primary extremity melanomas that have been resected is not generally done per standard of care in this patient population, and is not recommended by major international guidelines, the criterion was clarified to indicate that the imaging of extremities was not mandatory and that the baseline evaluation could be conducted and documented by the local standard of care. |
| | [redacted] | |



Approved v1.0 930137673 4.0

| Summary of key changes for Revised Protocol 01 | | |
|---|---|---|
| **Section Number and Title** | **Description of Change** | **Brief Rationale** |
| | | [redacted] |
| Section 6.2.1: Exclusion Criteria - Blinded Nivolumab or Placebo Treatment | To exclusion criterion “1.f”, additional direction regarding the evaluation of participants with prior SARS-CoV-2 infection was added. | Exclusion criterion “1.f” excludes randomizing participants with serious or uncontrolled medical disorders. The text regarding the evaluation of participants with prior SARS-CoV-2 infection was added to provide further clarity to sites on the handling of these situations. |
| | To exclusion criterion “2.c” an exception was added that prior adjuvant treatment with interferon (for melanoma other than study entry melanoma) is allowed if completed ≥ 6 months prior to randomization. | Prior treatment with interferon is not anticipated in this study population. In some countries, low-dose adjuvant treatment with interferon is approved in melanomas above 1.5 mm, although, the use of this agent is anticipated to be rare. The current protocol does not allow for the use of any systemic anti-cancer therapy beyond surgical resection for the study entry melanoma. The prior use of this agent for a melanoma resected in the remote past is anticipated to have minimal to no impact on the evaluation of outcomes for the study entry melanoma. |
| | [redacted] | [redacted] |
| Section 6.4.1: Retesting During Screening or Lead-In Period | Inserted text that testing for asymptomatic COVID-19 infection, for example, by reverse transcription polymerase chain reaction (RT-PCR) or viral antigen is not | Testing by RT-PCR or viral antigens of all participants during Screening to identify asymptomatic infection with SARS-CoV-2 is not required due to the current limitations in molecular testing. Additional guidance on handling |



Approved v1.0 930137673 4.0

| Summary of key changes for Revised Protocol 01 | | |
|---|---|---|
| **Section Number and Title** | **Description of Change** | **Brief Rationale** |
| | required. In addition, inserted text to provide guidance on handling of participants who may develop suspected or confirmed symptomatic COVID-19 infection or be discovered to have asymptomatic COVID-19 infection during the Screening period. | participants who may develop suspected or confirmed symptomatic COVID-19 infection or be discovered to have asymptomatic COVID-19 infection during the Screening period has been inserted based on the Center for Disease Control (CDC) guidance for return to work for healthcare workers. |
| Section 7: Treatment | Table 7-1 was updated to list the nivolumab potency that will be used and the ‘Packaging’ and ‘Storage Conditions’ information was also updated. | Text updated to reflect the intent of the column, which is to list the nivolumab potency, and not the presentation, as well as the packaging and storage conditions. |
| Section 7.2.1: Blinded Nivolumab or Placebo Treatment | Removed language regarding receipt of minimum tissue requirement to be assessed by central lab prior to randomization. | Deleted requirement for the central lab to confirm the receipt of Screening tumor tissue prior to randomization to eliminate delays in randomization since the information evaluated by the central lab was not used for stratification purposes. |
| Section 7.4.1: Dose Delay Criteria | Added text that for dose delays, events will be reported per CTCAE v5, and re-graded using CTCAE v4 for management.<br>Added bullet point to delay dosing for SARS-CoV-2 infection (confirmed or suspected). | The current toxicity management guidelines in the protocol are based on CTCAE v4 and will be updated in the future to CTCAE v5.<br>Text added in the interest of ensuring trial participant safety. |
| Section 7.4.2: Treatment of Nivolumab-Related Infusion Reactions | Added text that infusion reactions will be reported per CTCAE v5, and re-graded using CTCAE v4 for management. | The current toxicity management guidelines in the protocol are based on CTCAE v4 and will be updated in the future to CTCAE v5. |
| Section 7.4.3: Criteria to Resume Treatment | Added text that AEs will be re-graded per NCI CTCAE v4 to determine resuming treatment. | The current toxicity management guidelines in the protocol are based on CTCAE v4 and will be updated in the future to CTCAE v5. |



Approved v1.0 930137673 4.0

| Summary of key changes for Revised Protocol 01 | | |
|---|---|---|
| **Section Number and Title** | **Description of Change** | **Brief Rationale** |
| | Added criteria to resume treatment for participants with suspected or confirmed SARS-CoV-2 infection. | Criteria added to provide guidance to sites on resuming study treatment for participants in whom study treatment was held due to suspected or confirmed SARS-CoV-2 infection. |
| Section 7.7.1: Prohibited and/or Restricted Treatments | Added section reference for permitted corticosteroid therapy. | To provide further information and direct the reader to the section for permitted corticosteroid therapy. |
| Section 7.8.1: Treatment Beyond Disease Progression | Section 7.8.1.1: Blinded Nivolumab/Placebo Participants and Participants Receiving Optional Open-Label Nivolumab Monotherapy After Recurrence (in Arm 1 [resectable disease]), and its associated text referring to the appearance of a melanoma in situ lesion as a progression event was deleted. | This section in the protocol is reserved for the classical pseudoprogression phenomenon reported with immunotherapy treatment in patients with unresectable/metastatic disease. The development of a MIS does not fit the definition of classic pseudoprogression, and hence the text has been deleted. |
| Section 8.1.1: Discontinuation of Nivolumab or Nivolumab-Placebo | Added text that for dose discontinuations, events will be reported per CTCAE v5, and re-graded using CTCAE v4 for management. | The current toxicity management guidelines in the protocol are based on CTCAE v4 and will be updated in the future to CTCAE v5. |
| | Reference to “Recurrence” in the context of “Treatment Beyond Disease Progression” was deleted from the reference to Section 7.8.1 in the first bullet point. | Update was made consequent to deleting Section 7.8.1.1 above. |
| | Modified text addressing study treatment discontinuations due to dose delays > 8 weeks for non-drug related reasons to include COVID-19 as a non-drug-related reason. | Added text to provide guidance to sites regarding continuing treatment in participants whose study treatment were delayed beyond 8 weeks from the previous dose due to COVID-19 related reasons. |



Approved v4.0 930137673 4.0

| Summary of key changes for Revised Protocol 01 | | |
|---|---|---|
| **Section Number and Title** | **Description of Change** | **Brief Rationale** |
| Section 9.1.1.1: Methods of Measurement | Language regarding imaging of extremities added/clarified as follows: Imaging of extremities for resected melanomas located in the extremities is not a requirement and may be conducted per local standard of care. Extremity imaging should be conducted in the event of a suspected locoregional relapse that is not clinically unequivocal. | The current protocol requires baseline body imaging of chest, abdomen, pelvis (CT-chest/abdomen/pelvis or CT-chest and MRI-abdomen/pelvis), and all known sites of resected disease (lymph nodes ≥ 15 mm in short axis). Since the imaging of extremities by CT/MRI for primary extremity melanomas that have been resected is not generally done per standard of care in this patient population, and is not recommended by major international guidelines, the criterion was clarified to indicate that the imaging of extremities was not mandatory and that the baseline evaluation could be conducted and documented by the local standard of care. In addition, the need to image the extremity was added to follow-up on a suspected locoregional relapse that is clinically equivocal. |
| Section 9.1.2.1: General Considerations for Determining Recurrence | The original title of Section 9.1.2.1, “Investigator Assessment of Recurrence (Nivolumab/Placebo Blinded Treatment and Open-Label Nivolumab - Arm 1)” was rearranged as Section 9.1.2.2, and Section 9.1.2.1 was renamed with the title “General Considerations for Determining Recurrence”. In addition, the content in each of these sections was rearranged. | Changes made for better flow and readability. |
| | New text added to Section 9.1.2.1 to provide direction regarding cytological and/or histological confirmation of melanoma recurrence. | Provide greater clarity on situations where cytological and/or histological confirmation or recurrence is mandatory. In addition, emphasized the need at attempting cytological and/or histological confirmation of all |



Approved v1.0 930137673 4.0

| Summary of key changes for Revised Protocol 01 | | |
|---|---|---|
| **Section Number and Title** | **Description of Change** | **Brief Rationale** |
| | | recurrences when clinically feasible, per regulatory agency feedback. |
| Section 9.1.2.4: Date of Recurrence | Added a new section to specify how the date of recurrence will be determined for the primary endpoint evaluation. | To add clarity. |
| [redacted] | | |
| Section 9.2.1: Time Period and Frequency for Collecting AE and SAE information | Introduced text that for reporting purposes, all AEs/SAEs are to be graded using CTCAE v5. However, for management, events must be re-graded using CTCAE v4. | The current toxicity management guidelines in the protocol are based on CTCAE v4 and will be updated in the future to CTCAE v5. |
| | Separated AE/SAE reporting requirements for the Blinded Nivolumab/Placebo portion and the Open-Label portion. | Change made for better readability. |
| | Inserted text that all AEs (SAEs or nonserious AEs) related to SARS-CoV2 infection will be collected during the blinded nivolumab/placebo portion of the trial, as well as the open-label portion of the trial, with reporting intervals for each of these portions. | Text inserted to outline the collection time period for AEs attributed specifically to SARS-CoV-2 infection, to better facilitate the analysis and interpretation of the overall safety data. |
| | In the blinded portion, inserted language that new-onset serious AEs do not need to be reported beyond 100 days of discontinuation of study treatment, if subsequent anti-cancer therapy is initiated, | Initiation of subsequent anti-cancer therapy could confound the evaluation/attribution of the serious AE to CA20976K study therapy. An option, however, is still provided to report the event if it is felt to be related to the CA20976K study therapy to |

| Summary of key changes for Revised Protocol 01 | | |
|---|---|---|
| **Section Number and Title** | **Description of Change** | **Brief Rationale** |
| | unless the event was felt to be related to the CA20976K study treatment. In addition, inserted text that nonserious treatment-related AEs do not need to be reported with an onset date on or after initiation of subsequent anti-cancer therapy. For the open-label portion clarified that nonserious AEs do not need to be reported beyond 100 days of discontinuation of study treatment. | completely capture the SAE profile of the study treatment. |
| Section 9.2.2: Method of Detecting AEs and SAEs | Deleted text regarding the frequency of reporting nonserious AEs, as well as reference to the Schedule of Assessments and Long-term Follow-up tables for further details. | AE/SAE reporting details have been captured in Section 9.2.1; hence, text was deleted to reduce redundancy. |
| Section 9.2.3: Follow-up of AEs and SAEs | Inserted text stating that follow-up and recording of nonserious AEs and SAEs applies to AEs related to SARS-CoV-2 infection as well. | Text added for clarification that SARS-CoV-2 associated AEs will be collected during follow-up and to align with text in earlier safety sections. |
| Section 9.4: Safety | Updated text to state that AEs, including laboratory abnormalities, will be reported per NCI CTCAE v5, and re-graded using NCI CTCAE v4 for management. | The current toxicity management guidelines in the protocol are based on CTCAE v4 and will be updated in the future to CTCAE v5. |
| Section 9.5: Pharmacokinetic and Immunogenicity Assessments | Added text giving the nivolumab infusion and sampling timepoints. | Provide additional clarity to sites on the timing of End of Infusion blood draws to accommodate flushing of residual study treatment in intravenous (IV) lines. |
| | Table 9.5-1: Clarified footnote “c” regarding timing of sample draw for End of Infusion timepoint. | Provide additional clarity to sites on the timing of End of Infusion blood draws to accommodate flushing of residual |



Approved v1.0 930137673 4.0

| Summary of key changes for Revised Protocol 01 | | |
|---|---|---|
| **Section Number and Title** | **Description of Change** | **Brief Rationale** |
| | | study treatment in intravenous (IV) lines. |
| [redacted] | | |
| Section 9.8.1: Additional Research Collection | Inserted additional text regarding residual sample storage for additional research for US sites. In addition, updated Table 9.8.1-1 as well. | Text added to clarify coverage to ensure all sample types and derived materials are available for additional research. |
| [redacted] | | |



Approved v4.0 930137673 4.0

| Summary of key changes for Revised Protocol 01 | | |
|---|---|---|
| **Section Number and Title** | **Description of Change** | **Brief Rationale** |
| | [redacted] | |
| [redacted] | | |
| Section 10.1.2: Overall Survival (OS) | Adjusted the sample size from [redacted] to [redacted] participants.<br><br>[redacted]. | Sample size was adjusted from [redacted] to [redacted] participants to align with revised statistical parameters.<br><br>[redacted] |
| Section 10.3.1.2: Secondary Endpoint Analyses | Under 'Investigator-Assessed Outcomes on Next-Line Therapies', removed "These endpoints will be analyzed using a two-sided log-rank test at the overall significance level of 5% (two-sided) in all | No formal testing will be done on next-line therapy. |

| Summary of key changes for Revised Protocol 01 | | |
|---|---|---|
| **Section Number and Title** | **Description of Change** | **Brief Rationale** |
| | randomized participants" and "No multiplicity adjustment will be applied". | |
| [redacted] | | |
| Section 10.3.4: Interim Analyses | Updated the IA for RFS analysis to be conducted at increased number of events [redacted]<br>Removed language regarding OS interim analysis. | To ensure adequate maturity of the data at the time of IA.<br>OS interim analysis deleted due to data immaturity. |
| Appendix 1: Abbreviations and Trademarks | List updated with additional abbreviations used. | Newly added text in the protocol. |
| Appendix 5: Management Algorithms | Updated the algorithms and added the Myocardial algorithm. | Program updates. |
| All | Minor formatting and typographical corrections. | Minor, therefore have not been summarized. |



Approved v1.0 930137673 4.0
