# Supplementary material for: Adjuvant nivolumab in resected stage IIB/C melanoma: primary results from the randomized, phase 3 CheckMate 76K trial
Source: Nat Med. 2023 Oct 16;29(11):2835–43. doi: 10.1038/s41591-023-02583-2 (PMC10667090; doi:10.1038/s41591-023-02583-2)
Supplement: Supplementary file 2 — Reporting Summary [file 41591_2023_2583_MOESM2_ESM.pdf]

nature portfolio

Corresponding author(s): John M. Kirkwood

Last updated by author(s): YYYY-MM-DD

Reporting Summary

Nature Portfolio wishes to improve the reproducibility of the work that we publish. This form provides structure for consistency and transparency in reporting. For further information on Nature Portfolio policies, see our Editorial Policies and the Editorial Policy Checklist.

Statistics

For all statistical analyses, confirm that the following items are present in the figure legend, table legend, main text, or Methods section.

n/a	Confirmed	
☐	☒	The exact sample size (n) for each experimental group/condition, given as a discrete number and unit of measurement
☐	☒	A statement on whether measurements were taken from distinct samples or whether the same sample was measured repeatedly
☐	☒	The statistical test(s) used AND whether they are one- or two-sided<br>*Only common tests should be described solely by name; describe more complex techniques in the Methods section.*
☐	☒	A description of all covariates tested
☐	☒	A description of any assumptions or corrections, such as tests of normality and adjustment for multiple comparisons
☐	☒	A full description of the statistical parameters including central tendency (e.g. means) or other basic estimates (e.g. regression coefficient) AND variation (e.g. standard deviation) or associated estimates of uncertainty (e.g. confidence intervals)
☐	☒	For null hypothesis testing, the test statistic (e.g. F, t, r) with confidence intervals, effect sizes, degrees of freedom and P value noted<br>*Give P values as exact values whenever suitable.*
☒	☐	For Bayesian analysis, information on the choice of priors and Markov chain Monte Carlo settings
☒	☐	For hierarchical and complex designs, identification of the appropriate level for tests and full reporting of outcomes
☐	☒	Estimates of effect sizes (e.g. Cohen's d, Pearson's r), indicating how they were calculated

Our web collection on statistics for biologists contains articles on many of the points above.

Software and code

Policy information about availability of computer code

Data collection	Medidata Classic Rave (version 2022.3.2; New York, NY, USA) was used for data collection throughout this study.
Data analysis	All analyses were performed using SAS software (version 9.4; Cary, NC, USA).

For manuscripts utilizing custom algorithms or software that are central to the research but not yet described in published literature, software must be made available to editors and reviewers. We strongly encourage code deposition in a community repository (e.g. GitHub). See the Nature Portfolio guidelines for submitting code & software for further information.

Data

Policy information about availability of data

All manuscripts must include a data availability statement. This statement should provide the following information, where applicable:

- Accession codes, unique identifiers, or web links for publicly available datasets
- A description of any restrictions on data availability
- For clinical datasets or third party data, please ensure that the statement adheres to our policy

Qualified researchers may submit a proposal to access deidentified and anonymized datasets for this study to Bristol Myers Squibb. Data will be made available to researchers whose proposals are approved by the Independent Review Committee (Duke University), with available information dependent upon the individual request. The option to submit data requests as well as review criteria for data requests are available at https://vivli.org/ourmember/bristol-myers-squibb/.

Additional information on Bristol Myers Squibb's policy on data sharing may be found at https://www.bms.com/researchers-and-partners/clinical-trials-and-research/disclosure-commitment.html. The study protocol of CheckMate 76K is provided in the Supplementary Information.

Research involving human participants, their data, or biological material

Policy information about studies with human participants or human data. See also policy information about sex, gender (identity/presentation), and sexual orientation and race, ethnicity and racism.

Reporting on sex and gender	Both male and female patients were eligible for enrollment. Sex was self-reported. The number of male (n = 483) and female (n = 307) patients randomized in this study is reported in Table 1. Evaluations of recurrence-free survival in several prespecified subgroups, including subgroups defined by sex, are reported in Fig. 3.
Reporting on race, ethnicity, or other socially relevant groupings	There are no analyses based on race or ethnicity reported in this manuscript.
Population characteristics	Baseline patient demographics (including age, sex, geographic region) and disease characteristics for patients randomized in this study are reported in Table 1.
Recruitment	From October 2019 through November 2021, patients were recruited by investigators at 129 hospitals across 20 countries worldwide (Australia, Austria, Belgium, Canada, Czech Republic, Denmark, Finland, France, Germany, Greece, Italy, Netherlands, Norway, Poland, Romania, Spain, Sweden, Switzerland, United Kingdom, United States). Eligibility criteria and screening procedures minimized the potential of self-selection bias.
Ethics oversight	The full list of institutional review boards or ethics committees that approved the protocol and its amendments are provided in Supplementary Table 7.

Note that full information on the approval of the study protocol must also be provided in the manuscript.

Field-specific reporting

Please select the one below that is the best fit for your research. If you are not sure, read the appropriate sections before making your selection.

☒ Life sciences ☐ Behavioural & social sciences ☐ Ecological, evolutionary & environmental sciences

For a reference copy of the document with all sections, see nature.com/documents/nr-reporting-summary-flat.pdf

Life sciences study design

All studies must disclose on these points even when the disclosure is negative.

Sample size	An estimated sample size of 780 patients was planned to achieve the required 154 recurrence-free survival events to detect a statistically significant difference between the treatment arms at final analysis with at least 90% statistical power if the average hazard ratio of nivolumab versus placebo was 0.573, with a two-sided alpha of 0.05 by a stratified log-rank test. For the current reported prespecified interim analysis, approximately 123 events were planned (80% information fraction) with a critical hazard ratio of 0.65 and statistical power to detect a difference between arms of 62.8%. The stopping boundaries for both interim and final analyses were derived using Lan-DeMets alpha spending function with O'Brien-Fleming boundaries. A total of 790 patients were randomized and, at the data cutoff date of June 28, 2022, there were 135 reported recurrence-free survival events (88% information fraction) resulting in a critical hazard ratio of 0.678 and a 76.8% statistical power to detect a difference of recurrence-free survival between the arms.
Data exclusions	No data were excluded from the reported analyses.
Replication	Attempts of replication were not performed given that CheckMate 76K is a clinical trial.
Randomization	This study used stratified permuted block randomization. Enrolled patients were randomized 2:1 to receive nivolumab or placebo via interactive web response technology utilizing a randomization schedule; at randomization, patients were assigned the next available treatment arm in the schedule. Randomization was stratified according to AJCC 8th edition tumor (T)-category (T3b versus T4a versus T4b).
Blinding	The patients, investigators, and site staff were blinded to treatment arm assignments during the study. Unblinding of treatment arm assignment was permitted for patients who opted to receive optional, on-protocol, open-label nivolumab treatment following recurrence on either nivolumab (if at least 6 months from last treatment) or placebo (at any time after recurrence).

Reporting for specific materials, systems and methods

We require information from authors about some types of materials, experimental systems and methods used in many studies. Here, indicate whether each material, system or method listed is relevant to your study. If you are not sure if a list item applies to your research, read the appropriate section before selecting a response.

Materials & experimental systems

n/a	Involved in the study
☐	☒ Antibodies
☒	☐ Eukaryotic cell lines
☒	☐ Palaeontology and archaeology
☒	☐ Animals and other organisms
☐	☒ Clinical data
☒	☐ Dual use research of concern
☒	☐ Plants

Methods

n/a	Involved in the study
☒	☐ ChIP-seq
☒	☐ Flow cytometry
☒	☐ MRI-based neuroimaging

Antibodies

Antibodies used

Nivolumab, an anti-PD-1 monoclonal antibody, was administered as the experimental treatment in this study. Nivolumab administered as part of this study was provided by the study's sponsor (Bristol Myers Squibb).

Validation

Adjuvant nivolumab for patients with resected stage IIB/C melanoma was evaluated in this study as part of Bristol Myers Squibb's clinical study program.

Clinical data

Policy information about clinical studies

All manuscripts should comply with the ICMJE guidelines for publication of clinical research and a completed CONSORT checklist must be included with all submissions.

Clinical trial registration

NCT04099251

Study protocol

The study protocol is provided in the Supplementary Information.

Data collection

From October 2019 through November 2021, 986 patients in 20 countries worldwide (Australia, Austria, Belgium, Canada, Czech Republic, Denmark, Finland, France, Germany, Greece, Italy, Netherlands, Norway, Poland, Romania, Spain, Sweden, Switzerland, United Kingdom, United States) were screened at 129 study sites (hospitals), and 790 were randomized 2:1 to receive nivolumab (526 patients) or placebo (264 patients) at 119 hospitals. The data cutoff date for this prespecified interim analysis was June 28, 2022.

Outcomes

The primary endpoint for the trial was investigator-assessed RFS, defined as the time between randomization and the first recurrence event. Events included local, regional, or distant recurrence; new primary melanomas (including in situ); and death due to any cause. Patients could continue on blinded adjuvant treatment with a diagnosis of melanoma in situ, but not with an invasive new primary melanoma. Imaging was required every 26 weeks for the first 3 years and annually in years 4 and 5. Tumor assessments were performed using contrast-enhanced computerized tomography of the chest, abdomen, pelvis, and all other relevant sites based on known or suspected disease sites (slice thickness ≤5 mm, with no intervening gaps). Cytological and/or histological evidence of recurrence was required in all cases unless a biopsy was deemed to be clinically unsafe or not feasible by the Investigator. Secondary efficacy endpoints included DMFS (presented in this manuscript), defined as the time between randomization and first distant recurrence or death due to any cause, as well as OS and progression-free survival through next-line therapy (follow-up is ongoing, and those results are not presented in this manuscript). The secondary endpoint of safety for the blinded phase of the trial is presented here for treatment-related adverse events ≤30 days after last dose of study therapy, as well as for immune-mediated adverse events (non-endocrine events requiring immunomodulators and endocrine events, regardless of immune-modulating treatment) ≤100 days after last dose of study therapy. Time to onset and resolution data are also presented for immune-mediated adverse events. Occurrences and severity of adverse events were defined by National Cancer Institute's Common Terminology Criteria for Adverse Events version 5.

April 2023
